# Emergence of distinct syntenic density regimes is associated with early metazoan genomic transitions

**DOI:** 10.1186/s12864-022-08304-2

**Published:** 2022-02-17

**Authors:** Nicolas S. M. Robert, Fatih Sarigol, Bob Zimmermann, Axel Meyer, Christian R. Voolstra, Oleg Simakov

**Affiliations:** 1 Department of Neurosciences and Developmental Biology, University of Vienna, Althanstrasse 14, 1090 Wien, Austria; 2 Department of Biology, University of Konstanz, 78457 Constance, Germany

## Abstract

**Background:**

Animal genomes are strikingly conserved in terms of local gene order (microsynteny). While some of these microsyntenies have been shown to be coregulated or to form gene regulatory blocks, the diversity of their genomic and regulatory properties across the metazoan tree of life remains largely unknown.

**Results:**

Our comparative analyses of 49 animal genomes reveal that the largest gains of synteny occurred in the last common ancestor of bilaterians and cnidarians and in that of bilaterians. Depending on their node of emergence, we further show that novel syntenic blocks are characterized by distinct functional compositions (Gene Ontology terms enrichment) and gene density properties, such as high, average and low gene density regimes. This is particularly pronounced among bilaterian novel microsyntenies, most of which fall into high gene density regime associated with higher gene coexpression levels. Conversely, a majority of vertebrate novel microsyntenies display a low gene density regime associated with lower gene coexpression levels.

**Conclusions:**

Our study provides first evidence for evolutionary transitions between different modes of microsyntenic block regulation that coincide with key events of metazoan evolution. Moreover, the microsyntenic profiling strategy and interactive online application (Syntenic Density Browser, available at: http://synteny.csb.univie.ac.at/) we present here can be used to explore regulatory properties of microsyntenic blocks and predict their coexpression in a wide-range of animal genomes.

**Supplementary Information:**

The online version contains supplementary material available at 10.1186/s12864-022-08304-2.

## Background

Local gene order has been conserved across animal phyla over vast evolutionary time spans, and is referred to as microsynteny [1–5]. However, little is known about the loss of ancestrally inherited microsyntenies and the emergence of novel microsyntenies, due to genome rearrangements, gene gains and/or gene losses [1, 6–8]. Determining the node of microsynteny loss or emergence can provide insights into the evolution of animal genome architecture and the extent of microsynteny conservation.

It still remains unclear whether microsynteny is conserved due to functional constraints (e.g., *cis*-regulatory constraints, topological organization) [9–11], or if it is simply a result of low recombination rates (i.e. without functional significance for gene regulation). Only few studies have addressed the possible functional roles of microsyntenic blocks across the whole genome. There is increasing evidence that some evolutionarily conserved pairs of adjacent genes are maintained as gene regulatory blocks (GRBs) because of *cis*-regulatory constraints, i.e., the regulatory regions of a target gene are located within a so-called bystander, an unrelated neighboring gene [6, 9, 10]. In human GRBs, the expression of bystander and target genes does not correlate [12]. However, genes within microsyntenic blocks of at least three genes display a higher than expected coexpression in invertebrate genomes [13]. The regulatory constraints on the expression of conserved gene pairs are well characterized in some species [6, 12], whereas those on microsyntenic blocks comprising three or more genes are largely lacking, due to both missing genomic information to identify such regions as well as missing functional genomic data.

Gene density has been suggested as a proxy for inferring the level of coexpression of genes, as a positive correlation between gene proximity and gene coexpression has also been reported [14, 15]. Previous studies have shown that microsyntenic blocks can possess a gene density diverging from the genome average in some species. For instance, a genome-wide study of vertebrate amniotes showed that the conserved syntenic regions exhibited a lower gene density than the rest of the genome [16]. In contrast, the *Lrk* gene loci were shown to have a high gene density in wheat, barley, maize, and rice [17].

In this context, our objective was to conduct an exhaustive search among available animal genomes to profile the emergence of conserved microsyntenies across the animal tree of life and to investigate their genomic properties. We used comparative genomics approaches to determine the retention of gene density across orthologous microsyntenic blocks and, in conjuction with available developmental RNAseq data in a few phylogenetically dispersed animals, to study the impact of microsyntenic gene density on gene co-expression. Our results provide new insights into the evolution and functional significance of conserved microsyntenic genome architecture.

## Results & discussion

### Largest gain of microsynteny in the bilaterian and planulozoan ancestors

The increased taxonomic coverage of sequenced genomes allowed us to profile syntenic gains and losses across the metazoan tree. According to previous methods for synteny detection across distantly related animals [1, 2, 13], we define a microsyntenic block as a unit of three or more orthologous genes, each separated by up to five intervening genes (Supplementary Fig. 2A). As local order has been scrambled across the vast evolutionary distances [1, 18], we do not require the orthologs to exhibit conserved collinearity. Our focus on blocks of three or more orthologous genes allows for a dynamic capture of longer microsyntenic stretches and the threshold of five intervening genes minimizes the proportion of false positives [1].

**Fig. 1:**
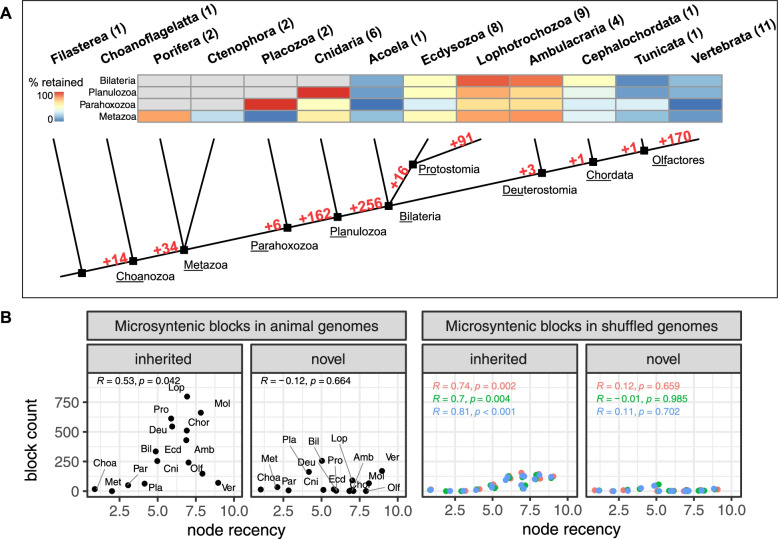
Microsynteny gains and losses along metazoan transitions. Microsyntenies indicated in the stems leading to key nodes. Relationships between major animal phyla based on [19], polytomy at the root of Metazoa and Bilateria due to the disputed position of Ctenophora [20], and Xenacoelomorpha [21, 22], respectively. Number of species surveyed for each group is indicated in brackets. The heatmap shows the percentage of novel blocks associated with the Metazoan, Parahoxozoan, Planulozoan and Bilaterian Last common ancestors that were retained in extant animals. **B** Block count in key nodes of the metazoan tree (topology from panel **A**), from animal genomes and shuffled genomes (3 sets), plotted as a function of node recency. Nodes are: Choanozoa (Choa), Metazoa (Met), Parahoxozoa (Par), Planulozoa (Pla), Bilateria (Bil), Cnidarian (Cni), Deuterostomia (Deu), Protostomia (Pro), Ecdysozoa (Ecd), Lophotrochozoa (Loph), Ambulacraria (Amb), Chordata (Chor), Olfactores (Olf), Mollusca (Mol), Vertebrata (Ver). The values of recency only reflect the relative ages of the nodes, based on [23]. Values are Pearson correlation coefficients (R) and their *p*-values (p)

Using the genomes of 49 metazoan species from 18 phyla (Supplementary Fig. 1) we reconstructed the ancestral complement of microsyntenic blocks throughout metazoan evolution (Fig. 1A). For every investigated node, we distinguished between inherited multi-species blocks (found in both the ingroup and the outgroup) and novel multi-species blocks (blocks found in the ingroup but not in the outgroup, see Supplementary Fig. 2B, and Methods). To assess false positive detection, we inferred microsyntenic blocks in three sets of shuffled genomes, i.e. genomes where the relative positions of genes have been randomly reshuffled (Methods).

We infer the largest gains of microsyntenic blocks to have occurred in the bilaterian last common ancestor (BLCA, 256 new blocks, only 45, 48 or 56 blocks found in shuffled genomes), and the planulozoan last common ancestor (PLCA, 162 new blocks, only 17, 19 or 25 in shuffled genomes) (Fig. 1B). In contrast, only 34 new blocks were found in the metazoan last common ancestor (MLCA, 0 in any shuffled genome). Thus, a substantial proportion (43%) of the microsyntenic blocks found in the BLCA emerged after the cnidarian-bilaterian split. Given the disputed position of the ctenophore and xenacoelomorpha phyla [21, 22, 24, 25], we calculated the amount of microsyntenic blocks novelties under various phylogenetic hypotheses. Regardless of the hypothesis, we obtained similar results regarding the counts of MLCA, PLCA and BLCA novel blocks (Supplementary Fig. 3). Accordingly, for every subsequent analysis, we used the set of blocks inferred using the multifurcating tree in Supplementary Fig. 1.

We next investigated the retention of microsyntenic blocks in extant species, defining a block to be lost if no species of a given clade possessed it in our analysis. The BLCA novelties have been largely retained among ambulacrarians and lophotrochozoans (Fig. 1A). Conversely, vertebrates lost 84% of the BLCA blocks, and ecdysozoans lost 50%. We observed medium to high percentages of lost BLCA novel blocks in acoel, tunicate, and cephalochordate (88, 95, and 51%, respectively). Since these loss rates are impacted by species sampling, the accuracy of these estimates will increase as more genomes become available for those clades. Nevertheless, the extensive loss of ancestral synteny in vertebrates can be explained by the whole genome duplications that were followed by paralog loss [26–29]. In ecdysozoans, this loss of synteny is explained by accelerated evolutionary rates involving a large number of chromosome fusions and rearrangements [30, 31] and extensive gene loss [32, 33].

It is expected that the number of genomic rearrangements between two taxa is proportional to the evolutionary time since common ancestry. In order to confirm that our estimations of the number of novel blocks were not biased by the timing of divergence, we correlated the inferred microsynteny block counts to phylogenetic node recency (a higher “recency” value means that the last common ancestor (LCA) is younger, Methods). In observed blocks, while the number of inherited blocks positively correlated with node recency (Pearson correlation coefficient 0.53, *p* < 0.05), the number of novel blocks did not (Fig. 1B), supporting the observation of extensive microsyntenic gains in the PLCA and BLCA. The positive correlation that we see between node recency and the associated number of inherited blocks in shuffled genomes can be explained by the increasing size of the outgroups, a larger outgroup leading to more blocks detected by chance. In most of the ancestors of the different metazoan lineages, the number of inherited blocks is several times lower in shuffled genomes. In contrast, the vertebrate LCA inherited only 70 blocks, less than the 110, 120 and 124 expected to occur by chance (Fig. 1B). The loss of microsyntenic blocks that followed the whole genome duplication events in vertebrates affected not only the BLCA novel microsyntenies, but even microsyntenic blocks inherited from older nodes, as exemplified by their loss of 91 and 85% of the MLCA and PLCA novelties, respectively (Fig. 1A).

Syntenic gains at various points in evolution were associated with different molecular functions. Among the 212 Gene Ontology (GO) terms enriched in the genes within the BLCA novel syntenies (Supplementary Table 1), some of the most significant ones were cell communication, signaling, and the establishment or maintenance of chromatin architecture (Methods, Supplementary Dataset 2). This contrasts with the 31 GO terms enriched in MLCA novel syntenies (Supplementary Table 3), mostly associated with the emergence of multicellularity, for example protein complex involved in cell adhesion, immune system process, and heterophilic cell-cell adhesion via plasma membrane cell adhesion molecules.

Taken together, our results demonstrate that the early evolutionary transitions towards bilaterally symmetrical animals [34] were associated with the emergence or higher retention of many microsyntenic blocks, as reflected in the substantial increase already in the planulozoan node (Fig. 1A). This is in strong contrast to the preceding phylogenetic transition and the emergence of multicellularity in the metazoan stem [35] which was associated with novel gene emergence [4, 36–38]. This suggests that different mechanisms of genomic innovation may underlie early animal evolutionary transitions.

**Fig. 2:**
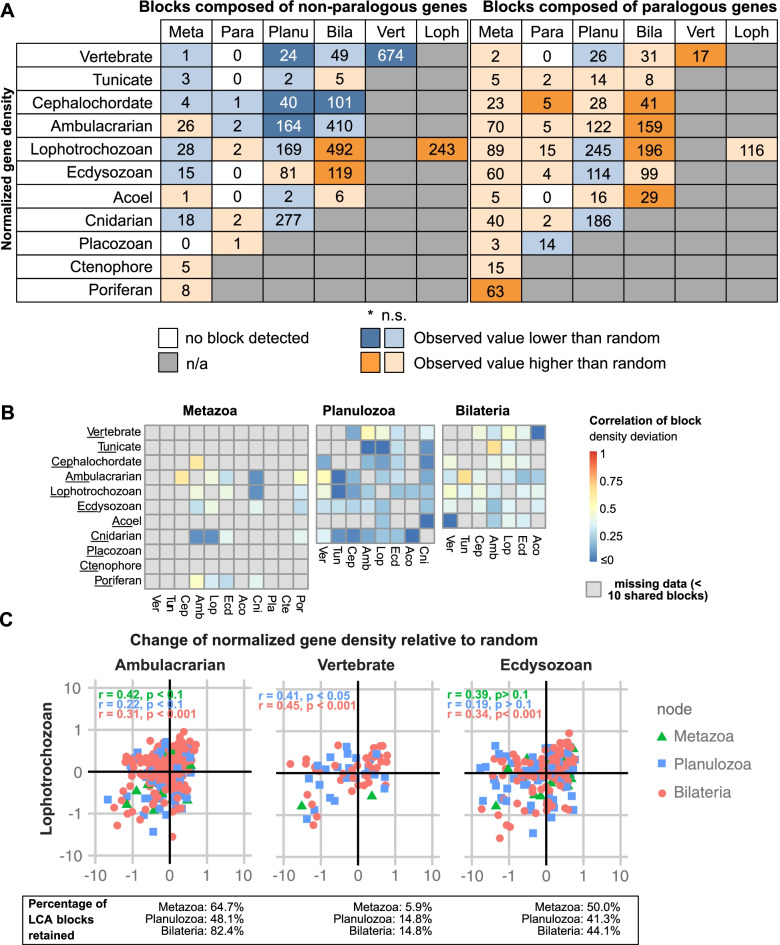
Gene density of microsyntenic novelties associated with key metazoan transitions and their scaling across bilaterians. **A** Number of microsyntenic blocks (including duplicates/split ones) inherited from key nodes (columns) found in extant metazoan taxa (rows). Colors note whether median normalized gene density of the block is higher in observed or randomly sampled blocks (see legend). Vivid colors note a statistically significant difference (*p* < 0.05, Wilcoxon rank-sum test). Left, non-paralogous blocks (less than 40% of syntenic genes belong to the same orthogroup); right, paralogous blocks (more than 40% of genes belong to the same orthogroup). Distributions of normalized gene density can be found in Supplementary Fig. 5. **B** Heatmaps showing the Spearman correlation coefficient of the relative deviation to random density of orthologous blocks, for every taxon pair sharing at least 10 blocks. Missing data is shown in grey. **C** Scatterplots displaying the relative change of normalized gene density (Methods) of blocks shared between lophotrochozoans and ambulacrarians, vertebrates or ecdysozoans blocks. For the novel MLCA, PLCA and BLCA blocks inherited by the aforementioned taxa, the correlation coefficients and p-values are indicated if the number of blocks shared is more than 10. The scatterplots for every possible taxon pair of our sample can be found in Supplementary Fig. 6

When investigating the functional significance of gene order, it is important to also consider the three-dimensional structure of the chromatin. Topologically associating domains or TADs, for example, are regions of the genome that show a high degree of internal physical interaction, but little to no physical interaction with their neighboring regions [39]. To date, much of the relationship between microsynteny and TADs remain unexplored, due to the lack of high-resolution HiC data for many invertebrate species, and only few syntenic regions have been described [11, 40]. Previous studies determined that bilaterians TAD formation is associated with CCCTC-binding factor (CTCF) proteins [39, 41]. The fact that CTCF protein could only be detected among bilaterians [42] suggests that the spatial organization of bilaterian chromatin differs from that of non-bilaterians. We hypothesize that distinct constraints on genome topology should be reflected in the syntenic signal. This is consistent with our observation that the evolutionary split between Cnidaria and Bilateria is associated with major changes of both the microsyntenic complement (Fig. 1A) and its genomic properties (Fig. 2). Accordingly, CTCF-dependent chromatin looping could be one of the forces underlying the maintenance of bilaterian microsyntenies [43], whereas the maintenance of PLCA novel blocks in extant cnidarians (devoid of CTCF) implies other constraints that predate the proposed emergence of CTCF.

### Distinct gene density properties of microsyntenic blocks

Next, we sought to describe the genomic properties of MLCA, PLCA, and BLCA novel microsyntenies across metazoans. Not only has the proximity of two genes been suggested to facilitate their coexpression [14, 15], but the topological organization of genomes also correlates with differences in local gene density in some species, e.g., in *Drosophila* the density of TAD interior and boundaries were shown to exhibit low and high gene density, respectively [44]. Therefore, gene density within microsyntenies may comprise a proxy measure for regulatory or topological properties. However, the comparison of block density across species is hampered by the differences in gene number and genome size, two parameters that are not tightly correlated in eukaryotes [45]. In order to account for differences in density across multiple genomes, we normalized block density (defined as the total number of protein-coding genes within a microsyntenic block, including intervening genes, divided by the block length) by the whole-genome density (total number of coding genes divided by the assembly size) (see Methods). This normalization was used because the reciprocal of gene density increases linearly with the assembly size (Supplementary Fig. 4). We term this value the “normalized gene density” within microsyntenic blocks. In order to determine whether normalized gene density in microsyntenic blocks differed from the rest of the genome, we compared it to that of randomly sampled blocks, i.e. regions of the genomes sampled based on the parameters of the observed blocks (number of syntenic genes, number of intervening genes; see Methods). As errors in coding exon predictions may affect our gene density estimates, we complemented this measure of the “normalized gene density” with the median intergenic distance between consecutive genes of each block (Supplementary Fig. 7).

Our analysis showed that syntenic blocks can have higher or lower normalized gene density and median intergenic distances than those of random blocks. We respectively term the blocks as following a high or a low density regime.

A major force that affects gene density regimes is tandem duplication, which can create gene-dense regions [46, 47]. To account for this, we classified microsyntenies into paralogous (more than 40% of genes are paralogs) and non-paralogous (40% or less of genes are paralogs) blocks. This threshold was chosen because it classifies a given block as paralogous already when at least two or three paralogs are present. Indeed, the tendency of paralogous blocks to show higher gene density regime and a lower median intergenic distance than random blocks was observed across every block. This is particularly prominent in BLCA novel microsyntenies (Fig. 2A, Supplementary Fig. 7).

Conversely, the density dynamics in non-paralogous microsyntenic blocks is more diverse. The normalized gene density and median intergenic distance of the MLCA and PLCA novelties retained in extant animals do not differ from the density of randomly sampled blocks (Fig. 2A, Supplementary Fig. 7A). Most BLCA non-paralogous microsyntenic blocks retained in ecdysozoans and lophotrochozoans, on the other hand, follow a high density regime. Not only is their density higher than that of random blocks (*p*-value Wilcoxon rank-sum test < 0.05, Fig. 2A, Supplementary Fig. 5), the intergenic distance of consecutive syntenic genes is also generally lower (Supplementary Fig. 7A, p-value Wilcoxon rank-sum test < 0.05). In addition, most lophotrochozoan novel blocks also follow a high gene density regime (Fig. 2A, Supplementary Fig. 5). Interestingly, most non-paralogous blocks that emerged in the stem leading to the last common ancestor of jawed vertebrates displayed a low density (Fig. 2A, Supplementary Fig. 5), and high intergenic distance (Supplementary Fig. 7), suggesting most of these blocks follow a low gene density regime. This result is consistent with the observation that the blocks conserved within amniotes exhibit a lower gene density than the rest of the genome [16].

Our data can be used to provide thresholds for estimating whether any block follows a high or low density regime, without sampling random blocks. The lower and higher quartiles of normalized gene density of the random blocks of amphioxus (*B. lanceolatum*, 1.47 and 1.84, respectively) and scallop (*M. yessoensis*, 1.34 and 1.98, respectively), provide tentative thresholds for high and low density regimes in those two species. Accordingly, if any block has a normalized gene density below 1, it is likely to follow a low density regime, whereas any block with a normalized gene density higher than 2 is more likely to follow a high density regime. To further facilitate exploration of the density regimes of microsyntenic blocks, we developed a new browser http://synteny.csb.univie.ac.at/.

In summary, paralogous blocks are in most cases more closely packed together than randomly sampled blocks, consistent with the fact that regions that underwent tandem duplications are associated with a higher gene density [46, 47]. Interestingly, the density of non-paralogous blocks relative to the rest of the genome differs between taxa (Fig. 2A). While most MLCA and PLCA novelties display average gene density, most of the BLCA novelties retained in lophotrochozoans and ecdysozoans exhibit a high density regime. A majority of lophotrochozoan novel microsyntenic blocks follow a high density regime as well, while the majority of jawed vertebrates microsyntenic blocks follow a low density regime.

### Maintenance of density regimes in orthologous blocks

We asked whether the observed difference in normalized gene densities is a result of contraction or expansion of microsyntenic blocks or from the preferential retention of blocks with high or low density.

In order to compare density regimes of orthologous blocks across taxa, we calculated the relative “change of normalized gene density” (CNGD, Methods). This value indicates whether blocks are likely to follow a low (CNGD << 0), high (CNGD > > 0) or average (CNGD ≈ 0) gene density regime. The comparison of all the possible taxon pairs revealed that the density regime of orthologous MLCA, PLCA and BLCA novel blocks were positively correlated in most cases (Fig. 2B, Supplementary Fig. 6), implying evolutionary conservation of gene density regimes.

As the density of most MLCA and PLCA novel blocks does not differ significantly from that of random blocks (Fig. 2A, Supplementary Fig. 5), yet correlates positively across taxa (Fig. 2B), we posit that these microsyntenic blocks could still experience selective pressures to maintain their density.

Since lophotrochozoans retained a particularly high number of the BLCA novel blocks (93%, Fig. 1B) they can be used as a reference to compare density regime conservation across all bilaterian clades. The majority of the lophotrochozoan BLCA novel blocks follow a high gene density regime (Fig. 2A, Supplementary Fig. 5). Moreover, lophotrochozoan BLCA blocks mostly follow the same density regimes as their orthologs in ambulacrarians (82.4% of BLCA blocks), vertebrates (14.8% of BLCA blocks) and ecdysozoans (44.1% of BLCA blocks) (Fig. 2B, C, Supplementary Fig. 8).

These results suggest that a substantial proportion of the microsyntenic blocks that emerged at the bilaterian stem followed a high density regime, and this ancestral regime was retained across extant taxa. In addition, most MLCA and PLCA blocks follow average genome density, a property conserved across taxa. The maintenance of density regimes across bilaterians suggests that most MLCA, PLCA and BLCA blocks did not undergo contraction or expansion in any lineages.

### Gene density predicts coexpression properties of microsyntenic genes

We next aimed to determine the impact of different microsyntenic density regimes onto gene expression. Given the proposed link between gene density and coexpression [14, 15], we hypothesized that denser microsyntenic blocks would exhibit a higher coexpression of their genes. To test this hypothesis, we used transcriptomic data (developmental series or tissues) from two lophotrochozoans (*Crassostrea gigas* and *Mizuhopecten yessoensis*), two ambulacrarians (*Strongylocentrotus purpuratus* and *Saccoglossus kowalevskii*) and two jawed vertebrates (*Mus musculus* and *Callorhinchus milii*). In line with a previous study [13], we define the coexpression levels of genes within a syntenic block as the mean Spearman correlation coefficient among all pairs of expressed genes of the block (which we will refer to as “block coexpression”, see Methods, Supplementary Fig. 2D).

**Fig. 3:**
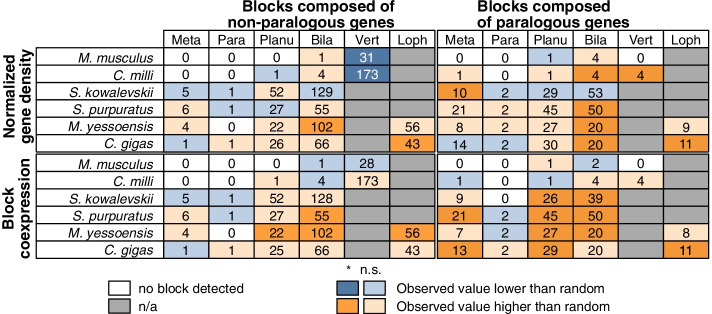
Higher coexpression in gene-dense microsyntenic blocks. Table noting the number of blocks inherited from key nodes (columns) that are found in bilaterian species (rows). The upper half of the table shows how the density of observed blocks differs from randomly sampled blocks, whereas the lower part shows how the block coexpression of observed microsyntenies differs from randomly sampled blocks. Colors highlight how the observed blocks with a median density/coexpression differs from that of randomly sampled blocks (see legend). Vivid colors note a significant difference (p-value < 0.05, Wilcoxon rank-sum test)

We then compared the coexpression of paralogous and non-paralogous blocks to that of randomly sampled blocks. We found that higher density blocks exhibit a higher coexpression than random blocks (Fig. 3, Supplementary Fig. 9). This suggests that the constraints to maintain the coexpression of genes drives the maintenance of high density blocks. Conversely, the block coexpression of vertebrate microsyntenic novelties, enriched in low density blocks, did not differ significantly from that of random blocks. As the expression of genes within vertebrate GRBs is not significantly correlated [12], this suggests that vertebrate microsyntenic novelties might be conserved more due to GRB [6, 9, 10, 12] rather than coexpression constraints.

**Fig. 4:**
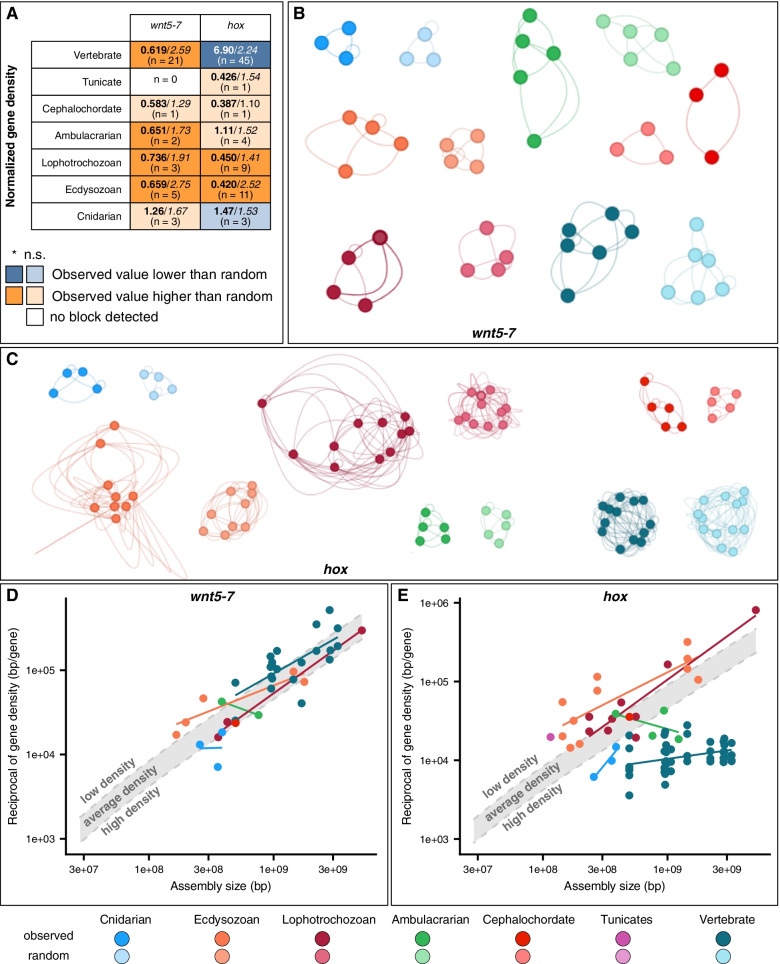
Gene density dynamics of Hox and Wnt5–7 microsyntenies across planulozoans. **A** Normalized gene density (observed/*random,* n is the number of blocks) in Wnt5–7 and Hox microsyntenic blocks (including duplicates and/or split ones) found in extant metazoan taxa (rows). Colors note whether median normalized gene density of the block is higher in observed or randomly sampled blocks (see legend). Vivid colors note a statistically significant difference (p < 0.05, Wilcoxon rank-sum test). Distributions of normalized gene density of both these blocks can be found in Supplementary Fig. 10. Wnt5–7 (**B**) and Hox (**C**) microsyntenic blocks displayed as graphs; nodes are gene families, edge lengths are the minimum normalized distance (distance in base pairs, normalized by assembly size) found between any given orthogroup pair within the taxonomic group. Self-edges are the minimum distance found between genes belonging to the same orthogroup. Reciprocal of raw gene density of Wnt5–7 (**D**) and Hox (**E**) as a function of assembly size, using a log-log scale. The upper and lower limits of the gray band correspond to the regression line explaining reciprocal gene density as a function of assembly size (Supplementary Fig. 3), and the regression line explaining reciprocal of the double of gene density as a function of assembly size, respectively. Both these values correspond to the tentative thresholds of 1 and 2 of the normalized gene density for identifying high and low gene density regimes, respectively

We investigated gene density and coexpression properties of microsyntenies to test their predictability of coregulation in several well studied microsyntenies. We first examined the Wnt5–7 block, which is comprised of two conserved syntenic pairs (*fbxl14-wnt5* and *atxn10-wnt7*, identified by [6]), as well as other known neighbors of *wnt5* and/or *wnt7* (e.g. *erc1/2*, *cacna2d*, [48, 49]). The Wnt5–7 block showed a normalized gene density significantly lower than that of randomly sampled blocks in most of the bilaterians we investigated (Fig. 4A, B, Supplementary Fig. 10A). Parsimony suggests that its gene density was low in the BLCA, and did not undergo contraction in any lineage. The fact that the reciprocal of gene density of the Wnt5–7 block scales at the same rate as the genome-wide average density, and is almost always of lower density than average (Fig. 4D) further supports this scenario. The presence of multiple functionally unrelated genes points to the absence of constraints on the coexpression of the block components. This is consistent with the hypothesis that *wnt7* and *atxn10* form a GRB [6]: The bystander gene (*atxn10*) contains regulatory elements in its introns that target the transdev gene (*wnt7*), resulting in a cis-regulatory constraint driving the conservation of their microsyntenic association.

Even though the vast majority of blocks retained their ancestral density regime across the investigated metazoans, there is one notable exception: the Hox cluster. While the Hox cluster shows a lower density than random blocks in most bilaterians (particularly significant in lophotrochozoans and ecdysozoans, Fig. 4A, C, Supplementary Fig. 10B), its density is significantly higher than random in vertebrates. In addition, the reciprocal of gene density of most invertebrate Hox clusters scales linearly with assembly size, at a similar rate as the genome-wide average, even though the density of these clusters is generally lower than the genome-wide average (Fig. 4E). In vertebrates, however, the Hox cluster does not scale at the same rate as the genome-wide average (Fig. 4E), and most Hox clusters are at least twice as dense as this average. Consistent with previous observations [50, 51], our findings show that the Hox cluster was less dense than the rest of the genome in the BLCA, and contracted before the vertebrate LCA.

Apart from coexpression, gene density regimes might also reflect changes in genome topology. As noted above, anecdotal evidence suggest that high- and low-density regions can be respectively associated with the boundaries and the interior of TADs in *Drosophila* [44]. Consistently, the gene-dense HoxD cluster of vertebrates is located at the interface of two TADs, while the low density amphioxus Hox cluster is located within a single TAD [11]. However, in order to test whether high-density microsyntenies are associated with specific topological properties, future efforts should be directed towards the generation of high resolution Hi-C data in a broad spectrum of invertebrate species. To this end, our density regimes of microsyntenies may be useful as validators and predictors of the topological properties.

## Conclusions

This study provides evidence that the evolutionary transition towards bilaterality in animals coincides with the emergence of a large set of microsyntenies, the majority of which are still retained in many extant cnidarian and bilaterian genomes. Our results suggest that microsyntenic blocks exist in different density regimes across animal genomes. In particular, syntenies that originated in the BLCA are generally associated with higher gene densities and display a higher level of gene coexpression than the remainder of the genome. While their topological organization remains to be investigated, this provides additional evidence for the proposed emergence of CTCF-driven topologically associating domain organization in bilaterians [42]. It should however be noted that a substantial amount of PLCA novel microsyntenies have been maintained in cnidarians, which implies the existence of additional constraints underlying the maintenance of microsyntenic blocks. We also reveal that the constraints on gene density within blocks are heterogeneous depending on the node of emergence of the blocks and that gene density is correlated with microsyntenic gene coexpression. In contrast, coexpression does not seem to drive the maintenance of low density synteny, hinting towards other types of gene regulation [6, 28]. Apart from the known distinct regulatory regimes in Hox clusters, little data exist to speculate about potential fundamental functional differences in how vertebrate and invertebrate genomes are regulated, constrained, and organized. Microsyntenic profiling together with further regulatory genomic information on topological organization and gene regulation across metazoans will thus help to better understand the regulatory causes or consequences of the ancient syntenic transitions during animal evolution.

## Methods

### List of software and scripts

List of software used can be found in the Additional File 1. The repository containing all the details on commands, scripts, and datasets used in this study at the time of submission is provided in pdf format as Supplementary Dataset 1. An online version is also available at https://bitbucket.org/N_Robert/syntenic-density-and-transitions/.

### Orthology assignment

Proteomes, annotations, and genomes of 38 species were downloaded from NCBI (*Homo sapiens*, *Mus musculus*, *Danio rerio*, *Gallus gallus*, *Xenopus tropicalis*, *Callorhinchus milii*, *Acropora millepora*, *Ciona intestinalis*, *Latimeria chalumnae*, *Maylandia zebra*, *Lepisosteus oculatus*, *Chelonia mydas*, *Parasteatoda tepidariorum*, *Exaiptasia pallida*, *Hippocampus comes*, *Acanthaster planci*), Ensembl Metazoa Release 45 (*Adineta vaga*, *Amphimedon queenslandica*, *Anopheles gambiae*, *Branchiostoma lanceolatum*, *Caenorhabditis elegans*, *Capitella teleta*, *Crassostrea gigas*, *Daphnia pulex*, *Drosophila melanogaster*, *Helobdella robusta*, *Ixodes scapularis*, *Lingula anatina*, *Lottia gigantea*, *Nematostella vectensis*, *Strigamia maritima*, *Strongylocentrotus purpuratus*, *Tribolium castaneum*, *Trichoplax adhaerens*, *Salpingoeca rosetta*, *Capsaspora owczarzaki*), or the National Human Genome Research Institute (*Hydra vulgaris*, *Mnemiopsis leidyi*). The *Clytia hemisphaerica* data was retrieved from http://marimba.obs-vlfr.fr. The *Aurelia aurita* genome, annotation and proteome were provided by the authors of [52]. The genome of *Hofstenia miamia* and its annotation were retrieved from http://srivastavalab.rc.fas.harvard.edu. The genome, proteome, and annotation of *Saccoglossus kowalevskii* and *Ptychodera flava* were downloaded from OIST (https://groups.oist.jp/molgenu/hemichordate-genomes), the ones of *Schmidtea mediterranea* from planmine (http://planmine.mpi-cbg.de), and the ones of *Hoilongia hongkongensis* from https://bitbucket.org/molpalmuc/hoilungia-genome/src/master/tracks/. The CDS sequences and genomes of *Pleurobrachia bachei* and *Sycon ciliatum* were retrieved, respectively, from Neurobase (https://neurobase.rc.ufl.edu), and DataDryad (https://datadryad.org/resource/doi:10.5061). The mapping of the transcripts of these two species onto their genomes was done using gmap. The genome and annotation of *Mizuhopecten yessoensis* was provided by the authors of [53]. *Euprymna scolopes* chromosome-level assembly and annotation was provided by the authors of Belcaid et al. [7]. The proteins of the 49 aforementioned species were assigned to orthogroups using the Orthofinder v2.3 algorithm [54], based on the results of an all-against-all BLASTP with an e-value threshold of 0.001. In order to avoid erroneous ortholog inference due to ambiguous tree topologies, all the genes within the same orthogroup were considered to be orthologs for all subsequent analyses.

### Inference of microsyntenic blocks and block emergence nodes

Syntenic blocks were inferred using in-house scripts, as described in [1, 2] (See Fig. 1A). These scripts can be found at https://github.com/nijibabulu/metazoan_synteny. All-against-all pairwise microsyntenic blocks were first built, requiring to comprise at least three orthologs, separated by no more than five intervening genes, but collinearity was not required. The minimum of three genes was chosen to increase sensitivity, as the use of scaffold-level assemblies do not allow the detection of large syntenic blocks. With a maximum of five intervening genes, the distribution of the number of intervening genes between consecutive orthologs in the block follows a power law [13]. This also limits the number of false positives, as an increase of the allowed number of intervening genes results in an increase in the number of false positives [1]. The pairwise syntenic blocks were then fused into multi-species blocks, if they shared at least three genes, or if there are more than three genes in the pairwise block, at least 50% of the orthologs. The multi-species microsyntenic blocks were then filtered according to the node where we inferred them to be present (Supplementary Fig. 2B). For each node, we define an ingroup as all the species descending from this node, and the outgroup as all the species outside of the ingroup. The children ingroups are one level above the nodes (e.g., acoel, deuterostomes and protostomes are the children clades of the LCA of Bilateria). We define a novel microsyntenic block as one found in two or more species of two or more children ingroup but not found in the outgroup (Supplementary Fig. 2B). An inherited microsyntenic block is a block found in at least two species of the ingroup and at least two species of the outgroup. In order to evaluate the number of blocks that we would detect by chance, we ran our microsynteny pipeline on three independent permutations of the genomes. We considered blocks to be lost in a taxonomic group if no species of the group was present in the multi species block. We used this method on the actual genomes and three iterations of shuffled genomes, where the relative position of genes on the genome was shuffled.

### Gene density analysis

The gene density within a given block was calculated as the number of genes per base pairs of a block. The boundaries of a block are defined as the outermost coding bases of genes exhibiting conserved synteny. This measure was used for the sake of consistency across all included species, as the coordinates of untranslated regions of the mRNAs were absent of some of the genome annotations we used. The number of genes includes all the genes of which both start and stop codons are located within the block boundaries. The gene density per block was then normalized by the gene density within the whole genome (total number of genes divided by total number of base pairs of the assembly). We refer to this value as normalized gene density. Since the reciprocal of whole genome density increases linearly with the assembly size (Supplementary Fig. 4) this normalization allows us to group normalized gene density measures from animals with different genome sizes according to their clade (see Supplementary Fig. 1).

We also calculated the median intergenic distance of consecutive syntenic orthologs. For each block, every distance (in base pairs) between consecutive orthologs was calculated (distance between boundaries of coding regions of the two genes), and the median value of each block was retained.

Each block was defined as non paralogous or paralogous. We define a paralogous block as comprising more than 40% of genes belonging to the same orthogroup (the region underwent several events of segmental duplications). Non paralogous blocks are all the other blocks that do not satisfy this criteria.

For each observed block, 100 blocks were randomly sampled. Randomly sampled blocks were built using the methods described in [13]. A detailed description of the methods can also be found in Supplementary Fig. 2C. Differences in the normalized gene density between the observed and randomly sampled blocks were assessed using a Wilcoxon rank-sum test and a threshold of significance of 0.05. For each multi species block found in at least two taxa, we also calculated the change of normalized gene density relative to random (CNGD), such as CNGD = (median(normalized_gene_density_obs_) – median(normalized_gene_density_rand_)) / median(normalized_gene_density_rand_). Accordingly, if the median normalized gene density of a multi species block (median(normalized_gene_density_obs_)) is twice as dense as that of their randomly sampled counterparts (median(normalized_gene_density_rand_))), this will result in CGND = 1. If the observed blocks are of a density that is half of the random blocks, this will result in CNGD = − 1. If the median normalized gene density of observed and randomly sampled blocks is identical, then CNGD = 0.

### Annotation of the genes of the Hox and Wnt5–7 clusters

If available, annotations of *hox* genes were recovered from the literature, and their orthologs in our taxonomic sample were identified by reciprocal BLAST searches and phylogenies. The *hox* queries were from *Latimeria chalumnae* [55], *Homo sapiens*, *Mus musculus*, *Danio rerio*, *Callorhinchus milii*, *Chelonia mydas*, *Ciona intestinalis*, *Strongylocentrotus purpuratus* [27, 56], *Euprymna scolopes* [7], *Hofstenia miamia* [57], *Schmidtea mediterranea* [58], *Nematostella vectensis* [59], *Caehnorhabditis elegans* [60], *Clytia hemisphaerica* [61], *Parasteatoda tepidariorum* [62], *Anopheles gambiae*, *Drosophila melanogaster*, *Tribolium castaneum*, *Strigamia maritima*, *Ixodes scapularis* [63], *Capitella teleta*, *Lottia gigantea*, *Daphnia pulex* [1], *Saccoglossus kowalevskii*, *Ptychodera flava* [2], *Mizuhopecten yessoensis* [53]. Among the microsyntenic blocks identified by our pipeline, one block (multi-species block id 953, see Supplementary Dataset 3) comprised the following genes: *wnt5, wnt7*, *fbxl14*, *atxn10*, *erc1/2*, *cacna1d*, *cacna2d ninj1/ninj2* and *dcp1a/dcp1b*. This block is referred to as the Wnt5–7 block throughout the manuscript. We identified the orthologs of the genes in all the species of our dataset by reciprocal BLAST searches and phylogenies. Only the Hox and Wnt5–7 clusters comprising at least three genes, separated by no more than five intervening genes were retained.

### Functional annotation and GO term enrichment analysis

Annotation and GO annotation of the proteomes of all species was done using eggNOG-mapper version 2 [64]. GO enrichment analysis was done using the GOAtools API [65]. The GO enrichment was done without propagating GO counts to parent GOs, using a threshold of 0.05 for Benjamini-Hochberg corrected *p*-values. GO terms enriched in the MLCA novel blocks were required to be enriched in at least eight metazoan species, distributed in at least two metazoan ingroups (i.e., Porifera, Ctenophora, and Parahoxozoa). GO terms enriched in the PLCA novel blocks were required to be enriched in at least three cnidarians and eight bilaterian species. GO terms enriched in the BLCA novel blocks were required to be enriched in at least four protostomes and four deuterostomes species. We provide the tables listing the GO terms enriched in MLCA (Supplementary Table 1), PLCA (Supplementary Table 2) and BLCA (Supplementary Table 3) blocks.

### Block coexpression analysis

The methods used for determining transcripts abundance in *Strongylocentrotus purpuratus*, *Saccoglossus kowalevskii*, *Mizuhopecten yessoensis* and *Crassostrea gigas* were described in Zieger et al. [66]. In line with this study, the transcript abundances in *Mus musculus* [67] and *Callorhinchus milii* [68] tissues were quantified as TPMs (Transcript per Kilobase per Million of reads) using Kallisto [69] with default settings for paired end reads. For each block, we calculated the block co expression value as described in Zimmerman et al. [13]. For each block comprising at least three expressed genes, we calculated the Spearman correlation of expression of every combination of expressed genes. To allow the averaging of correlations [70], the array of correlations of a given block was then transformed using the inverse hyperbolic tangent function (Fisher transformation), the mean of the Fisher-transformed correlation was then calculated, and this value was then transformed into a correlation using the hyperbolic tangent function (reverse Fisher transformation). In addition, if an untransformed correlation value was − 1 or 1, we transformed it into the next possible floating point value towards zero using the numpy.nextafter() function. This was done as the inverse hyperbolic tangent of 1 is infinite, and that of − 1 is negative infinite.

### Synteny browser

We introduce a novel browser containing our complete data set which allows users to investigate the syntenic density relationships of orthogroups with their genes of interest. In order to present a better visual comparison, we normalized the minimum base pair distances between each syntenic orthogroup pair of each multi-species block that we identified, by the total genome assembly size of each species. Our browser has four panels: while the first panel displays the boxplots of distance distributions for each taxonomic node, the second one displays the geometric densities of these; the third panel illustrates the orthogroups and the base pair distances between them in a network graph, and the fourth panel is a dictionary of all genes and their functions. Each panel is user-interactive and it is also possible to compare our observed values with the randomized cases. Users can first search for genes or annotations of their interest that exist in our dataset to find the multi-species block ID and the taxonomic group information, and then navigate to that block. Our browser is available at: http://synteny.csb.univie.ac.at/.

## Supplementary Information

**Additional file 1: Supplementary Figure 1.** List of software used and Supplementary Figures 1 to 10.

**Additional file 2: Supplementary Dataset 1.** Code and scripts from the repository.

**Additional file 3: Supplementary Dataset 2.** GO enrichment tables.

**Additional file 4: Supplementary Dataset 3.** Syntenic density database.

**Additional file 5: Supplementary Table 1.** Summary of GO terms enriched in MLCA blocks

**Additional file 6: Supplementary Table 2.** Summary of GO terms enriched in PLCA blocks.

**Additional file 7: Supplementary Table 3.** Summary of GO terms enriched in BLCA blocks.

## Data Availability

The repository containing all the details on commands, scripts, and datasets used in this study at the time of submission is provided in pdf format as Supplementary Dataset 1. An online version is also available at https://bitbucket.org/N_Robert/syntenic-density-and-transitions/.

## Abbreviations

GRB: Gene Regulatory Block
BLCA: Bilaterian Last Common Ancestor
PLCA: Planulozoan Last Common Ancestor
MLCA: Metazoan Last Common Ancestor
GO: Gene Ontology
TAD: Topologically Associating Domain
CTCF: CCCTC-binding factor
CNGD: Relative change of normalized gene density
TPM: Transcript per Kilobase per Million of reads
