# Supplementary material for: Emergence of distinct syntenic density regimes is associated with early metazoan genomic transitions
Source: BMC Genomics. 2022 Feb 17;23:143. doi: 10.1186/s12864-022-08304-2 (PMC8851819; doi:10.1186/s12864-022-08304-2)
Supplement: Supplementary file 1 — Additional file 1: Supplementary Figure 1. List of software used and Supplementary Figures 1 to 10. [file 12864_2022_8304_MOESM1_ESM.pdf]

Additional File 1 for

Emergence of distinct syntenic density regimes is associated with early metazoan genomic transitions

Nicolas S.M. Robert[1], Fatih Sarigol[1], Bob Zimmermann[1], Axel Meyer[2], Christian R. Voolstra[2], Oleg Simakov[1]

1. Department of Neurosciences and Developmental Biology, University of Vienna, Althanstrasse 14, 1090 Wien, Austria.

2. Department of Biology, University of Konstanz, 78457 Konstanz, Germany.

List of software used

Main Text and Figures, Supplementary Figures

BLAST+ suite 2.8.1 (https://ftp.ncbi.nlm.nih.gov/blast/executables/blast+/LATEST/)

Orthofinder 2.3.12 (https://github.com/davidemms/OrthoFinder)

mcl 14.137 (https://micans.org/mcl/)

homer 4.9 (http://homer.ucsd.edu/homer/motif/)

eggnoggmapper v2.0.0 (https://github.com/eggnogdb/eggnog-mapper)

grip v4.5.2 (https://github.com/joeyespo/grip)

Python 3.8.5 (https://www.python.org/)

Python API goatools (https://github.com/tanghaibao/goatools)

Python library numpy 1.19.4 (https://github.com/numpy/numpy)

Python library pandas 1.1.4 (https://github.com/pandas-dev/pandas)

Python library scipy 1.5.4 (https://github.com/scipy/scipy)

R version 4.0.2

R package dplyr 1.0.0 (https://github.com/tidyverse/dplyr)

R package tidyr 1.1.0 (https://github.com/tidyverse/tidyr)

R package tibble 3.0.3 (https://github.com/tidyverse/tibble)

R package readr 1.1.3 (https://github.com/tidyverse/readr)

R package ggplot2 3.3.2 (https://github.com/tidyverse/ggplot2)

R package ggpubr 0.4.0 (https://github.com/kassambara/ggpubr)

R package ggrepel 0.8.2 (https://github.com/slowkow/ggrepel)

R package RColorBrewer 1.1-2 (https://github.com/cran/RColorBrewer)

R package scales 1.1.1 (https://github.com/r-lib/scales)

R package cowplot 1.1.0 (https://github.com/wilkelab/cowplot)

R package gdata 2.18.0 (https://github.com/r-gregmisc/gdata)

R package pheatmap 1.0.12 (https://github.com/raivokolde/pheatmap)

R package gridExtra 2.3 (https://github.com/cran/gridExtra)

Online application (http://synteny.csb.univie.ac.at)

R version 3.6.0

R package ggplot2 (https://github.com/tidyverse/ggplot2)

R package plotly (https://github.com/ropensci/plotly)

R package visNetwork (https://github.com/datastorm-open/visNetwork)

R package Shiny (https://github.com/rstudio/shiny)

R package htmlwidgets (https://github.com/ramnathv/htmlwidgets)

R package Shiny Themes (https://github.com/rstudio/shinythemes)

R package DT (https://github.com/rstudio/DT)

R package dplyr (https://github.com/tidyverse/dplyr)

vis.js visualisation library (https://github.com/visjs-community/vis)

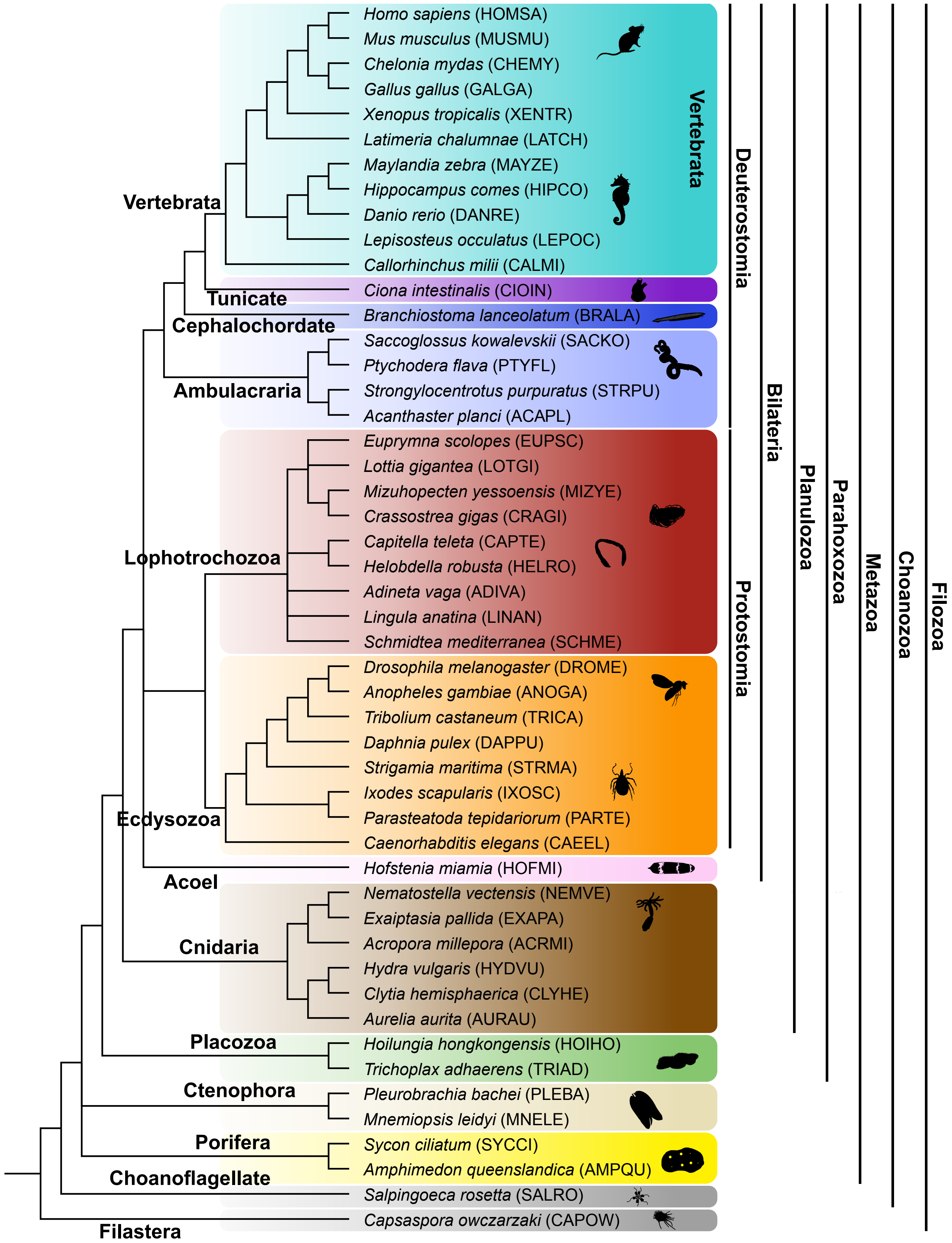

Supplementary Figure 1.

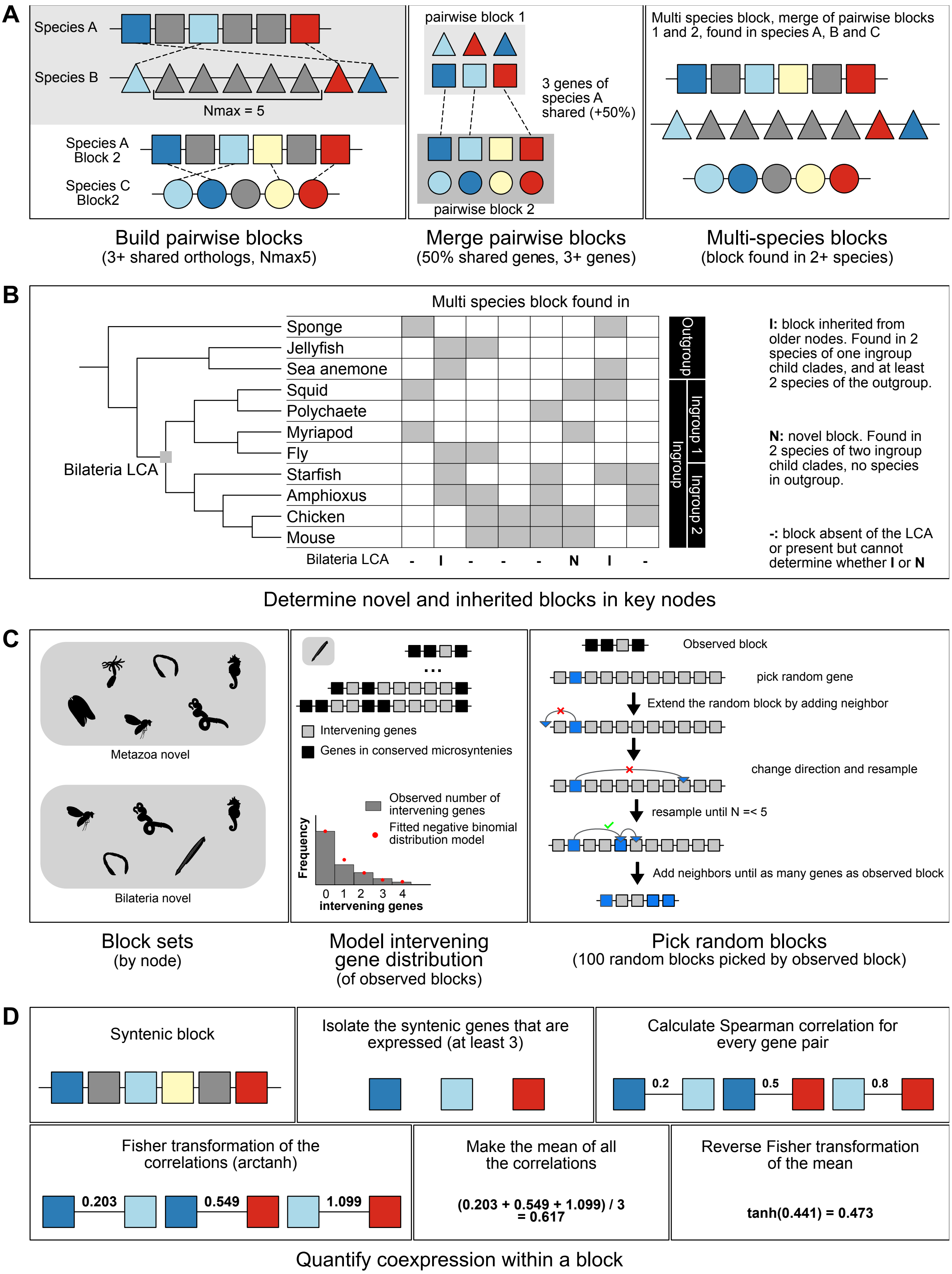

Supplementary Figure 2.

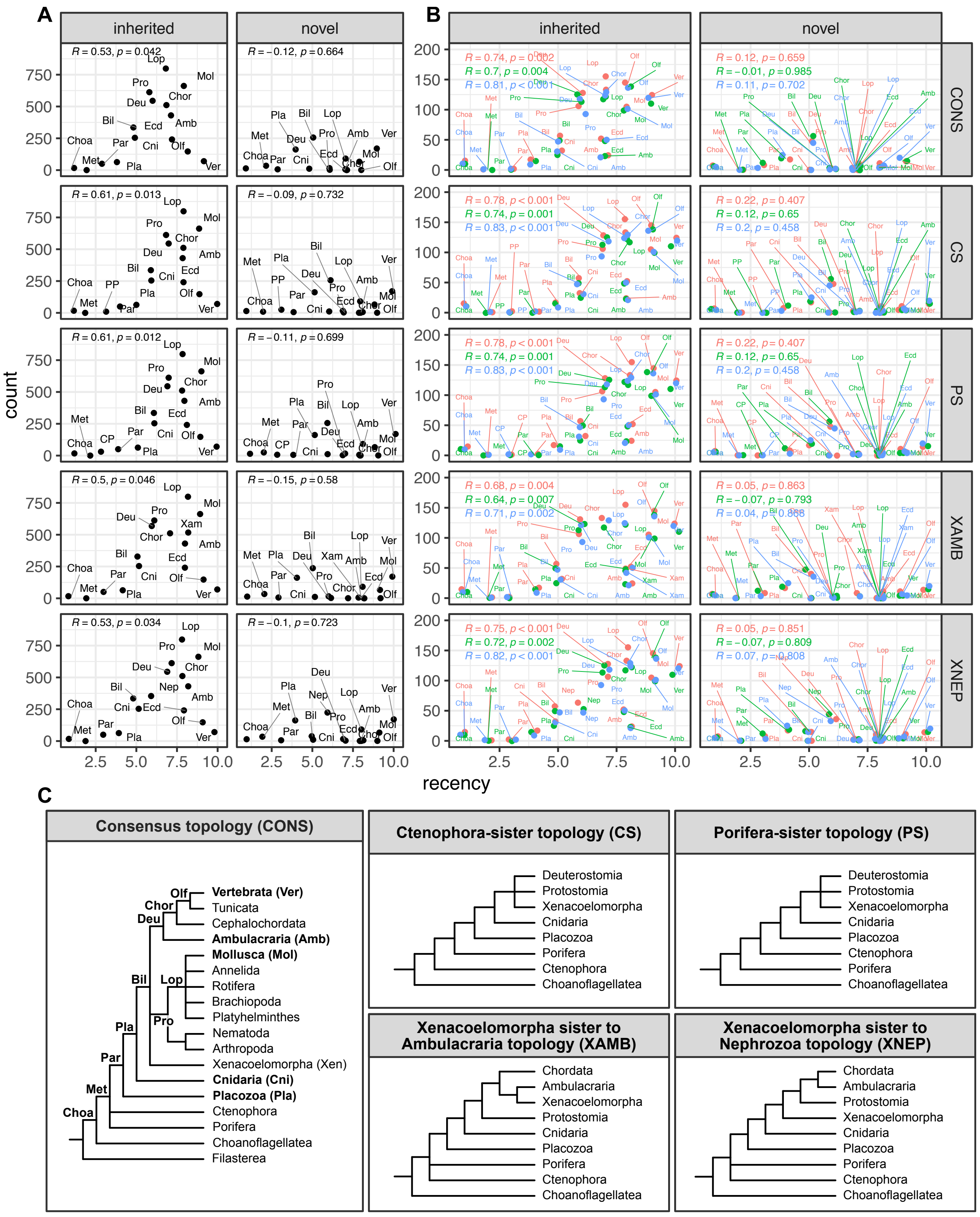

Supplementary Figure 3.

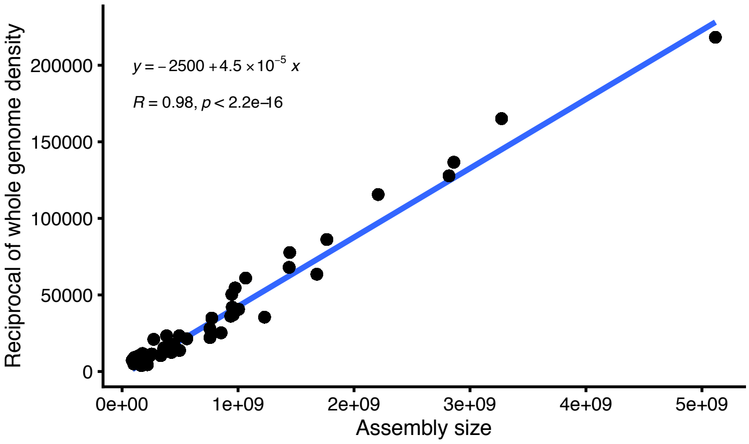

Supplementary Figure 4.

Supplementary Figure 5.

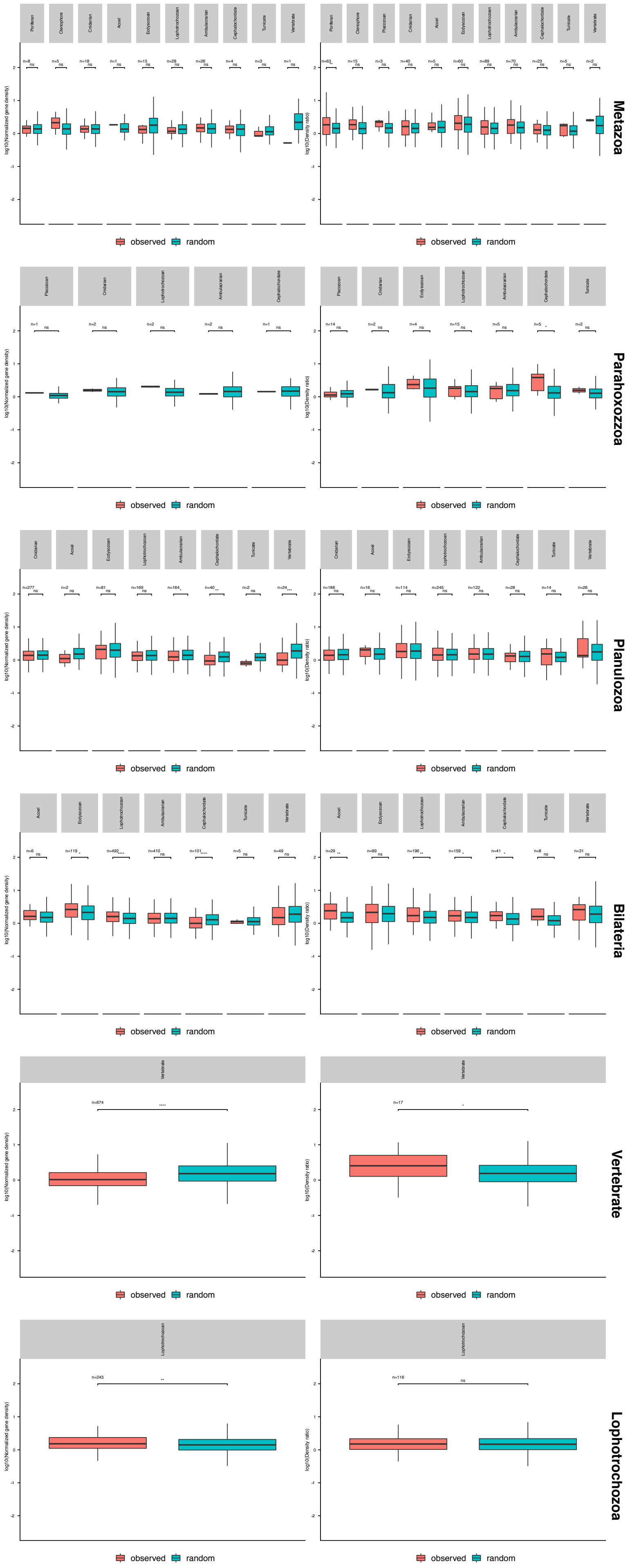

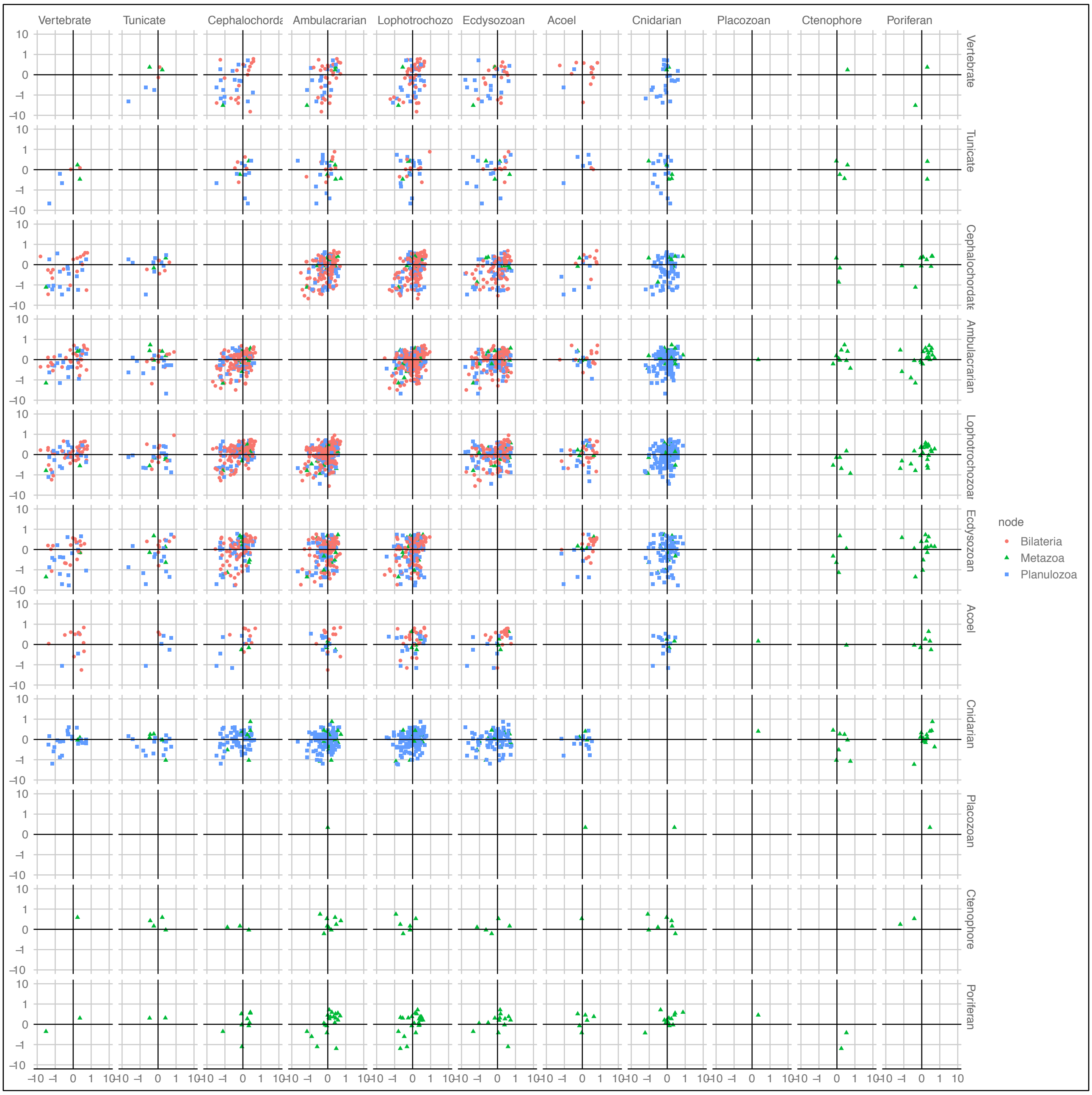

Supplementary Figure 6.

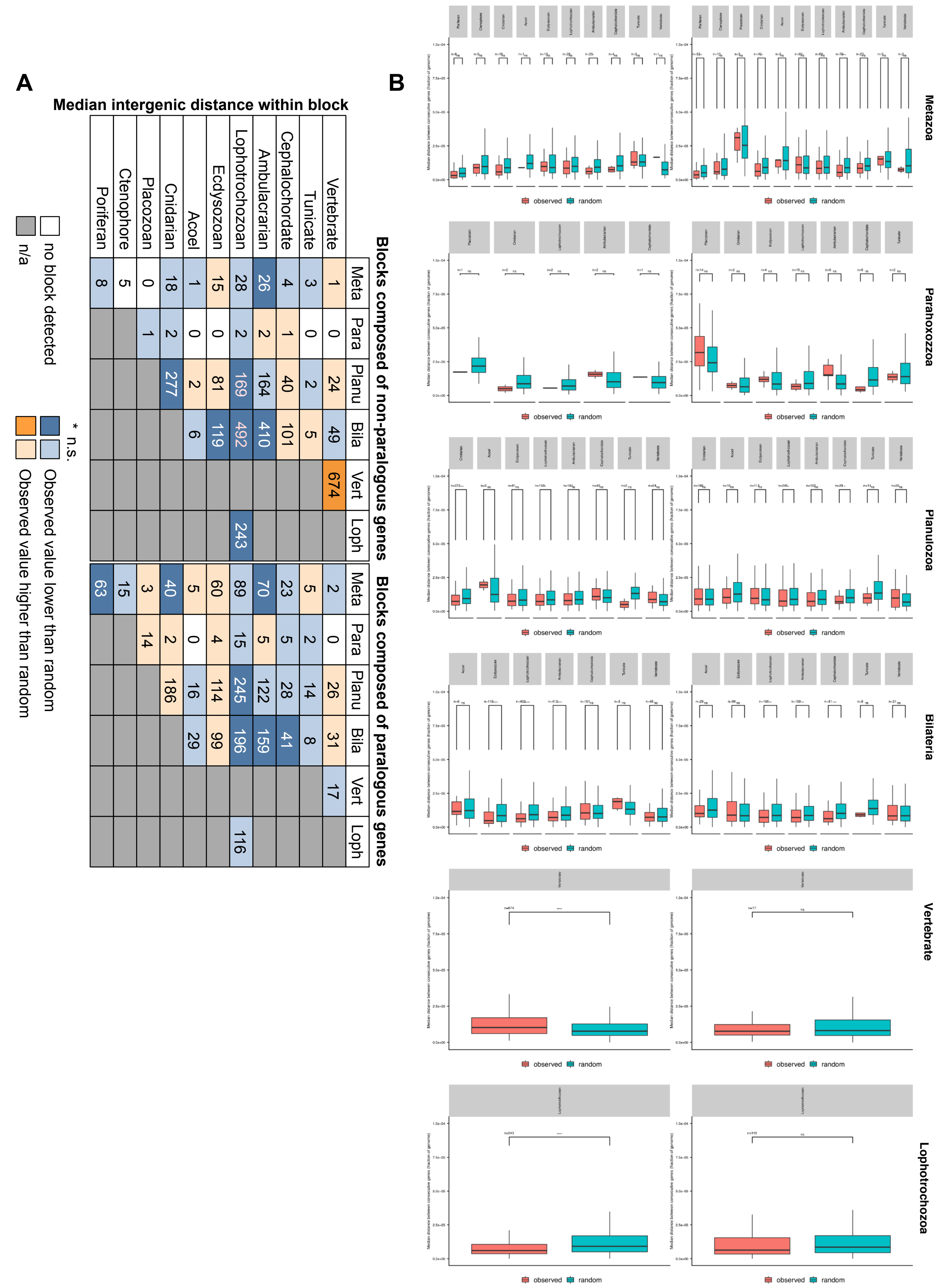

Supplementary Figure 7.

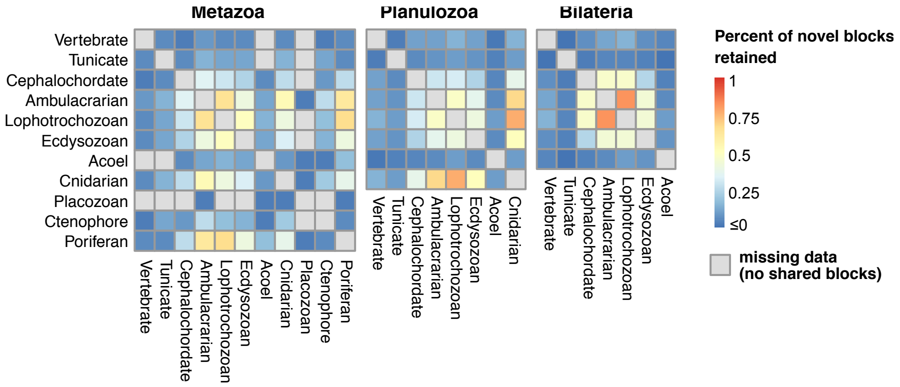

Supplementary Figure 8.

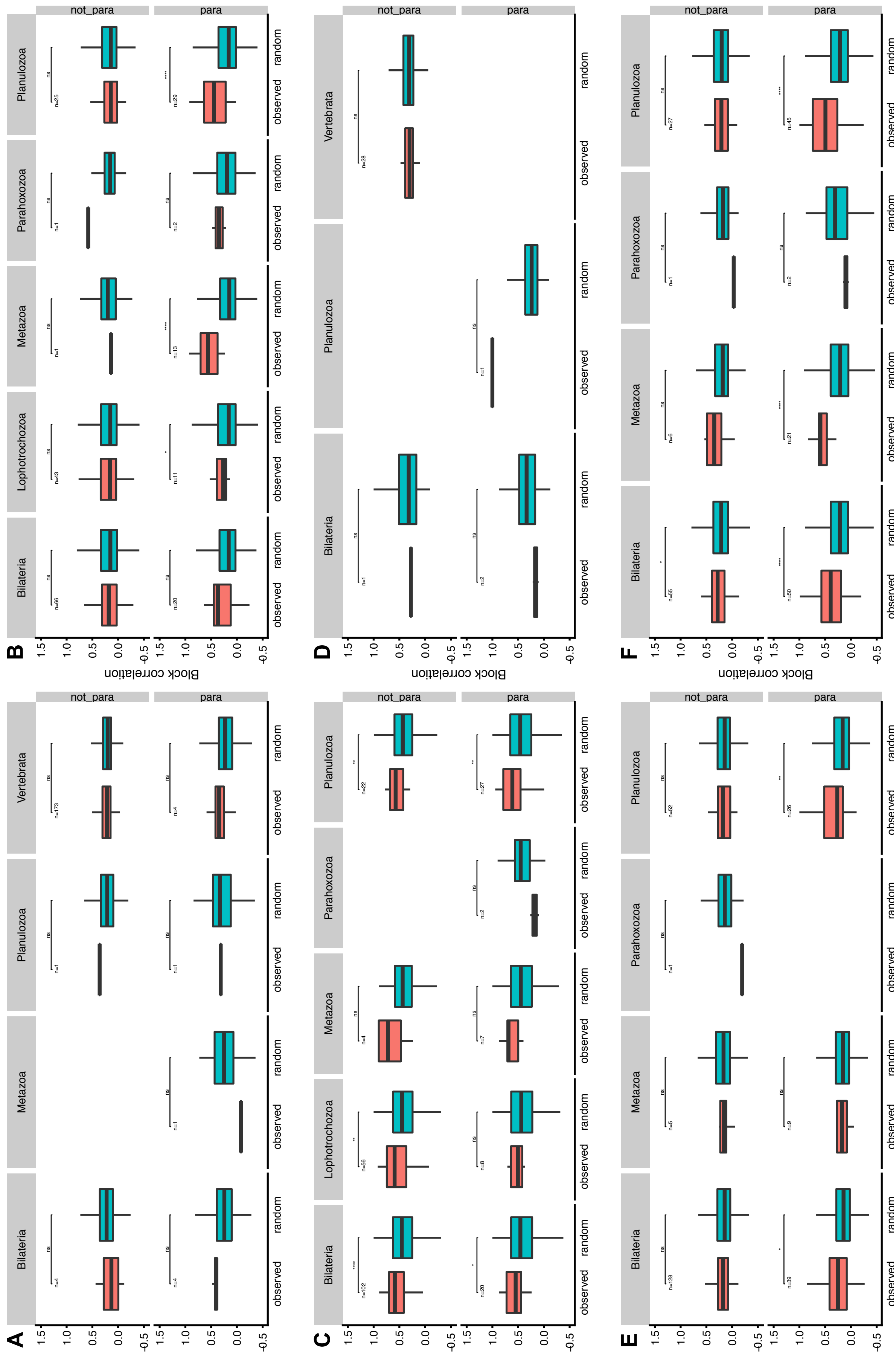

Supplementary Figure 9.

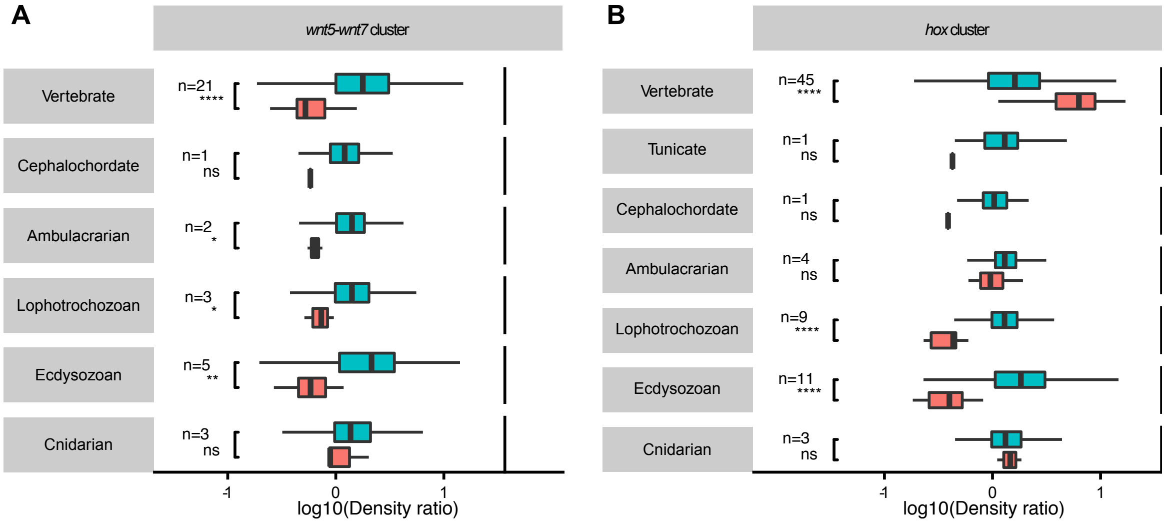

Supplementary Figure 10.

Supplementary Dataset 1. Code and scripts from the repository

Pdf of the repository comprising the code and scripts used this analysis at the time of submission.

Supplementary Dataset 2. GO enrichment tables.

GO enrichment for proteins encoded by genes located within BLCA (AA-BD), MLCA (BE-CN) and PLCA (CO-EA) novel blocks in *S. kowalevskii (*AA, CG, DV), *S. maritima* (AB, CI, DX), *S. purpuratus* (AC, CJ, DY), *I. scapularis* (AD, CA, DL), *A. planci* (AE, BI, CR), *A. vaga* (AF, BK, CT), *A. gambiae* (AG, BM, CU), *B. lanceolatum* (AH, BO, CW), *C.elegans* (AI), *C. milli* (AJ, CX), *C. teleta* (AK, BP, CY), *C. intestinalis* (AL, BQ, DA), *C. gigas* (AM, BS, DC), *D. rerio* (AN, DD), *D. pulex* (AO, BT, CQ), *D. melanogaster* (AP, BU, DE), *E. scolopes* (AQ, BV, DF), *H. robusta* (AR, BX, DI), *H. miamia* (AS, BY, DK), *H. sapiens* (AT, CO), *L. chalumnae* (AU, DM), *L. anatina* (AV, CB, DN), *L. gigantea* (AW, CC, DO), *M. zebra* (AX, DP), *M. yessoensis* (AY, CD, DQ), *M. musculus* (AZ, DR), *P. tepidariorum* (BA, BF, DT), *P. flava* (BB, BH, DU), *T. castaneum* (BC, CM, DZ), *X. tropicalis* (BD, CN, EA), *H. hongkongensis* (BE), *P. bachei* (BG), *A. millepora* (BJ, CS), *A. queenslandica* (BL), *A. aurita* (BN, CV), *C. hemisphaerica* (BR, DB), *E. pallida* (BW, DG), *H. vulgaris* (BZ, CP), *M. leidyi* (CE), *N. vectensis* (CF, DS), *S. mediterranea* (CH, DW), *S. ciliatum* (CK), *T. adhaerens* (CL), *C. midas* (CZ), *G. gallus* (DH) and *H. comes* (DK).

Supplementary Dataset 3. Syntenic density database

The list of the genes found in the novel syntenies of Metazoa, Parahoxozoa, Planulozoa, Bilateria, Vertebrata or Lophotrochozoa. Their respective block, multi species block, orthogroup, species, taxon, and functional annotation are provided.

Supplementary Table 1. Summary of GO terms enriched in MLCA blocks

List of the GO terms enriched in at least eight metazoans, distributed in at least two metazoan ingroups (i.e. Porifera, Ctenophora and Parahoxozoa).

Supplementary Table 2. Summary of GO terms enriched in PLCA blocks

List of the GO terms enriched in at least three cnidarians and eight bilaterian species

Supplementary Table 3. Summary of GO terms enriched in BLCA blocks

List of the GO terms enriched in at least four protostomes and four deuterostomes.
