# Supplementary material for: Emergence of distinct syntenic density regimes is associated with early metazoan genomic transitions
Source: BMC Genomics. 2022 Feb 17;23:143. doi: 10.1186/s12864-022-08304-2 (PMC8851819; doi:10.1186/s12864-022-08304-2)
Supplement: Supplementary file 2 — Additional file 2: Supplementary Dataset 1. Code and scripts from the repository. [file 12864_2022_8304_MOESM2_ESM.pdf]

list of species

See Methods from raw files download for the sources.

Species abreviation	Binomial name	Source
ACAPL	Acanthaster planci	NCBI GCF_001949145.1
ACRMI	Acropora millepora	NCBI GCF_004143615.1
ADIVA	Adineta vaga	ENSEMBL Release-45
AMPQU	Amphimedon queenslandica	ENSEMBL Release-45
ANOGA	Anopheles gambiae	ENSEMBL Release-45
AURAU	Aurelia aurita	David Gold Google Drive Aurelia.Genome_v1.2
BRALA	Branchiostoma lanceolatum	ENSEMBL Release-45
CAEEL	Caenorhabditis elegans	ENSEMBL Release-45
CALMI	Callorhinchus milii	NCBI GCF_000165045.1
CAPOW	Capitella teleta	ENSEMBL Release-45
CAPTE	Capsaspora owczarzaki	ENSEMBL Release-45
CHEMY	Chelonia mydas	NCBI GCF_000344595.1
CIOIN	Ciona intestinalis	NCBI GCF_000224145.3
CLYHE	Clytia hemisphaerica	MARIMBA
CRAGI	Crassostrea gigas	ENSEMBL Release-45
DANRE	Danio rerio	NCBI GCF_000002035.6
DAPPU	Daphnia pulex	ENSEMBL Release-45
DROME	Drosophila melanogaster	ENSEMBL Release-45
EUPSC	Euprymna scolopes	Lachesis assembly (Schmidbaur et al. in prep.)
EXAPA	Exaiptasia pallida	NCBI GCF_001417965.1
GALGA	Gallus gallus	NCBI GCF_000002315.6
HELRO	Helobdella robusta	ENSEMBL Release-45

Species abreviation	Binomial name	Source
HIPCO	Hippocampus comes	NCBI GCF_001891065.1
HOFMI	Hofstenia miamia	Downloaded from http://srivastavalab.rc.fas.harvard.edu also available in ENSEMBL Release-45
HOIHO	Hoilungia hongkongensis	https://bitbucket.org/molpalmuc/hoilungia-genome/src/master/tracks/
HOMSA	Homo sapiens	NCBI GCF_000001405.39
HYDVU	Hydra vulgaris	NHGRI hydra2.0
IXOSC	Ixodes scapularis	ENSEMBL Release-45
LATCH	Latimeria chalumnae	NCBI GCF_000225785.1
LEPOC	Lepisosteus oculatus	NCBI GCF_000242695.1
LINAN	Lingula anatina	ENSEMBL Release-45
LOTGI	Lottia gigantea	ENSEMBL Release-45
MAYZE	Maylandia zebra	NCBI GCF_000238955.4
MIZYE	Mizuhopecten yessoensis	Wang et al. 2017
MNELE	Mnemiopsis leidyi	NHGRI ML2.2
MUSMU	Mus musculus	NCBI GCF_000001635.26
NEMVE	Nematostella vectensis	ENSEMBL Release-45
PARTE	Parasteatoda tepidariorum	NCBI GCF_000365465.2
PLEBA	Pleurobrachia bachei	NCBI GCA_000695325.1, Neurobase
PTYFL	Ptychodera flava	OIST pfl_public_ver1.0
SACKO	Saccoglossus kowalevskii	OIST Sackov3
SALRO	Salpingoeca rosetta	ENSEMBL Release-45
SCHME	Schmidtea mediterranea	Planmine
STRMA	Strigamia maritima	ENSEMBL Release-45
STRPU	Strongylocentrotus purpuratus	ENSEMBL Release-45

Species abreviation	Binomial name	Source
SYCCI	Sycon ciliatum	DataDryad
TRIAD	Trichoplax adhaerens	ENSEMBL Release-45
TRICA	Tribolium castaneum	ENSEMBL Release-45
XENTR	Xenopus tropicalis	NCBI GCF_000004195.3

External scripts

orthoFinderToOrthogroup.pl: https://github.com/nijibabulu/metazoan_synteny/tree/master/scripts

1. Download of the raw data

1.1. Download peptide files

42/49 peptide databases directly downloaded using curl (or copied from proj, or cloned with git clone): ENSEMBL from release 45

```
#From NCBI: 16 genomes
curl
ftp://ftp.ncbi.nlm.nih.gov/genomes/all/GCF/000/001/405/GCF_000001405.39_GRCh38.p13
/GCF_000001405.39_GRCh38.p13_protein.faa.gz -o HOMSA_NCBI_raw_pep.fa.gz
curl
ftp://ftp.ncbi.nlm.nih.gov/genomes/all/GCF/000/001/635/GCF_000001635.26_GRCm38.p6/
GCF_000001635.26_GRCm38.p6_protein.faa.gz -o MUSMU_NCBI_raw_pep.fa.gz
curl
ftp://ftp.ncbi.nlm.nih.gov/genomes/all/GCF/000/002/035/GCF_000002035.6_GRCz11/GCF_
000002035.6_GRCz11_protein.faa.gz -o DANRE_NCBI_raw_pep.fa.gz
curl
ftp://ftp.ncbi.nlm.nih.gov/genomes/all/GCF/000/002/315/GCF_000002315.6_GRCg6a/GCF_
000002315.6_GRCg6a_protein.faa.gz -o GALGA_NCBI_raw_pep.fa.gz
curl
ftp://ftp.ncbi.nlm.nih.gov/genomes/all/GCF/000/004/195/GCF_000004195.3_Xenopus_tro
picalis_v9.1/GCF_000004195.3_Xenopus_tropicalis_v9.1_protein.faa.gz -o
XENTR_NCBI_raw_pep.fa.gz
curl
ftp://ftp.ncbi.nlm.nih.gov/genomes/all/GCF/000/165/045/GCF_000165045.1_Callorhinch
us_milii-6.1.3/GCF_000165045.1_Callorhinchus_milii-6.1.3_protein.faa.gz -o
CALMI_NCBI_raw_pep.fa.gz
curl
ftp://ftp.ncbi.nlm.nih.gov/genomes/all/GCF/004/143/615/GCF_004143615.1_amil_sf_1.1
/GCF_004143615.1_amil_sf_1.1_protein.faa.gz -o ACRMI_NCBI_raw_pep.fa.gz
curl
ftp://ftp.ncbi.nlm.nih.gov/genomes/all/GCF/000/224/145/GCF_000224145.3_KH/GCF_0002
24145.3_KH_protein.faa.gz -o CIOIN_NCBI_raw_pep.fa.gz
curl
ftp://ftp.ncbi.nlm.nih.gov/genomes/all/GCF/000/225/785/GCF_000225785.1_LatCha1/GCF
```

```
_000225785.1_LatCha1_protein.faa.gz -o LATCH_NCBI_raw_pep.fa.gz
curl
ftp://ftp.ncbi.nlm.nih.gov/genomes/all/GCF/000/238/955/GCF_000238955.4_M_zebra_UMD
2a/GCF_000238955.4_M_zebra_UMD2a_protein.faa.gz -o MAYZE_NCBI_raw_pep.fa.gz
curl
ftp://ftp.ncbi.nlm.nih.gov/genomes/all/GCF/000/242/695/GCF_000242695.1_LepOcu1/GCF
_000242695.1_LepOcu1_protein.faa.gz -o LEPOC_NCBI_raw_pep.fa.gz
curl
ftp://ftp.ncbi.nlm.nih.gov/genomes/all/GCF/000/344/595/GCF_000344595.1_CheMyd_1.0/
GCF_000344595.1_CheMyd_1.0_protein.faa.gz -o CHEMY_NCBI_raw_pep.fa.gz
curl
ftp://ftp.ncbi.nlm.nih.gov/genomes/all/GCF/000/365/465/GCF_000365465.2_Ptep_2.0/GC
F_000365465.2_Ptep_2.0_protein.faa.gz -o PARTE_NCBI_raw_pep.fa.gz
curl
ftp://ftp.ncbi.nlm.nih.gov/genomes/all/GCF/001/417/965/GCF_001417965.1_Aiptasia_ge
nome_1.1/GCF_001417965.1_Aiptasia_genome_1.1_protein.faa.gz -o
EXAPA_NCBI_raw_pep.fa.gz
curl
ftp://ftp.ncbi.nlm.nih.gov/genomes/all/GCF/001/891/065/GCF_001891065.1_H_comes_QL1
_v1/GCF_001891065.1_H_comes_QL1_v1_protein.faa.gz -o HIPCO_NCBI_raw_pep.fa.gz
curl ftp://ftp.ncbi.nlm.nih.gov/genomes/all/GCF/001/949/145/GCF_001949145.1_OKI-
Apl_1.0/GCF_001949145.1_OKI-Apl_1.0_protein.faa.gz -o ACAPL_NCBI_raw_pep.fa.gz

#From ENSEMBL: 20 genomes
curl ftp://ftp.ensemblgenomes.org/pub/metazoa/release-
45/fasta/adineta_vaga/pep/Adineta_vaga.AMS_PRJEB1171_v1.pep.all.fa.gz -o
ADIVA_ENSEMBL_raw_pep.fa.gz
curl ftp://ftp.ensemblgenomes.org/pub/metazoa/release-
45/fasta/amphimedon_queenslandica/pep/Amphimedon_queenslandica.Aqu1.pep.all.fa.gz
-o AMPQU_ENSEMBL_raw_pep.fa.gz
curl ftp://ftp.ensemblgenomes.org/pub/metazoa/release-
45/fasta/anopheles_gambiae/pep/Anopheles_gambiae.AgamP4.pep.all.fa.gz -o
ANOGA_ENSEMBL_raw_pep.fa.gz
curl ftp://ftp.ensemblgenomes.org/pub/release-
45/metazoa/fasta/branchiostoma_lanceolatum/pep/Branchiostoma_lanceolatum.BraLan2.p
ep.all.fa.gz -o BRALA_ENSEMBL_raw_pep.fa.gz
curl ftp://ftp.ensemblgenomes.org/pub/metazoa/release-
45/fasta/caenorhabditis_elegans/pep/Caenorhabditis_elegans.WBcel235.pep.all.fa.gz
-o CAEEL_ENSEMBL_raw_pep.fa.gz
curl ftp://ftp.ensemblgenomes.org/pub/metazoa/release-
45/fasta/capitella_teleta/pep/Capitella_teleta.Capitella_teleta_v1.0.pep.all.fa.gz
-o CAPTE_ENSEMBL_raw_pep.fa.gz
curl ftp://ftp.ensemblgenomes.org/pub/metazoa/release-
45/fasta/crassostrea_gigas/pep/Crassostrea_gigas.oyster_v9.pep.all.fa.gz -o
CRAGI_ENSEMBL_raw_pep.fa.gz
curl ftp://ftp.ensemblgenomes.org/pub/metazoa/release-
45/fasta/daphnia_pulex/pep/Daphnia_pulex.V1.0.pep.all.fa.gz -o
DAPPU_ENSEMBL_raw_pep.fa.gz
curl ftp://ftp.ensemblgenomes.org/pub/metazoa/release-
45/fasta/drosophila_melanogaster/pep/Drosophila_melanogaster.BDGP6.22.pep.all.fa.g
z -o DROME_ENSEMBL_raw_pep.fa.gz
curl ftp://ftp.ensemblgenomes.org/pub/metazoa/release-
45/fasta/helobdella_robusta/pep/Helobdella_robusta.Helro1.pep.all.fa.gz -o
HELRO_ENSEMBL_raw_pep.fa.gz
```

```
curl ftp://ftp.ensemblgenomes.org/pub/metazoa/release-45/fasta/ixodes_scapularis/pep/Ixodes_scapularis.IscaW1.pep.all.fa.gz -o IXOSC_ENSEMBL_raw_pep.fa.gz
curl ftp://ftp.ensemblgenomes.org/pub/metazoa/release-45/fasta/lingula_anatina/pep/Lingula_anatina.LinAna1.0.pep.all.fa.gz -o LINAN_ENSEMBL_raw_pep.fa.gz
curl ftp://ftp.ensemblgenomes.org/pub/metazoa/release-45/fasta/lottia_gigantea/pep/Lottia_gigantea.Lotgi1.pep.all.fa.gz -o LOTGI_ENSEMBL_raw_pep.fa.gz
curl ftp://ftp.ensemblgenomes.org/pub/metazoa/release-45/fasta/nematostella_vectensis/pep/Nematostella_vectensis.ASM20922v1.pep.all.fa.gz -o NEMVE_ENSEMBL_raw_pep.fa.gz
curl ftp://ftp.ensemblgenomes.org/pub/metazoa/release-45/fasta/strigamia_maritima/pep/Strigamia_maritima.Smar1.pep.all.fa.gz -o STRMA_ENSEMBL_raw_pep.fa.gz
curl ftp://ftp.ensemblgenomes.org/pub/metazoa/release-45/fasta/strongylocentrotus_purpuratus/pep/Strongylocentrotus_purpuratus.Spur_3.1.pep.all.fa.gz -o STRPU_ENSEMBL_raw_pep.fa.gz
curl ftp://ftp.ensemblgenomes.org/pub/metazoa/release-45/fasta/tribolium_castaneum/pep/Tribolium_castaneum.Tcas5.2.pep.all.fa.gz -o TRICA_ENSEMBL_raw_pep.fa.gz
curl ftp://ftp.ensemblgenomes.org/pub/metazoa/release-45/fasta/trichoplax_adhaerens/pep/Trichoplax_adhaerens.ASM15027v1.pep.all.fa.gz -o TRIAD_ENSEMBL_raw_pep.fa.gz
curl ftp://ftp.ensemblgenomes.org/pub/protists/release-45/fasta/protists_choanoflagellida1_collection/salpingoeca_rosetta_gca_000188695/pep/Salpingoeca_rosetta_gca_000188695.Proterospongia_sp_ATCC50818.pep.all.fa.gz -o SALRO_ENSEMBL_raw_pep.fa.gz
curl ftp://ftp.ensemblgenomes.org/pub/protists/release-45/fasta/protists_ichthyosporea1_collection/capsaspora_owczarzaki_atcc_30864_gca_000151315/pep/Capsaspora_owczarzaki_atcc_30864_gca_000151315.C_owczarzaki_V2.pep.all.fa.gz -o CAPOW_ENSEMBL_raw_pep.fa.gz

#From other sources: 7 genomes
curl http://marimba.obs-vlfr.fr/download/file/fid/50 -o CLYHE_OTHER_raw_pep.fa
curl https://research.nhgri.nih.gov/hydra/download/genemodels_proteins/hydra2.0_genemodels.aa.gz -o HYDVU_OTHER_raw_pep.fa.gz
git clone https://bitbucket.org/molpalmuc/hoilungia-genome/src/master/sequences/Hhon_BRAKER1_proteins.fasta.gz HOIHO_raw_pep.fa.gz
cp /proj/Simakov/OTHER/SCALLOP/PYgenome-pep.fa MIZYE_OTHER_raw_pep.fa
curl https://research.nhgri.nih.gov/mnemiopsis/download/proteome/ML2.2.aa.gz -o MNELE_OTHER_raw_pep.fa.gz
curl https://marinegenomics.oist.jp/acornworm/download/pfl_public_ver1.0.prot > PTYFL_OTHER_raw_pep.fa
```

The 7 remaining genomes were downloaded with a browser (and or built using annotation files with gffread):

- Aurelia aurita from: https://drive.google.com/drive/folders/1NC6bZ9cxWkZyofOsMPzrxIH3C7m1ySiu, Aurelia.Genome_v1.2_Protein_Models_12-28-18.fasta, renamed AURAU_raw_pep.fa
- Euprymna scolopes clusters peptide file was copied from: /proj/Simakov/EUPRYMNA/MAHDITRAN/Euprymna_scolopes.fa , named EUPSC_raw_pep.fa

- Hofstenia miamia gff3 file was downloaded from http://srivastavalab.rc.fas.harvard.edu gffread was used to build HOFMI_raw_pep.fa using hmi_gene_annotation.gff3 and hmi_genome.fa
- Saccoglossus kowalevskii: SkowalevskiiJGIv3.0.longestTrs.pep.fa.gz Downloaded from Metazome v3 (https://metazome.jgi.doe.gov/pz/portal.html#!bulk?org=Org_Skowalevskii_er), called SACKO_OTHER_raw_pep.fa.gz
- Schmidtea mediterranea: gffread used to build SCHME_OTHER_ra_pep.fa using gff http://planmine.mpi-cbg.de/planmine/model/bulkdata/smes_v2_hconf_SMESG.gff3.zip (high confidence transcripts) and http://planmine.mpi-cbg.de/planmine/model/bulkdata/dd_Smes_g4.fasta.zip

```
gffread SCHME_OTHER.gff3 -g dd_Smes_g4.fasta -y SCHME_OTHER_raw_pep.fa
```

- Sycon ciliatum genome, CDS and peptide downloaded from datadryad (https://datadryad.org/resource/doi:10.5061/dryad.tn0f3).
- Pleurobrachia bachei filtered gene models mRNAs (CDS) were downloaded from https://neurobase.rc.ufl.edu/pleurobrachia/download (03_P-bachei_Filtered_Gene_Models_RNA.txt) and also translated into peptides (also using transeq, frame 1), file was named PLEBA_raw_pep.fa we use `sed -i 's/_1$//' PLEBA.fa` to delete the frame added by transseq to the accession

Finally, we gunzip all the gz files

```
gunzip *.fa.gz
```

1.2. Download annotation files

1.2.1 Download of available gff files

gff files were available for 47 out of the 49 species. For SYCCI and PLEBA, we'll map the transcripts (see 1.2.2) 39/49 downloaded using curl: We want the gffs which show all the sequences. For example, in the case of a chromosomal level assembly, we want the unplaced scaffolds as well.

```
#16 Gffs downloaded from NCBI
curl ftp://ftp.ncbi.nlm.nih.gov/genomes/all/GCF/001/949/145/GCF_001949145.1_OKI-Apl_1.0/GCF_001949145.1_OKI-Apl_1.0_genomic.gff.gz -o ACAPL_NCBI.gff3.gz
curl
ftp://ftp.ncbi.nlm.nih.gov/genomes/all/GCF/004/143/615/GCF_004143615.1_amil_sf_1.1/GCF_004143615.1_amil_sf_1.1_genomic.gff.gz -o ACRMI_NCBI.gff.gz
curl
ftp://ftp.ncbi.nlm.nih.gov/genomes/all/GCF/000/165/045/GCF_000165045.1_Callorhinchus_milii-6.1.3/GCF_000165045.1_Callorhinchus_milii-6.1.3_genomic.gff.gz -o CALMI_NCBI.gff3.gz
curl
ftp://ftp.ncbi.nlm.nih.gov/genomes/all/GCF/000/224/145/GCF_000224145.3_KH/GCF_000224145.3_KH_genomic.gff.gz -o CIOIN_NCBI.gff3.gz
curl
ftp://ftp.ncbi.nlm.nih.gov/genomes/all/GCF/000/002/035/GCF_000002035.6_GRCz11/GCF_000002035.6_GRCz11_genomic.gff.gz -o DANRE_NCBI.gff3.gz
```

```
curl
ftp://ftp.ncbi.nlm.nih.gov/genomes/all/GCF/001/417/965/GCF_001417965.1_Aiptasia_genome_1.1/GCF_001417965.1_Aiptasia_genome_1.1_genomic.gff.gz -o EXAPA_NCBI.gff.gz
curl
ftp://ftp.ncbi.nlm.nih.gov/genomes/all/GCF/000/002/315/GCF_000002315.6_GRCg6a/GCF_000002315.6_GRCg6a_genomic.gff.gz -o GALGA_NCBI.gff3.gz
curl
ftp://ftp.ncbi.nlm.nih.gov/genomes/all/GCF/000/242/695/GCF_000242695.1_LepOcu1/GCF_000242695.1_LepOcu1_genomic.gff.gz -o LEPOC_NCBI.gff3.gz
curl
ftp://ftp.ncbi.nlm.nih.gov/genomes/all/GCF/000/001/635/GCF_000001635.26_GRCm38.p6/GCF_000001635.26_GRCm38.p6_genomic.gff.gz -o MUSMU_NCBI.gff3.gz
curl
ftp://ftp.ncbi.nlm.nih.gov/genomes/all/GCF/000/001/405/GCF_000001405.39_GRCh38.p13/GCF_000001405.39_GRCh38.p13_genomic.gff.gz -o HOMSA_NCBI.gff.gz
curl
ftp://ftp.ncbi.nlm.nih.gov/genomes/all/GCF/000/365/465/GCF_000365465.2_Ptep_2.0/GCF_000365465.2_Ptep_2.0_genomic.gff.gz -o PARTE_NCBI.gff3.gz
curl
ftp://ftp.ncbi.nlm.nih.gov/genomes/all/GCF/000/225/785/GCF_000225785.1_LatCha1/GCF_000225785.1_LatCha1_genomic.gff.gz -o LATCH_NCBI.gff.gz
curl
ftp://ftp.ncbi.nlm.nih.gov/genomes/all/GCF/001/891/065/GCF_001891065.1_H_comes_QL1_v1/GCF_001891065.1_H_comes_QL1_v1_genomic.gff.gz -o HIPCO_NCBI.gff.gz
curl
ftp://ftp.ncbi.nlm.nih.gov/genomes/all/GCF/000/238/955/GCF_000238955.4_M_zebra_UMD2a/GCF_000238955.4_M_zebra_UMD2a_genomic.gff.gz -o MAYZE_NCBI.gff.gz
curl
ftp://ftp.ncbi.nlm.nih.gov/genomes/all/GCF/000/344/595/GCF_000344595.1_CheMyd_1.0/GCF_000344595.1_CheMyd_1.0_genomic.gff.gz -o CHEMY_NCBI.gff.gz
curl
ftp://ftp.ncbi.nlm.nih.gov/genomes/all/GCF/000/004/195/GCF_000004195.3_Xenopus_tropicalis_v9.1/GCF_000004195.3_Xenopus_tropicalis_v9.1_genomic.gff.gz -o
XENTR_NCBI.gff.gz

#20 Gffs downloaded from ENSEMBL
curl ftp://ftp.ensemblgenomes.org/pub/metazoa/release-45/gff3/adineta_vaga/Adineta_vaga.AMS_PRJEB1171_v1.45.gff3.gz -o
ADIVA_ENSEMBL.gff3.gz
curl ftp://ftp.ensemblgenomes.org/pub/metazoa/release-45/gff3/anopheles_gambiae//Anopheles_gambiae.AgamP4.45.gff3.gz -o
ANOGA_ENSEMBL.gff3.gz
curl ftp://ftp.ensemblgenomes.org/pub/metazoa/release-45/gff3/caenorhabditis_elegans//Caenorhabditis_elegans.WBcel235.45.gff3.gz -o
CAEEL_ENSEMBL.gff3.gz
curl ftp://ftp.ensemblgenomes.org/pub/metazoa/release-45/gff3/capitella_teleta//Capitella_teleta.Capitella_teleta_v1.0.45.gff3.gz -o
CAPTE_ENSEMBL.gff3.gz
curl ftp://ftp.ensemblgenomes.org/pub/metazoa/release-45/gff3/crassostrea_gigas/Crassostrea_gigas.oyster_v9.45.gff3.gz -o
CRAGI_ENSEMBL.gff3.gz
curl ftp://ftp.ensemblgenomes.org/pub/metazoa/release-45/gff3/daphnia_pulex/Daphnia_pulex.V1.0.45.gff3.gz -o DAPPU_ENSEMBL.gff3.gz
curl ftp://ftp.ensemblgenomes.org/pub/metazoa/release-
```

```
45/gff3/drosophila_melanogaster/Drosophila_melanogaster.BDGP6.22.45.gff3.gz -o
DROME_ENSEMBL.gff3.gz
curl ftp://ftp.ensemblgenomes.org/pub/metazoa/release-
45/gff3/helobdella_robusta/Helobdella_robusta.Helro1.45.gff3.gz -o
HELRO_ENSEMBL.gff3.gz
curl ftp://ftp.ensemblgenomes.org/pub/metazoa/release-
45/gff3/ixodes_scapularis/Ixodes_scapularis.IscaW1.45.gff3.gz -o
IXOSC_ENSEMBL.gff3.gz
curl ftp://ftp.ensemblgenomes.org/pub/metazoa/release-
45/gff3/lingula_anatina/Lingula_anatina.LinAna1.0.45.gff3.gz -o
LINAN_ENSEMBL.gff3.gz
curl ftp://ftp.ensemblgenomes.org/pub/metazoa/release-
45/gff3/lottia_gigantea/Lottia_gigantea.Lotgi1.45.gff3.gz -o LOTGI_ENSEMBL.gff3.gz
curl ftp://ftp.ensemblgenomes.org/pub/metazoa/release-
45/gff3/nematostella_vectensis//Nematostella_vectensis.ASM20922v1.45.gff3.gz -o
NEMVE_ENSEMBL.gff3.gz
curl ftp://ftp.ensemblgenomes.org/pub/metazoa/release-
45/gff3/strigamia_maritima//Strigamia_maritima.Smar1.45.gff3.gz -o
STRMA_ENSEMBL.gff3.gz
curl ftp://ftp.ensemblgenomes.org/pub/metazoa/release-
45/gff3/strongylocentrotus_purpuratus//Strongylocentrotus_purpuratus.Spur_3.1.45.g
ff3.gz -o STRPU_ENSEMBL.gff3.gz
curl ftp://ftp.ensemblgenomes.org/pub/metazoa/release-
45/gff3/tribolium_castaneum/Tribolium_castaneum.Tcas5.2.45.gff3.gz -o
TRICA_ENSEMBL.gff3.gz
curl ftp://ftp.ensemblgenomes.org/pub/metazoa/release-
45/gff3/trichoplax_adhaerens//Trichoplax_adhaerens.ASM15027v1.45.gff3.gz -o
TRIAD_ENSEMBL.gff3.gz
curl ftp://ftp.ensemblgenomes.org/pub/protists/release-
45/gff3/protists_choanoflagellida1_collection/salpingoeca_rosetta_gca_000188695/Sa
lpingoeca_rosetta_gca_000188695.Proterospongia_sp_ATCC50818.45.gff3.gz -o
SALRO_ENSEMBL.gff3.gz
curl ftp://ftp.ensemblgenomes.org/pub/protists/release-
45/gff3/protists_ichthyosporea1_collection/capsaspora_owczarzaki_atcc_30864_gca_00
0151315/Capsaspora_owczarzaki_atcc_30864_gca_000151315.C_owczarzaki_V2.45.gff3.gz
-o CAPOW_ENSEMBL.gff3.gz
curl ftp://ftp.ensemblgenomes.org/pub/release-
45/metazoa/gff3/amphimedon_queenslandica/Amphimedon_queenslandica.Aqu1.45.gff3.gz
-o AMPQU_ENSEMBL.gff3.gz
curl ftp://ftp.ensemblgenomes.org/pub/release-
45/metazoa/gff3/branchiostoma_lanceolatum/Branchiostoma_lanceolatum.BraLan2.45.gff
3.gz -o BRALA_ENSEMBL.gff3.gz

#4 gffs downloaded from other ressources
curl
https://research.nhgri.nih.gov/hydra/download/genemodels_gff3/hydra2.0_genemodels.
gff3.gz -o HYDVU_OTHER.gff3.gz
curl curl https://bitbucket.org/molpalmuc/hoilungia-
genome/raw/0d523a5b8556741a37918f3f30d0ed0414833912/tracks/Hhon_BRAKER1_CDS.gff3.g
z -o HOIHO_OTHER.gff3.gz
curl https://marinegenomics.oist.jp/acornworm/download/pfl_public_ver1.0.gff3 >
PTYFL_OTHER.gff3
curl https://research.nhgri.nih.gov/mnemiopsis/download/proteome/ML2.2.gff3.gz -o
MNELE_OTHER.gff3.gz
```

- *Aurelia aurita* (https://drive.google.com/drive/folders/1NC6bZ9cxWkZyofOsMPzrxIH3C7m1ySiu), named AURAU_OTHER.gff3
- *Branchiostoma lanceolatum*, gtf downloaded from https://www.dropbox.com/s/d4fqnoa8gdix3pa/Bla_annot_final.gtf.gz named BRALA_OTHER.gtf.gz
- *Clytia hemisphaerica*, gff from http://marimba.obs-vlfr.fr/download/file/fid/51 called CLYHE_OTHER.gff3
- *Euprymna scolopes*: gff located in /proj/Simakov/EUPRYMNA/MAHDITRAN/clusters_esc.gff3 use gffparser in the next parts firectly on this file.
- *Hofstenia miamia*, gff3 downloaded from http://srivastavalab.rc.fas.harvard.edu, hmi_gene_annotation.gff3 was renamed HOFMI_OTHER.gff3
- *Hoilungia hongkongensis*, gff3 downloaded from https://bitbucket.org/molpalmuc/hoilungia-genome/src/master/tracks/Hhon_BRAKER1_CDS.gff3.gz named HOIHO_OTHER.gff3
- *Mizuhopecten yessoensis* gff located in /proj/Simakov/OTHER/SCALLOP/chr.id.gff3 use gffparser in the next parts directly on this file.
- *Schmidtea mediterranea*: donwloaded high confidence gene predictions from http://planmine.mpi-cbg.de/planmine/aspect.do?name=Gene%20Predictions, called the file SCHME_OTHER.gff3
- *Saccoglossus kowalevskii*: downloaded the longest transcrfipts form Metazome v3 SkowalevskiiJGIv3.0.longestTrs.gff3.gz

1.2.2 Mapping of transcripts/gene models onto the genome when annotation files were not uploaded by the authors (Pleurobrachia)

The annotations for *Pleurobrachia bachei* gene models and *Sycon ciliatum* transcripts were not available. Genome database build using **gmap_gbuild**, transcripts mapped onto the genome using **gmap**

```
module load gmap
gmap_build -D Pleurobrachia_bachei -d PLEBA_genome
GCA_000695325.1_P.bachei_draft_genome_v.1.1_genomic.fna
gmap -D Pleurobrachia_bachei/ -d PLEBA_genome 03_P-
bachei_Filtered_Gene_Models_RNA.txt --gff3-add-separators=1 -f gff3_gene >
PLEBA_03_mapped.gff3

gmap_build -D Sycon_ciliatum/ -d SYCCI_genome sycon.genome.fa
gmap -D Sycon_ciliatum/ -d SYCCI_genome sycon.cds.fa --gff3-add-separators=1 -f
gff3_gene > SYCCI_mapped.gff3
```

- *Pleurobrachia bachei* : **18871 out of 18950** transcripts are mapped (i.e. 99.58%)
- *Sycon ciliatum* : **49278 out of 50731** transcripts are mapped (i.e. 97.00%)

We'll keep all the peptides in the following analyses (and not only the ones mapped onto the genome), because we want to differentiate between loss of genes and loss of synteny. Keep the best mapped transcript

```
grep mrna1 SYCCI_mapped.gff3 | grep -P '\texon\t' > SYCCI_mapped_final.gff3
sed -i 's/sctid/scpid/g' SYCCI_mapped_final.gff3
grep mrna1 PLEBA_03_mapped.gff3 | grep -P '\texon\t' > PLEBA_mapped_final.gff3
```

2. Preparation of data for susbequent analyses

There is some preliminary steps we want to do before running the gff parser.

- For *H .miamia*, we modify the gff like this:

`sed -i '/\|/s/=.*\|/=/' HOFMI_hmi_gene_annotation.gff3`

- In the case of *S. ciliatum* and *P. bachei*, gmap outputs multiple paths per mRNA. We want to keep only one. Also, gmap maps both CDS and exons. Since we're mapping only CDS (some don't start with ATG), we'll keep only exons features.

```
grep mrna1 SYCCI_mapped.gff3 | grep -P '\texon\t' > SYCCI_mapped_final.gff3
sed -i 's/sctid/scpid/g' SYCCI_mapped_final.gff3
grep mrna1 PLEBA_03_mapped.gff3 | grep -P '\texon\t' > PLEBA_mapped_final.gff3
```

In the case of *S. kowalevskii* the accessions in the fasta files are gene names, and are not in the CDS lines. So we'll edit the gff with this script. It'll add up ";protein_id=Sakowv12345678m" comment in the last field of the gff on the lines containing CDS features. This is to make the file parseable by pyMakeMap

```
sk_gff_preparation.py SACKO_SkowalevskiiJGIv3.0.longestTrs.gff3 >
SACKO_prepped.gff3
rm SACKO_SkowalevskiiJGIv3.0.longestTrs.gff3
```

2.1 Prepare non-redundant chrom and fasta files from gff

CHROM files are the format we commonly use. They're made by parsing gff annotation files. Some gffs comprise info about exon, gene, CDS, UTR.

We define gene coordinates as the CDS information for every species.

2.1.1 Prepare chrom files for NCBI data

First, we'll delete the mitochondrial scaffolds from the gffs. Not all genome assemblies posess mitochondrial, we don't want mitochondrial cluster as falsely assigned synteny novelties.

```
grep "NC_007788.1" CALMI_NCBI.gff3| cut -f1 | grep -wvf - ACAPL_NCBI.gff3 >
ACAPL_NCBI_filt.gff3; rm ACAPL_NCBI.gff3
#ACRMI has no MT chrom
grep "NC_014285.1" CALMI_NCBI.gff3| cut -f1 | grep -wvf - CALMI_NCBI.gff3 >
CALMI_NCBI_filt.gff; rm CALMI_NCBI.gff3
grep "NC_017929.1" CIOIN_NCBI.gff3| cut -f1 | grep -wvf - CIOIN_NCBI.gff3 >
CIOIN_NCBI_filt.gff3; rm CIOIN_NCBI.gff3
grep "NC_002333.2" DANRE_NCBI.gff3| cut -f1 | grep -wvf - DANRE_NCBI.gff3 >
DANRE_filt_NCBI.gff3; rm DANRE_NCBI.gff3
#EXAPA has no MT chrom
grep -v "NC_040902.1" GALGA_NCBI.gff3 > GALGA_filt_NCBI.gff3; rm GALGA_NCBI.gff3
```

```
grep "NC_004744.1" LEPOC_NCBI.gff3| cut -f1 | grep -wvf - LEPOC_NCBI.gff3 >
LEPOC_filt_NCBI.gff3; rm LEPOC_NCBI.gff3
grep "NC_005089.1" MUSMU_NCBI.gff3| cut -f1 | grep -wvf - MUSMU_NCBI.gff3 >
MUSMU_filt_NCBI.gff3; rm MUSMU_NCBI.gff3
grep "NC_012920.1" HOMSA_NCBI.gff| cut -f1|   grep -wvf - HOMSA_NCBI.gff  >
HOMSA_filt_NCBI.gff; rm HOMSA_NCBI.gff
#PARTE has no MT
grep "NC_001804.1" LATCH_NCBI.gff|cut -f1|grep -wvf - LATCH_NCBI.gff >
LATCH_filt_NCBI.gff; rm LATCH_NCBI.gff
grep "NC_020336.1" HIPCO_NCBI.gff|cut -f1|grep -wvf - HIPCO_NCBI.gff >
HIPCO_filt_NCBI.gff; rm HIPCO_NCBI.gff
grep "NC_027944.1" MAYZE_NCBI.gff|cut -f1|grep -wvf - MAYZE_NCBI.gff >
MAYZE_filt_NCBI.gff; rm MAYZE_NCBI.gff
grep "NC_000886.1" CHEMY_NCBI.gff|cut -f1|grep -wvf - CHEMY_NCBI.gff >
CHEMY_NCBI_filt.gff; rm CHEMY_NCBI.gff
grep "NC_006839.1" XENTR_NCBI.gff|cut -f1|grep -wvf - XENTR_NCBI.gff >
XENTR_filt_NCBI.gff; rm XENTR_NCBI.gff
```

We use custom scripts to filters the longest isoforms (as identified by shared gene IDs). ENSEMBL gene ids are in the fasta headers. In the case of NCBI, gene IDs are not in the fasta file, so the annotation file (gff format) is also needed to determine to which gene the isoforms come from.

- ACAPL

```
LongestIsoforms_NCBI.py ../../proteins/ACAPL_NCBI_raw_pep.fa ACAPL_NCBI_filt.gff3
ACAPL_NCBI_longest_isoforms.fa
sed -i 's/^>/>ACAPL_/' ACAPL_NCBI_longest_isoforms.fa
pymakeMap.py -gff ACAPL_NCBI_filt.gff3 -p ACAPL -f CDS -k protein_id -o
../chrom/ACAPL.chrom -r fasta -F ACAPL_NCBI_longest_isoforms.fa
cut -f2 ../chrom/ACAPL.chrom > ACAPL.list ; pyfasta extract --file ACAPL.list --
header --space --fasta ACAPL_NCBI_longest_isoforms.fa >
../../proteins_processed/ACAPL.fasta
rm ACAPL_NCBI_longest_isoforms* ACAPL.list
```

- ACRMI

```
LongestIsoforms_NCBI.py ../../proteins/ACRMI_NCBI_raw_pep.fa ACRMI_NCBI.gff
ACRMI_NCBI_longest_isoforms.fa
sed -i 's/^>/>ACRMI_/' ACRMI_NCBI_longest_isoforms.fa
pymakeMap.py -gff ACRMI_NCBI.gff -p ACRMI -f CDS -k protein_id -o
../chrom/ACRMI.chrom -r fasta -F ACRMI_NCBI_longest_isoforms.fa
cut -f2 ../chrom/ACRMI.chrom > ACRMI.list ; pyfasta extract --file ACRMI.list --
header --space --fasta ACRMI_NCBI_longest_isoforms.fa >
../../proteins_processed/ACRMI.fasta
rm ACRMI_NCBI_longest_isoforms* ACRMI.list
```

- CALMI

```
LongestIsoforms_NCBI.py ../../proteins/CALMI_NCBI_raw_pep.fa CALMI_NCBI.gff3
CALMI_NCBI_longest_isoforms.fa
sed -i 's/^>/>CALMI_/' CALMI_NCBI_longest_isoforms.fa
pymakeMap.py -gff CALMI_NCBI.gff3 -p CALMI -f CDS -k protein_id -o
../chrom/CALMI.chrom -r fasta -F CALMI_NCBI_longest_isoforms.fa
cut -f2 ../chrom/CALMI.chrom > CALMI.list ; pyfasta extract --file CALMI.list --
header --space --fasta CALMI_NCBI_longest_isoforms.fa >
../../proteins_processed/CALMI.fasta
rm CALMI_NCBI_longest_isoforms* CALMI.list
```

- CHEMY

```
LongestIsoforms_NCBI.py ../../proteins/CHEMY_NCBI_raw_pep.fa CHEMY_NCBI_filt.gff
CHEMY_NCBI_longest_isoforms.fa
sed -i 's/^>/>CHEMY_/' CHEMY_NCBI_longest_isoforms.fa
pymakeMap.py -gff CHEMY_NCBI_filt.gff -p CHEMY -f CDS -k protein_id -o
../chrom/CHEMY.chrom -r fasta -F CHEMY_NCBI_longest_isoforms.fa
cut -f2 ../chrom/CHEMY.chrom > CHEMY.list ; pyfasta extract --file CHEMY.list --
header --space --fasta CHEMY_NCBI_longest_isoforms.fa >
../../proteins_processed/CHEMY.fasta
rm CHEMY_NCBI_longest_isoforms* CHEMY.list
```

- CIOIN

```
LongestIsoforms_NCBI.py ../../proteins/CIOIN_NCBI_raw_pep.fa CIOIN_NCBI_filt.gff3
CIOIN_NCBI_longest_isoforms.fa
sed -i 's/^>/>CIOIN_/' CIOIN_NCBI_longest_isoforms.fa
pymakeMap.py -gff CIOIN_NCBI_filt.gff3 -p CIOIN -f CDS -k protein_id -o
../chrom/CIOIN.chrom -r fasta -F CIOIN_NCBI_longest_isoforms.fa
cut -f2 ../chrom/CIOIN.chrom > CIOIN.list ; pyfasta extract --file CIOIN.list --
header --space --fasta CIOIN_NCBI_longest_isoforms.fa >
../../proteins_processed/CIOIN.fasta
rm CIOIN_NCBI_longest_isoforms* CIOIN.list
```

- DANRE

```
LongestIsoforms_NCBI.py ../../proteins/DANRE_NCBI_raw_pep.fa DANRE_filt_NCBI.gff3
DANRE_NCBI_longest_isoforms.fa
sed -i 's/^>/>DANRE_/' DANRE_NCBI_longest_isoforms.fa
pymakeMap.py -gff DANRE_filt_NCBI.gff3 -p DANRE -f CDS -k protein_id -o
../chrom/DANRE.chrom -r fasta -F DANRE_NCBI_longest_isoforms.fa
cut -f2 ../chrom/DANRE.chrom > DANRE.list ; pyfasta extract --file DANRE.list --
header --space --fasta DANRE_NCBI_longest_isoforms.fa >
../../proteins_processed/DANRE.fasta
rm DANRE_NCBI_longest_isoforms* DANRE.list
```

- EXAPA

```
LongestIsoforms_NCBI.py ../../proteins/EXAPA_NCBI_raw_pep.fa EXAPA_NCBI.gff
EXAPA_NCBI_longest_isoforms.fa
sed -i 's/^>/>EXAPA_/' EXAPA_NCBI_longest_isoforms.fa
pymakeMap.py -gff EXAPA_NCBI.gff -p EXAPA -f CDS -k protein_id -o
../chrom/EXAPA.chrom -r fasta -F EXAPA_NCBI_longest_isoforms.fa
cut -f2 ../chrom/EXAPA.chrom > EXAPA.list ; pyfasta extract --file EXAPA.list --
header --space --fasta EXAPA_NCBI_longest_isoforms.fa >
../../proteins_processed/EXAPA.fasta
rm EXAPA_NCBI_longest_isoforms* EXAPA.list
```

- GALGA

```
LongestIsoforms_NCBI.py ../../proteins/GALGA_NCBI_raw_pep.fa GALGA_filt_NCBI.gff3
GALGA_NCBI_longest_isoforms.fa
sed -i 's/^>/>GALGA_/' GALGA_NCBI_longest_isoforms.fa
pymakeMap.py -gff GALGA_filt_NCBI.gff3 -p GALGA -f CDS -k protein_id -o
../chrom/GALGA.chrom -r fasta -F GALGA_NCBI_longest_isoforms.fa
cut -f2 ../chrom/GALGA.chrom > GALGA.list ; pyfasta extract --file GALGA.list --
header --space --fasta GALGA_NCBI_longest_isoforms.fa >
../../proteins_processed/GALGA.fasta
rm GALGA_NCBI_longest_isoforms* GALGA.list
```

- HIPCO

```
LongestIsoforms_NCBI.py ../../proteins/HIPCO_NCBI_raw_pep.fa HIPCO_filt_NCBI.gff
HIPCO_NCBI_longest_isoforms.fa
sed -i 's/^>/>HIPCO_/' HIPCO_NCBI_longest_isoforms.fa
pymakeMap.py -gff HIPCO_filt_NCBI.gff -p HIPCO -f CDS -k protein_id -o
../chrom/HIPCO.chrom -r fasta -F HIPCO_NCBI_longest_isoforms.fa
cut -f2 ../chrom/HIPCO.chrom > HIPCO.list ; pyfasta extract --file HIPCO.list --
header --space --fasta HIPCO_NCBI_longest_isoforms.fa >
../../proteins_processed/HIPCO.fasta
rm HIPCO_NCBI_longest_isoforms* HIPCO.list
```

- HOMSA

```
LongestIsoforms_NCBI.py ../../proteins/HOMSA_NCBI_raw_pep.fa HOMSA_filt_NCBI.gff
HOMSA_NCBI_longest_isoforms.fa
sed -i 's/^>/>HOMSA_/' HOMSA_NCBI_longest_isoforms.fa
pymakeMap.py -gff HOMSA_filt_NCBI.gff -p HOMSA -f CDS -k protein_id -o
../chrom/HOMSA.chrom -r fasta -F HOMSA_NCBI_longest_isoforms.fa
cut -f2 ../chrom/HOMSA.chrom > HOMSA.list ; pyfasta extract --file HOMSA.list --
header --space --fasta HOMSA_NCBI_longest_isoforms.fa >
```

```
../../proteins_processed/HOMSA.fasta
rm HOMSA_NCBI_longest_isoforms* HOMSA.list
```

- LATCH

```
LongestIsoforms_NCBI.py ../../proteins/LATCH_NCBI_raw_pep.fa LATCH_filt_NCBI.gff
LATCH_NCBI_longest_isoforms.fa
sed -i 's/^>/>LATCH_/' LATCH_NCBI_longest_isoforms.fa
pymakeMap.py -gff LATCH_filt_NCBI.gff -p LATCH -f CDS -k protein_id -o
../chrom/LATCH.chrom -r fasta -F LATCH_NCBI_longest_isoforms.fa
cut -f2 ../chrom/LATCH.chrom > LATCH.list ; pyfasta extract --file LATCH.list --
header --space --fasta LATCH_NCBI_longest_isoforms.fa >
../../proteins_processed/LATCH.fasta
rm LATCH_NCBI_longest_isoforms* LATCH.list
```

- LEPOC

```
LongestIsoforms_NCBI.py ../../proteins/LEPOC_NCBI_raw_pep.fa LEPOC_filt_NCBI.gff3
LEPOC_NCBI_longest_isoforms.fa
sed -i 's/^>/>LEPOC_/' LEPOC_NCBI_longest_isoforms.fa
pymakeMap.py -gff LEPOC_filt_NCBI.gff3 -p LEPOC -f CDS -k protein_id -o
../chrom/LEPOC.chrom -r fasta -F LEPOC_NCBI_longest_isoforms.fa
cut -f2 ../chrom/LEPOC.chrom > LEPOC.list ; pyfasta extract --file LEPOC.list --
header --space --fasta LEPOC_NCBI_longest_isoforms.fa >
../../proteins_processed/LEPOC.fasta
rm LEPOC_NCBI_longest_isoforms* LEPOC.list
```

- MAYZE

```
LongestIsoforms_NCBI.py ../../proteins/MAYZE_NCBI_raw_pep.fa MAYZE_filt_NCBI.gff
MAYZE_NCBI_longest_isoforms.fa
sed -i 's/^>/>MAYZE_/' MAYZE_NCBI_longest_isoforms.fa
pymakeMap.py -gff MAYZE_filt_NCBI.gff -p MAYZE -f CDS -k protein_id -o
../chrom/MAYZE.chrom -r fasta -F MAYZE_NCBI_longest_isoforms.fa
cut -f2 ../chrom/MAYZE.chrom > MAYZE.list ; pyfasta extract --file MAYZE.list --
header --space --fasta MAYZE_NCBI_longest_isoforms.fa >
../../proteins_processed/MAYZE.fasta
rm MAYZE_NCBI_longest_isoforms* MAYZE.list
```

- MUSMU

```
LongestIsoforms_NCBI.py ../../proteins/MUSMU_NCBI_raw_pep.fa MUSMU_filt_NCBI.gff3
MUSMU_NCBI_longest_isoforms.fa
sed -i 's/^>/>MUSMU_/' MUSMU_NCBI_longest_isoforms.fa
pymakeMap.py -gff MUSMU_filt_NCBI.gff3 -p MUSMU -f CDS -k protein_id -o
```

```
../chrom/MUSMU.chrom -r fasta -F MUSMU_NCBI_longest_isoforms.fa
cut -f2 ../chrom/MUSMU.chrom > MUSMU.list ; pyfasta extract --file MUSMU.list --
header --space --fasta MUSMU_NCBI_longest_isoforms.fa >
../../proteins_processed/MUSMU.fasta
rm MUSMU_NCBI_longest_isoforms* MUSMU.list
```

- PARTE

```
LongestIsoforms_NCBI.py ../../proteins/PARTE_NCBI_raw_pep.fa PARTE_NCBI.gff3
PARTE_NCBI_longest_isoforms.fa
sed -i 's/^>/>PARTE_/' PARTE_NCBI_longest_isoforms.fa
pymakeMap.py -gff PARTE_NCBI.gff3 -p PARTE -f CDS -k protein_id -o
../chrom/PARTE.chrom -r fasta -F PARTE_NCBI_longest_isoforms.fa
cut -f2 ../chrom/PARTE.chrom > PARTE.list ; pyfasta extract --file PARTE.list --
header --space --fasta PARTE_NCBI_longest_isoforms.fa >
../../proteins_processed/PARTE.fasta
rm PARTE_NCBI_longest_isoforms* PARTE.list
```

- XENTR

```
LongestIsoforms_NCBI.py ../../proteins/XENTR_NCBI_raw_pep.fa XENTR_filt_NCBI.gff
XENTR_NCBI_longest_isoforms.fa
sed -i 's/^>/>XENTR_/' XENTR_NCBI_longest_isoforms.fa
pymakeMap.py -gff XENTR_filt_NCBI.gff -p XENTR -f CDS -k protein_id -o
../chrom/XENTR.chrom -r fasta -F XENTR_NCBI_longest_isoforms.fa
cut -f2 ../chrom/XENTR.chrom > XENTR.list ; pyfasta extract --file XENTR.list --
header --space --fasta XENTR_NCBI_longest_isoforms.fa >
../../proteins_processed/XENTR.fasta
rm XENTR_NCBI_longest_isoforms* XENTR.list
```

2.1.2 Prepare chrom files for ENSEMBL data

- ADIVA

```
LongestIsoforms_ENSEMBL.py ../../proteins/ADIVA_ENSEMBL_raw_pep.fa
ADIVA_ENSEMBL_longest_isoforms.fa
sed -i 's/^>/>ADIVA_/' ADIVA_ENSEMBL_longest_isoforms.fa
pymakeMap.py -gff ADIVA_ENSEMBL.gff3 -p ADIVA -f CDS -k protein_id -o
../chrom/ADIVA.chrom -r fasta -F ADIVA_ENSEMBL_longest_isoforms.fa
cut -f2 ../chrom/ADIVA.chrom > ADIVA.list ; pyfasta extract --file ADIVA.list --
header --space --fasta ADIVA_ENSEMBL_longest_isoforms.fa >
../../proteins_processed/ADIVA.fasta
rm ADIVA_ENSEMBL_longest_isoforms* ADIVA.list
```

- AMPQU

```
LongestIsoforms_ENSEMBL.py ../../proteins/AMPQU_ENSEMBL_raw_pep.fa
AMPQU_ENSEMBL_longest_isoforms.fa
sed -i 's/^>/>AMPQU_/' AMPQU_ENSEMBL_longest_isoforms.fa
pymakeMap.py -gff AMPQU_ENSEMBL.gff3 -p AMPQU -f CDS -k protein_id -o
../chrom/AMPQU.chrom -r fasta -F AMPQU_ENSEMBL_longest_isoforms.fa
cut -f2 ../chrom/AMPQU.chrom > AMPQU.list ; pyfasta extract --file AMPQU.list --
header --space --fasta AMPQU_ENSEMBL_longest_isoforms.fa >
../../proteins_processed/AMPQU.fasta
rm AMPQU_ENSEMBL_longest_isoforms* AMPQU.list
```

- ANOGA

```
grep -vP "Mt\tVectorBase"  ANOGA_ENSEMBL.gff3 > ANOGA_filt_ENSEMBL.gff3; rm
ANOGA_ENSEMBL.gff3
LongestIsoforms_ENSEMBL.py ../../proteins/ANOGA_ENSEMBL_raw_pep.fa
ANOGA_ENSEMBL_longest_isoforms.fa
sed -i 's/^>/>ANOGA_/' ANOGA_ENSEMBL_longest_isoforms.fa
pymakeMap.py -gff ANOGA_filt_ENSEMBL.gff3 -p ANOGA -f CDS -k protein_id -o
../chrom/ANOGA.chrom -r fasta -F ANOGA_ENSEMBL_longest_isoforms.fa
cut -f2 ../chrom/ANOGA.chrom > ANOGA.list ; pyfasta extract --file ANOGA.list --
header --space --fasta ANOGA_ENSEMBL_longest_isoforms.fa >
../../proteins_processed/ANOGA.fasta
rm ANOGA_ENSEMBL_longest_isoforms* ANOGA.list
```

- BRALA

```
LongestIsoforms_ENSEMBL.py ../../proteins/BRALA_ENSEMBL_raw_pep.fa
BRALA_ENSEMBL_longest_isoforms.fa
sed -i 's/^>/>BRALA_/' BRALA_ENSEMBL_longest_isoforms.fa
pymakeMap.py -gff BRALA_ENSEMBL.gff3 -p BRALA -f CDS -k protein_id -o
../chrom/BRALA.chrom -r fasta -F BRALA_ENSEMBL_longest_isoforms.fa
cut -f2 ../chrom/BRALA.chrom > BRALA.list ; pyfasta extract --file BRALA.list --
header --space --fasta BRALA_ENSEMBL_longest_isoforms.fa >
../../proteins_processed/BRALA.fasta
rm BRALA_ENSEMBL_longest_isoforms* BRALA.list
```

- CAEEL

```
grep -vP "MtDNA\tWormBase"  CAEEL_ENSEMBL.gff3 > CAEEL_filt_ENSEMBL.gff3; rm
CAEEL_ENSEMBL.gff3
LongestIsoforms_ENSEMBL.py ../../proteins/CAEEL_ENSEMBL_raw_pep.fa
CAEEL_ENSEMBL_longest_isoforms.fa
sed -i 's/^>/>CAEEL_/' CAEEL_ENSEMBL_longest_isoforms.fa
pymakeMap.py -gff CAEEL_filt_ENSEMBL.gff3 -p CAEEL -f CDS -k protein_id -o
../chrom/CAEEL.chrom -r fasta -F CAEEL_ENSEMBL_longest_isoforms.fa
cut -f2 ../chrom/CAEEL.chrom > CAEEL.list ; pyfasta extract --file CAEEL.list --
```

```
header --space --fasta CAEEL_ENSEMBL_longest_isoforms.fa >
../../proteins_processed/CAEEL.fasta
rm CAEEL_ENSEMBL_longest_isoforms* CAEEL.list
```

- CAPOW

```
LongestIsoforms_ENSEMBL.py ../../proteins/CAPOW_ENSEMBL_raw_pep.fa
CAPOW_ENSEMBL_longest_isoforms.fa
sed -i 's/^>/>CAPOW_/' CAPOW_ENSEMBL_longest_isoforms.fa
pymakeMap.py -gff CAPOW_ENSEMBL.gff3 -p CAPOW -f CDS -k protein_id -o
../chrom/CAPOW.chrom -r fasta -F CAPOW_ENSEMBL_longest_isoforms.fa
cut -f2 ../chrom/CAPOW.chrom > CAPOW.list ; pyfasta extract --file CAPOW.list --
header --space --fasta CAPOW_ENSEMBL_longest_isoforms.fa >
../../proteins_processed/CAPOW.fasta
rm CAPOW_ENSEMBL_longest_isoforms* CAPOW.list
```

- CAPTE

```
LongestIsoforms_ENSEMBL.py ../../proteins/CAPTE_ENSEMBL_raw_pep.fa
CAPTE_ENSEMBL_longest_isoforms.fa
sed -i 's/^>/>CAPTE_/' CAPTE_ENSEMBL_longest_isoforms.fa
pymakeMap.py -gff CAPTE_ENSEMBL.gff3 -p CAPTE -f CDS -k protein_id -o
../chrom/CAPTE.chrom -r fasta -F CAPTE_ENSEMBL_longest_isoforms.fa
cut -f2 ../chrom/CAPTE.chrom > CAPTE.list ; pyfasta extract --file CAPTE.list --
header --space --fasta CAPTE_ENSEMBL_longest_isoforms.fa >
../../proteins_processed/CAPTE.fasta
rm CAPTE_ENSEMBL_longest_isoforms* CAPTE.list
```

- CRAGI

```
LongestIsoforms_ENSEMBL.py ../../proteins/CRAGI_ENSEMBL_raw_pep.fa
CRAGI_ENSEMBL_longest_isoforms.fa
sed -i 's/^>/>CRAGI_/' CRAGI_ENSEMBL_longest_isoforms.fa
pymakeMap.py -gff CRAGI_ENSEMBL.gff3 -p CRAGI -f CDS -k protein_id -o
../chrom/CRAGI.chrom -r fasta -F CRAGI_ENSEMBL_longest_isoforms.fa
cut -f2 ../chrom/CRAGI.chrom > CRAGI.list ; pyfasta extract --file CRAGI.list --
header --space --fasta CRAGI_ENSEMBL_longest_isoforms.fa >
../../proteins_processed/CRAGI.fasta
rm CRAGI_ENSEMBL_longest_isoforms* CRAGI.list
```

- DAPPU

```
LongestIsoforms_ENSEMBL.py ../../proteins/DAPPU_ENSEMBL_raw_pep.fa
DAPPU_ENSEMBL_longest_isoforms.fa
sed -i 's/^>/>DAPPU_/' DAPPU_ENSEMBL_longest_isoforms.fa
```

```
pymakeMap.py -gff DAPPU_ENSEMBL.gff3 -p DAPPU -f CDS -k protein_id -o
../chrom/DAPPU.chrom -r fasta -F DAPPU_ENSEMBL_longest_isoforms.fa
cut -f2 ../chrom/DAPPU.chrom > DAPPU.list ; pyfasta extract --file DAPPU.list --
header --space --fasta DAPPU_ENSEMBL_longest_isoforms.fa >
../../proteins_processed/DAPPU.fasta
rm DAPPU_ENSEMBL_longest_isoforms* DAPPU.list
```

- DROME

```
grep -vP "mitochondrion_genome\tFlyBase"  DROME_ENSEMBL.gff3 >
DROME_filt_ENSEMBL.gff3; rm DROME_ENSEMBL.gff3
LongestIsoforms_ENSEMBL.py ../../proteins/DROME_ENSEMBL_raw_pep.fa
DROME_ENSEMBL_longest_isoforms.fa
sed -i 's/^>/>DROME_/' DROME_ENSEMBL_longest_isoforms.fa
pymakeMap.py -gff DROME_filt_ENSEMBL.gff3 -p DROME -f CDS -k protein_id -o
../chrom/DROME.chrom -r fasta -F DROME_ENSEMBL_longest_isoforms.fa
cut -f2 ../chrom/DROME.chrom > DROME.list ; pyfasta extract --file DROME.list --
header --space --fasta DROME_ENSEMBL_longest_isoforms.fa >
../../proteins_processed/DROME.fasta
rm DROME_ENSEMBL_longest_isoforms* DROME.list
```

- HELRO

```
LongestIsoforms_ENSEMBL.py ../../proteins/HELRO_ENSEMBL_raw_pep.fa
HELRO_ENSEMBL_longest_isoforms.fa
sed -i 's/^>/>HELRO_/' HELRO_ENSEMBL_longest_isoforms.fa
pymakeMap.py -gff HELRO_ENSEMBL.gff3 -p HELRO -f CDS -k protein_id -o
../chrom/HELRO.chrom -r fasta -F HELRO_ENSEMBL_longest_isoforms.fa
cut -f2 ../chrom/HELRO.chrom > HELRO.list ; pyfasta extract --file HELRO.list --
header --space --fasta HELRO_ENSEMBL_longest_isoforms.fa >
../../proteins_processed/HELRO.fasta
rm HELRO_ENSEMBL_longest_isoforms* HELRO.list
```

- IXOSC

```
LongestIsoforms_ENSEMBL.py ../../proteins/IXOSC_ENSEMBL_raw_pep.fa
IXOSC_ENSEMBL_longest_isoforms.fa
sed -i 's/^>/>IXOSC_/' IXOSC_ENSEMBL_longest_isoforms.fa
pymakeMap.py -gff IXOSC_ENSEMBL.gff3 -p IXOSC -f CDS -k protein_id -o
../chrom/IXOSC.chrom -r fasta -F IXOSC_ENSEMBL_longest_isoforms.fa
cut -f2 ../chrom/IXOSC.chrom > IXOSC.list ; pyfasta extract --file IXOSC.list --
header --space --fasta IXOSC_ENSEMBL_longest_isoforms.fa >
../../proteins_processed/IXOSC.fasta
rm IXOSC_ENSEMBL_longest_isoforms* IXOSC.list
```

- LINAN

```
LongestIsoforms_ENSEMBL.py ../../proteins/LINAN_ENSEMBL_raw_pep.fa
LINAN_ENSEMBL_longest_isoforms.fa
sed -i 's/^>/>LINAN_/' LINAN_ENSEMBL_longest_isoforms.fa
pymakeMap.py -gff LINAN_ENSEMBL.gff3 -p LINAN -f CDS -k protein_id -o
../chrom/LINAN.chrom -r fasta -F LINAN_ENSEMBL_longest_isoforms.fa
cut -f2 ../chrom/LINAN.chrom > LINAN.list ; pyfasta extract --file LINAN.list --
header --space --fasta LINAN_ENSEMBL_longest_isoforms.fa >
../../proteins_processed/LINAN.fasta
rm LINAN_ENSEMBL_longest_isoforms* LINAN.list
```

- LOTGI

```
LongestIsoforms_ENSEMBL.py ../../proteins/LOTGI_ENSEMBL_raw_pep.fa
LOTGI_ENSEMBL_longest_isoforms.fa
sed -i 's/^>/>LOTGI_/' LOTGI_ENSEMBL_longest_isoforms.fa
pymakeMap.py -gff LOTGI_ENSEMBL.gff3 -p LOTGI -f CDS -k protein_id -o
../chrom/LOTGI.chrom -r fasta -F LOTGI_ENSEMBL_longest_isoforms.fa
cut -f2 ../chrom/LOTGI.chrom > LOTGI.list ; pyfasta extract --file LOTGI.list --
header --space --fasta LOTGI_ENSEMBL_longest_isoforms.fa >
../../proteins_processed/LOTGI.fasta
rm LOTGI_ENSEMBL_longest_isoforms* LOTGI.list
```

- NEMVE

```
LongestIsoforms_ENSEMBL.py ../../proteins/NEMVE_ENSEMBL_raw_pep.fa
NEMVE_ENSEMBL_longest_isoforms.fa
sed -i 's/^>/>NEMVE_/' NEMVE_ENSEMBL_longest_isoforms.fa
pymakeMap.py -gff NEMVE_ENSEMBL.gff3 -p NEMVE -f CDS -k protein_id -o
../chrom/NEMVE.chrom -r fasta -F NEMVE_ENSEMBL_longest_isoforms.fa
cut -f2 ../chrom/NEMVE.chrom > NEMVE.list ; pyfasta extract --file NEMVE.list --
header --space --fasta NEMVE_ENSEMBL_longest_isoforms.fa >
../../proteins_processed/NEMVE.fasta
rm NEMVE_ENSEMBL_longest_isoforms* NEMVE.list
```

- SALRO

```
LongestIsoforms_ENSEMBL.py ../../proteins/SALRO_ENSEMBL_raw_pep.fa
SALRO_ENSEMBL_longest_isoforms.fa
sed -i 's/^>/>SALRO_/' SALRO_ENSEMBL_longest_isoforms.fa
pymakeMap.py -gff SALRO_ENSEMBL.gff3 -p SALRO -f CDS -k protein_id -o
../chrom/SALRO.chrom -r fasta -F SALRO_ENSEMBL_longest_isoforms.fa
cut -f2 ../chrom/SALRO.chrom > SALRO.list ; pyfasta extract --file SALRO.list --
header --space --fasta SALRO_ENSEMBL_longest_isoforms.fa >
../../proteins_processed/SALRO.fasta
rm SALRO_ENSEMBL_longest_isoforms* SALRO.list
```

- STRMA

```
LongestIsoforms_ENSEMBL.py ../../proteins/STRMA_ENSEMBL_raw_pep.fa
STRMA_ENSEMBL_longest_isoforms.fa
sed -i 's/^>/>STRMA_/' STRMA_ENSEMBL_longest_isoforms.fa
pymakeMap.py -gff STRMA_ENSEMBL.gff3 -p STRMA -f CDS -k protein_id -o
../chrom/STRMA.chrom -r fasta -F STRMA_ENSEMBL_longest_isoforms.fa
cut -f2 ../chrom/STRMA.chrom > STRMA.list ; pyfasta extract --file STRMA.list --
header --space --fasta STRMA_ENSEMBL_longest_isoforms.fa >
../../proteins_processed/STRMA.fasta
rm STRMA_ENSEMBL_longest_isoforms* STRMA.list
```

- STRPU, we'll use `-d` flag of pymakemap to delete isoforms registered with different gene ids. It deletes 2891 proteins This flag deletes overlapping genes with the following criteria:
- isoform with 1 exon is merged with overlapping isoform of 1+ size if any exon is overlapping on more than 20 base pairs
- isoform with 2 exons is merged with overlapping isoform of 2+ size if exons share at least a start or a stop
- isoform with 3 exons is merged with overlapping isoform of 3+ size if they share at least one internal exon

```
LongestIsoforms_ENSEMBL.py ../../proteins/STRPU_ENSEMBL_raw_pep.fa
STRPU_ENSEMBL_longest_isoforms.fa
sed -i 's/^>/>STRPU_/' STRPU_ENSEMBL_longest_isoforms.fa
pymakeMap.py -gff STRPU_ENSEMBL.gff3 -p STRPU -f CDS -k protein_id -o
../chrom/STRPU.chrom -r fasta -F STRPU_ENSEMBL_longest_isoforms.fa
cut -f2 ../chrom/STRPU.chrom > STRPU.list ; pyfasta extract --file STRPU.list --
header --space --fasta STRPU_ENSEMBL_longest_isoforms.fa >
../../proteins_processed/STRPU.fasta
rm STRPU_ENSEMBL_longest_isoforms* STRPU.list
```

- TRIAD

```
LongestIsoforms_ENSEMBL.py ../../proteins/TRIAD_ENSEMBL_raw_pep.fa
TRIAD_ENSEMBL_longest_isoforms.fa
sed -i 's/^>/>TRIAD_/' TRIAD_ENSEMBL_longest_isoforms.fa
pymakeMap.py -gff TRIAD_ENSEMBL.gff3 -p TRIAD -f CDS -k protein_id -o
../chrom/TRIAD.chrom -r fasta -F TRIAD_ENSEMBL_longest_isoforms.fa
cut -f2 ../chrom/TRIAD.chrom > TRIAD.list ; pyfasta extract --file TRIAD.list --
header --space --fasta TRIAD_ENSEMBL_longest_isoforms.fa >
../../proteins_processed/TRIAD.fasta
rm TRIAD_ENSEMBL_longest_isoforms* TRIAD.list
```

- TRICA

```
LongestIsoforms_ENSEMBL.py ../../proteins/TRICA_ENSEMBL_raw_pep.fa
TRICA_ENSEMBL_longest_isoforms.fa
sed -i 's/^>/>TRICA_/' TRICA_ENSEMBL_longest_isoforms.fa
pymakeMap.py -gff TRICA_ENSEMBL.gff3 -p TRICA -f CDS -k protein_id -o
../chrom/TRICA.chrom -r fasta -F TRICA_ENSEMBL_longest_isoforms.fa
cut -f2 ../chrom/TRICA.chrom > TRICA.list ; pyfasta extract --file TRICA.list --
header --space --fasta TRICA_ENSEMBL_longest_isoforms.fa >
../../proteins_processed/TRICA.fasta
rm TRICA_ENSEMBL_longest_isoforms* TRICA.list
```

2.1.3 Prepare chrom files for the data from OTHER sources

2.1.3.1 Prepare data with transcript variants

- HOIHO

```
LongestIsoforms_HOIHO_HYDVU_PTYFL.py ../../proteins/HOIHO_OTHER_raw_pep.fa
HOIHO_OTHER_longest_isoforms.fa
sed -i 's/^>/>HOIHO_/' HOIHO_OTHER_longest_isoforms.fa
pymakeMap.py -gff HOIHO_OTHER.gff3 -p HOIHO -f CDS -k Name -o ../chrom/HOIHO.chrom
-r fasta -F HOIHO_OTHER_longest_isoforms.fa
cut -f2 ../chrom/HOIHO.chrom > HOIHO.list ; pyfasta extract --file HOIHO.list --
header --space --fasta HOIHO_OTHER_longest_isoforms.fa >
../../proteins_processed/HOIHO.fasta
rm HOIHO_OTHER_longest_isoforms* HOIHO.list
```

- HYDVU

```
LongestIsoforms_HOIHO_HYDVU_PTYFL.py ../../proteins/HYDVU_OTHER_raw_pep.fa
HYDVU_OTHER_longest_isoforms.fa
sed -i 's/^>/>HYDVU_/' HYDVU_OTHER_longest_isoforms.fa
pymakeMap.py -gff HYDVU_OTHER.gff3 -p HYDVU -f CDS -k Parent -o
../chrom/HYDVU.chrom -r fasta -F HYDVU_OTHER_longest_isoforms.fa
cut -f2 ../chrom/HYDVU.chrom > HYDVU.list ; pyfasta extract --file HYDVU.list --
header --space --fasta HYDVU_OTHER_longest_isoforms.fa >
../../proteins_processed/HYDVU.fasta
rm HYDVU_OTHER_longest_isoforms* HYDVU.list
```

- PTYFL

```
LongestIsoforms_HOIHO_HYDVU_PTYFL.py ../../proteins/PTYFL_OTHER_raw_pep.fa
PTYFL_OTHER_longest_isoforms.fa
sed -i 's/^>/>PTYFL_/' PTYFL_OTHER_longest_isoforms.fa
pymakeMap.py -gff PTYFL_OTHER.gff3 -p PTYFL -f CDS -k Parent -o
../chrom/PTYFL.chrom -r fasta -F PTYFL_OTHER_longest_isoforms.fa
cut -f2 ../chrom/PTYFL.chrom > PTYFL.list ; pyfasta extract --file PTYFL.list --
```

```
header --space --fasta PTYFL_OTHER_longest_isoforms.fa >
../../proteins_processed/PTYFL.fasta
rm PTYFL_OTHER_longest_isoforms* PTYFL.list
```

- SCHME

```
LongestIsoforms_SCHME.py ../../proteins/SCHME_OTHER_raw_pep.fa SCHME_OTHER.gff3
SCHME_OTHER_longest_isoforms.fa
sed -i 's/^>/>SCHME_/' SCHME_OTHER_longest_isoforms.fa
pymakeMap.py -gff SCHME_OTHER.gff3 -p SCHME -f CDS -k Parent -o
../chrom/SCHME.chrom -r fasta -F SCHME_OTHER_longest_isoforms.fa
cut -f2 ../chrom/SCHME.chrom > SCHME.list ; pyfasta extract --file SCHME.list --
header --space --fasta SCHME_OTHER_longest_isoforms.fa >
../../proteins_processed/SCHME.fasta
rm SCHME_OTHER_longest_isoforms* SCHME.list
```

2.1.3.2 Prepare data without transcript variants

For all the following animals, proteomes are already filtered for redundancy

- AURAU

```
sed 's/^>/>AURAU_/' ../../proteins/AURAU_OTHER_raw_pep.fa >
AURAU_OTHER_longest_isoforms.fa
pymakeMap.py -gff AURAU_other.gff3 -p AURAU -f CDS -k protein_id -o
../chrom/AURAU.chrom -r fasta -F AURAU_OTHER_longest_isoforms.fa
cut -f2 ../chrom/AURAU.chrom > AURAU.list ; pyfasta extract --file AURAU.list --
header --space --fasta AURAU_OTHER_longest_isoforms.fa >
../../proteins_processed/AURAU.fasta
rm AURAU_OTHER_longest_isoforms* AURAU.list
```

- CLYHE

```
sed 's/^>\([^-]*\)-protein/>CLYHE_\1/' ../../proteins/CLYHE_OTHER_raw_pep.fa >
CLYHE_OTHER_longest_isoforms.fa
pymakeMap.py -gff CLYHE_OTHER.gff3 -p CLYHE -f exon -k Parent -o
../chrom/CLYHE.chrom -r fasta -F CLYHE_OTHER_longest_isoforms.fa
cut -f2 ../chrom/CLYHE.chrom > CLYHE.list ; pyfasta extract --file CLYHE.list --
header --space --fasta CLYHE_OTHER_longest_isoforms.fa >
../../proteins_processed/CLYHE.fasta
rm CLYHE_OTHER_longest_isoforms* CLYHE.list
```

- EUPSC

```
sed 's/\tcluster_/\tID=cluster_/' esc_allchroms_uniq_15jul2019.gff > EUPSC.gff
pymakeMap.py -gff EUPSC.gff -p EUPSC -f exon -k ID -o ../chrom/EUPSC.chrom -r
fasta -F EUPSC_OTHER_longest_isoforms.fa
cut -f2 ../chrom/EUPSC.chrom > EUPSC.list ; pyfasta extract --file EUPSC.list --
header --space --fasta EUPSC_OTHER_longest_isoforms.fa >
../../proteins_processed/EUPSC.fasta
rm EUPSC_OTHER_longest_isoforms* EUPSC.list
```

- HOFMI

```
sed 's/^>\([^_]\+_[^_]\+\)_\(g[0-9]\+.t[0-9]\)/>HOFMI_\2\ \1/'
../../proteins/HOFMI_OTHER_raw_pep.fa > HOFMI_OTHER_longest_isoforms.fa
pymakeMap.py -gff HOFMI_OTHER.gff3 -p HOFMI -f CDS -k Parent -o
../chrom/HOFMI.chrom -r fasta -F HOFMI_OTHER_longest_isoforms.fa
cut -f2 ../chrom/HOFMI.chrom > HOFMI.list ; pyfasta extract --file HOFMI.list --
header --space --fasta HOFMI_OTHER_longest_isoforms.fa >
../../proteins_processed/HOFMI.fasta
rm HOFMI_OTHER_longest_isoforms* HOFMI.list
```

- MIZYE

```
sed 's/^>/>MIZYE_/' ../../proteins/MIZYE_OTHER_raw_pep.fa >
MIZYE_OTHER_longest_isoforms.fa
pymakeMap.py -gff SCALLOP/chr.id.gff3 -p MIZYE -f CDS -k Parent -o
../chrom/MIZYE.chrom -r fasta -F MIZYE_OTHER_longest_isoforms.fa
cut -f2 ../chrom/MIZYE.chrom > MIZYE.list ; pyfasta extract --file MIZYE.list --
header --space --fasta MIZYE_OTHER_longest_isoforms.fa >
../../proteins_processed/MIZYE.fasta
rm MIZYE_OTHER_longest_isoforms* MIZYE.list
```

- MNELE

```
sed 's/^>/>MNELE_/' ../../proteins/MNELE_OTHER_raw_pep.fa >
MNELE_OTHER_longest_isoforms.fa
pymakeMap.py -gff MNELE_OTHER.gff3 -p MNELE -f CDS -k Parent -o
../chrom/MNELE.chrom -r fasta -F MNELE_OTHER_longest_isoforms.fa
cut -f2 ../chrom/MNELE.chrom > MNELE.list ; pyfasta extract --file MNELE.list --
header --space --fasta MNELE_OTHER_longest_isoforms.fa >
../../proteins_processed/MNELE.fasta
rm MNELE_OTHER_longest_isoforms* MNELE.list
```

- PLEBA

```
sed 's/^>/>PLEBA_/' ../../proteins/PLEBA_OTHER_raw_pep.fa >
PLEBA_OTHER_longest_isoforms.fa
pymakeMap.py -gff PLEBA_mapped_final.gff3 -p PLEBA -f exon -k Name -o
../chrom/PLEBA.chrom -r fasta -F PLEBA_OTHER_longest_isoforms.fa
cut -f2 ../chrom/PLEBA.chrom > PLEBA.list ; pyfasta extract --file PLEBA.list --
header --space --fasta PLEBA_OTHER_longest_isoforms.fa >
../../proteins_processed/PLEBA.fasta
rm PLEBA_OTHER_longest_isoforms* PLEBA.list
```

- SACKO

```
sed 's/^>/>SACKO_/' ../../proteins/SACKO_OTHER_raw_pep.fa >
SACKO_OTHER_longest_isoforms.fa
pymakeMap.py -gff SACKO_prepped.gff3 -p SACKO -f CDS -k protein_id -o
../chrom/SACKO.chrom -r fasta -F SACKO_OTHER_longest_isoforms.fa
cut -f2 ../chrom/SACKO.chrom > SACKO.list ; pyfasta extract --file SACKO.list --
header --space --fasta SACKO_OTHER_longest_isoforms.fa >
../../proteins_processed/SACKO.fasta
rm SACKO_OTHER_longest_isoforms* SACKO.list
```

- SYCCI

```
sed 's/^>/>SYCCI_/' ../../proteins/SYCCI_OTHER_raw_pep.fa >
SYCCI_OTHER_longest_isoforms.fa
pymakeMap.py -gff SYCCI_mapped_final.gff3 -p SYCCI -f exon -k Name -o
../chrom/SYCCI.chrom -r fasta -F SYCCI_OTHER_longest_isoforms.fa -d
cut -f2 ../chrom/SYCCI.chrom > SYCCI.list ; pyfasta extract --file SYCCI.list --
header --space --fasta SYCCI_OTHER_longest_isoforms.fa >
../../proteins_processed/SYCCI.fasta
rm SYCCI_OTHER_longest_isoforms* SYCCI.list
```

3. Orthology assignment

First, we want to generate the blast commands in preparation for orthofinder. Small check that the proteins do not end with symbols such as * or . to represent translated STOP codons, this trips up makeblastdb.

```
sed -i 's/*//' EUPSC.fasta
sed -i 's/*//' PLEBA.fasta
sed -i 's/*//' SACKO.fasta
sed -i 's/\([A-Z]\)\.\([A-Z]\)/\1\2/g' HOFMI.fasta
sed -i 's/\([A-Z]\)\.\([A-Z]\)/\1\2/g' SCHME.fasta

module load orthofinder ncbiblastplus
orthofinder -S blast -f 00_orthology_assignment/proteins_processed/ -op
```

Next, we want to enqueue the jobs. We thus use slurmtasks and tmprewrite to build the slurm script (https://github.com/nijibabulu/slurm-utils).

```
module load ncbiblastplus


for i in $(seq 0 48); do touch
00_orthology_assignment/proteins_processed/OrthoFinder/Results_Nov15/WorkingDirect
ory/BlastDBSpecies$i; done
for i in $(seq 0 48); do
  for j in $(seq 0 48); do

faspeciesi="00_orthology_assignment/proteins_processed/OrthoFinder/Results_Nov15/W
orkingDirectory/Species${i}.fa"

DBspeciesj="00_orthology_assignment/proteins_processed/OrthoFinder/Results_Nov15/W
orkingDirectory/BlastDBSpecies${j}"

outfile="00_orthology_assignment/proteins_processed/OrthoFinder/Results_Nov15/Work
ingDirectory/Blast${i}_${j}.txt"
    tmprewrite "echo {$DBspeciesj.phr:i} {$DBspeciesj.pin:i} {$DBspeciesj.psq:i};
blastp -num_threads 8 -outfmt 6 -evalue 0.001 -query {$faspeciesi:i} -db
{$DBspeciesj:i} -out {$outfile:o}"
  done
done > 00_orthology_assignment/proteins_processed/ofblast
sed -i 's/echo\ [^;]*;\ //' ofblast

slurmtasks -p 8core 00_orthology_assignment/proteins_processed/ofblast| sbatch --
array=1-192
slurmtasks -p 8core 00_orthology_assignment/proteins_processed/ofblast| sbatch
```

Now we want to run the graph building (Orthofinder) and clustering (MCL).

```
module load orthofinder
orthofinder -og -b
00_orthology_assignment/proteins_processed/OrthoFinder/Results_Nov15/WorkingDirect
ory
# convert file to clus
orthoFinderToOrthogroup.pl
00_orthology_assignment/proteins_processed/OrthoFinder/Results_Nov15/WorkingDirect
ory/Orthogroups.txt > 01_microsynteny/Orthofinder.clus
```## 4. Infer microsyntenic blocks
The two perl scripts `prepMicroSynt.pl` and `makeClusters3.pl` can be found at
https://github.com/nijibabulu/metazoan_synteny/tree/master/scripts. These scripts
are part of the microsynteny pipeline intially published in Simakov et al. 2013
(10.1038/nature11696):

4.1 randomize genomes
We'll randomize the genomes 3 times. Using the randomizations for both approaches.
```
```

```
we use `ls` to get a list of chrom files, and pymakeRandChrom takes the list from
stdin.
```

ls 01_microsynteny/chrom/*.chrom | pymakeRandChrom.py -n 3 -o 01_microsynteny/randomized_chrom

```
### 4.2 Microsynteny computed using Orthofinder's OGs
First, let's compute the microsynteny with the Orthofinder clus file
```

cd 01_microsynteny/chrom prepMicroSynt.pl ACAPL.chrom,ACRMI.chrom,ADIVA.chrom,AMPQU.chrom,ANOGA.chrom,AURAU.chrom,BRALA.chrom,CAEEL.chrom,CALMI.chrom,CAPOW.chrom,CAPTE.chrom,CHEMY.chrom,CIOIN.chrom,CLYHE.chrom,CRAGI.chrom,DANRE.chrom,DAPPU.chrom,DROME.chrom,EUPSC.chrom,EXAPA.chrom,GALGA.chrom,HELRO.chrom,HIPCO.chrom,HOFMI.chrom,HOIHO.chrom,HOMSA.chrom,HYDVU.chrom,IXOSC.chrom,LATCH.chrom,LEPOC.chrom,LINAN.chrom,LOTGI.chrom,MAYZE.chrom,MIZYE.chrom,MNELE.chrom,MUSMU.chrom,NEMVE.chrom,PARTE.chrom,PLEBA.chrom,PTYFL.chrom,SACKO.chrom,SALRO.chrom,SCHME.chrom,STRMA.chrom,STRPU.chrom,SYCCI.chrom,TRIAD.chrom,TRICA.chrom,XENTR.chrom 5 01_microsynteny/Orthofinder.clus sbatch --array=1-1176 --constraint=array-1core --job-name=synt_of_OBS job.sh

#reorganize a bit the files. mkdir bash_scripts log_files pairwise_blocks; mv *.out log_files; mv *.sh bash_scripts; mv *.blocks pairwise_blocks

CHROMS=../../chrom/ACAPL.chrom,../../chrom/ACRMI.chrom,../../chrom/ADIVA.chrom,../../chrom/AMPQU.chrom,../../chrom/ANOGA.chrom,../../chrom/AURAU.chrom,../../chrom/BRALA.chrom,../../chrom/CAEEL.chrom,../../chrom/CALMI.chrom,../../chrom/CAPOW.chrom,../../chrom/CAPTE.chrom,../../chrom/CHEMY.chrom,../../chrom/CIOIN.chrom,../../chrom/CLYHE.chrom,../../chrom/CRAGI.chrom,../../chrom/DANRE.chrom,../../chrom/DAPPU.chrom,../../chrom/DROME.chrom,../../chrom/EUPSC.chrom,../../chrom/EXAPA.chrom,../../chrom/GALGA.chrom,../../chrom/HELRO.chrom,../../chrom/HIPCO.chrom,../../chrom/HOFMI.chrom,../../chrom/HOIHO.chrom,../../chrom/HOMSA.chrom,../../chrom/HYDVU.chrom,../../chrom/IXOSC.chrom,../../chrom/LATCH.chrom,../../chrom/LEPOC.chrom,../../chrom/LINAN.chrom,../../chrom/LOTGI.chrom,../../chrom/MAYZE.chrom,../../chrom/MIZYE.chrom,../../chrom/MNELE.chrom,../../chrom/MUSMU.chrom,../../chrom/NEMVE.chrom,../../chrom/PARTE.chrom,../../chrom/PLEBA.chrom,../../chrom/PTYFL.chrom,../../chrom/SACKO.chrom,../../chrom/SALRO.chrom,../../chrom/SCHME.chrom,../../chrom/STRMA.chrom,../../chrom/STRPU.chrom,../../chrom/SYCCI.chrom,../../chrom/TRIAD.chrom,../../chrom/TRICA.chrom,../../chrom/XENTR.chrom

cd 01_microsynteny/chrom_of/pairwise_blocks makeClusters3.pl $CHROMS .5.blocks 3 0.3 0.5 &> 5.blocks.3.syn.synt

#correct the coordinates in the synt file, coordinates outputted by perl scripts are not exact block boundaries. correct_blocks_coordinates.py 5.blocks.3.syn.synt $CHROMS > 5.blocks.3.syn_corrected.synt

```
Now, for the first randomized genome
```

cd 01_microsynteny/randomized_chrom/rand.1/Orthofinder prepMicroSynt.pl ../ACAPL.chrom.rand.1,../ACRMI.chrom.rand.1,../ADIVA.chrom.rand.1,../AMPQU.chrom.rand.1,../ANOGA.chrom.rand.1,../AURAU.chrom.rand.1,../BRALA.chrom.rand.1,../CAEEL.chrom.rand.1,../CALMI.chrom.rand.1,../CAPOW.chrom.rand.1,../CAPTE.chrom.rand.1,../CHEMY.chrom.rand.1,../CIOIN.chrom.rand.1,../CLYHE.chrom.rand.1,../CRAGI.chrom.rand.1,../DANRE.chrom.rand.1,../DAPPU.chrom.rand.1,../DROME.chrom.rand.1,../EUPSC.chrom.rand.1,../EXAPA.chrom.rand.1,../GALGA.chrom.rand.1,../HELRO.chrom.rand.1,../HIPCO.chrom.rand.1,../HOFMI.chrom.rand.1,../HOIHO.chrom.rand.1,../HOMSA.chrom.rand.1,../HYDVU.chrom.rand.1,../IXOSC.chrom.rand.1,../LATCH.chrom.rand.1,../LEPOC.chrom.rand.1,../LINAN.chrom.rand.1,../LOTGI.chrom.rand.1,../MAYZE.chrom.rand.1,../MIZYE.chrom.rand.1,../MNELE.chrom.rand.1,../MUSMU.chrom.rand.1,../NEMVE.chrom.rand.1,../PARTE.chrom.rand.1,../PLEBA.chrom.rand.1,../PTYFL.chrom.rand.1,../SACKO.chrom.rand.1,../SALRO.chrom.rand.1,../SCHME.chrom.rand.1,../STRMA.chrom.rand.1,../STRPU.chrom.rand.1,../SYCCI.chrom.rand.1,../TRIAD.chrom.rand.1,../TRICA.chrom.rand.1,../XENTR.chrom.rand.1 5 01_microsynteny/Orthofinder.clus sbatch --array=1-1176 --constraint=array-1core --job-name=synt_of_RAND1 job.sh

CHROMS_RAND1="../../ACAPL.chrom.rand.1,../../ACRMI.chrom.rand.1,../../ADIVA.chrom.rand.1,../../AMPQU.chrom.rand.1,../../ANOGA.chrom.rand.1,../../AURAU.chrom.rand.1,../../BRALA.chrom.rand.1,../../CAEEL.chrom.rand.1,../../CALMI.chrom.rand.1,../../CAPOW.chrom.rand.1,../../CAPTE.chrom.rand.1,../../CHEMY.chrom.rand.1,../../CIOIN.chrom.rand.1,../../CLYHE.chrom.rand.1,../../CRAGI.chrom.rand.1,../../DANRE.chrom.rand.1,../../DAPPU.chrom.rand.1,../../DROME.chrom.rand.1,../../EUPSC.chrom.rand.1,../../EXAPA.chrom.rand.1,../../GALGA.chrom.rand.1,../../HELRO.chrom.rand.1,../../HIPCO.chrom.rand.1,../../HOFMI.chrom.rand.1,../../HOIHO.chrom.rand.1,../../HOMSA.chrom.rand.1,../../HYDVU.chrom.rand.1,../../IXOSC.chrom.rand.1,../../LATCH.chrom.rand.1,../../LEPOC.chrom.rand.1,../../LINAN.chrom.rand.1,../../LOTGI.chrom.rand.1,../../MAYZE.chrom.rand.1,../../MIZYE.chrom.rand.1,../../MNELE.chrom.rand.1,../../MUSMU.chrom.rand.1,../../NEMVE.chrom.rand.1,../../PARTE.chrom.rand.1,../../PLEBA.chrom.rand.1,../../PTYFL.chrom.rand.1,../../SACKO.chrom.rand.1,../../SALRO.chrom.rand.1,../../SCHME.chrom.rand.1,../../STRMA.chrom.rand.1,../../STRPU.chrom.rand.1,../../SYCCI.chrom.rand.1,../../TRIAD.chrom.rand.1,../../TRICA.chrom.rand.1,../../XENTR.chrom.rand.1"

mkdir bash_scripts log_files pairwise_blocks; mv *.out log_files; mv *.sh bash_scripts; mv *.blocks pairwise_blocks cd 01_microsynteny/randomized_chrom/rand.1/Orthofinder/pairwise_blocks makeClusters3.pl $CHROMS_RAND1 .5.blocks 3 0.3 0.5 > 5.blocks.3.syn.synt

correct_blocks_coordinates.py 5.blocks.3.syn.synt $CHROMS_RAND1 > 5.blocks.3.syn_corrected.synt

```
The second randomized genome
```

cd 01_microsynteny/randomized_chrom/rand.2/Orthofinder prepMicroSynt.pl ../ACAPL.chrom.rand.2,../ACRMI.chrom.rand.2,../ADIVA.chrom.rand.2,../AMPQU.chrom.rand.2,../ANOGA.chrom.rand.2,../AURAU.chrom.rand.2,../BRALA.chrom.rand.2,../CAEEL.chrom.rand.2,../CALMI.chrom.rand.2,../CAPOW.chrom.rand.2,../CAPTE.chrom.rand.2,../CHEMY.chrom.rand.2,../CIOIN.chrom.rand.2,../CLYHE.chrom.rand.2,../CRAGI.chrom.rand.2,../DANRE.chrom.rand.2,../DAPPU.chrom.rand.2,../DROME.chrom.rand.2,../EUPSC.chrom.rand.2,../EXAPA.chrom.rand.2,../GALGA.chrom.rand.2,../HELRO.chrom.rand.2,../HIPCO.chrom.rand.2,../HOFMI.chrom.rand.2,../HOIHO.chrom.rand.2,../HOMSA.chrom.rand.2,../HYDVU.chrom.rand.2,../IXOSC.chrom.rand.2,../LATCH.chrom.rand.2,../LEPOC.chrom.rand.2,../LINAN.chrom.rand.2,../LOTGI.chrom.rand.2,../MAYZE.chrom.rand.2,../MIZYE.chrom.rand.2,../MNELE.chrom.rand.2,../MUSMU.chrom.rand.2,../NEMVE.chrom.rand.2,../PARTE.chrom.rand.2,../PLE

BA.chrom.rand.2,../PTYFL.chrom.rand.2,../SACKO.chrom.rand.2,../SALRO.chrom.rand.2,../SCHME.chrom.rand.2,../STRMA.chrom.rand.2,../STRPU.chrom.rand.2,../SYCCI.chrom.rand.2,../TRIAD.chrom.rand.2,../TRICA.chrom.rand.2,../XENTR.chrom.rand.2 5 01_microsynteny/Orthofinder.clus sbatch --array=1-1176 --constraint=array-1core --job-name=synt_of_RAND2 job.sh

CHROM_RAND2="../../ACAPL.chrom.rand.2,../../ACRMI.chrom.rand.2,../../ADIVA.chrom.rand.2,../../AMPQU.chrom.rand.2,../../ANOGA.chrom.rand.2,../../AURAU.chrom.rand.2,../../BRALA.chrom.rand.2,../../CAEEL.chrom.rand.2,../../CALMI.chrom.rand.2,../../CAPOW.chrom.rand.2,../../CAPTE.chrom.rand.2,../../CHEMY.chrom.rand.2,../../CIOIN.chrom.rand.2,../../CLYHE.chrom.rand.2,../../CRAGI.chrom.rand.2,../../DANRE.chrom.rand.2,../../DAPPU.chrom.rand.2,../../DROME.chrom.rand.2,../../EUPSC.chrom.rand.2,../../EXAPA.chrom.rand.2,../../GALGA.chrom.rand.2,../../HELRO.chrom.rand.2,../../HIPCO.chrom.rand.2,../../HOFMI.chrom.rand.2,../../HOIHO.chrom.rand.2,../../HOMSA.chrom.rand.2,../../HYDVU.chrom.rand.2,../../IXOSC.chrom.rand.2,../../LATCH.chrom.rand.2,../../LEPOC.chrom.rand.2,../../LINAN.chrom.rand.2,../../LOTGI.chrom.rand.2,../../MAYZE.chrom.rand.2,../../MIZYE.chrom.rand.2,../../MNELE.chrom.rand.2,../../MUSMU.chrom.rand.2,../../NEMVE.chrom.rand.2,../../PARTE.chrom.rand.2,../../PLEBA.chrom.rand.2,../../PTYFL.chrom.rand.2,../../SACKO.chrom.rand.2,../../SALRO.chrom.rand.2,../../SCHME.chrom.rand.2,../../STRMA.chrom.rand.2,../../STRPU.chrom.rand.2,../../SYCCI.chrom.rand.2,../../TRIAD.chrom.rand.2,../../TRICA.chrom.rand.2,../../XENTR.chrom.rand.2"

mkdir bash_scripts log_files pairwise_blocks; mv *.out log_files; mv *.sh bash_scripts; mv *.blocks pairwise_blocks cd 01_microsynteny/randomized_chrom/rand.1/Orthofinder/pairwise_blocks makeClusters3.pl $CHROM_RAND2 3 0.3 0.5 > 5.blocks.3.syn.synt

correct_blocks_coordinates.py 5.blocks.3.syn.synt $CHROM_RAND2 > 5.blocks.3.syn_corrected.synt

```
And the third
```

cd 01_microsynteny/randomized_chrom/rand.3/Orthofinder prepMicroSynt.pl ../ACAPL.chrom.rand.3,../ACRMI.chrom.rand.3,../ADIVA.chrom.rand.3,../AMPQU.chrom.rand.3,../ANOGA.chrom.rand.3,../AURAU.chrom.rand.3,../BRALA.chrom.rand.3,../CAEEL.chrom.rand.3,../CALMI.chrom.rand.3,../CAPOW.chrom.rand.3,../CAPTE.chrom.rand.3,../CHEMY.chrom.rand.3,../CIOIN.chrom.rand.3,../CLYHE.chrom.rand.3,../CRAGI.chrom.rand.3,../DANRE.chrom.rand.3,../DAPPU.chrom.rand.3,../DROME.chrom.rand.3,../EUPSC.chrom.rand.3,../EXAPA.chrom.rand.3,../GALGA.chrom.rand.3,../HELRO.chrom.rand.3,../HIPCO.chrom.rand.3,../HOFMI.chrom.rand.3,../HOIHO.chrom.rand.3,../HOMSA.chrom.rand.3,../HYDVU.chrom.rand.3,../IXOSC.chrom.rand.3,../LATCH.chrom.rand.3,../LEPOC.chrom.rand.3,../LINAN.chrom.rand.3,../LOTGI.chrom.rand.3,../MAYZE.chrom.rand.3,../MIZYE.chrom.rand.3,../MNELE.chrom.rand.3,../MUSMU.chrom.rand.3,../NEMVE.chrom.rand.3,../PARTE.chrom.rand.3,../PLEBA.chrom.rand.3,../PTYFL.chrom.rand.3,../SACKO.chrom.rand.3,../SALRO.chrom.rand.3,../SCHME.chrom.rand.3,../STRMA.chrom.rand.3,../STRPU.chrom.rand.3,../SYCCI.chrom.rand.3,../TRIAD.chrom.rand.3,../TRICA.chrom.rand.3,../XENTR.chrom.rand.3 5 01_microsynteny/Orthofinder.clus sbatch --array=1-1176 --constraint=array-1core --job-name=synt_of_RAND3 job.sh

CHROM_RAND3=../../ACAPL.chrom.rand.3,../../ACRMI.chrom.rand.3,../../ADIVA.chrom.rand.3,../../AMPQU.chrom.rand.3,../../ANOGA.chrom.rand.3,../../AURAU.chrom.rand.3,../../BRALA.chrom.rand.3,../../CAEEL.chrom.rand.3,../../CALMI.chrom.rand.3,../../CAPOW.chrom.rand.3,../../CAPTE.chrom.rand.3,../../CHEMY.chrom.rand.3,../../CIOIN.chrom.rand.3,../../CLYHE.chrom.rand.3,../../CRAGI.chrom.rand.3,../../DANRE.chrom.rand.3,../../DAPPU.chrom.rand.3,../../DROME.chrom.rand.3,../../EUPSC.chrom.rand.3,../../EXAPA.chrom.rand.3,../../GALGA.chrom.rand.3,../../HELRO

.chrom.rand.3,../../HIPCO.chrom.rand.3,../../HOFMI.chrom.rand.3,../../HOIHO.chrom.rand.3,../../HOMSA.chrom.rand.3,../../HYDVU.chrom.rand.3,../../IXOSC.chrom.rand.3,../../LATCH.chrom.rand.3,../../LEPOC.chrom.rand.3,../../LINAN.chrom.rand.3,../../LOTGI.chrom.rand.3,../../MAYZE.chrom.rand.3,../../MIZYE.chrom.rand.3,../../MNELE.chrom.rand.3,../../MUSMU.chrom.rand.3,../../NEMVE.chrom.rand.3,../../PARTE.chrom.rand.3,../../PLEBA.chrom.rand.3,../../PTYFL.chrom.rand.3,../../SACKO.chrom.rand.3,../../SALRO.chrom.rand.3,../../SCHME.chrom.rand.3,../../STRMA.chrom.rand.3,../../STRPU.chrom.rand.3,../../SYCCI.chrom.rand.3,../../TRIAD.chrom.rand.3,../../TRICA.chrom.rand.3,../../XENTR.chrom.rand.3 mkdir bash_scripts log_files pairwise_blocks; mv *.out log_files; mv *.sh bash_scripts; mv *.blocks pairwise_blocks

cd 01_microsynteny/randomized_chrom/rand.3/Orthofinder/pairwise_blocks makeClusters3.pl $CHROM_RAND3 .5.blocks 3 0.3 0.5 > 5.blocks.3.syn.synt

correct_blocks_coordinates.py 5.blocks.3.syn.synt $CHROM_RAND3 > 5.blocks.3.syn_corrected.synt

```
## 5. Gene density analysis
### 5.1 Data preparation
#### 5.1.1 Download genome files
If possible, sequences have been downloaded as softmasked (repetitive sequences in
lowercase). This is more informative than hardmask (all residues in uppercase,
masked residues replaces by Ns).
```

#From NCBI: 16 genomes curl ftp://ftp.ncbi.nlm.nih.gov/genomes/all/GCF/000/001/405/GCF_000001405.39_GRCh38.p13/GCF_000001405.39_GRCh38.p13_genomic.fna.gz -o HOMSA.masked_genome.gz curl ftp://ftp.ncbi.nlm.nih.gov/genomes/all/GCF/000/001/635/GCF_000001635.26_GRCm38.p6/GCF_000001635.26_GRCm38.p6_genomic.fna.gz -o MUSMU.masked_genome.gz curl ftp://ftp.ncbi.nlm.nih.gov/genomes/all/GCF/000/002/035/GCF_000002035.6_GRCz11/GCF_000002035.6_GRCz11_genomic.fna.gz -o DANRE.masked_genome.gz curl ftp://ftp.ncbi.nlm.nih.gov/genomes/all/GCF/000/002/315/GCF_000002315.6_GRCg6a/GCF_000002315.6_GRCg6a_genomic.fna.gz -o GALGA.masked_genome.gz curl ftp://ftp.ncbi.nlm.nih.gov/genomes/all/GCF/000/004/195/GCF_000004195.3_Xenopus_tropicalis_v9.1/GCF_000004195.3_Xenopus_tropicalis_v9.1_genomic.fna.gz -o XENTR.masked_genome.gz curl ftp://ftp.ncbi.nlm.nih.gov/genomes/all/GCF/000/165/045/GCF_000165045.1_Callorhinchus_milii-6.1.3/GCF_000165045.1_Callorhinchus_milii-6.1.3_genomic.fna.gz -o CALMI.masked_genome.gz curl ftp://ftp.ncbi.nlm.nih.gov/genomes/all/GCF/004/143/615/GCF_004143615.1_amil_sf_1.1/GCF_004143615.1_amil_sf_1.1_genomic.fna.gz -o ACRMI.masked_genome.gz curl ftp://ftp.ncbi.nlm.nih.gov/genomes/all/GCF/000/224/145/GCF_000224145.3_KH/GCF_000224145.3_KH_genomic.fna.gz -o CIOIN.masked_genome.gz curl ftp://ftp.ncbi.nlm.nih.gov/genomes/all/GCF/000/225/785/GCF_000225785.1_LatCha1/GCF_000225785.1_LatCha1_genomic.fna.gz -o LATCH.masked_genome.gz curl ftp://ftp.ncbi.nlm.nih.gov/genomes/all/GCF/000/238/955/GCF_000238955.4_M_zebra_UMD2a/GCF_000238955.4_M_zebra_UMD2a_genomic.fna.gz -o MAYZE.masked_genome.gz curl ftp://ftp.ncbi.nlm.nih.gov/genomes/all/GCF/000/242/695/GCF_000242695.1_LepOcu1/GCF_000242695.1_LepOcu1_genomic.fna.gz -o LEPOC.masked_genome.gz curl ftp://ftp.ncbi.nlm.nih.gov/genomes/all/GCF/000/344/595/GCF_000344595.1_CheMyd_1.0/GCF_000344595.1_CheMyd_1.0_genomic.fna.gz -o CHEMY.masked_genome.gz curl

ftp://ftp.ncbi.nlm.nih.gov/genomes/all/GCF/000/365/465/GCF_000365465.2_Ptep_2.0/GCF_000365465.2_Ptep_2.0_genomic.fna.gz -o PARTE.masked_genome.gz curl ftp://ftp.ncbi.nlm.nih.gov/genomes/all/GCF/001/417/965/GCF_001417965.1_Aiptasia_genome_1.1/GCF_001417965.1_Aiptasia_genome_1.1_genomic.fna.gz -o EXAPA.masked_genome.gz curl ftp://ftp.ncbi.nlm.nih.gov/genomes/all/GCF/001/891/065/GCF_001891065.1_H_comes_QL1_v1/GCF_001891065.1_H_comes_QL1_v1_genomic.fna.gz -o HIPCO.masked_genome.gz curl ftp://ftp.ncbi.nlm.nih.gov/genomes/all/GCF/001/949/145/GCF_001949145.1_OKI-Apl_1.0/GCF_001949145.1_OKI-Apl_1.0_genomic.fna.gz -o ACAPL.masked_genome.gz

#From ENSEMBL: 20 genomes curl ftp://ftp.ensemblgenomes.org/pub/metazoa/release-45/fasta/adineta_vaga/dna/Adineta_vaga.AMS_PRJEB1171_v1.dna_sm.toplevel.fa.gz -o ADIVA.masked_genome.gz curl ftp://ftp.ensemblgenomes.org/pub/metazoa/release-45/fasta/amphimedon_queenslandica/dna/Amphimedon_queenslandica.Aqu1.dna_sm.toplevel.fa.gz -o AMPQU.masked_genome.gz curl ftp://ftp.ensemblgenomes.org/pub/metazoa/release-45/fasta/anopheles_gambiae/dna/Anopheles_gambiae.AgamP4.dna_sm.toplevel.fa.gz -o ANOGA.masked_genome.gz curl ftp://ftp.ensemblgenomes.org/pub/release-45/metazoa/fasta/branchiostoma_lanceolatum/dna/Branchiostoma_lanceolatum.BraLan2.dna_sm.toplevel.fa.gz -o BRALA.masked_genome.gz curl ftp://ftp.ensemblgenomes.org/pub/metazoa/release-45/fasta/caenorhabditis_elegans/dna/Caenorhabditis_elegans.WBcel235.dna_sm.toplevel.fa.gz -o CAEEL.masked_genome.gz curl ftp://ftp.ensemblgenomes.org/pub/metazoa/release-45/fasta/capitella_teleta/dna/Capitella_teleta.Capitella_teleta_v1.0.dna_sm.toplevel.fa.gz -o CAPTE.masked_genome.gz curl ftp://ftp.ensemblgenomes.org/pub/metazoa/release-45/fasta/crassostrea_gigas/dna/Crassostrea_gigas.oyster_v9.dna_sm.toplevel.fa.gz -o CRAGI.masked_genome.gz curl ftp://ftp.ensemblgenomes.org/pub/metazoa/release-45/fasta/daphnia_pulex/dna/Daphnia_pulex.V1.0.dna_sm.toplevel.fa.gz -o DAPPU.masked_genome.gz curl ftp://ftp.ensemblgenomes.org/pub/metazoa/release-45/fasta/drosophila_melanogaster/dna/Drosophila_melanogaster.BDGP6.22.dna_sm.toplevel.fa.gz -o DROME.masked_genome.gz curl ftp://ftp.ensemblgenomes.org/pub/metazoa/release-45/fasta/helobdella_robusta/dna/Helobdella_robusta.Helro1.dna_sm.toplevel.fa.gz -o HELRO.masked_genome.gz curl ftp://ftp.ensemblgenomes.org/pub/metazoa/release-45/fasta/ixodes_scapularis/dna/Ixodes_scapularis.IscaW1.dna_sm.toplevel.fa.gz -o IXOSC.masked_genome.gz curl ftp://ftp.ensemblgenomes.org/pub/metazoa/release-45/fasta/lingula_anatina/dna/Lingula_anatina.LinAna1.0.dna_sm.toplevel.fa.gz -o LINAN.masked_genome.gz curl ftp://ftp.ensemblgenomes.org/pub/metazoa/release-45/fasta/lottia_gigantea/dna/Lottia_gigantea.Lotgi1.dna_sm.toplevel.fa.gz -o LOTGI.masked_genome.gz curl ftp://ftp.ensemblgenomes.org/pub/metazoa/release-45/fasta/nematostella_vectensis/dna/Nematostella_vectensis.ASM20922v1.dna_sm.toplevel.fa.gz -o NEMVE.masked_genome.gz curl ftp://ftp.ensemblgenomes.org/pub/metazoa/release-45/fasta/strigamia_maritima/dna/Strigamia_maritima.Smar1.dna_sm.toplevel.fa.gz -o STRMA.masked_genome.gz curl ftp://ftp.ensemblgenomes.org/pub/metazoa/release-45/fasta/strongylocentrotus_purpuratus/dna/Strongylocentrotus_purpuratus.Spur_3.1.dna_sm.toplevel.fa.gz -o STRPU.masked_genome.gz curl ftp://ftp.ensemblgenomes.org/pub/metazoa/release-45/fasta/tribolium_castaneum/dna/Tribolium_castaneum.Tcas5.2.dna_sm.toplevel.fa.gz -o TRICA.masked_genome.gz curl ftp://ftp.ensemblgenomes.org/pub/metazoa/release-45/fasta/trichoplax_adhaerens/dna/Trichoplax_adhaerens.ASM15027v1.dna_sm.toplevel.fa.gz -o TRIAD.masked_genome.gz curl ftp://ftp.ensemblgenomes.org/pub/protists/release-45/fasta/protists_choanoflagellida1_collection/salpingoeca_rosetta_gca_000188695/dna/Salpingoeca_rosetta_

gca_000188695.Proterospongia_sp_ATCC50818.dna_sm.toplevel.fa.gz -o SALRO.masked_genome.gz curl ftp://ftp.ensemblgenomes.org/pub/protists/release-45/fasta/protists_ichthyosporea1_collection/capsaspora_owczarzaki_atcc_30864_gca_000151315/dna/Capsaspora_owczarzaki_atcc_30864_gca_000151315.C_owczarzaki_V2.dna_sm.toplevel.fa.gz -o CAPOW.masked_genome.gz

#From other sources: 7 genomes curl http://marimba.obs-vlfr.fr/download/file/fid/48 -o CLYHE.hardmasked_genome.gz curl https://research.nhgri.nih.gov/hydra/download/assembly/Hm105_Dovetail_Assembly_1.0.fa.gz -o HYDVU.masked_genome.gz curl https://bitbucket.org/molpalmuc/hoilungia-genome/raw/0d523a5b8556741a37918f3f30d0ed0414833912/sequences/Hhon_final_contigs_softmasked.fasta.gz -o HOIHO.masked_genome.gz curl https://research.nhgri.nih.gov/mnemiopsis/download/genome/MlScaffold09.nt.gz -o MNELE.masked_genome.gz curl https://marinegenomics.oist.jp/acornworm/download/pfl_scaffold_ver1.0.14.masked.fasta.gz -o PTYFL.masked_genome.gz curl http://planmine.mpi-cbg.de/planmine/model/bulkdata/dd_Smes_g4.fasta.zip -o SCHME.masked_genome curl ftp://ftp.ncbi.nlm.nih.gov/genomes/all/GCA/000/695/325/GCA_000695325.1_P.bachei_draft_genome_v.1.1/GCA_000695325.1_P.bachei_draft_genome_v.1.1_genomic.fna.gz -o PLEBA.masked_genome

```

The 3 remaining genomes were downloaded with a browser (and or made using
annotation files) or used from another emplacment in cube:

* Aurelia aurita from:
https://drive.google.com/drive/folders/1NC6bZ9cxWkZyofOsMPzrxIH3C7m1ySiu,
Aurelia.Genome_v1.2_Protein_Models_12-28-18.fasta, renamed AURAU.hardmasked_genome
* Saccoglossus kowalevskii: SkowalevskiiJGIv3.0.longestTrs.pep.fa.gz Downloaded
from Metazome v3 (https://metazome.jgi.doe.gov/pz/portal.html#!bulk?
org=Org_Skowalevskii_er), called SACKO.masked_genome.gz
* Sycon ciliatum genome, CDS and peptide downloaded from datadryad
(https://datadryad.org/resource/doi:10.5061/dryad.tn0f3).


EUPSC.masked_genome genome from Schmidbaur et al. in prep.

Finally, we gunzip all the gz files

```

gunzip *.fa.gz

```
## 5 Gene density analysis (REDUX)
### 5.1. reconstructing ancestral counts of syntenic blocks
We'll use 5 alternating topologies:

* Ctenophore-sister (CS) (cteno,(sponge,(plac,(cnid,(acoel,prot,deut)))));
* Sponge-sister (PS) (sponge,(cteno,(plac,(cnid,(acoel,prot,deut)))));
* Xenacoelomorpha (XNEP): (cteno,sponge,(plac,(cnid,(acoel(prot,deut))))));
```

```
* Xenambulacraria (XAMB): (cteno,sponge,(plac,(cnid,(prot((chord,
(acoel,ambulacraria)))))));
* Consensus (CONS) (cteno,sponge,(plac,(cnid,(acoel,prot,deut))));

Here are the actual trees:

* CONS
```

((((((((((((((HOMSA,MUSMU),(CHEMY,GALGA)),XENTR),LATCH),
(((MAYZE,HIPCO),DANRE),LEPOC)),CALMI)Vertebrata,CIOIN)Olfactores,BRALA)Chordata,((SACKO,PTYFL),
(STRPU,ACAPL))Ambulacraria)Deuterostomia,(((EUPSC,LOTGI,(MIZYE,CRAGI))Mollusca,
(CAPTE,HELRO),ADIVA,LINAN,SCHME)Lophotrochozoa,((((((DROME,ANOGA),TRICA),DAPPU),STRMA),
(IXOSC,PARTE)),CAEEL)Ecdysozoa)Protostomia,HOFMI)Bilateria,(((NEMVE,EXAPA),ACRMI),
((HYDVU,CLYHE),AURAU))Cnidaria)Planulozoa,(HOIHO,TRIAD)Placozoa)Parahoxozoa,
(PLEBA,MNELE)Ctenophora,(SYCCI,AMPQU)Porifera)Metazoa,SALRO)Choanozoa,CAPOW)Filozoa;

```
* CS
```

(((((((((((((((HOMSA,MUSMU),(CHEMY,GALGA)),XENTR),LATCH),
(((MAYZE,HIPCO),DANRE),LEPOC)),CALMI)Vertebrata,CIOIN)Olfactores,BRALA)Chordata,((SACKO,PTYFL),
(STRPU,ACAPL))Ambulacraria)Deuterostomia,(((EUPSC,LOTGI,(MIZYE,CRAGI))Mollusca,
(CAPTE,HELRO),ADIVA,LINAN,SCHME)Lophotrochozoa,((((((DROME,ANOGA),TRICA),DAPPU),STRMA),
(IXOSC,PARTE)),CAEEL)Ecdysozoa)Protostomia,HOFMI)Bilateria,(((NEMVE,EXAPA),ACRMI),
((HYDVU,CLYHE),AURAU))Cnidaria)Planulozoa,(HOIHO,TRIAD)Placozoa)Parahoxozoa,
(SYCCI,AMPQU)Porifera)Porifera_Parahoxozoa,
(PLEBA,MNELE)Ctenophora)Metazoa,SALRO)Choanozoa,CAPOW)Filozoa;

```
* PS
```

(((((((((((((((HOMSA,MUSMU),(CHEMY,GALGA)),XENTR),LATCH),
(((MAYZE,HIPCO),DANRE),LEPOC)),CALMI)Vertebrata,CIOIN)Olfactores,BRALA)Chordata,((SACKO,PTYFL),
(STRPU,ACAPL))Ambulacraria)Deuterostomia,(((EUPSC,LOTGI,(MIZYE,CRAGI))Mollusca,
(CAPTE,HELRO),ADIVA,LINAN,SCHME)Lophotrochozoa,((((((DROME,ANOGA),TRICA),DAPPU),STRMA),
(IXOSC,PARTE)),CAEEL)Ecdysozoa)Protostomia,HOFMI)Bilateria,(((NEMVE,EXAPA),ACRMI),
((HYDVU,CLYHE),AURAU))Cnidaria)Planulozoa,(HOIHO,TRIAD)Placozoa)Parahoxozoa,
(PLEBA,MNELE)Ctenophora)Ctenophora_Parahoxozoa,
(SYCCI,AMPQU)Porifera)Metazoa,SALRO)Choanozoa,CAPOW)Filozoa;

```
* XAMB
```

((((((((((((((HOMSA,MUSMU),(CHEMY,GALGA)),XENTR),LATCH),
(((MAYZE,HIPCO),DANRE),LEPOC)),CALMI)Vertebrata,CIOIN)Olfactores,BRALA)Chordata,(HOFMI,
((SACKO,PTYFL),(STRPU,ACAPL))Ambulacraria)Xenambulacraria)Deuterostomia,(((EUPSC,LOTGI,

(MIZYE,CRAGI))Mollusca,(CAPTE,HELRO),ADIVA,LINAN,SCHME)Lophotrochozoa,((((((DROME,ANOGA),TRICA),DAPPU),STRMA),(IXOSC,PARTE)),CAEEL)Ecdysozoa)Protostomia)Bilateria,(((NEMVE,EXAPA),ACRMI),((HYDVU,CLYHE),AURAU))Cnidaria)Planulozoa,(HOIHO,TRIAD)Placozoa)Parahoxozoa,(PLEBA,MNELE)Ctenophora,(SYCCI,AMPQU)Porifera)Metazoa,SALRO)Choanozoa,CAPOW)Filozoa;

```
* XNEP
```

(((((((((((((((HOMSA,MUSMU),(CHEMY,GALGA)),XENTR),LATCH),(((MAYZE,HIPCO),DANRE),LEPOC)),CALMI)Vertebrata,CIOIN)Olfactores,BRALA)Chordata,((SACKO,PTYFL),(STRPU,ACAPL))Ambulacraria)Deuterostomia,(((EUPSC,LOTGI,(MIZYE,CRAGI))Mollusca,(CAPTE,HELRO),ADIVA,LINAN,SCHME)Lophotrochozoa,((((((DROME,ANOGA),TRICA),DAPPU),STRMA),(IXOSC,PARTE)),CAEEL)Ecdysozoa)Protostomia)Nephrozoa,HOFMI)Bilateria,(((NEMVE,EXAPA),ACRMI),((HYDVU,CLYHE),AURAU))Cnidaria)Planulozoa,(HOIHO,TRIAD)Placozoa)Parahoxozoa,(PLEBA,MNELE)Ctenophora,(SYCCI,AMPQU)Porifera)Metazoa,SALRO)Choanozoa,CAPOW)Filozoa;

```
CONS is the tree we want to use for everything (since  alternate topologies
generate similar results).
CS and PS will be used only for checking that the polytomies in our tree have no
incidence on the estimation of metazoan novelties.
XNEP and XAMB are to be used for checking that the polytomies in our tree don't
affect estimation of novelties in  Bilateria/Nephrozoa (LCA of protostomes and
deuterostomes...) and deuterostomia LCA.
And ofc, the initial multifurcating tree, polytomic at the base of metazoa and at
the base of bilateria

`filt.clusters` is filtered out version of the total multi_species block (minus
cluster 1, 8406 blocks considered as one multi species block, due to fusing
orthologous groups together. Randomized chromosomes don't have such artifacts, no
need to filter them.

* Ctenophore-sister
```

cd 02_gene_density_analysis python3 BlocksByNode.py -c filt.clusters -b ../01_microsynteny/chrom_of/5.blocks.3.syn_corrected.synt -s trees/cteno_sister_bila_poly.tre -n Choanozoa Metazoa Porifera_Parahoxozoa Parahoxozoa Planulozoa Cnidaria Bilateria Deuterostomia Protostomia Chordata Ambulacraria Lophotrochozoa Ecdysozoa Olfactores Mollusca Vertebrata -m 2 -r short -t ancestral novel

#clusters list, to draw Venn diagrams of clusters found in nodes python3 BlocksByNode.py -c filt.clusters -b ../01_microsynteny/chrom_of/5.blocks.3.syn_corrected.synt -s trees/cteno_sister_bila_poly.tre -n Metazoa Porifera_Parahoxozoa -m 2 -r clusters_list -t novel|cut -f1,2 > venn_diag/CS.tsv

#counts for randomized blocks python3 BlocksByNode.py -c ../01_microsynteny/randomized_chrom/rand.1/Orthofinder/5.blocks.3.syn.clusters -b ../01_microsynteny/randomized_chrom/rand.1/Orthofinder/5.blocks.3.syn_corrected.synt -s

trees/cteno_sister_bila_poly.tre -n Choanozoa Metazoa Porifera_Parahoxozoa Parahoxozoa Planulozoa Cnidaria Bilateria Deuterostomia Protostomia Chordata Ambulacraria Lophotrochozoa Ecdysozoa Olfactores Mollusca Vertebrata -m 2 -r short -t ancestral novel python3 BlocksByNode.py -c ../01_microsynteny/randomized_chrom/rand.2/Orthofinder/5.blocks.3.syn.clusters -b ../01_microsynteny/randomized_chrom/rand.2/Orthofinder/5.blocks.3.syn_corrected.synt -s trees/cteno_sister_bila_poly.tre -n Choanozoa Metazoa Porifera_Parahoxozoa Parahoxozoa Planulozoa Cnidaria Bilateria Deuterostomia Protostomia Chordata Ambulacraria Lophotrochozoa Ecdysozoa Olfactores Mollusca Vertebrata -m 2 -r short -t ancestral novel python3 BlocksByNode.py -c ../01_microsynteny/randomized_chrom/rand.3/Orthofinder/5.blocks.3.syn.clusters -b ../01_microsynteny/randomized_chrom/rand.3/Orthofinder/5.blocks.3.syn_corrected.synt -s trees/cteno_sister_bila_poly.tre -n Choanozoa Metazoa Porifera_Parahoxozoa Parahoxozoa Planulozoa Cnidaria Bilateria Deuterostomia Protostomia Chordata Ambulacraria Lophotrochozoa Ecdysozoa Olfactores Mollusca Vertebrata -m 2 -r short -t ancestral novel

```
Ctenophore-sister    | ancestral | novel | recency
---------------------|-----------|-------|--------
Choanozoa            |    17     |   14  |    1
Metazoa              |     0     |    9  |    2
Porifera_Parahoxozoa |     9     |   25  |    3
Parahoxozoa          |    50     |    6  |    4
Planulozoa           |    63     |  162  |    5
Cnidaria             |   254     |   11  |    6
Bilateria            |   335     |  256  |    6
Deuterostomia        |   545     |    3  |    7
Protostomia          |   612     |   16  |    7
Chordata             |   511     |    1  |    8
Ambulacraria         |   430     |    6  |    8
Lophotrochozoa       |   798     |   91  |    8
Ecdysozoa            |   241     |    1  |    8
Olfactores           |   147     |    1  |    9
Mollusca             |   662     |   66  |    9
Vertebrata           |    70     |  170  |   10



* Porifera-sister
```

python3 BlocksByNode.py -c filt.clusters -b ../01_microsynteny/chrom_of/5.blocks.3.syn_corrected.synt -s trees/sponge_sister_bila_poly.tre -n Choanozoa Metazoa Ctenophora_Parahoxozoa Parahoxozoa Planulozoa Cnidaria Bilateria Deuterostomia Protostomia Chordata Ambulacraria Lophotrochozoa Ecdysozoa Olfactores Mollusca Vertebrata -m 2 -r short -t ancestral novel

#clusters list, to draw Venn diagrams of clusters found in nodes python3 BlocksByNode.py -c filt.clusters -b ../01_microsynteny/chrom_of/5.blocks.3.syn_corrected.synt -s trees/sponge_sister_bila_poly.tre -n Metazoa Ctenophora_Parahoxozoa -m 2 -r clusters_list -t novel|cut -f1,2 > venn_diag/PS.tsv

#counts for randomized blocks python3 BlocksByNode.py -c ../01_microsynteny/randomized_chrom/rand.1/Orthofinder/5.blocks.3.syn.clusters -b

../01_microsynteny/randomized_chrom/rand.1/Orthofinder/5.blocks.3.syn_corrected.synt -s trees/sponge_sister_bila_poly.tre -n Choanozoa Metazoa Ctenophora_Parahoxozoa Parahoxozoa Planulozoa Cnidaria Bilateria Deuterostomia Protostomia Chordata Ambulacraria Lophotrochozoa Ecdysozoa Olfactores Mollusca Vertebrata -m 2 -r short -t ancestral novel python3 BlocksByNode.py -c ../01_microsynteny/randomized_chrom/rand.2/Orthofinder/5.blocks.3.syn.clusters -b ../01_microsynteny/randomized_chrom/rand.2/Orthofinder/5.blocks.3.syn_corrected.synt -s trees/sponge_sister_bila_poly.tre -n Choanozoa Metazoa Ctenophora_Parahoxozoa Parahoxozoa Planulozoa Cnidaria Bilateria Deuterostomia Protostomia Chordata Ambulacraria Lophotrochozoa Ecdysozoa Olfactores Mollusca Vertebrata -m 2 -r short -t ancestral novel python3 BlocksByNode.py -c ../01_microsynteny/randomized_chrom/rand.3/Orthofinder/5.blocks.3.syn.clusters -b ../01_microsynteny/randomized_chrom/rand.3/Orthofinder/5.blocks.3.syn_corrected.synt -s trees/sponge_sister_bila_poly.tre -n Choanozoa Metazoa Ctenophora_Parahoxozoa Parahoxozoa Planulozoa Cnidaria Bilateria Deuterostomia Protostomia Chordata Ambulacraria Lophotrochozoa Ecdysozoa Olfactores Mollusca Vertebrata -m 2 -r short -t ancestral novel

```
Porifera-sister        | ancestral | novel | recency
-----------------------|-----------|-------|--------
Choanozoa              |    17     |  14   |    1
Metazoa                |     0     |  25   |    2
Ctenophora_Parahoxozoa |    31     |   7   |    3
Parahoxozoa            |    50     |   6   |    4
Planulozoa             |    63     | 162   |    5
Cnidaria               |   254     |  11   |    6
Bilateria              |   335     | 256   |    6
Deuterostomia          |   545     |   3   |    7
Protostomia            |   612     |  16   |    7
Chordata               |   511     |   1   |    8
Ambulacraria           |   430     |   6   |    8
Lophotrochozoa         |   798     |  91   |    8
Ecdysozoa              |   241     |   1   |    8
Olfactores             |   147     |   1   |    9
Mollusca               |   662     |  66   |    9
Vertebrata             |    70     | 170   |   10



* Xenacoelomorpha
```

python3 BlocksByNode.py -c filt.clusters -b ../01_microsynteny/chrom_of/5.blocks.3.syn_corrected.synt -s trees/meta_poly_xenacoelomorpha.tre -n Choanozoa Metazoa Parahoxozoa Planulozoa Cnidaria Bilateria Nephrozoa Deuterostomia Protostomia Chordata Ambulacraria Lophotrochozoa Ecdysozoa Olfactores Mollusca Vertebrata -m 2 -r short -t ancestral novel

#clusters list, to draw Venn diagrams of clusters found in nodes python3 BlocksByNode.py -c filt.clusters -b ../01_microsynteny/chrom_of/5.blocks.3.syn_corrected.synt -s trees/meta_poly_xenacoelomorpha.tre -n Bilateria Nephrozoa Deuterostomia Ambulacraria -m 2 -r clusters_list -t novel|cut -f1,2 > venn_diag/XNEP.tsv

#count blocks in randomized genomes python3 BlocksByNode.py -c ../01_microsynteny/randomized_chrom/rand.1/Orthofinder/5.blocks.3.syn.clusters -b ../01_microsynteny/randomized_chrom/rand.1/Orthofinder/5.blocks.3.syn_corrected.synt -s trees/meta_poly_xenacoelomorpha.tre -n Choanozoa Metazoa Parahoxozoa Planulozoa Cnidaria Bilateria Nephrozoa Deuterostomia Protostomia Chordata Ambulacraria Lophotrochozoa Ecdysozoa Olfactores Mollusca Vertebrata -m 2 -r short -t ancestral novel python3 BlocksByNode.py -c ../01_microsynteny/randomized_chrom/rand.2/Orthofinder/5.blocks.3.syn.clusters -b ../01_microsynteny/randomized_chrom/rand.2/Orthofinder/5.blocks.3.syn_corrected.synt -s trees/meta_poly_xenacoelomorpha.tre -n Choanozoa Metazoa Parahoxozoa Planulozoa Cnidaria Bilateria Nephrozoa Deuterostomia Protostomia Chordata Ambulacraria Lophotrochozoa Ecdysozoa Olfactores Mollusca Vertebrata -m 2 -r short -t ancestral novel python3 BlocksByNode.py -c ../01_microsynteny/randomized_chrom/rand.3/Orthofinder/5.blocks.3.syn.clusters -b ../01_microsynteny/randomized_chrom/rand.3/Orthofinder/5.blocks.3.syn_corrected.synt -s trees/meta_poly_xenacoelomorpha.tre -n Choanozoa Metazoa Parahoxozoa Planulozoa Cnidaria Bilateria Nephrozoa Deuterostomia Protostomia Chordata Ambulacraria Lophotrochozoa Ecdysozoa Olfactores Mollusca Vertebrata -m 2 -r short -t ancestral novel

```
Xenacoelomorpha        | ancestral | novel | recency
-----------------------|-----------|-------|--------
Choanozoa              |    17     |  14   |    1
Metazoa                |     0     |  34   |    2
Parahoxozoa            |    50     |   6   |    3
Planulozoa             |    60     | 162   |    4
Cnidaria               |   254     |  11   |    5
Bilateria              |   335     |  38   |    5
Nephrozoa              |   354     | 224   |    6
Deuterostomia          |   545     |   3   |    7
Protostomia            |   612     |  16   |    7
Chordata               |   511     |   1   |    8
Ambulacraria           |   430     |   6   |    8
Lophotrochozoa         |   798     |  91   |    8
Ecdysozoa              |   241     |   1   |    8
Olfactores             |   147     |   1   |    9
Mollusca               |   662     |  66   |    9
Vertebrata             |    70     | 170   |   10

* Xenambulacraria
```

python3 BlocksByNode.py -c filt.clusters -b ../01_microsynteny/chrom_of/5.blocks.3.syn_corrected.synt -s trees/meta_poly_xenambulacraria.tre -n Choanozoa Metazoa Parahoxozoa Planulozoa Cnidaria Bilateria Deuterostomia Protostomia Chordata Xenambulacraria Ambulacraria Lophotrochozoa Ecdysozoa Olfactores Mollusca Vertebrata -m 2 -r short -t ancestral novel

#clusters list, to draw Venn diagrams of clusters found in nodes python3 BlocksByNode.py -c filt.clusters -b ../01_microsynteny/chrom_of/5.blocks.3.syn_corrected.synt -s trees/meta_poly_xenambulacraria.tre -n Bilateria Deuterostomia Xenambulacraria Ambulacraria -m 2 -r clusters_list -t novel|cut -f1,2 > venn_diag/XAMB.tsv

#Isolate blocks, and clusters

#random block counts python3 BlocksByNode.py -c ../01_microsynteny/randomized_chrom/rand.1/Orthofinder/5.blocks.3.syn.clusters -b ../01_microsynteny/randomized_chrom/rand.1/Orthofinder/5.blocks.3.syn_corrected.synt -s trees/meta_poly_xenambulacraria.tre -n Choanozoa Metazoa Parahoxozoa Planulozoa Cnidaria Bilateria Deuterostomia Protostomia Chordata Xenambulacraria Ambulacraria Lophotrochozoa Ecdysozoa Olfactores Mollusca Vertebrata -m 2 -r short -t ancestral novel python3 BlocksByNode.py -c ../01_microsynteny/randomized_chrom/rand.2/Orthofinder/5.blocks.3.syn.clusters -b ../01_microsynteny/randomized_chrom/rand.2/Orthofinder/5.blocks.3.syn_corrected.synt -s trees/meta_poly_xenambulacraria.tre -n Choanozoa Metazoa Parahoxozoa Planulozoa Cnidaria Bilateria Deuterostomia Protostomia Chordata Xenambulacraria Ambulacraria Lophotrochozoa Ecdysozoa Olfactores Mollusca Vertebrata -m 2 -r short -t ancestral novel python3 BlocksByNode.py -c ../01_microsynteny/randomized_chrom/rand.3/Orthofinder/5.blocks.3.syn.clusters -b ../01_microsynteny/randomized_chrom/rand.3/Orthofinder/5.blocks.3.syn_corrected.synt -s trees/meta_poly_xenambulacraria.tre -n Choanozoa Metazoa Parahoxozoa Planulozoa Cnidaria Bilateria Deuterostomia Protostomia Chordata Xenambulacraria Ambulacraria Lophotrochozoa Ecdysozoa Olfactores Mollusca Vertebrata -m 2 -r short -t ancestral novel

```
No syntenic novelty retained in xenacoelomorpha

Xenambulacraria        | ancestral | novel | recency
-----------------------|-----------|-------|--------
Choanozoa              |    17     |   14  |    1
Metazoa                |     0     |   34  |    2
Parahoxozoa            |    50     |    6  |    3
Planulozoa             |  63     |  162  |    4
Cnidaria               |  254      |   11  |    5
Bilateria              |  328      |  237  |    5
Deuterostomia          |  568      |    3  |    6
Protostomia            |  612      |   16  |    6
Xenambulacraria        |  511      |    0  |    7
Chordata               |  517      |    1  |    8
Ambulacraria           |  430      |    6  |    8
Lophotrochozoa         |  798      |   91  |    8
Ecdysozoa              |  241      |    1  |    8
Olfactores             |  147      |    1  |    9
Mollusca               |  662      |   66  |    9
Vertebrata             |    70     |  170  |   10


* Consensus
```

python3 BlocksByNode.py -c filt.clusters -b ../01_microsynteny/chrom_of/5.blocks.3.syn_corrected.synt -s trees/meta_poly_bila_poly.tre -n Choanozoa Metazoa Parahoxozoa Planulozoa Cnidaria Bilateria Deuterostomia Protostomia Chordata Ambulacraria Lophotrochozoa Ecdysozoa Olfactores Mollusca Vertebrata -m 2 -r short -t ancestral novel

#clusters list, to draw Venn diagrams of clusters found in nodes python3 BlocksByNode.py -c filt.clusters -b ../01_microsynteny/chrom_of/5.blocks.3.syn_corrected.synt -s trees/meta_poly_bila_poly.tre -n Metazoa Bilateria Deuterostomia Ambulacraria -m 2 -r clusters_list -t novel|cut -f1,2 > venn_diag/CONS.tsv

nodelist=(Bilateria Lophotrochozoa Metazoa Parahoxozoa Planulozoa Vertebrata) #Block types for node in "${nodelist[@]}"; do python3 BlocksByNode.py -c filt.clusters -b ../01_microsynteny/chrom_of/5.blocks.3.syn_corrected.synt -s trees/meta_poly_bila_poly.tre -n $node -m 2 -r blocks_list -t novel|cut -f2- > bynode/CONS/$node.novel.synt python3 BlocksByNode.py -c filt.clusters -b ../01_microsynteny/chrom_of/5.blocks.3.syn_corrected.synt -s trees/meta_poly_bila_poly.tre -n $node -m 2 -r clusters_list -t novel|cut -f1,3- > bynode/CONS/$node.novel.clusters; done

#random block counts python3 BlocksByNode.py -c ../01_microsynteny/randomized_chrom/rand.1/Orthofinder/5.blocks.3.syn.clusters -b ../01_microsynteny/randomized_chrom/rand.1/Orthofinder/5.blocks.3.syn_corrected.synt -s trees/meta_poly_bila_poly.tre -n Choanozoa Metazoa Parahoxozoa Planulozoa Cnidaria Bilateria Deuterostomia Protostomia Chordata Ambulacraria Lophotrochozoa Ecdysozoa Olfactores Mollusca Vertebrata -m 2 -r short -t ancestral novel python3 BlocksByNode.py -c ../01_microsynteny/randomized_chrom/rand.2/Orthofinder/5.blocks.3.syn.clusters -b ../01_microsynteny/randomized_chrom/rand.2/Orthofinder/5.blocks.3.syn_corrected.synt -s trees/meta_poly_bila_poly.tre -n Choanozoa Metazoa Parahoxozoa Planulozoa Cnidaria Bilateria Deuterostomia Protostomia Chordata Ambulacraria Lophotrochozoa Ecdysozoa Olfactores Mollusca Vertebrata -m 2 -r short -t ancestral novel python3 BlocksByNode.py -c ../01_microsynteny/randomized_chrom/rand.3/Orthofinder/5.blocks.3.syn.clusters -b ../01_microsynteny/randomized_chrom/rand.3/Orthofinder/5.blocks.3.syn_corrected.synt -s trees/meta_poly_bila_poly.tre -n Choanozoa Metazoa Parahoxozoa Planulozoa Cnidaria Bilateria Deuterostomia Protostomia Chordata Ambulacraria Lophotrochozoa Ecdysozoa Olfactores Mollusca Vertebrata -m 2 -r short -t ancestral novel

```
Consensus               | ancestral | novel | recency
-----------------------|-----------|-------|--------
Choanozoa              |    17     |   14  |    1
Metazoa                |     0     |   34  |    2
Parahoxozoa            |    50     |    6  |    3
Planulozoa             |    63     |  162  |    4
Cnidaria               |   254     |   11  |    5
Bilateria              |   335     |  256  |    5
Deuterostomia          |   545     |    3  |    6
Protostomia            |   612     |   16  |    6
Chordata               |   511     |    1  |    7
Ambulacraria           |   430     |    6  |    7
Lophotrochozoa         |   798     |   91  |    7
Ecdysozoa              |   241     |    1  |    7
Olfactores             |   147     |    1  |    8
Mollusca               |   662     |   66  |    8
Vertebrata             |    70     |  170  |    9
```

```
### 5.2 Measuring gene density at key nodes
We'll use consensus tree
```

cd 02_gene_density_analysis/bynode/CONS #randomized blocks nodelist=(Bilateria Lophotrochozoa Metazoa Parahoxozoa Planulozoa Vertebrata) for node in "${nodelist[@]}"; do while read species; do echo "Rscript pick_random_blocks.R --only-genome=$species -n 100 random/$node/ $node.novel.synt ../../../01_microsynteny/chrom/$species.chrom"; done < $node.species; done > random.sh

cat random.sh |slurmtasks -n random_blocks |sbatch

for node in "${nodelist[@]}"; do cat random/$node/*tsv|grep -v "block_id" > $node.novel.random.synt ; done

```
Now for measuring gene density in every animal and node, we use
`make_tidy_density_df`.  It can take a while (2-3 hours for the 49 species
dataset, 6 different syntenic nodes, everything runs sequentially on a single core
of an AMD Opteron 6320).
This outputs a huge table with gene density by block, genome size, median
intergenic distance, node, taxon, species, gene list, para/not_para classification
(>40% or less than  40% of genes belonging to the same OG) (i.e. all info needed
to build figures).

Total assembly size is measured by parsing fasta files
```

python3 make_tidy_density_df.py -s ../bynode/CONS/ -g ../genomes/ -c ../../01_microsynteny/chrom/ -m ../../01_microsynteny/chrom_of/5.blocks.3.syn.clusters -og ../../01_microsynteny/Orthofinder.clus

```
Make data to be used for scatterplots of SF4 (run in the same folder as tidy
density df)
```

figure2_prep_data_scatterplots.py

```
We'll use eggnogmapper.
Two steps: first emapper is homology searches with diamond, second is the actual
annotation
```

fastafolder="00_orthology_assignment/proteins_processed/" outfolder="05_GO/emapper"

cd $fastafolder for f in *.fasta; do echo "python /apps/eggnogmapper/2.0.0/emapper.py -m diamond --no_annot --no_file_comments --cpu 1 -i 00_orthology_assignment/proteins_processed/$f -o 05_GO/emapper/$f"; done>$outfolder/homology_searches cd $outfolder

cat homology_searches |slurmtasks -n emapper -m 10 |sbatch

ls |grep emapper.seed_orthologs|cut -f1 -d '.' > species.list while read species; do echo "emapper.py --annotate_hits_table $species.fasta.emapper.seed_orthologs --no_file_comments -o $species --cpu 2"; done<species_list>annotation_job cat annotation_job |slurmtasks -n emapper -m 1 -c 2 -f array-2core |sbatch

```
We want to make ids2go file for  all our species. We'll change the comma-delimited
GO IDs to semi-colon delimited, as this is the format the GOATOOLS API parses.
```

for species in $(cat species.list); do cut -f 1,7 $species.emapper.annotations|grep GO:|sed 's/,/;/g' > ids2GO_files/$species.ids2go; done

```
Isolate lists of proteins for each species
First, we make lists for all the species of all the proteins in each proteome
```

cd 00_orthology_assignment/proteins_processed/ species_list=$(ls *.fasta|cut -f1 -d '.') for species in $species_list; do grep '>' $species.fasta | cut -f 2 -d '>' > 05_GO/emapper/ids2GO_files/$species.list done

```
we won't do parahoxozoa enrichments, since blocks we found are roughly as many as
background ones
```

cd 05_GO/GO_enrichment

annotations="../emapper/annotations" species_list=$(ls $annotations/*emapper.annotations|rev|cut -d '/' -f 1|rev|cut -f1 -d '.') #cut to keep only the last field ids2go_folder=../emapper/ids2GO_files

#MLCA enrichments name=Metazoa syntfile=../../02_REDUX_gene_density_analysis/bynode/CONS/Metazoa.novel.synt for species in $species_list; do grep -P "^\d+\t$species" $syntfile | cut -f10|tr "," "\n"> Metazoa/$species.$name.list; done

#PLCA enrichments name=Planulozoa syntfile=../../02_REDUX_gene_density_analysis/bynode/CONS/Planulozoa.novel.synt for species in $species_list; do grep -P "^\d+\t$species" $syntfile | cut -f10|tr "," "\n"> Planulozoa/$species.$name.list; done

#BLCA enrichments name=Bilateria syntfile=../../02_REDUX_gene_density_analysis/bynode/CONS/Bilateria.novel.synt for species in $species_list; do grep -P "^\d+\t$species" $syntfile | cut -f10|tr "," "\n"> Bilateria/$species.$name.list; done

#remove empty files (no blocks) find / -empty -type f -delete

#get latest version of GO terms (downloaded on the 5th april 2020) wget http://purl.obolibrary.org/obo/go/go-basic.obo

#run GO enrichment for all species posessing MCLA blocks species_list=$(ls Metazoa |cut -f2 -d "/"|cut -f1 -d ".") #only species in given folder for species in $species_list; do ids2go=$ids2go_folder/$species.ids2go background_seq=$ids2go_folder/$species.list sample_seq=Metazoa/$species.Metazoa.list python3

/proj/robert/scripts/GO/GO_analysis.py -i $ids2go -go go-basic.obo -b $background_seq -s $sample_seq; done

#run GO enrichment for all species posessing PCLA blocks species_list=$(ls Planulozoa |cut -f2 -d "/"|cut -f1 -d ".") #only species in given folder for species in $species_list; do ids2go=$ids2go_folder/$species.ids2go background_seq=$ids2go_folder/$species.list sample_seq=Planulozoa/$species.Planulozoa.list python3 /proj/robert/scripts/GO/GO_analysis.py -i $ids2go -go go-basic.obo -b $background_seq -s $sample_seq; done

#run GO enrichment for all species posessing BLCA blocks species_list=$(ls Bilateria |cut -f2 -d "/"|cut -f1 -d ".") #only species in given folder for species in $species_list; do ids2go=$ids2go_folder/$species.ids2go background_seq=$ids2go_folder/$species.list sample_seq=Bilateria/$species.Bilateria.list python3 /proj/robert/scripts/GO/GO_analysis.py -i $ids2go -go go-basic.obo -b $background_seq -s $sample_seq; done

```
check which terms are enriched in which taxons. GO enrichment results are in the
GO_enrichment_results folder
```

cd 05_GO/GO_enrichment enrichment_comparisons.py

```
### 7.1 Download Expression data for CALMI and MUSMU
All other transcript abundances taken from Zieger et al. 2020 (doi:
10.1016/j.cub.2020.10.004)
#### CALMI
We first want to use our filtered gff file (no MT genome). And download the
transcripts
```

08_exp/CALMI/CALMI.gff

wget ftp://ftp.ncbi.nlm.nih.gov/genomes/all/GCF/000/165/045/GCF_000165045.1_Callorhinchus_milii-6.1.3/GCF_000165045.1_Callorhinchus_milii-6.1.3_rna.fna.gz -O CALMI.transcripts.fa.gz

```
Now this is for renaming the transcripts in the mRNA file by the protein names and
filter out transcripts that are not the ones encoding our filtered proteins
```

/proj/robert/scripts/dev_RNAseq/rename_filter_transcripts.py -a CALMI_NCBI.gff -ft mRNA -fp CDS -kpp Parent -kpt ID -kp protein_id -kt transcript_id -o CALMI_protnames.fna -f CALMI.transcripts.fa -c 01_microsynteny/chrom/CALMI.chrom

```
Download all the reads
```

module load sratoolkit fasterq-dump SRR1735385 -e 8 -o thymus fasterq-dump SRR513757 -e 8 -o testis fasterq-dump SRR513758 -e 8 -o spleen fasterq-dump SRR513759 -e 8 -o ovary fasterq-dump SRR513760 -e 8 -o liver fasterq-dump SRR514104 -e 8 -o muscle fasterq-dump SRR514105 -e 8 -o kidney fasterq-dump SRR514106 -e 8 -o intestine fasterq-dump SRR514107 -e 8 -o heart fasterq-dump SRR514109 -e 8 -o brain fasterq-dump SRR534176 -e 8 -o gills

```
Now for the kallisto run
```

module load kallisto #cerate index kallisto index -i CALMI.index CALMI_protnames.fna

reads=08_exp/CALMI/reads

le1="_1.fastq" le2="_2.fastq"

#submit kallisto quantification jobs for file in $(ls reads/|rev|cut -f 2- -d '_'|rev|uniq); do echo "kallisto quant --index=CALMI.index --rf-stranded --output-dir=$file --plaintext reads/$file$le1 reads/$file$le2" ; done | /proj/rpz/slurm_scripts/slurmtasks --mem 5 --name quantCALMI | sbatch

```

```

paste */abundance.tsv | cut -f 1,5,10,15,20,25,30,35,40,45,50,55,60,65,70,75,80,85,90,95,100,105,110,115 > CALMI_transcript_tpms_all_samples.tsv ls -1 */abundance.tsv | perl -ne 'chomp $; if ($ =~ /(\S+)/abundance.tsv/){print "\t$1"}' | perl -ne 'print "target_id$_\n"' > header.tsv cat header.tsv CALMI_transcript_tpms_all_samples.tsv | grep -v "tpm" > CALMI_transcript_tpms_all_samples.tsv2 mv CALMI_transcript_tpms_all_samples.tsv2 CALMI_transcript_tpms_all_samples.tsv rm -f header.tsv

```

## MUSMU
```

python3 /proj/robert/scripts/dev_RNAseq/rename_filter_transcripts.py -a 00_orthology_assignment/coordinates/gff/MUSMU_filt_NCBI.gff3 -ft mRNA -fp CDS -kpp gene -kpt gene -kp protein_id -kt Name -o MUSMU_protnames.fna -f MUSMU.transcripts.fa -c 01_microsynteny/chrom/MUSMU.chrom

```

```

fasterq-dump SRR5273648 -e 4 -o female_bone_marrow_a fasterq-dump SRR5273664 -e 4 -o female_bone_marrow_b

module load sratoolkit prefetch SRR5273654 cd SRR5273654 fastq-dump --split-e --skip-technical SRR5273654.sra mv SRR5273654_1.fastq ../female_adrenal_gland_b_1.fastq mv SRR5273654_2.fastq

../female_adrenal_gland_b_2.fastq

prefetch SRR5273670 cd SRR5273670 fastq-dump --split-e --skip-technical SRR5273670.sra mv SRR5273670_1.fastq ../female_adrenal_gland_a_1.fastq mv SRR5273670_2.fastq ../female_adrenal_gland_a_2.fastq cd ../ rm -r SRR5273670

prefetch SRR5273635 cd SRR5273635 fastq-dump --split-e --skip-technical SRR5273635.sra mv SRR5273635_1.fastq ../female_brain_a_1.fastq mv SRR5273635_2.fastq ../female_brain_a_2.fastq cd ../ rm -r SRR5273635

prefetch SRR5273637 cd SRR5273637 fastq-dump --split-e --skip-technical SRR5273637.sra mv SRR5273637_1.fastq ../female_brain_b_1.fastq mv SRR5273637_2.fastq ../female_brain_b_2.fastq cd ../ rm -r SRR5273637

#todo prefetch SRR5273657 cd SRR5273657 fastq-dump --split-e --skip-technical SRR5273657.sra mv SRR5273657_1.fastq ../female_brain_c_1.fastq mv SRR5273657_2.fastq ../female_brain_c_2.fastq cd ../ rm -r SRR5273657

prefetch SRR5273673 cd SRR5273673 fastq-dump --split-e --skip-technical SRR5273673.sra mv SRR5273673_1.fastq ../female_brain_d_1.fastq mv SRR5273673_2.fastq ../female_brain_d_2.fastq cd ../ rm -r SRR5273673

prefetch SRR5273646 cd SRR5273646 fastq-dump --split-e --skip-technical SRR5273646.sra mv SRR5273646_1.fastq ../female_forestomach_a_1.fastq mv SRR5273646_2.fastq ../female_forestomach_a_2.fastq cd ../ rm -r SRR5273646

prefetch SRR5273662 cd SRR5273662 fastq-dump --split-e --skip-technical SRR5273662.sra mv SRR5273662_1.fastq ../female_forestomach_b_1.fastq mv SRR5273662_2.fastq ../female_forestomach_b_2.fastq cd ../ rm -r SRR5273662

prefetch SRR5273651 cd SRR5273651 fastq-dump --split-e --skip-technical SRR5273651.sra mv SRR5273651_1.fastq ../female_heart_a_1.fastq mv SRR5273651_2.fastq ../female_heart_a_2.fastq cd ../ rm -r SRR5273651

prefetch SRR5273667 cd SRR5273667 fastq-dump --split-e --skip-technical SRR5273667.sra mv SRR5273667_1.fastq ../female_heart_b_1.fastq mv SRR5273667_2.fastq ../female_heart_b_2.fastq cd ../ rm -r SRR5273667

prefetch SRR5273655 cd SRR5273655 fastq-dump --split-e --skip-technical SRR5273655.sra mv SRR5273655_1.fastq ../female_kidney_a_1.fastq mv SRR5273655_2.fastq ../female_kidney_a_2.fastq cd ../ rm -r SRR5273655

prefetch SRR5273671 cd SRR5273671 fastq-dump --split-e --skip-technical SRR5273671.sra mv SRR5273671_1.fastq ../female_kidney_b_1.fastq mv SRR5273671_2.fastq ../female_kidney_b_2.fastq cd ../ rm -r SRR5273671

prefetch SRR5273644 cd SRR5273644 fastq-dump --split-e --skip-technical SRR5273644.sra mv SRR5273644_1.fastq ../female_large_intestine_a_1.fastq mv SRR5273644_2.fastq ../female_large_intestine_a_2.fastq cd ../ rm -r SRR5273644

prefetch SRR5273660 cd SRR5273660 fastq-dump --split-e --skip-technical SRR5273660.sra mv SRR5273660_1.fastq ../female_large_intestine_b_1.fastq mv SRR5273660_2.fastq ../female_large_intestine_b_2.fastq cd ../ rm -r SRR5273660

prefetch SRR5273634 cd SRR5273634 fastq-dump --split-e --skip-technical SRR5273634.sra mv SRR5273634_1.fastq ../female_liver_a_1.fastq mv SRR5273634_2.fastq ../female_liver_a_2.fastq cd ../ rm -r SRR5273634

prefetch SRR5273636 cd SRR5273636 fastq-dump --split-e --skip-technical SRR5273636.sra mv SRR5273636_1.fastq ../female_liver_b_1.fastq mv SRR5273636_2.fastq ../female_liver_b_2.fastq cd ../ rm -r SRR5273636

prefetch SRR5273656 cd SRR5273656 fastq-dump --split-e --skip-technical SRR5273656.sra mv SRR5273656_1.fastq ../female_liver_c_1.fastq mv SRR5273656_2.fastq ../female_liver_c_2.fastq cd ../ rm -r SRR5273656

prefetch SRR5273672 cd SRR5273672 fastq-dump --split-e --skip-technical SRR5273672.sra mv SRR5273672_1.fastq ../female_liver_d_1.fastq mv SRR5273672_2.fastq ../female_liver_d_2.fastq cd ../ rm -r SRR5273672

prefetch SRR5273652 cd SRR5273652 fastq-dump --split-e --skip-technical SRR5273652.sra mv SRR5273652_1.fastq ../female_lung_a_1.fastq mv SRR5273652_2.fastq ../female_lung_a_2.fastq cd ../ rm -r SRR5273652

prefetch SRR5273668 cd SRR5273668 fastq-dump --split-e --skip-technical SRR5273668.sra mv SRR5273668_1.fastq ../female_lung_b_1.fastq mv SRR5273668_2.fastq ../female_lung_b_2.fastq cd ../ rm -r SRR5273668

prefetch SRR5273643 cd SRR5273643 fastq-dump --split-e --skip-technical SRR5273643.sra mv SRR5273643_1.fastq ../female_muscle_a_1.fastq mv SRR5273643_2.fastq ../female_muscle_a_2.fastq cd ../ rm -r SRR5273643

prefetch SRR5273659 cd SRR5273659 fastq-dump --split-e --skip-technical SRR5273659.sra mv SRR5273659_1.fastq ../female_muscle_b_1.fastq mv SRR5273659_2.fastq ../female_muscle_b_2.fastq cd ../ rm -r SRR5273659

prefetch SRR5273649 cd SRR5273649 fastq-dump --split-e --skip-technical SRR5273649.sra mv SRR5273649_1.fastq ../female_ovary_a_1.fastq mv SRR5273649_2.fastq ../female_ovary_a_2.fastq cd ../ rm -r SRR5273649

prefetch SRR5273665 cd SRR5273665 fastq-dump --split-e --skip-technical SRR5273665.sra mv SRR5273665_1.fastq ../female_ovary_b_1.fastq mv SRR5273665_2.fastq ../female_ovary_b_2.fastq cd ../ rm -r SRR5273665

prefetch SRR5273645 cd SRR5273645 fastq-dump --split-e --skip-technical SRR5273645.sra mv SRR5273645_1.fastq ../female_small_intestine_a_1.fastq mv SRR5273645_2.fastq ../female_small_intestine_a_2.fastq cd ../ rm -r SRR5273645

prefetch SRR5273661 cd SRR5273661 fastq-dump --split-e --skip-technical SRR5273661.sra mv SRR5273661_1.fastq ../female_small_intestine_b_1.fastq mv SRR5273661_2.fastq ../female_small_intestine_b_2.fastq cd ../ rm -r SRR5273661

prefetch SRR5273653 cd SRR5273653 fastq-dump --split-e --skip-technical SRR5273653.sra mv SRR5273653_1.fastq ../female_spleen_a_1.fastq mv SRR5273653_2.fastq ../female_spleen_a_2.fastq cd ../ rm -r SRR5273653

prefetch SRR5273669 cd SRR5273669 fastq-dump --split-e --skip-technical SRR5273669.sra mv SRR5273669_1.fastq ../female_spleen_b_1.fastq mv SRR5273669_2.fastq ../female_spleen_b_2.fastq cd ../ rm -r SRR5273669

prefetch SRR5273647 cd SRR5273647 fastq-dump --split-e --skip-technical SRR5273647.sra mv SRR5273647_1.fastq ../female_stomach_a_1.fastq mv SRR5273647_2.fastq ../female_stomach_a_2.fastq cd ../ rm -r SRR5273647

prefetch SRR5273663 cd SRR5273663 fastq-dump --split-e --skip-technical SRR5273663.sra mv SRR5273663_1.fastq ../female_stomach_b_1.fastq mv SRR5273663_2.fastq ../female_stomach_b_2.fastq cd ../ rm -r SRR5273663

prefetch SRR5273650 cd SRR5273650 fastq-dump --split-e --skip-technical SRR5273650.sra mv SRR5273650_1.fastq ../female_thymus_a_1.fastq mv SRR5273650_2.fastq ../female_thymus_a_2.fastq cd ../ rm -r SRR5273650

prefetch SRR5273666 cd SRR5273666 fastq-dump --split-e --skip-technical SRR5273666.sra mv SRR5273666_1.fastq ../female_thymus_b_1.fastq mv SRR5273666_2.fastq ../female_thymus_b_2.fastq cd ../ rm -r SRR5273666

prefetch SRR5273642 cd SRR5273642 fastq-dump --split-e --skip-technical SRR5273642.sra mv SRR5273642_1.fastq ../female_uterus_1.fastq mv SRR5273642_2.fastq ../female_uterus_2.fastq cd ../ rm -r SRR5273642

prefetch SRR5273658 cd SRR5273658 fastq-dump --split-e --skip-technical SRR5273658.sra mv SRR5273658_1.fastq ../female_vesicular_gland_1.fastq mv SRR5273658_2.fastq ../female_vesicular_gland_2.fastq cd ../ rm -r SRR5273658

prefetch SRR5273686 cd SRR5273686 fastq-dump --split-e --skip-technical SRR5273686.sra mv SRR5273686_1.fastq ../male_adrenal_gland_a_1.fastq mv SRR5273686_2.fastq ../male_adrenal_gland_a_2.fastq cd ../ rm -r SRR5273686

prefetch SRR5273702 cd SRR5273702 fastq-dump --split-e --skip-technical SRR5273702.sra mv SRR5273702_1.fastq ../male_adrenal_gland_b_1.fastq mv SRR5273702_2.fastq ../male_adrenal_gland_b_2.fastq cd ../ rm -r SRR5273702

prefetch SRR5273680 cd SRR5273680 fastq-dump --split-e --skip-technical SRR5273680.sra mv SRR5273680_1.fastq ../male_bone_marrow_a_1.fastq mv SRR5273680_2.fastq ../male_bone_marrow_a_2.fastq cd ../ rm -r SRR5273680

prefetch SRR5273696 cd SRR5273696 fastq-dump --split-e --skip-technical SRR5273696.sra mv SRR5273696_1.fastq ../male_bone_marrow_b_1.fastq mv SRR5273696_2.fastq ../male_bone_marrow_b_2.fastq cd ../ rm -r SRR5273696

prefetch SRR5273639 cd SRR5273639 fastq-dump --split-e --skip-technical SRR5273639.sra mv SRR5273639_1.fastq ../male_brain_a_1.fastq mv SRR5273639_2.fastq ../male_brain_a_2.fastq cd ../ rm -r SRR5273639

prefetch SRR5273641 cd SRR5273641 fastq-dump --split-e --skip-technical SRR5273641.sra mv SRR5273641_1.fastq ../male_brain_b_1.fastq mv SRR5273641_2.fastq ../male_brain_b_2.fastq cd ../ rm -r SRR5273641

prefetch SRR5273689 cd SRR5273689 fastq-dump --split-e --skip-technical SRR5273689.sra mv SRR5273689_1.fastq ../male_brain_c_1.fastq mv SRR5273689_2.fastq ../male_brain_c_2.fastq cd ../ rm -r SRR5273689

prefetch SRR5273705 cd SRR5273705 fastq-dump --split-e --skip-technical SRR5273705.sra mv SRR5273705_1.fastq ../male_brain_d_1.fastq mv SRR5273705_2.fastq ../male_brain_d_2.fastq cd ../ rm -r SRR5273705

prefetch SRR5273678 cd SRR5273678 fastq-dump --split-e --skip-technical SRR5273678.sra mv SRR5273678_1.fastq ../male_forestomach_a_1.fastq mv SRR5273678_2.fastq ../male_forestomach_a_2.fastq cd ../ rm -r SRR5273678

prefetch SRR5273694 cd SRR5273694 fastq-dump --split-e --skip-technical SRR5273694.sra mv SRR5273694_1.fastq ../male_forestomach_b_1.fastq mv SRR5273694_2.fastq ../male_forestomach_b_2.fastq cd ../ rm -r SRR5273694

prefetch SRR5273683 cd SRR5273683 fastq-dump --split-e --skip-technical SRR5273683.sra mv SRR5273683_1.fastq ../male_heart_a_1.fastq mv SRR5273683_2.fastq ../male_heart_a_2.fastq cd ../ rm -r SRR5273683

prefetch SRR5273699 cd SRR5273699 fastq-dump --split-e --skip-technical SRR5273699.sra mv SRR5273699_1.fastq ../male_heart_b_1.fastq mv SRR5273699_2.fastq ../male_heart_b_2.fastq cd ../ rm -r SRR5273699

prefetch SRR5273687 cd SRR5273687 fastq-dump --split-e --skip-technical SRR5273687.sra mv SRR5273687_1.fastq ../male_kidney_a_1.fastq mv SRR5273687_2.fastq ../male_kidney_a_2.fastq cd ../ rm -r SRR5273687

prefetch SRR5273703 cd SRR5273703 fastq-dump --split-e --skip-technical SRR5273703.sra mv SRR5273703_1.fastq ../male_kidney_b_1.fastq mv SRR5273703_2.fastq ../male_kidney_b_2.fastq cd ../ rm -r SRR5273703

prefetch SRR5273676 cd SRR5273676 fastq-dump --split-e --skip-technical SRR5273676.sra mv SRR5273676_1.fastq ../male_large_intestine_a_1.fastq mv SRR5273676_2.fastq ../male_large_intestine_a_2.fastq cd ../ rm -r SRR5273676

prefetch SRR5273692 cd SRR5273692 fastq-dump --split-e --skip-technical SRR5273692.sra mv SRR5273692_1.fastq ../male_large_intestine_b_1.fastq mv SRR5273692_2.fastq ../male_large_intestine_b_2.fastq cd ../ rm -r SRR5273692

prefetch SRR5273638 cd SRR5273638 fastq-dump --split-e --skip-technical SRR5273638.sra mv SRR5273638_1.fastq ../male_liver_a_1.fastq mv SRR5273638_2.fastq ../male_liver_a_2.fastq cd ../ rm -r SRR5273638

prefetch SRR5273640 cd SRR5273640 fastq-dump --split-e --skip-technical SRR5273640.sra mv SRR5273640_1.fastq ../male_liver_b_1.fastq mv SRR5273640_2.fastq ../male_liver_b_2.fastq cd ../ rm -r SRR5273640

prefetch SRR5273688 cd SRR5273688 fastq-dump --split-e --skip-technical SRR5273688.sra mv SRR5273688_1.fastq ../male_liver_c_1.fastq mv SRR5273688_2.fastq ../male_liver_c_2.fastq cd ../ rm -r SRR5273688

prefetch SRR5273704 cd SRR5273704 fastq-dump --split-e --skip-technical SRR5273704.sra mv SRR5273704_1.fastq ../male_liver_d_1.fastq mv SRR5273704_2.fastq ../male_liver_d_2.fastq cd ../ rm -r SRR5273704

prefetch SRR5273684 cd SRR5273684 fastq-dump --split-e --skip-technical SRR5273684.sra mv SRR5273684_1.fastq ../male_lung_a_1.fastq mv SRR5273684_2.fastq ../male_lung_a_2.fastq cd ../ rm -r SRR5273684

prefetch SRR5273700 cd SRR5273700 fastq-dump --split-e --skip-technical SRR5273700.sra mv SRR5273700_1.fastq ../male_lung_b_1.fastq mv SRR5273700_2.fastq ../male_lung_b_2.fastq cd ../ rm -r SRR5273700

prefetch SRR5273675 cd SRR5273675 fastq-dump --split-e --skip-technical SRR5273675.sra mv SRR5273675_1.fastq ../male_muscle_a_1.fastq mv SRR5273675_2.fastq ../male_muscle_a_2.fastq cd ../ rm -r SRR5273675

prefetch SRR5273691 cd SRR5273691 fastq-dump --split-e --skip-technical SRR5273691.sra mv SRR5273691_1.fastq ../male_muscle_b_1.fastq mv SRR5273691_2.fastq ../male_muscle_b_2.fastq cd ../ rm -r SRR5273691

prefetch SRR5273677 cd SRR5273677 fastq-dump --split-e --skip-technical SRR5273677.sra mv SRR5273677_1.fastq ../male_small_intestine_a_1.fastq mv SRR5273677_2.fastq ../male_small_intestine_a_2.fastq cd ../ rm -r SRR5273677

prefetch SRR5273693 cd SRR5273693 fastq-dump --split-e --skip-technical SRR5273693.sra mv SRR5273693_1.fastq ../male_small_intestine_b_1.fastq mv SRR5273693_2.fastq ../male_small_intestine_b_2.fastq cd ../ rm -r SRR5273693

prefetch SRR5273685 cd SRR5273685 fastq-dump --split-e --skip-technical SRR5273685.sra mv SRR5273685_1.fastq ../male_spleen_a_1.fastq mv SRR5273685_2.fastq ../male_spleen_a_2.fastq cd ../ rm -r SRR5273685

prefetch SRR5273701 cd SRR5273701 fastq-dump --split-e --skip-technical SRR5273701.sra mv SRR5273701_1.fastq ../male_spleen_b_1.fastq mv SRR5273701_2.fastq ../male_spleen_b_2.fastq cd ../ rm -r SRR5273701

prefetch SRR5273679 cd SRR5273679 fastq-dump --split-e --skip-technical SRR5273679.sra mv SRR5273679_1.fastq ../male_stomach_a_1.fastq mv SRR5273679_2.fastq ../male_stomach_a_2.fastq cd ../ rm -r SRR5273679

prefetch SRR5273695 cd SRR5273695 fastq-dump --split-e --skip-technical SRR5273695.sra mv SRR5273695_1.fastq ../male_stomach_b_1.fastq mv SRR5273695_2.fastq ../male_stomach_b_2.fastq cd ../ rm -r SRR5273695

prefetch SRR5273681 cd SRR5273681 fastq-dump --split-e --skip-technical SRR5273681.sra mv SRR5273681_1.fastq ../male_testis_a_1.fastq mv SRR5273681_2.fastq ../male_testis_a_2.fastq cd ../ rm -r SRR5273681

prefetch SRR5273697 cd SRR5273697 fastq-dump --split-e --skip-technical SRR5273697.sra mv SRR5273697_1.fastq ../male_testis_b_1.fastq mv SRR5273697_2.fastq ../male_testis_b_2.fastq cd ../ rm -r SRR5273697

prefetch SRR5273682 cd SRR5273682 fastq-dump --split-e --skip-technical SRR5273682.sra mv SRR5273682_1.fastq ../male_thymus_a_1.fastq mv SRR5273682_2.fastq ../male_thymus_a_2.fastq cd ../ rm -r SRR5273682

prefetch SRR5273698 cd SRR5273698 fastq-dump --split-e --skip-technical SRR5273698.sra mv SRR5273698_1.fastq ../male_thymus_b_1.fastq mv SRR5273698_2.fastq ../male_thymus_b_2.fastq cd ../ rm -r SRR5273698

prefetch SRR5273674 cd SRR5273674 fastq-dump --split-e --skip-technical SRR5273674.sra mv SRR5273674_1.fastq ../male_vesicular_gland_a_1.fastq mv SRR5273674_2.fastq ../male_vesicular_gland_a_2.fastq cd ../ rm -r SRR5273674

prefetch SRR5273690 cd SRR5273690 fastq-dump --split-e --skip-technical SRR5273690.sra mv SRR5273690_1.fastq ../male_vesicular_gland_b_1.fastq mv SRR5273690_2.fastq ../male_vesicular_gland_b_2.fastq cd ../ rm -r SRR5273690

```
kallisto run
```

module load kallisto #cerate index kallisto index -i MUSMU.index MUSMU_protnames.fna

reads=08_exp/MUSMU/reads

le1="_1.fastq" le2="_2.fastq"

#submit kallisto quantification jobs for file in $(ls reads/|rev|cut -f 2- -d '_'|rev|uniq); do echo "kallisto quant --index=MUSMU.index --rf-stranded --output-dir=$file --plaintext reads/$file$le1 reads/$file$le2" ; done | /proj/rpz/slurm_scripts/slurmtasks --mem 5 --name quantMUSMU | sbatch

paste */abundance.tsv | cut -f 1,5,10,15,20,25,30,35,40,45,50,55,60,65,70,75,80,85,90,95,100,105,110,115,120,125,130,135,140,145,150,155,160,165,170,175,180,185,190,195,200,205,210,215,220,225,230,235,240,245,250,255,260,265,270,275,280,285,290,295,300,305,310,315,320,325,330,335,340,345,350,355,360,365,370,375,380,385,390 > MUSMU_transcript_tpms_all_samples.tsv ls -1 */abundance.tsv | perl -ne 'chomp $; if ($ =~ /(\S+)/abundance.tsv/){print "\t$1"}' | perl -ne 'print "target_id$_\n"' > header.tsv cat header.tsv MUSMU_transcript_tpms_all_samples.tsv | grep -v "tpm" > MUSMU_transcript_tpms_all_samples.tsv2 mv MUSMU_transcript_tpms_all_samples.tsv2 MUSMU_transcript_tpms_all_samples.tsv rm -f header.tsv

python3 /proj/robert/scripts/Utilities/make_tpm_stage_medians.py "female_adrenal_gland female_bone_marrow female_brain female_forestomach female_heart female_kidney female_large_intestine female_liver female_lung female_muscle female_ovary female_small_intestine female_spleen female_stomach female_thymus female_uterus female_vesicular_gland male_adrenal_gland male_bone_marrow male_brain male_forestomach male_heart male_kidney male_large_intestine male_liver male_lung male_muscle male_small_intestine male_spleen male_stomach male_testis male_thymus male_vesicular_gland" -i MUSMU_transcript_tpms_all_samples.tsv

```
## Calculate the actual block correlations
Really large blocks in vertebrates. since it involve a bit of combinations, it can take a while, especially with the long gnathostome blocks.

Elephant shark
```

python3 /proj/robert/scripts/density/block_correlation_analysis.py -t 02_gene_density_analysis/density_whole_genome/key_nodes.tidydf.csv -s 01_microsynteny/chrom_of/5.blocks.3.syn_corrected.synt -e CALMI/CALMI_transcript_tpms_all_samples.tsv -p CALMI -o CALMI_tidy_corr.csv

```
Scallop
```

python3 /proj/robert/scripts/density/block_correlation_analysis.py -t 02_gene_density_analysis/density_whole_genome/key_nodes.tidydf.csv -s 01_microsynteny/chrom_of/5.blocks.3.syn_corrected.synt -e MIZYE/MIZYE_transcript_tpms_all_samples_medians.tsv -p MIZYE -o MIZYE_tidy_corr.csv

```
Mouse
```

python3 /proj/robert/scripts/density/block_correlation_analysis.py -t 02_gene_density_analysis/density_whole_genome/key_nodes.tidydf.csv -s 01_microsynteny/chrom_of/5.blocks.3.syn_corrected.synt -e MUSMU/MUSMU_transcript_tpms_all_samples_medians.tsv -p MUSMU -o MUSMU_tidy_corr.csv

```
Oyster
```

python3 /proj/robert/scripts/density/block_correlation_analysis.py -t 02_gene_density_analysis/density_whole_genome/key_nodes.tidydf.csv -s 01_microsynteny/chrom_of/5.blocks.3.syn_corrected.synt -e CRAGI/CRAGI_transcript_tpms_all_samples.tsv -p CRAGI -o CRAGI_tidy_corr.csv

```
Urchin
```

python3 /proj/robert/scripts/density/block_correlation_analysis.py -t 02_gene_density_analysis/density_whole_genome/key_nodes.tidydf.csv -s 01_microsynteny/chrom_of/5.blocks.3.syn_corrected.synt -e STRPU/STRPU_transcript_tpms_all_samples.tsv -p STRPU -o STRPU_tidy_corr.csv

```
Hemichordate
```

python3 /proj/robert/scripts/density/block_correlation_analysis.py -t 02_gene_density_analysis/density_whole_genome/key_nodes.tidydf.csv -s 01_microsynteny/chrom_of/5.blocks.3.syn_corrected.synt -e SACKO/SACKO_transcript_tpms_all_samples.tsv -p SACKO -o SACKO_tidy_corr.csv

```
## 8. Particular cases manual annotation
### 8.1 HOX
#### 8.1.1 HOX annotation
References and animals for which *hox* sequences used as queries for reciprocal
BLAST searches in the planulozoan sequences of our sample:

* Amemiya et al. 2013 (doi: doi.org/10.1038/nature12027); LATCH

* Anaya et al. 2013 (doi:10.1186/1471-213X-13-26); HOMSA, MUSMU, DANRE, CALMI,
CHEMY, CIOIN, BRALA, STRPU, SACKO, PTYFL

* Belcaid et al. 2018 (doi: 10.1073/pnas.1817322116); EUPSC

* Brauchle et al. 2018 (doi: doi.org/10.1093/gbe/evy170); HOFMI

* Currie et al. 2016 (doi: 10.1186/s13227-016-0044-8); SCHME

* DuBuc et al. 2012 (doi: 10.1093/icb/ics098); NEMVE

* Hench et al. 2015 (doi: 10.1371/journal.pone.0126947); CAEEL

* Kuraku and Meyer 2009 (doi: 10.1387/ijdb.072533km); DANRE, HOMSA, GALGA, XENTR,
CALMI

* Leclère et al. 2019 (doi: 10.1038/s41559-019-0833-2); CLYHE

* Leite et al. 2018 (doi: 10.1093/molbev/msy125); PARTE

* Pace et al. 2016 (doi: 10.1186/s13227-016-0048-4); ANOGA, DROME, TRICA, DAPPU,
STRMA, IXOSC

* Simakov et al. 2013 (doi: 10.1038/nature11696); CAPTE, LOTGI, DAPPU

* Simakov et al. 2015 (doi: 10.1038/nature16150); PTYFL, SACKO

* Wang et al. 2017 (doi: 10.1038/s41559-017-0120); MIZYE

Orthologs of hoxes in the 49 species of our sample were identified by reciprocal
BLASTs using as queries aforementionned sequences.
Annotation was given according to their top hit in the NR database.

#### 8.1.2 Hox microsynteny and gene density analysis
`HOX.clus` Is a cluster file constituted of only one OG (accessions of all
```

```
annoatted hoxes in http://synteny.csb.univie.ac.at/, planulozoan novelty, `hox
manually curated`).
```

cd 01_microsynteny/chrom prepMicroSynt.pl ACAPL.chrom,ACRMI.chrom,ADIVA.chrom,AMPQU.chrom,ANOGA.chrom,AURAU.chrom,BRALA.chrom,CAEEL.chrom,CALMI.chrom,CAPOW.chrom,CAPTE.chrom,CHEMY.chrom,CIOIN.chrom,CLYHE.chrom,CRAGI.chrom,DANRE.chrom,DAPPU.chrom,DROME.chrom,EUPSC.chrom,EXAPA.chrom,GALGA.chrom,HELRO.chrom,HIPCO.chrom,HOFMI.chrom,HOIHO.chrom,HOMSA.chrom,HYDVU.chrom,IXOSC.chrom,LATCH.chrom,LEPOC.chrom,LINAN.chrom,LOTGI.chrom,MAYZE.chrom,MIZYE.chrom,MNELE.chrom,MUSMU.chrom,NEMVE.chrom,PARTE.chrom,PLEBA.chrom,PTYFL.chrom,SACKO.chrom,SALRO.chrom,SCHME.chrom,STRMA.chrom,STRPU.chrom,SYCCI.chrom,TRIAD.chrom,TRICA.chrom,XENTR.chrom 5 03_HOX/HOX_density/HOX.clus sbatch --array=1-1176 --constraint=array-1core --job-name=hox_synt job.sh

chromfile_folder='01_microsynteny/chrom' makeClusters3.pl $chromfile_folder/ACAPL.chrom,$chromfile_folder/ACRMI.chrom,$chromfile_folder/ADIVA.chrom,$chromfile_folder/AMPQU.chrom,$chromfile_folder/ANOGA.chrom,$chromfile_folder/AURAU.chrom,$chromfile_folder/BRALA.chrom,$chromfile_folder/CAEEL.chrom,$chromfile_folder/CALMI.chrom,$chromfile_folder/CAPOW.chrom,$chromfile_folder/CAPTE.chrom,$chromfile_folder/CHEMY.chrom,$chromfile_folder/CIOIN.chrom,$chromfile_folder/CLYHE.chrom,$chromfile_folder/CRAGI.chrom,$chromfile_folder/DANRE.chrom,$chromfile_folder/DAPPU.chrom,$chromfile_folder/DROME.chrom,$chromfile_folder/EUPSC.chrom,$chromfile_folder/EXAPA.chrom,$chromfile_folder/GALGA.chrom,$chromfile_folder/HELRO.chrom,$chromfile_folder/HIPCO.chrom,$chromfile_folder/HOFMI.chrom,$chromfile_folder/HOIHO.chrom,$chromfile_folder/HOMSA.chrom,$chromfile_folder/HYDVU.chrom,$chromfile_folder/IXOSC.chrom,$chromfile_folder/LATCH.chrom,$chromfile_folder/LEPOC.chrom,$chromfile_folder/LINAN.chrom,$chromfile_folder/LOTGI.chrom,$chromfile_folder/MAYZE.chrom,$chromfile_folder/MIZYE.chrom,$chromfile_folder/MNELE.chrom,$chromfile_folder/MUSMU.chrom,$chromfile_folder/NEMVE.chrom,$chromfile_folder/PARTE.chrom,$chromfile_folder/PLEBA.chrom,$chromfile_folder/PTYFL.chrom,$chromfile_folder/SACKO.chrom,$chromfile_folder/SALRO.chrom,$chromfile_folder/SCHME.chrom,$chromfile_folder/STRMA.chrom,$chromfile_folder/STRPU.chrom,$chromfile_folder/SYCCI.chrom,$chromfile_folder/TRIAD.chrom,$chromfile_folder/TRICA.chrom,$chromfile_folder/XENTR.chrom .5.blocks 3 0.3 0.5 03_HOX/HOX_density/5.blocks.3.syn.synt

correct_blocks_coordinates.py 03_HOX/HOX_density/5.blocks.3.syn.synt ACAPL.chrom,ACRMI.chrom,ADIVA.chrom,AMPQU.chrom,ANOGA.chrom,AURAU.chrom,BRALA.chrom,CAEEL.chrom,CALMI.chrom,CAPOW.chrom,CAPTE.chrom,CHEMY.chrom,CIOIN.chrom,CLYHE.chrom,CRAGI.chrom,DANRE.chrom,DAPPU.chrom,DROME.chrom,EUPSC.chrom,EXAPA.chrom,GALGA.chrom,HELRO.chrom,HIPCO.chrom,HOFMI.chrom,HOIHO.chrom,HOMSA.chrom,HYDVU.chrom,IXOSC.chrom,LATCH.chrom,LEPOC.chrom,LINAN.chrom,LOTGI.chrom,MAYZE.chrom,MIZYE.chrom,MNELE.chrom,MUSMU.chrom,NEMVE.chrom,PARTE.chrom,PLEBA.chrom,PTYFL.chrom,SACKO.chrom,SALRO.chrom,SCHME.chrom,STRMA.chrom,STRPU.chrom,SYCCI.chrom,TRIAD.chrom,TRICA.chrom,XENTR.chrom > 03_HOX/HOX_density/5.blocks.3.syn_corrected.synt

```
Make the randomized HOX blocks.
```

cd 03_HOX/HOX_density/rand_blocks cut -f2 ../5.blocks.3.syn_corrected.synt|sort -u > genomes.list n=100 outfolder='03_HOX/HOX_density/rand_blocks' chromfile_folder='01_microsynteny/chrom'

inputfile='../5.blocks.3.syn_corrected.synt'

while read species; do echo "pick_random_blocks.R --only-genome=$species -n $n $outfolder $inputfile $chromfile_folder/$species.chrom"; done < genomes.list > random_hoxblocks

slurmtasks -m 10 -n random_hoxblocks random_hoxblocks | sbatch cat *.tsv | grep -v block_id > Hoxblocks.100r.syntx #merge all the tsvs together

cd $chromfile_folder correct_blocks_coordinates.py 03_HOX/HOX_density/rand_blocks/Hoxblocks.100r.syntx ACAPL.chrom,ACRMI.chrom,ADIVA.chrom,AMPQU.chrom,ANOGA.chrom,AURAU.chrom,BRALA.chrom,CAEEL.chrom,CALMI.chrom,CAPOW.chrom,CAPTE.chrom,CHEMY.chrom,CIOIN.chrom,CLYHE.chrom,CRAGI.chrom,DANRE.chrom,DAPPU.chrom,DROME.chrom,EUPSC.chrom,EXAPA.chrom,GALGA.chrom,HELRO.chrom,HIPCO.chrom,HOFMI.chrom,HOIHO.chrom,HOMSA.chrom,HYDVU.chrom,IXOSC.chrom,LATCH.chrom,LEPOC.chrom,LINAN.chrom,LOTGI.chrom,MAYZE.chrom,MIZYE.chrom,MNELE.chrom,MUSMU.chrom,NEMVE.chrom,PARTE.chrom,PLEBA.chrom,PTYFL.chrom,SACKO.chrom,SALRO.chrom,SCHME.chrom,STRMA.chrom,STRPU.chrom,SYCCI.chrom,TRIAD.chrom,TRICA.chrom,XENTR.chrom > 03_HOX/HOX_density/rand_blocks/Hoxblocks.100r.synt

```

```

python3 make_tidy_density_df.py -s ../../03_REDUX_density_HOX_WNT/hox -g ../../02_REDUX_gene_density_analysis/genomes/ -c ../../01_microsynteny/chrom/ -m ../../03_HOX/hox_graph_data/hox_planu.clus -og ../..//03_HOX/hox_graph_data/hox_planu.clus -o hox

```


### 8.2 WNT
#### 8.2.1 Characterization of wnt5-wnt7 cluster
The cluster detected by the microsynteny pipeline is cluster 953, planulozoan
novelty (http://synteny.csb.univie.ac.at/).

References for evidence of synteny of members of the wnt5-wnt7 cluster:

* Cho et al. 2011 (doi: 10.1093/molbev/msq052)

* Janssen et al. 2010 (doi: 10.1186/1471-2148-10-374)

* Sullivan et al. 2007 (doi: 10.1007/s00427-007-0136-5)

* Irimia et al. 2012 (doi: 10.1101/gr.139725.112)

* Kapasa et al. 2010 (doi: 10.1186/1745-6150-5-49)

* Garriock et al. 2012 (doi: 10.1002/dvdy.21156)

Known syntenies by reference

* *wnt5* and *wnt7* in Planulozoans: (Cho et al. 2011), (Janssen et al. 2010),
(Sullivan et al. 2007)

```

```
* *wnt5* and *fbxl14* in Planulozoans (Irimia et al. 2012), and bilaterians
(Kapasa et al. 2010)

* *wnt7* and *atxn10* in Planulozoans (Irimia et al. 2012) in Vertebrates
(Garriock et al. 2012)

* *wnt5* and *erc1/2* in Olfactores (Kapasa at al. 2010)

* *wnt5* and *cacna2d* in Vertebrates (Kapasa et al. 2010, Garriock et al. 2012)

* *cacna1d*, *ninj1/2* and *dcp1*, this study


Orthologs to members of the *wnt5-wnt7* cluster (*wnt5-wnt7* pair, *fbxl14*,
*atxn10*, *erc1/2*, *cacna1d*, *cacna2d* *ninj1/ninj2* and *dcp1a/dcp1b*) in the
planulozoans of the 49 species of our sample were identified by reciprocal BLAST
using as inital queries human sequences.
#### 8.2.2 wnt5-wnt7 microsynteny and gene density analysis

Similarmy to what we did with hoxes, we build an orthology file where all members
of the cluster are in a single OG (*wnt5-wnt7* pair, *fbxl14*, *atxn10*, *erc1/2*,
*cacna1d*, *cacna2d* *ninj1/ninj2* and *dcp1a/dcp1b*), the file is
`wnt5_wnt7.clus`
```

cd 01_microsynteny/chrom perl /proj/Simakov/scripts/MICROSYNT/prepMicroSynt.pl ACAPL.chrom,ACRMI.chrom,ADIVA.chrom,ANOGA.chrom,AURAU.chrom,BRALA.chrom,CAEEL.chrom,CALMI.chrom,CAPTE.chrom,CHEMY.chrom,CIOIN.chrom,CLYHE.chrom,CRAGI.chrom,DANRE.chrom,DAPPU.chrom,DROME.chrom,EUPSC.chrom,EXAPA.chrom,GALGA.chrom,HELRO.chrom,HIPCO.chrom,HOFMI.chrom,HOMSA.chrom,HYDVU.chrom,IXOSC.chrom,LATCH.chrom,LEPOC.chrom,LINAN.chrom,LOTGI.chrom,MAYZE.chrom,MIZYE.chrom,MUSMU.chrom,NEMVE.chrom,PARTE.chrom,PTYFL.chrom,SACKO.chrom,SCHME.chrom,STRMA.chrom,STRPU.chrom,TRICA.chrom,XENTR.chrom 5 07_wnt5_wnt7/wnt5_wnt7.clus sbatch --array=1-820 --constraint=array-1core --job-name=wnt5_wnt7 job.sh

chromfile_folder='01_microsynteny/chrom' perl /proj/Simakov/scripts/MICROSYNT/makeClusters3.pl $chromfile_folder/ACAPL.chrom,$chromfile_folder/ACRMI.chrom,$chromfile_folder/ADIVA.chrom,$chromfile_folder/AMPQU.chrom,$chromfile_folder/ANOGA.chrom,$chromfile_folder/AURAU.chrom,$chromfile_folder/BRALA.chrom,$chromfile_folder/CAEEL.chrom,$chromfile_folder/CALMI.chrom,$chromfile_folder/CAPOW.chrom,$chromfile_folder/CAPTE.chrom,$chromfile_folder/CHEMY.chrom,$chromfile_folder/CIOIN.chrom,$chromfile_folder/CLYHE.chrom,$chromfile_folder/CRAGI.chrom,$chromfile_folder/DANRE.chrom,$chromfile_folder/DAPPU.chrom,$chromfile_folder/DROME.chrom,$chromfile_folder/EUPSC.chrom,$chromfile_folder/EXAPA.chrom,$chromfile_folder/GALGA.chrom,$chromfile_folder/HELRO.chrom,$chromfile_folder/HIPCO.chrom,$chromfile_folder/HOFMI.chrom,$chromfile_folder/HOIHO.chrom,$chromfile_folder/HOMSA.chrom,$chromfile_folder/HYDVU.chrom,$chromfile_folder/IXOSC.chrom,$chromfile_folder/LATCH.chrom,$chromfile_folder/LEPOC.chrom,$chromfile_folder/LINAN.chrom,$chromfile_folder/LOTGI.chrom,$chromfile_folder/MAYZE.chrom,$chromfile_folder/MIZYE.chrom,$chromfile_folder/MNELE.chrom,$chromfile_folder/MUSMU.chrom,$chromfile_folder/NEMVE.chrom,$chromfile_folder/PARTE.chrom,$chromfile_folder/PLEBA.chrom,$chromfile_folder/PTYFL.chrom,$chromfile_folder/SACKO.chrom,$chromfile_folder/SALRO.chrom,$chromfile_folder/SCHME.chrom,$chromfile_folder/STRMA.chrom,$chromfile_folder/STRPU.chrom,$chromfile_folder/SYCCI.chrom,$chromfile_folder/TRIAD.chrom,$

chromfile_folder/TRICA.chrom,$chromfile_folder/XENTR.chrom .5.blocks 3 0.3 0.5 > 07_wnt5_wnt7/wnt5_wnt7_density/pairwise_blocks/5.blocks.3.syn.synt

cd $chromfile_folder correct_blocks_coordinates.py 07_wnt5_wnt7/wnt5_wnt7_density/5.blocks.3.syn.synt ACAPL.chrom,ACRMI.chrom,ADIVA.chrom,AMPQU.chrom,ANOGA.chrom,AURAU.chrom,BRALA.chrom,CAEEL.chrom,CALMI.chrom,CAPOW.chrom,CAPTE.chrom,CHEMY.chrom,CIOIN.chrom,CLYHE.chrom,CRAGI.chrom,DANRE.chrom,DAPPU.chrom,DROME.chrom,EUPSC.chrom,EXAPA.chrom,GALGA.chrom,HELRO.chrom,HIPCO.chrom,HOFMI.chrom,HOIHO.chrom,HOMSA.chrom,HYDVU.chrom,IXOSC.chrom,LATCH.chrom,LEPOC.chrom,LINAN.chrom,LOTGI.chrom,MAYZE.chrom,MIZYE.chrom,MNELE.chrom,MUSMU.chrom,NEMVE.chrom,PARTE.chrom,PLEBA.chrom,PTYFL.chrom,SACKO.chrom,SALRO.chrom,SCHME.chrom,STRMA.chrom,STRPU.chrom,SYCCI.chrom,TRIAD.chrom,TRICA.chrom,XENTR.chrom > 07_wnt5_wnt7/wnt5_wnt7_density/5.blocks.3.syn_corrected.synt

cd 07_wnt5_wnt7/wnt5_wnt7_density/rand_blocks

cut -f2 ../5.blocks.3.syn_corrected.synt|sort -u > genomes.list n=100 outfolder='07_wnt5_wnt7/wnt5_wnt7_density/rand_blocks' chromfile_folder='01_microsynteny/chrom' inputfile='../5.blocks.3.syn_corrected.synt'

while read species; do echo "Rscript /proj/robert/scripts/microsynteny/pick_random_blocks.R --only-genome=$species -n $n $outfolder $inputfile $chromfile_folder/$species.chrom"; done < genomes.list > wnt5_wnt7

nohup bash wnt5_wnt7&

cat *.tsv | grep -v block_id > wnt5_wnt7.100r.syntx #merge all the tsvs together

cd $chromfile_folder correct_blocks_coordinates.py 07_wnt5_wnt7/wnt5_wnt7_density/rand_blocks/wnt5_wnt7.100r.syntx ACAPL.chrom,ACRMI.chrom,ADIVA.chrom,AMPQU.chrom,ANOGA.chrom,AURAU.chrom,BRALA.chrom,CAEEL.chrom,CALMI.chrom,CAPOW.chrom,CAPTE.chrom,CHEMY.chrom,CIOIN.chrom,CLYHE.chrom,CRAGI.chrom,DANRE.chrom,DAPPU.chrom,DROME.chrom,EUPSC.chrom,EXAPA.chrom,GALGA.chrom,HELRO.chrom,HIPCO.chrom,HOFMI.chrom,HOIHO.chrom,HOMSA.chrom,HYDVU.chrom,IXOSC.chrom,LATCH.chrom,LEPOC.chrom,LINAN.chrom,LOTGI.chrom,MAYZE.chrom,MIZYE.chrom,MNELE.chrom,MUSMU.chrom,NEMVE.chrom,PARTE.chrom,PLEBA.chrom,PTYFL.chrom,SACKO.chrom,SALRO.chrom,SCHME.chrom,STRMA.chrom,STRPU.chrom,SYCCI.chrom,TRIAD.chrom,TRICA.chrom,XENTR.chrom > 07_wnt5_wnt7/wnt5_wnt7_density/rand_blocks/wnt5_wnt7.100r.synt

python3 make_tidy_density_df.py -s ../../03_HOX_WNT/wnt -g ../../02_REDUX_gene_density_analysis/genomes/ -c ../../01_microsynteny/chrom/ -m ../../07_wnt5_wnt7/NeuroWnt_density_manual_redone/manual_final.clusters -og /scratch/robert/2019_microsynteny_size_constraints/07_wnt5_wnt7/NeuroWnt_density_manual_redone/wnt5_7_graph_data/wnt5_wnt7.clus -o wnt

```


Regarding blocks, some of them a lineage specific expansions of CACNA1 or CACNA2D
subunits. We filter the blocks for keeping only the ones with *wnt5* or *wnt7*## 9
Graph representation of blocks
## 2. File preparation
get assembly length of the genomes. In the normali (different from lengths than
the used for normalizing)
```

```
```

for i in $(ls 02_gene_density_analysis/genomes/*genome); do get_length_genome.py -n 0; done

```
Convert the blocks to edgelists (graphs where OGs are the nodes, and edges are the distances of syntenic orthogroups.
We also make edgelists for manually curated hox and wnt genes.
```

Block_to_OGcommus.py -c 02_gene_density_analysis/gene_density/second_sampling/Metazoa_total/Metazoa.m2.total.clusters -b 02_gene_density_analysis/gene_density/second_sampling/Metazoa_total/Metazoa.m2.total.synt -g ACAPL.chrom ACRMI.chrom ADIVA.chrom AMPQU.chrom ANOGA.chrom AURAU.chrom BRALA.chrom CAEEL.chrom CALMI.chrom CAPOW.chrom CAPTE.chrom CIOIN.chrom CLYHE.chrom CRAGI.chrom DAPPU.chrom DROME.chrom EUPSC.chrom EXAPA.chrom HELRO.chrom HOFMI.chrom HOIHO.chrom HYDVU.chrom IXOSC.chrom LATCH.chrom LINAN.chrom LOTGI.chrom MIZYE.chrom MNELE.chrom NEMVE.chrom PARTE.chrom PLEBA.chrom PTYFL.chrom SACKO.chrom SCHME.chrom STRMA.chrom STRPU.chrom SYCCI.chrom TRIAD.chrom TRICA.chrom XENTR.chrom -og 01_microsynteny/Orthofinder.clus -o 10_OG_graphs_representation/Metazoa_total -r 02_gene_density_analysis/gene_density/second_sampling/Metazoa_total/rand_blocks/Metazoa.m2.total.100r.synt

Block_to_OGcommus.py -c 02_gene_density_analysis/gene_density/second_sampling/Planu_placo/Planu_Placo.m2.novel.clusters -b 02_gene_density_analysis/gene_density/second_sampling/Planu_placo/Planu_Placo.m2.novel.synt -g ACRMI.chrom ADIVA.chrom ANOGA.chrom BRALA.chrom CAPTE.chrom CIOIN.chrom CRAGI.chrom EUPSC.chrom EXAPA.chrom HOIHO.chrom HYDVU.chrom LINAN.chrom LOTGI.chrom MIZYE.chrom PARTE.chrom PTYFL.chrom SACKO.chrom SCHME.chrom STRPU.chrom TRIAD.chrom TRICA.chrom -og 01_microsynteny/Orthofinder.clus -o 10_OG_graphs_representation/Planu_Placo_novel -r 02_gene_density_analysis/gene_density/second_sampling/Planu_placo/rand_blocks/Planu_Placo.m2.novel.100r.synt

Block_to_OGcommus.py -c 02_gene_density_analysis/gene_density/second_sampling/Bilateria/Bilateria.m2.novel.clusters -b 02_gene_density_analysis/gene_density/second_sampling/Bilateria/Bilateria.m2.novel.synt -g ACAPL.chrom ADIVA.chrom ANOGA.chrom BRALA.chrom CAEEL.chrom CALMI.chrom CAPTE.chrom CHEMY.chrom CIOIN.chrom CRAGI.chrom DANRE.chrom DAPPU.chrom DROME.chrom EUPSC.chrom GALGA.chrom HELRO.chrom HIPCO.chrom HOFMI.chrom HOMSA.chrom IXOSC.chrom LATCH.chrom LEPOC.chrom LINAN.chrom LOTGI.chrom MAYZE.chrom MIZYE.chrom MUSMU.chrom PARTE.chrom PTYFL.chrom SACKO.chrom SCHME.chrom STRMA.chrom STRPU.chrom TRICA.chrom XENTR.chrom -og 01_microsynteny/Orthofinder.clus -o 10_OG_graphs_representation/Bilateria_novel -r 02_gene_density_analysis/gene_density/second_sampling/Bilateria/rand_blocks/Bilateria.m2.novel.100r.synt

Block_to_OGcommus.py -c 02_gene_density_analysis/gene_density/second_sampling/Planulozoa/Planulozoa.m2.novel.clusters -b 02_gene_density_analysis/gene_density/second_sampling/Planulozoa/Planulozoa.m2.novel.synt -g ACAPL.chrom ACRMI.chrom ADIVA.chrom ANOGA.chrom AURAU.chrom BRALA.chrom CAEEL.chrom CALMI.chrom CAPTE.chrom CHEMY.chrom CIOIN.chrom CLYHE.chrom CRAGI.chrom DANRE.chrom

DAPPU.chrom DROME.chrom EUPSC.chrom EXAPA.chrom GALGA.chrom HELRO.chrom HIPCO.chrom HOFMI.chrom HOMSA.chrom HYDVU.chrom IXOSC.chrom LATCH.chrom LEPOC.chrom LINAN.chrom LOTGI.chrom MAYZE.chrom MIZYE.chrom MUSMU.chrom NEMVE.chrom PARTE.chrom PTYFL.chrom SACKO.chrom SCHME.chrom STRMA.chrom STRPU.chrom TRICA.chrom XENTR.chrom -og 01_microsynteny/Orthofinder.clus -o 10_OG_graphs_representation/Planulozoa_novel -r 02_gene_density_analysis/gene_density/second_sampling/Planulozoa/rand_blocks/Planulozoa.m2.novel.100r.synt

Block_to_OGcommus.py -c 02_gene_density_analysis/gene_density/second_sampling/recent_blocks/Lophotrochozoa/Lophotrochozoa_novel.clusters -b 02_gene_density_analysis/gene_density/second_sampling/recent_blocks/Lophotrochozoa/Lophotrochozoa_novel.synt -g ADIVA.chrom CAPTE.chrom CRAGI.chrom EUPSC.chrom HELRO.chrom LINAN.chrom LOTGI.chrom MIZYE.chrom SCHME.chrom -og 01_microsynteny/Orthofinder.clus -o 10_OG_graphs_representation/Lophotrochozoa_novel -r 02_gene_density_analysis/gene_density/second_sampling/recent_blocks/Lophotrochozoa/rand_blocks/Lophotrochozoa_novel.100r.synt

Block_to_OGcommus.py -c 02_gene_density_analysis/gene_density/second_sampling/recent_blocks/Vertebrata/Vertebrata_novel.clusters -b 02_gene_density_analysis/gene_density/second_sampling/recent_blocks/Vertebrata/Vertebrata_novel.synt -g CALMI.chrom CHEMY.chrom DANRE.chrom GALGA.chrom HIPCO.chrom HOMSA.chrom LATCH.chrom LEPOC.chrom MAYZE.chrom MUSMU.chrom XENTR.chrom -og 01_microsynteny/Orthofinder.clus -o 10_OG_graphs_representation/Vertebrata_novel -r 02_gene_density_analysis/gene_density/second_sampling/recent_blocks/Vertebrata/rand_blocks/Vertebrata_novel.100r.synt

```


We use the lengths obtained with `get_length_genome.py` to normalise the basepairs
distances in the OG pairs edgelists.



WNT graph
We'll use our annotation. There is 8 OGs that are annotated as being part of the
syntenic block. We'll have 8 OGs in the
07_wnt5_wnt7/wnt5_7_graph_data/wnt5_wnt7.clus.

ATXN10, CACNA1, CACNA2,DCP1, ERC, FBXL14, NINJ, WNT
```

cd 01_microsynteny/chrom Block_to_OGcommus_2.py -c 07_wnt5_wnt7/manual_final.clusters -b 07_wnt5_wnt7/manual_final.synt -g ACAPL.chrom ACRMI.chrom ANOGA.chrom BRALA.chrom CALMI.chrom CHEMY.chrom DANRE.chrom DAPPU.chrom EUPSC.chrom EXAPA.chrom GALGA.chrom HIPCO.chrom HOMSA.chrom IXOSC.chrom LATCH.chrom LEPOC.chrom LINAN.chrom LOTGI.chrom MAYZE.chrom MUSMU.chrom NEMVE.chrom PARTE.chrom SACKO.chrom TRICA.chrom XENTR.chrom -og 07_wnt5_wnt7/wnt5_7_graph_data/wnt5_wnt7.clus -o 07_wnt5_wnt7/wnt5_7_graph_data/wnt5_wnt7 -r 07_wnt5_wnt7/rand_blocks/manual_final.100r.synt --custom_orthology

```
HOX graph
We'll use our annotation `03_HOX/hox_graph_data/hox_planu.clus`, where hoxes are
grouped by taxonomic groups (Lophotrochozoan hox OGs, Vertebrate hox Ogs, etc.)
specific.
```

cd 01_microsynteny/chrom Block_to_OGcommus_2.py -c 03_HOX/HOX_density/5.blocks.3.syn.clusters -b 03_HOX/HOX_density/5.blocks.3.syn_corrected.synt -g ACAPL.chrom ACRMI.chrom ANOGA.chrom BRALA.chrom CALMI.chrom CAPTE.chrom CHEMY.chrom CIOIN.chrom CRAGI.chrom DAPPU.chrom DROME.chrom EUPSC.chrom EXAPA.chrom GALGA.chrom HELRO.chrom HIPCO.chrom HOMSA.chrom IXOSC.chrom LATCH.chrom LEPOC.chrom LINAN.chrom LOTGI.chrom MAYZE.chrom MIZYE.chrom MUSMU.chrom NEMVE.chrom PARTE.chrom PTYFL.chrom SACKO.chrom STRMA.chrom STRPU.chrom TRICA.chrom XENTR.chrom -og 03_HOX/hox_graph_data/hox_planu.clus -o 03_HOX/hox_graph_data/hox -r 03_HOX/HOX_density/rand_blocks/Hoxblocks.100r.synt --custom_orthology

```
```

Figure 1 and supplements

NSMR

```r
library(readr)
library(ggplot2)
library(ggrepel)
library(ggpubr)
library(pheatmap)
library(gridExtra)
library(RColorBrewer)
```

Now we'll load up the data, change iteration/phylo_hypothesis to factor type data

```r
df <- readr::read_delim("node_counts.tsv", delim = "\t")
```

```
## Parsed with column specification:
## cols(
##   node_name = col_character(),
##   node_short = col_character(),
##   block_type = col_character(),
##   count = col_double(),
##   phylo_hypothesis = col_character(),
##   recency = col_double(),
##   observed = col_character(),
##   iteration = col_double()
## )
```

```r
df$iteration <- as.factor(df$iteration)
df$block_type <- as.factor(df$block_type)
df$node_name <- as.character(df$node_name)
df$recency <- as.integer(df$recency)
df$phylo_hypothesis <- as.factor(df$phylo_hypothesis)

df_obs <- dplyr::filter(df, observed == 'obs')
df_rand <- dplyr::filter(df, observed == 'rand')
```

observed blocks counts as a function of recency

```r
obs_scatter <- ggplot(df_obs, aes(x= recency, y = count, ymax = 950, label = node_short)) +
                geom_point(position = position_jitter(width = 0.2), size = 1) +
                ggrepel::geom_text_repel(size = 2,
                                         segment.size  = 0.2,
                                         segment.color = "grey50") +
                ggpubr::stat_cor(p.accuracy = 0.001,
                                 r.accuracy = 0.01,
                                 method = 'spearman',
                                 label.x = 1,
                                 label.y = 925,
                                 color = 'black',
```

```
                               size = 2)


obs_scatter + facet_grid(phylo_hypothesis ~ block_type) + theme_bw()
```

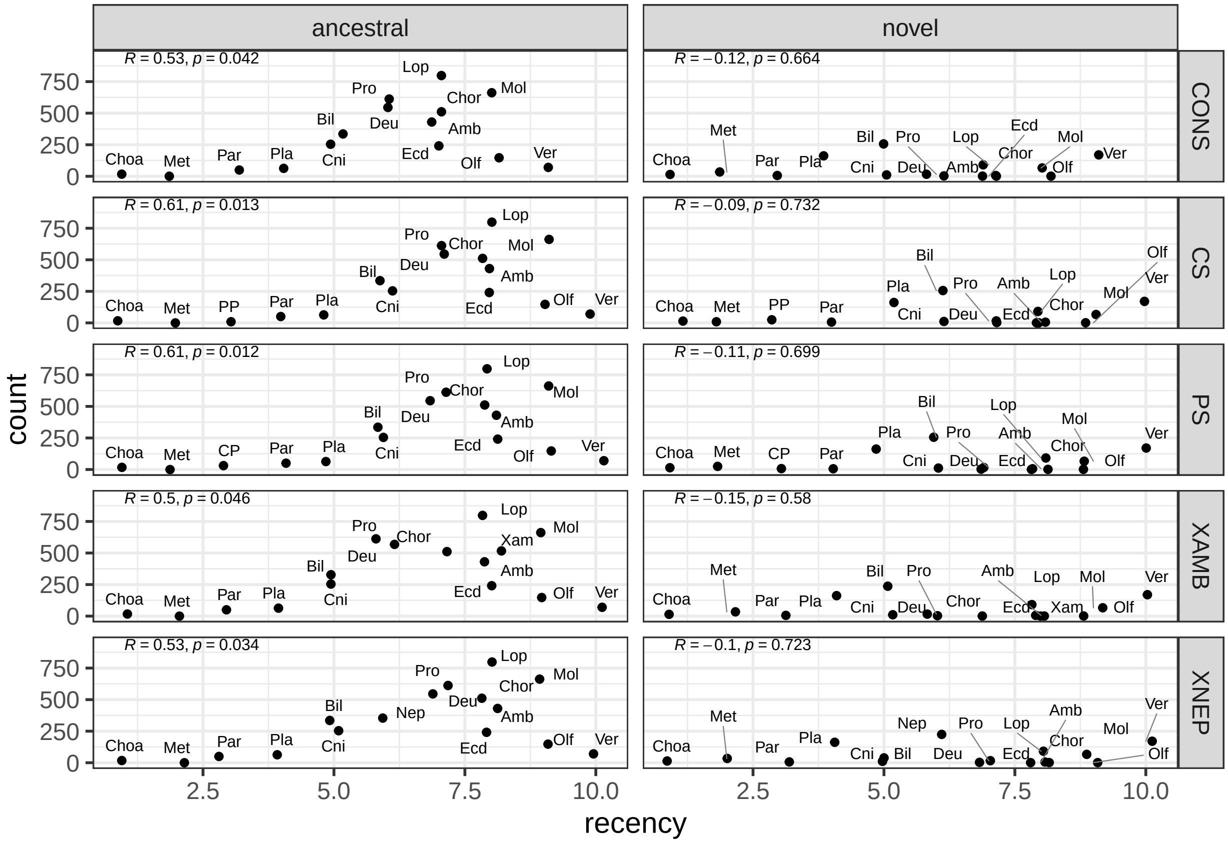


```
ggsave(filename = 'SF1A_obs_block_counts_recency.pdf',
       units = 'cm',
       width = 9,
       height = 16)
```

random blocks counts as a function of recency (supplementary figure)

```
rand_scatter <- ggplot(df_rand, aes(x= recency, y = count, ymax = 200, color = iteration, label = node_s
                  geom_point(position = position_jitter(width = 0.2), size = 1) +
                  ggrepel::geom_text_repel(size = 2,
                                           segment.size  = 0.2) +
                  guides(color = FALSE) + #remove legend, iteration nb is not important
                  ggpubr::stat_cor(p.accuracy = 0.001,
                                   r.accuracy = 0.01,
                                   method = 'spearman',
                                   size = 2)


rand_scatter + facet_grid(phylo_hypothesis ~ block_type) + theme_bw()
```

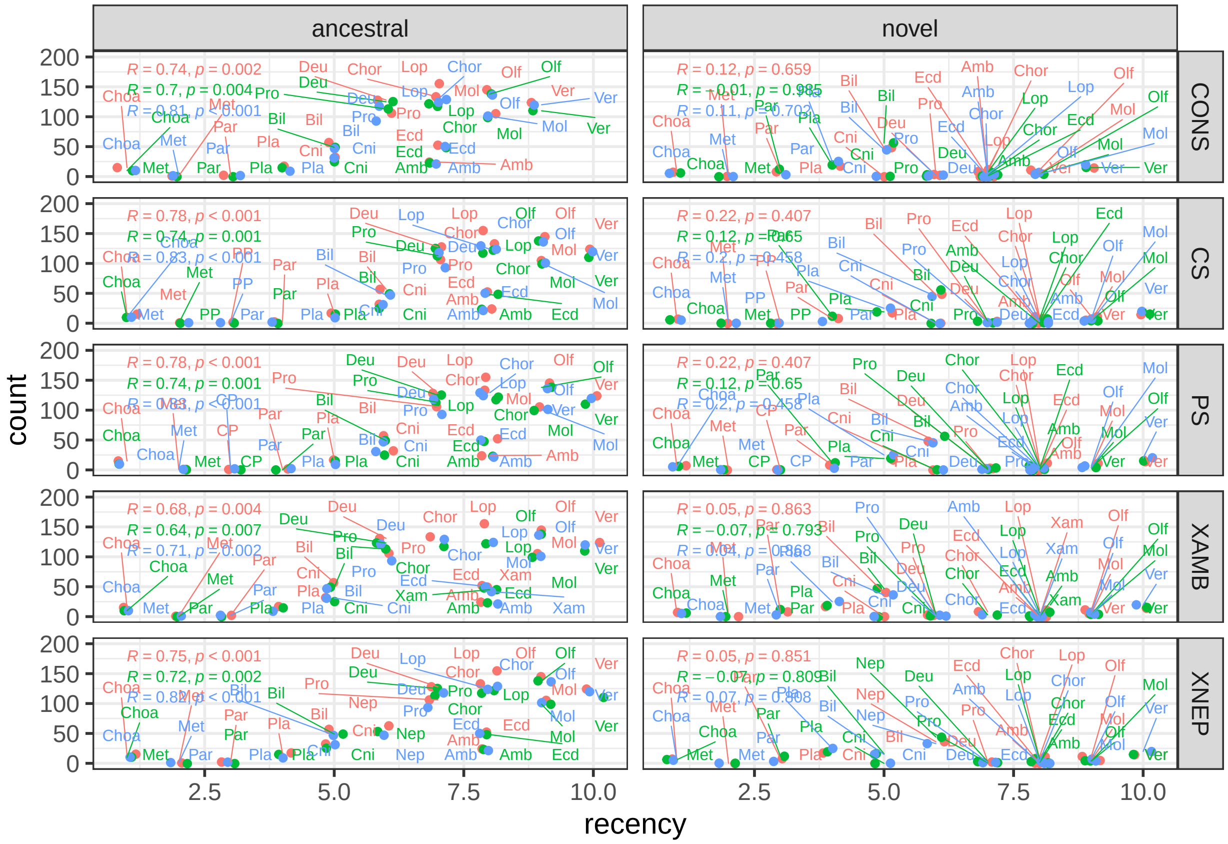


```r
ggsave(filename = 'SF1B_rand_block_counts_recency.pdf',
       units = 'cm',
       width = 21,
       height = 29.7)
```

retention matrix used for the heatmap in figure 1 We use the scatterplot density data used in figure 2

```r
df <- readr::read_csv('raw_data_scatter.csv')
```

```
## Parsed with column specification:
## cols(
##   multi_sp = col_double(),
##   node = col_character(),
##   Vertebrate = col_double(),
##   Tunicate = col_double(),
##   Cephalochordate = col_double(),
##   Ambulacrarian = col_double(),
##   Lophotrochozoan = col_double(),
##   Ecdysozoan = col_double(),
##   Acoel = col_double(),
##   Cnidarian = col_double(),
##   Placozoan = col_double(),
##   Ctenophore = col_double(),
##   Poriferan = col_double()
## )
```

```r
node_names <- c('Bilateria', 'Planulozoa','Parahoxozoa', 'Metazoa')
meta_names <- c("Poriferan", "Ctenophore", "Placozoan", "Cnidarian", "Acoel", "Ecdysozoan", "Lophotroch
```

```r
ancestral_retention_matrix <- matrix(nrow = length(node_names), ncol = length(meta_names))
colnames(ancestral_retention_matrix) <- meta_names
rownames(ancestral_retention_matrix) <- node_names

for (mytaxon in meta_names){
  for (mynode in node_names){
    node_df <- df %>% dplyr::filter(node == mynode)
    df_retained_blocks <- node_df[mytaxon] %>% na.omit()
    retention_percent <- dplyr::tally(df_retained_blocks)$n / dplyr::tally(node_df)$n
    ancestral_retention_matrix[mynode, mytaxon] <- retention_percent
  }
}


ancestral_retention_matrix['Parahoxozoa', c('Poriferan', 'Ctenophore')] <- NA
ancestral_retention_matrix['Planulozoa', c('Poriferan', 'Ctenophore', 'Placozoan')] <- NA
ancestral_retention_matrix['Bilateria', c('Poriferan', 'Ctenophore', 'Placozoan', 'Cnidarian')] <- NA
```

Now for plotting the actual heatmap

retention heatmap that goes in figure 1

```r
bk2 <- c(seq(0, 1,  length=100))

plot <-pheatmap::pheatmap(ancestral_retention_matrix,
                          cluster_rows = F,
                          cluster_cols = F,
                          breaks = bk2,
                          fontsize = 8)
```

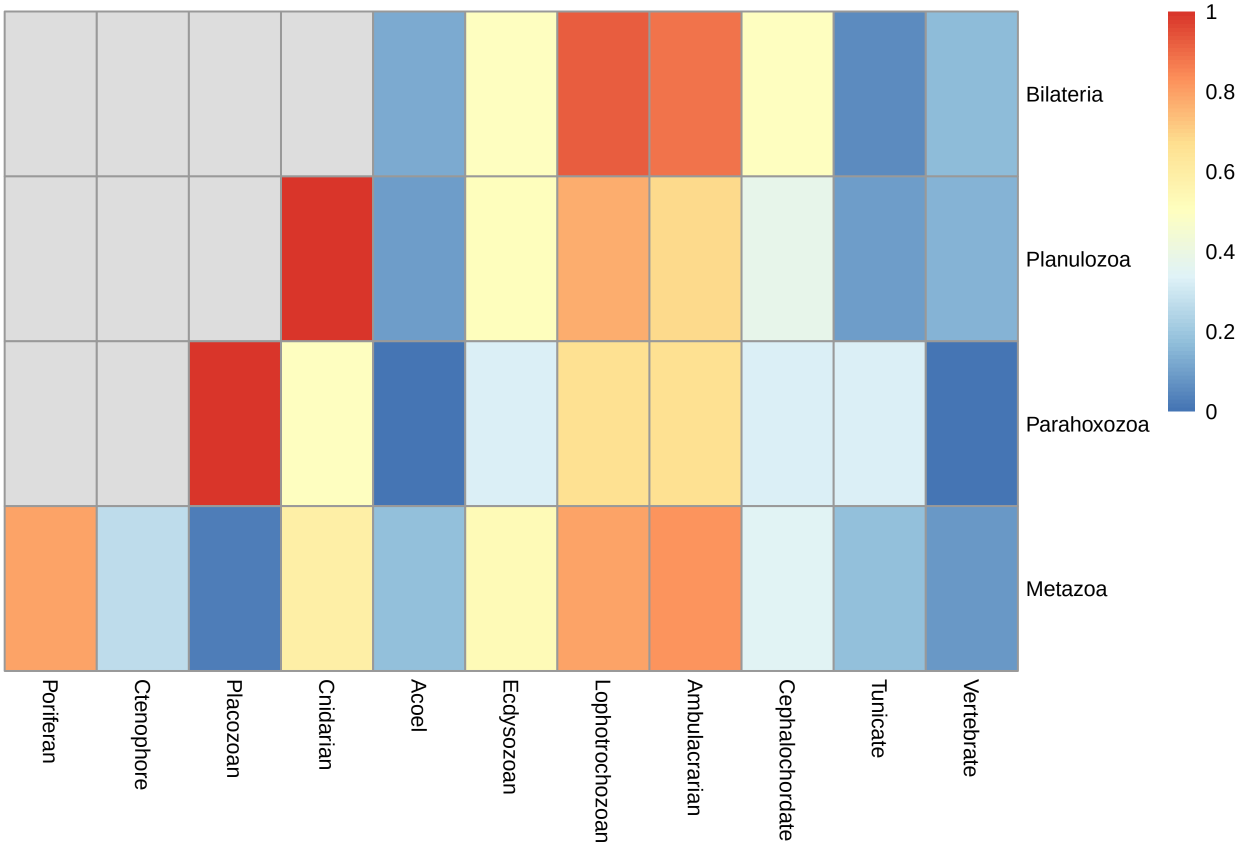


```
ggsave(plot = plot,
       filename = 'fig1_heatmaps_retention.pdf',
       unit = 'cm',
       width = 15,
       height = 5)
```

Figure 2 and supplements

NSMR

```r
library(readr)
library(ggplot2)
library(gridExtra)
library(grid)
library(gdata)
```

```
## gdata: read.xls support for 'XLS' (Excel 97-2004) files ENABLED.

##

## gdata: read.xls support for 'XLSX' (Excel 2007+) files ENABLED.

##
## Attaching package: 'gdata'

## The following object is masked from 'package:gridExtra':
##
##     combine

## The following object is masked from 'package:stats':
##
##     nobs

## The following object is masked from 'package:utils':
##
##     object.size

## The following object is masked from 'package:base':
##
##     startsWith
```

```r
library(ggpubr)
library(cowplot)
```

```
##
## Attaching package: 'cowplot'

## The following object is masked from 'package:ggpubr':
##
##     get_legend
```

```r
library(RColorBrewer)
library(pheatmap)
library(tidyr)

#function taken from https://stackoverflow.com/a/14674703
symlog_trans <- function(base = 10, thr = 1, scale = 1){
  trans <- function(x)
    ifelse(abs(x) < thr, x, sign(x) *
```

```
             (thr + scale * suppressWarnings(log(sign(x) * x / thr, base))))

  inv <- function(x)
    ifelse(abs(x) < thr, x, sign(x) *
             base^((sign(x) * x - thr) / scale) * thr)

  breaks <- function(x){
    sgn <- sign(x[which.max(abs(x))])
    if(all(abs(x) < thr))
      pretty_breaks()(x)
    else if(prod(x) >= 0){
      if(min(abs(x)) < thr)
        sgn * unique(c(pretty_breaks()(c(min(abs(x)), thr)),
                       log_breaks(base)(c(max(abs(x)), thr))))
      else
        sgn * log_breaks(base)(sgn * x)
    } else {
      if(min(abs(x)) < thr)
        unique(c(sgn * log_breaks()(c(max(abs(x)), thr)),
                 pretty_breaks()(c(sgn * thr, x[which.min(abs(x))]))))
      else
        unique(c(-log_breaks(base)(c(thr, -x[1])),
                 pretty_breaks()(c(-thr, thr)),
                 log_breaks(base)(c(thr, x[2]))))
    }
  }
  scales::trans_new(paste("symlog", thr, base, scale, sep = "-"), trans, inv, breaks)
}
```

```
df <- readr::read_csv('key_nodes.tidydf.csv')
```

```
## Parsed with column specification:
## cols(
##   node = col_character(),
##   taxon = col_character(),
##   species = col_character(),
##   random = col_character(),
##   block_id = col_double(),
##   iteration = col_double(),
##   density = col_double(),
##   acc_ls = col_character(),
##   all_acc_ls = col_character(),
##   total_density = col_double(),
##   total_genome_length = col_double(),
##   density_ratio = col_double(),
##   multi_sp = col_double(),
##   para = col_character(),
##   mean_dist_pair = col_double(),
##   mean_dist_pair_norm = col_double(),
##   median_dist_pair = col_double(),
##   median_dist_pair_norm = col_double()
## )
```

Change data into factor, to reorganize the names in a manner consistent with between figures

```
df$taxon <- factor(df$taxon, levels=c('Poriferan','Ctenophore','Placozoan','Cnidarian', 'Acoel','Ecdyso
df$node <- as.factor(df$node)
df$random <- as.factor(df$random)
df$species <- factor(df$species, levels = c('CAPOW', 'SALRO', 'AMPQU', 'SYCCI', 'MNELE', 'PLEBA', 'TRIA
df$log10_density_ratio <- log10(df$density_ratio)

df_para_Met <- df %>% dplyr::filter(para == 'para' & node == 'Metazoa')
df_para_Par <- df %>% dplyr::filter(para == 'para' & node == 'Parahoxozoa')
df_para_Pla <- df %>% dplyr::filter(para == 'para' & node == 'Planulozoa')
df_para_Bil <- df %>% dplyr::filter(para == 'para' & node == 'Bilateria')
df_para_Ver <- df %>% dplyr::filter(para == 'para' & node == 'Vertebrata')
df_para_Lop <- df %>% dplyr::filter(para == 'para'  & node == 'Lophotrochozoa')
df_not_para_Met <- df %>% dplyr::filter(para == 'not_para' & node == 'Metazoa')
df_not_para_Par <- df %>% dplyr::filter(para == 'not_para' & node == 'Parahoxozoa')
df_not_para_Pla <- df %>% dplyr::filter(para == 'not_para' & node == 'Planulozoa')
df_not_para_Bil <- df %>% dplyr::filter(para == 'not_para' & node == 'Bilateria')
df_not_para_Ver <- df %>% dplyr::filter(para == 'not_para' & node == 'Vertebrata')
df_not_para_Lop <- df %>% dplyr::filter(para == 'not_para' & node == 'Lophotrochozoa')
```

Here we define a function to make boxplots of the supp figure (by taxon).

```
map_signif_level <- c(`****` = 1e-04, `***` = 0.001,  `**` = 0.01, `*` = 0.05, ns = 1)

make_plot <- function(tbl,
                      key = "observed",
                      comparisons = list(c("observed", "random")),
                      bracket_y = NULL,
                      ylims = c(-2.5 , 2.5)) {

  if(is.null(bracket_y)) {
    h = ylims[2] - ylims[1]
    bracket_y = c(.9,.825,.75)*h + ylims[1]
  }

  size.summary <- tbl %>% dplyr::filter(random == "observed") %>%  dplyr::group_by(taxon) %>%  dplyr::s
  ggplot(tbl, aes_string(x = 'random', y = 'log10_density_ratio', fill = 'random')) +
    geom_boxplot(outlier.shape = NA) +
    facet_grid(~ taxon) +
    theme_cowplot() +
    theme(axis.title.x = element_blank(), axis.text.x = element_blank()) +
    geom_signif(comparisons = comparisons,
                test = "wilcox.test", test.args = list(paired = FALSE, exact = FALSE), na.rm = TRUE,
                map_signif_level = map_signif_level,
                color="black", tip_length = 0.01, size = .5, textsize = 2,
                y_position = bracket_y,  data = NULL) +
    scale_y_continuous(name = "log10(Density ratio)", limits = c(-2.5, 2.5)) +
    theme(legend.title = element_blank(),
          plot.margin = unit(c(1,0,0,0), units='cm'),
          legend.position = 'bottom',
          legend.justification = 'center',
          strip.text = element_text(size = 6, angle = 90, margin = margin(5,0,5,0,'pt')),
          axis.ticks.x = element_blank(),
          axis.title.y = element_text(size = 7),
          axis.text = element_text(size = 6)) +
```

```r
    geom_text(data=size.summary, aes(x=1,y=2.2,hjust = 0.5,label = label), size = 2, inherit.aes=F)
}
```

Every make_plot call for all the possibilities. Done so so that we can have ggpubr tests with facetting.

```r
p1 <- make_plot(df_not_para_Met)
```

```
## `summarise()` ungrouping output (override with `.groups` argument)
```

```r
p2 <- make_plot(df_para_Met)
```

```
## `summarise()` ungrouping output (override with `.groups` argument)
```

```r
p3 <- make_plot(df_not_para_Par)
```

```
## `summarise()` ungrouping output (override with `.groups` argument)
```

```r
p4 <- make_plot(df_para_Par)
```

```
## `summarise()` ungrouping output (override with `.groups` argument)
```

```r
p5 <- make_plot(df_not_para_Pla)
```

```
## `summarise()` ungrouping output (override with `.groups` argument)
```

```r
p6 <- make_plot(df_para_Pla)
```

```
## `summarise()` ungrouping output (override with `.groups` argument)
```

```r
p7 <- make_plot(df_not_para_Bil)
```

```
## `summarise()` ungrouping output (override with `.groups` argument)
```

```r
p8 <- make_plot(df_para_Bil)
```

```
## `summarise()` ungrouping output (override with `.groups` argument)
```

```r
p9 <- make_plot(df_not_para_Ver)
```

```
## `summarise()` ungrouping output (override with `.groups` argument)
```

```r
p10 <- make_plot(df_para_Ver)
```

```
## `summarise()` ungrouping output (override with `.groups` argument)
```

```r
p11 <- make_plot(df_not_para_Lop)
```

```
## `summarise()` ungrouping output (override with `.groups` argument)
```

```r
p12 <- make_plot(df_para_Lop)
```

```
## `summarise()` ungrouping output (override with `.groups` argument)
```

```r
gridplot <- gridExtra::grid.arrange(grobs = list(p1,p2,p3,p4,p5,p6,p7,p8,p9,p10,p11,p12), ncol = 2)
```

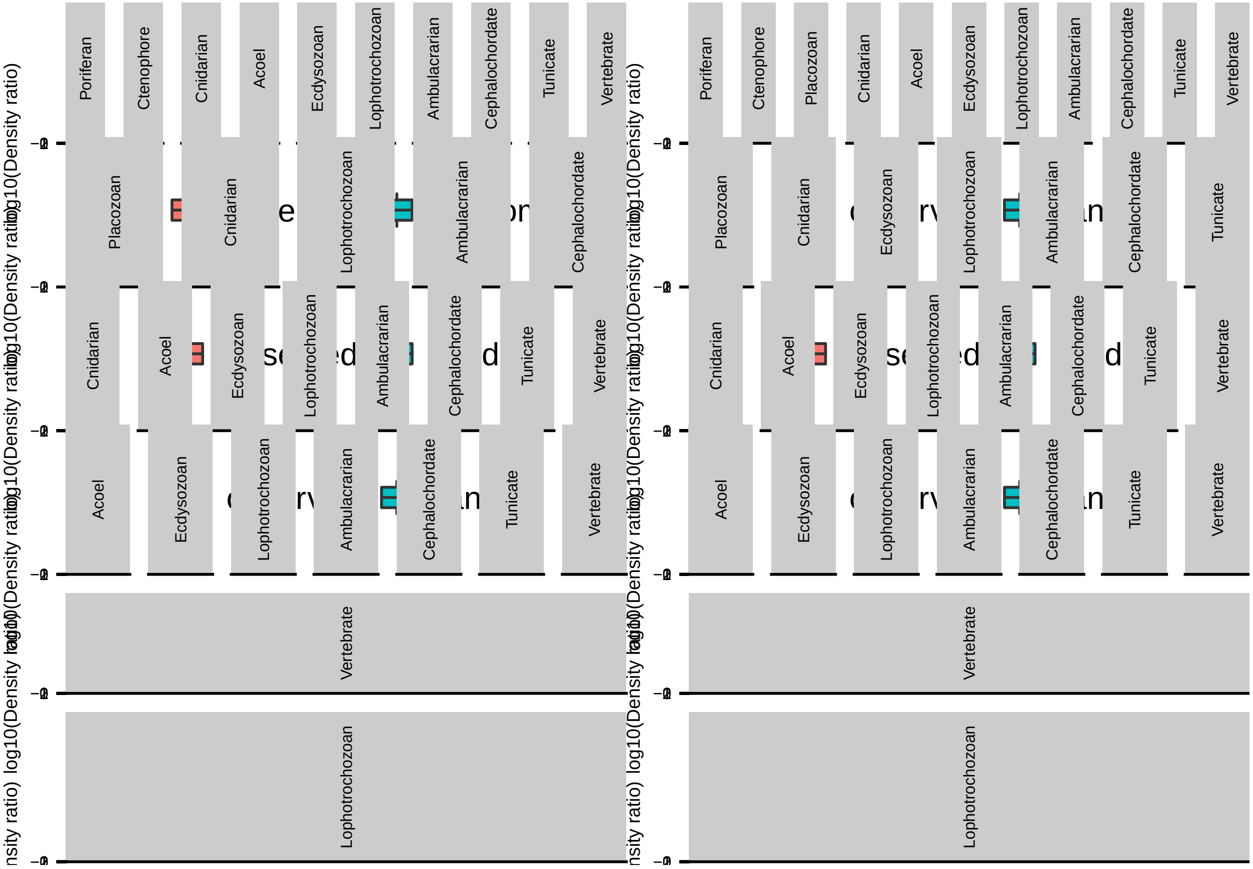


```r
ggsave(plot = gridplot,
       filename = 'SF3.pdf',
       unit = 'cm',
       width = 30,
       height = 80)
```

Now we do the scatterplots for SF4

```r
df <- readr::read_csv('raw_data_scatter.csv')
```

```
## Parsed with column specification:
## cols(
##   multi_sp = col_double(),
##   node = col_character(),
##   Vertebrate = col_double(),
##   Tunicate = col_double(),
##   Cephalochordate = col_double(),
##   Ambulacrarian = col_double(),
##   Lophotrochozoan = col_double(),
##   Ecdysozoan = col_double(),
##   Acoel = col_double(),
##   Cnidarian = col_double(),
##   Placozoan = col_double(),
##   Ctenophore = col_double(),
##   Poriferan = col_double()
```

```
## )
```

```
nodelist <-c('Bilateria', 'Planulozoa', 'Metazoa')
df2 <- df %>% dplyr::filter(node %in% nodelist) %>%
    purrr::discard(~sum(is.na(.x))/length(.x) > 9)
taxons_vars <- colnames(df2)[c(-1,-2)]

# build tables with pairs of values
controlTable <- data.frame(expand.grid(taxons_vars, taxons_vars,
                                       stringsAsFactors = FALSE))
# rename the columns with our taxon names
colnames(controlTable) <- c("x", "y")

# add the key column
controlTable <- cbind(
  data.frame(pair_key = paste(controlTable[[1]], controlTable[[2]]),
             stringsAsFactors = FALSE),
  controlTable)

#Now I can create the new data frame, using the cdata funciton rowrecs_to_blocks(). I'll also carry alo

df2_aug = cdata::rowrecs_to_blocks(
  df2,
  controlTable,
  columnsToCopy = "node")

#let's remove taxon pairs where there is NAs
df2_aug <- na.omit(df2_aug)

splt <- strsplit(df2_aug$pair_key, split = " ", fixed = TRUE)
df2_aug$xv <- vapply(splt, function(si) si[[1]], character(1))
df2_aug$yv <- vapply(splt, function(si) si[[2]], character(1))


# reorder the key columns to be the same order
# as the taxons_vars
df2_aug$xv <- factor(as.character(df2_aug$xv),
                           taxons_vars)
df2_aug$yv <- factor(as.character(df2_aug$yv),
                           taxons_vars)
df2_aug <- df2_aug %>% dplyr::filter(xv != yv)
```

now for the big scatterplot

```
p <- ggplot(df2_aug, aes(x = x, y = y)) +
  geom_point(aes(color = node, shape = node)) +
  facet_grid(yv~xv, scale = "free") +
  scale_y_continuous(trans = symlog_trans(), breaks = c(-10,-1,0,1,10), limits = c(-10.0, 10.0)) +
  scale_x_continuous(trans = symlog_trans(), breaks = c(-10,-1,0,1,10), limits = c(-10.0, 10.0)) +
  ylab(NULL) +
  xlab(NULL) +
  geom_hline(yintercept = 0) +
  geom_vline(xintercept = 0) +
  ggthemes::theme_gdocs() +
  ggpubr::stat_cor(method = 'spearman', label.x = -8, label.y = 8)
```

```r
ggsave('SF4_scatter_density_deviation.pdf',
       plot = p,
       unit = 'cm',
       width = 40,
       height = 40)
```

```
## Warning: Removed 16 rows containing non-finite values (stat_cor).
```

```
## Warning: Removed 16 rows containing missing values (geom_point).
```

```
## Warning: Removed 86 rows containing missing values (geom_text).
```

Correlation of the density deviation (ie. relative change of block density ratio)

```r
df_Bila <- df %>% dplyr::filter(node == 'Bilateria')
df_Planu <- df %>% dplyr::filter(node == 'Planulozoa')
df_Meta <- df %>% dplyr::filter(node == 'Metazoa')

bila <- taxons_vars[c(1,2,3,4,5,6,7)]
planu <- taxons_vars[c(1,2,3,4,5,6,7,8)]
meta <- taxons_vars

corr_matrix_Bila <- matrix(nrow = length(bila), ncol = length(bila))
colnames(corr_matrix_Bila) <- bila
rownames(corr_matrix_Bila) <- bila
pval_corr_matrix_Bila <- corr_matrix_Bila

corr_matrix_Planu <- matrix(nrow = length(planu), ncol = length(planu))
colnames(corr_matrix_Planu) <- planu
rownames(corr_matrix_Planu) <- planu
pval_corr_matrix_Planu <- corr_matrix_Planu

corr_matrix_Meta <- matrix(nrow = length(meta), ncol = length(meta))
colnames(corr_matrix_Meta) <- meta
rownames(corr_matrix_Meta) <- meta
pval_corr_matrix_Meta <-corr_matrix_Meta

retention_matrix_Bila <- corr_matrix_Bila
retention_matrix_Planu <- corr_matrix_Planu
retention_matrix_Meta <- corr_matrix_Meta

for (taxon1 in taxons_vars)
  {
  for (taxon2 in taxons_vars)
    {
    if (taxon1 != taxon2){
      tmp_df <- df_Bila[,c('multi_sp', taxon1, taxon2)]
      tmp_df <- tmp_df %>% na.omit()
      len_df <- dplyr::tally(tmp_df)$n
      if (len_df > 0 ) {retention_matrix_Bila[taxon1, taxon2] <- len_df / 256}
      if (len_df > 10 ) {
        corr_matrix_Bila[taxon1, taxon2] <- cor.test(tmp_df[[taxon1]], tmp_df[[taxon2]], method = "spea
        pval_corr_matrix_Bila[taxon1, taxon2] <- cor.test(tmp_df[[taxon1]], tmp_df[[taxon2]], method =
          }
```

```
      tmp_df <- df_Planu[,c('multi_sp', taxon1, taxon2)]
      tmp_df <- tmp_df %>% na.omit()
      len_df <- length(tmp_df[[taxon1]])
      if (len_df > 0 ) {retention_matrix_Planu[taxon1, taxon2] <- len_df / 162}
      if (len_df > 10 ) {
        corr_matrix_Planu[taxon1, taxon2] <- cor.test(tmp_df[[taxon1]], tmp_df[[taxon2]], method = "spea
        pval_corr_matrix_Planu[taxon1, taxon2] <- cor.test(tmp_df[[taxon1]], tmp_df[[taxon2]], method =
      }

      tmp_df <- df_Meta[,c('multi_sp', taxon1, taxon2)]
      tmp_df <- tmp_df %>% na.omit()
      len_df <- length(tmp_df[[taxon1]])
      if (len_df > 0 ) {retention_matrix_Meta[taxon1, taxon2] <- len_df / 34}
      if (len_df > 10 ) {
        corr_matrix_Meta[taxon1, taxon2] <- cor.test(tmp_df[[taxon1]], tmp_df[[taxon2]], method = "spea
        pval_corr_matrix_Meta[taxon1, taxon2] <- cor.test(tmp_df[[taxon1]], tmp_df[[taxon2]], method =
      }
      }
  }
}
```

Heatmap of density correlation Also heatrmap of pairwise retention (supplement to scatterplots)

```
bk <- c(seq(0, 1,  length=100))

p1 <- pheatmap::pheatmap(corr_matrix_Meta,
                         cluster_rows = F,
                         cluster_cols = F,
                         breaks = bk,
                         legend = F,
                         fontsize = 8,
                         main = 'Metazoa')
```

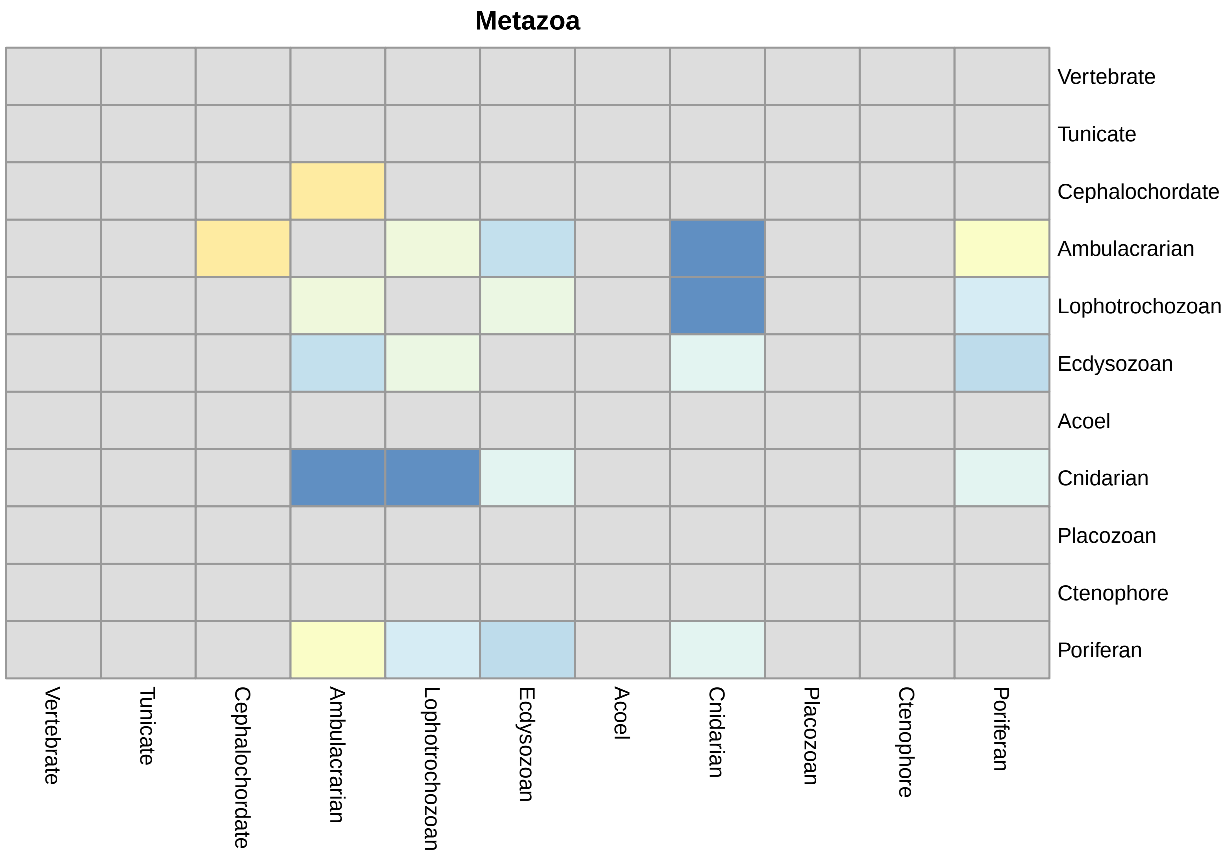


```r
p2 <- pheatmap::pheatmap(corr_matrix_Planu,
                         cluster_rows = F,
                         cluster_cols = F,
                         breaks = bk,
                         legend = F,
                         fontsize = 8,
                         main = 'Planulozoa')
```

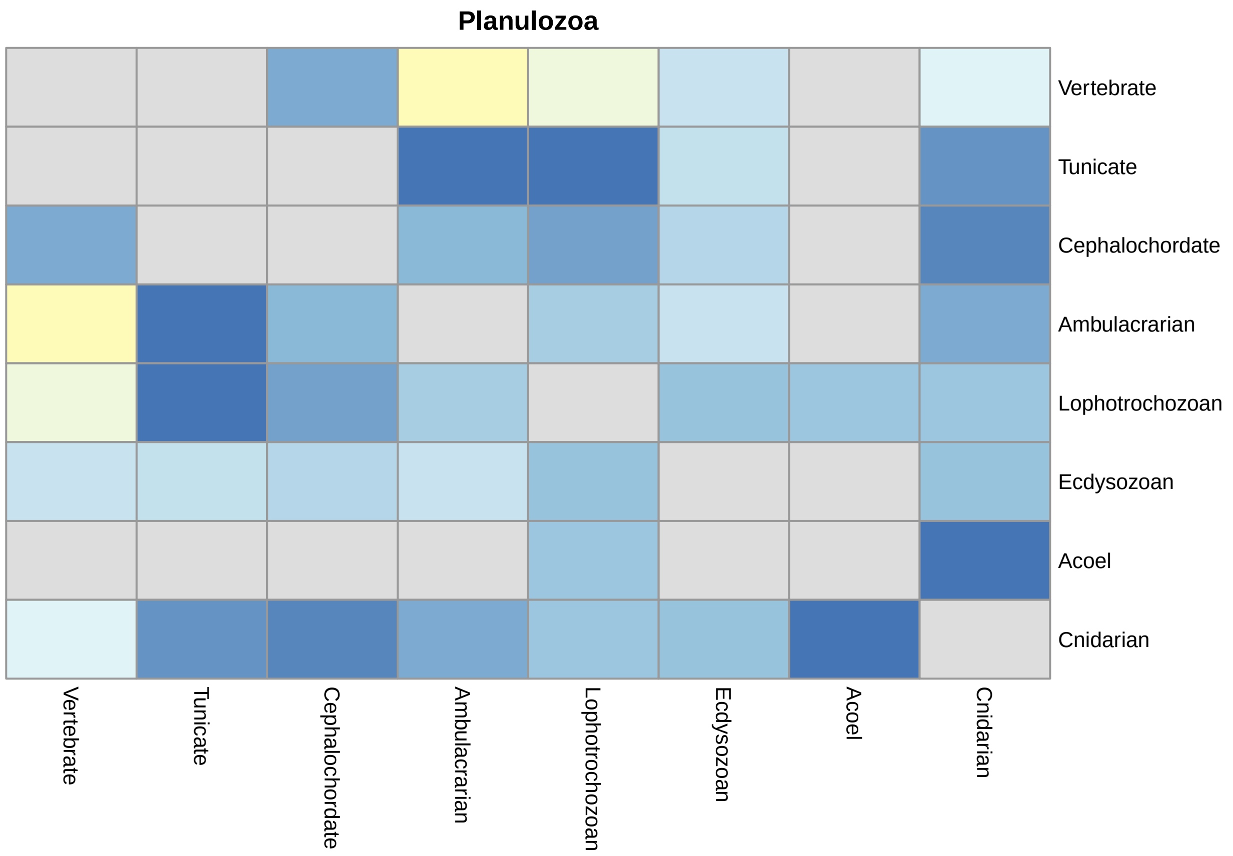


```
p3 <- pheatmap::pheatmap(corr_matrix_Bila,
                         cluster_rows = F,
                         cluster_cols = F,
                         breaks = bk,
                         fontsize = 8,
                         main = 'Bilateria')
```

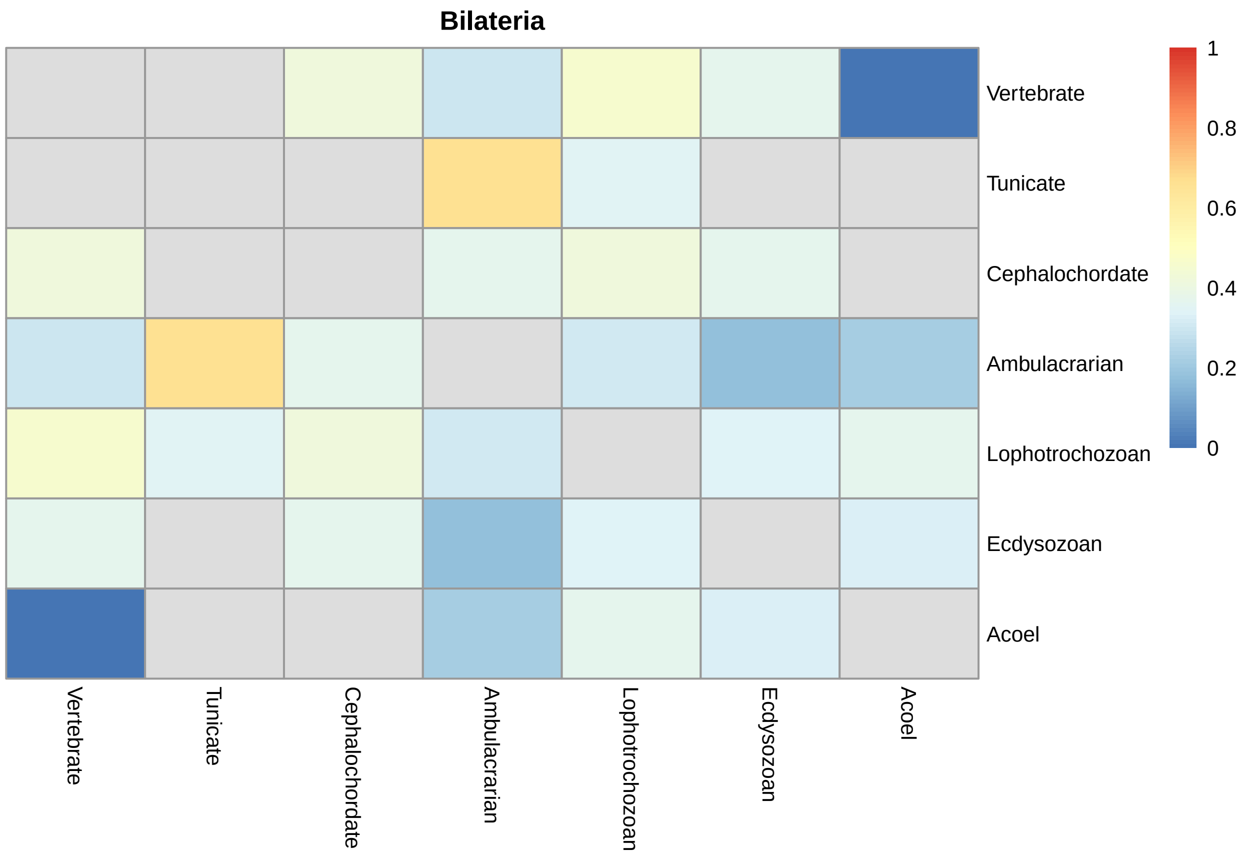


```
gridplot <- gridExtra::grid.arrange(grobs = list(p1$gtable,p2$gtable,p3$gtable), ncol = 3)
```

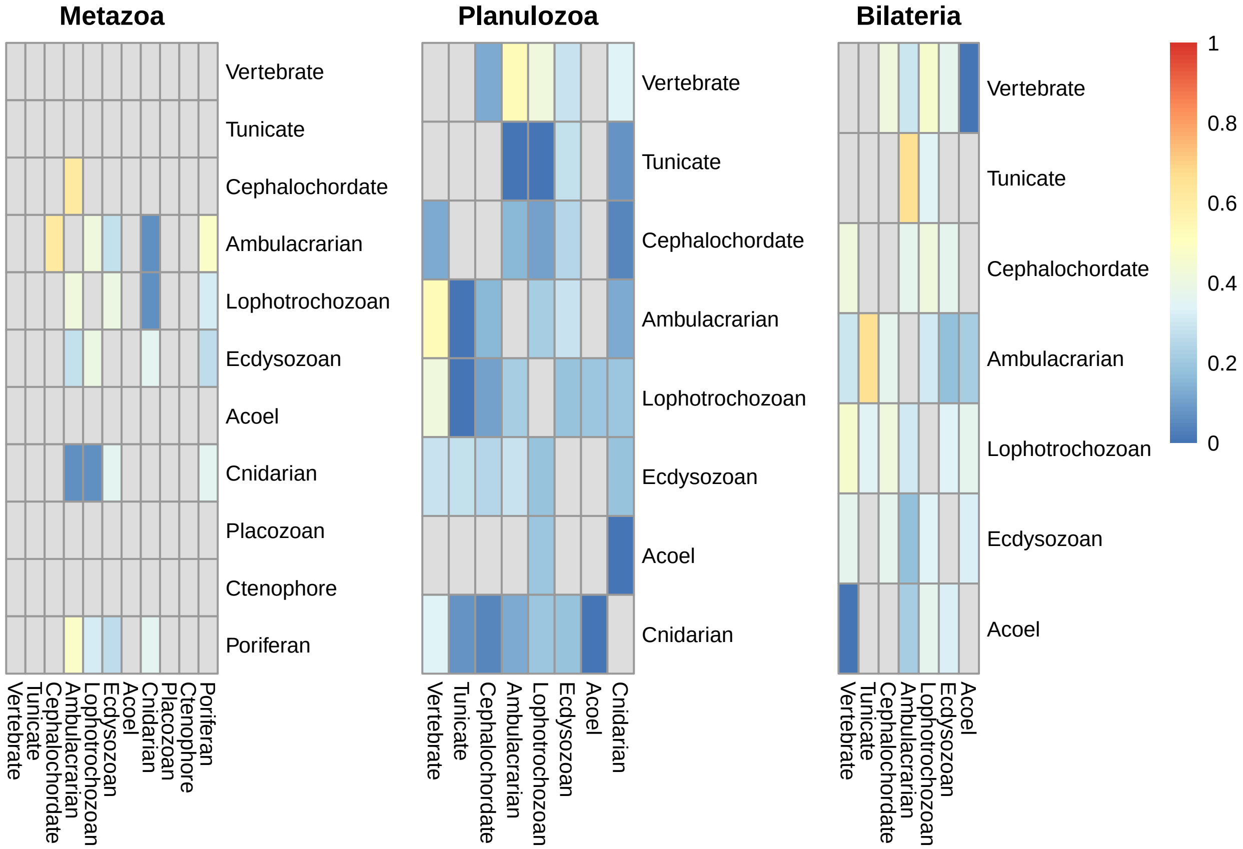


```
ggsave(plot = gridplot,
       filename = 'fig2_heatmaps_corr_density.pdf',
       unit = 'cm',
       width = 16,
       height = 6)
```

Now the pairwise retention matrix (SF5)

```
p4 <- pheatmap::pheatmap(retention_matrix_Meta,
                          cluster_rows = F,
                          cluster_cols = F,
                          breaks = bk,
                          legend = F,
                          fontsize = 8)
```

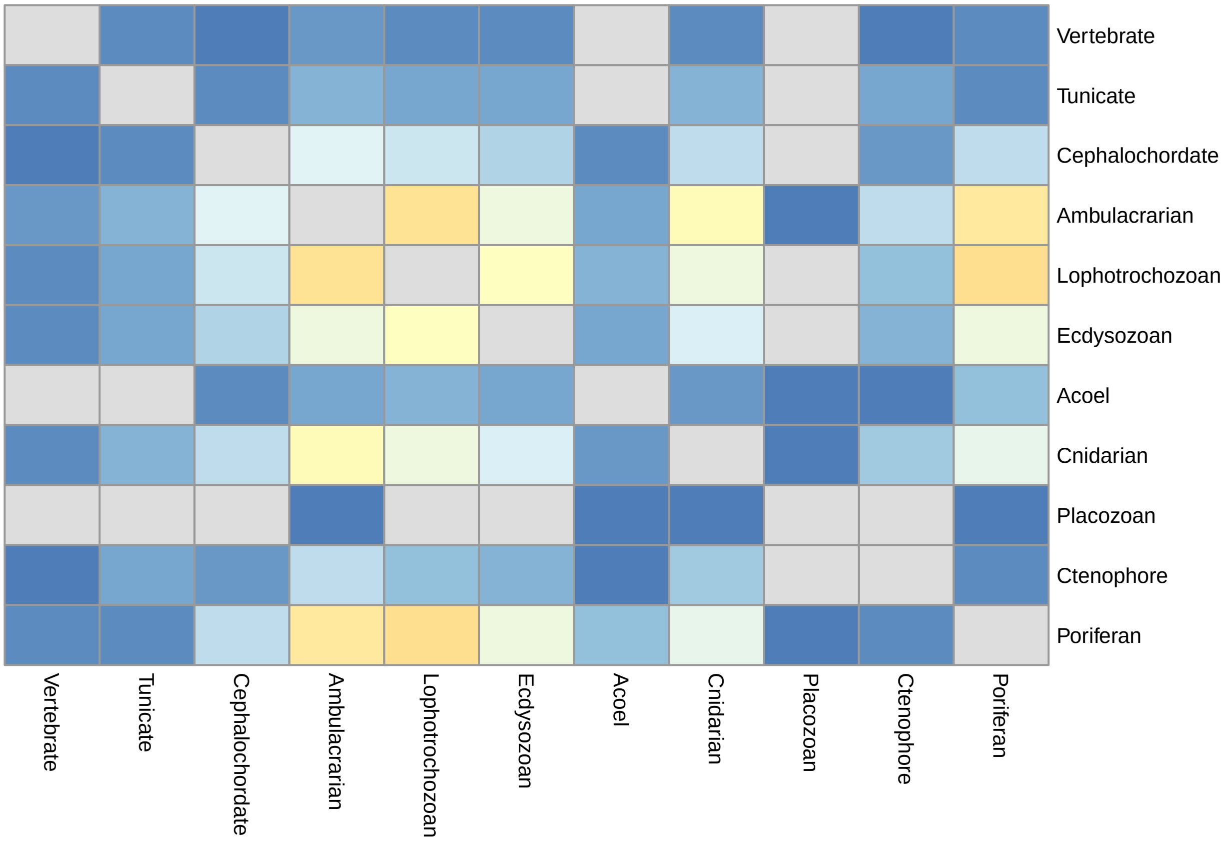


```r
p5 <- pheatmap::pheatmap(retention_matrix_Planu,
                         cluster_rows = F,
                         cluster_cols = F,
                         breaks = bk,
                         legend = F,
                         fontsize = 8)
```

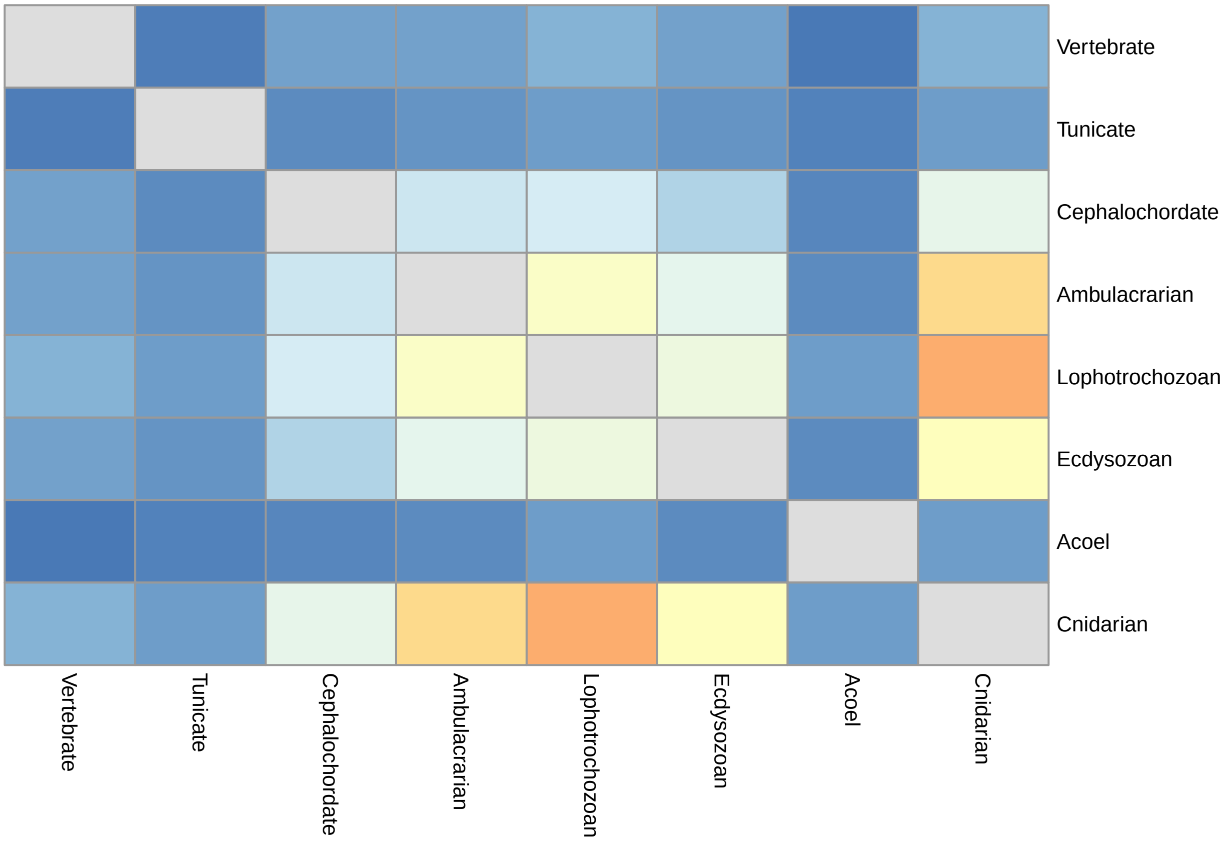


```
p6 <- pheatmap::pheatmap(retention_matrix_Bila,
                         cluster_rows = F,
                         cluster_cols = F,
                         breaks = bk,
                         fontsize = 8)
```

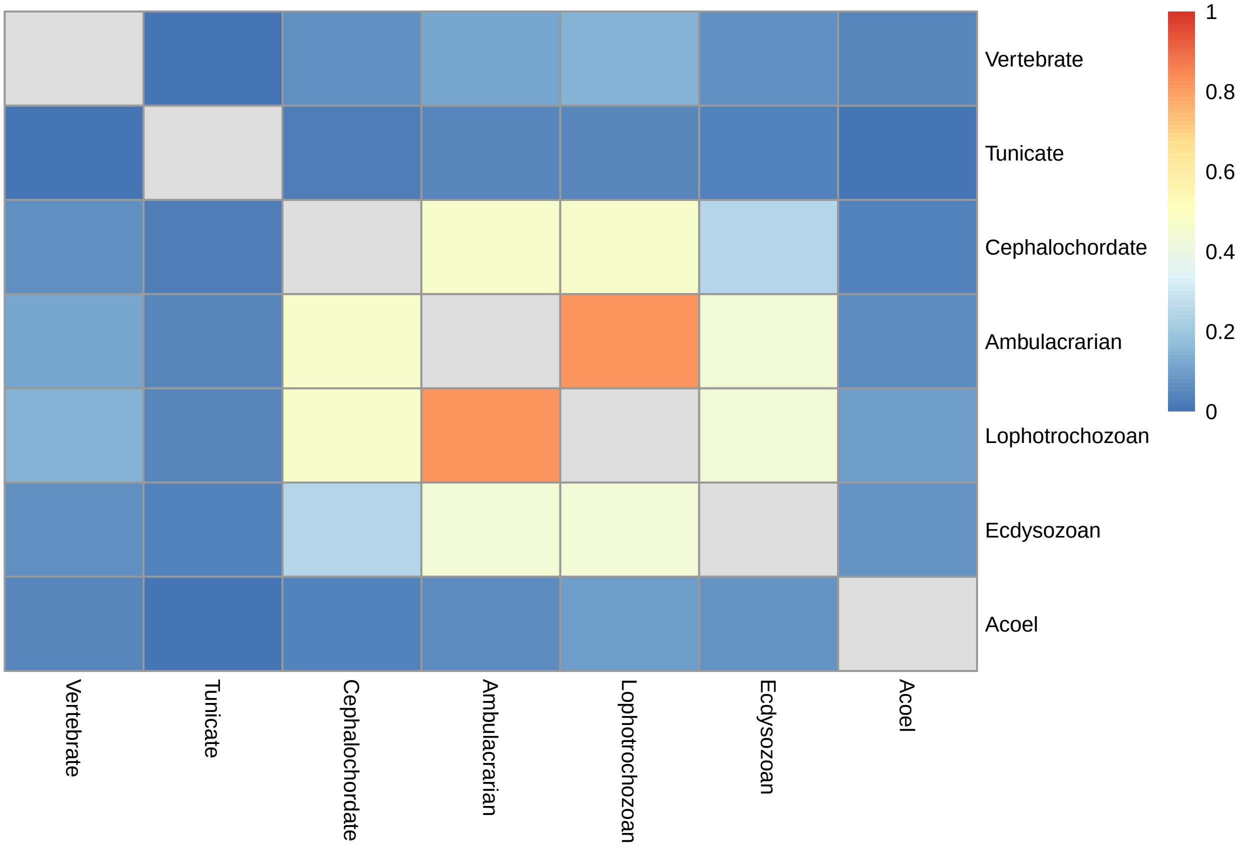


```
gridplot2 <- gridExtra::grid.arrange(grobs = list(p4$gtable,p5$gtable,p6$gtable), ncol = 3)
```

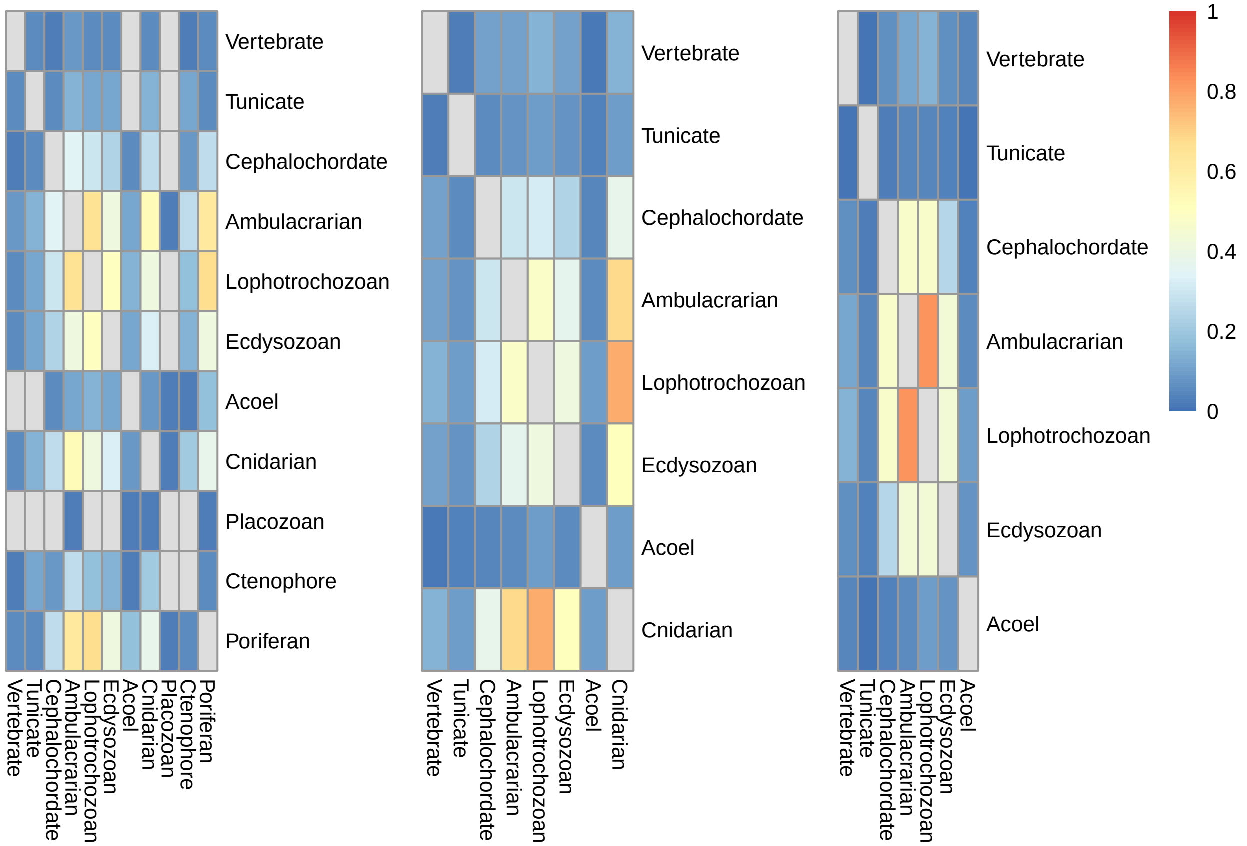


```
ggsave(plot = gridplot2,
       filename = 'SF6A_pairwise retention_corr_check.pdf',
       unit = 'cm',
       width = 16,
       height = 6)
```

Figure 3 and supplements

NSMR

```r
library(readr)
library(ggplot2)
library(ggrepel)
library(ggpubr)
library(cowplot)
```

```
##
## Attaching package: 'cowplot'

## The following object is masked from 'package:ggpubr':
##
##     get_legend
```

Now we'll load up the data, change iteration/phylo_hypothesis to factor type data

```r
df_cragi <- readr::read_csv("CRAGI_tidy_corr.csv")
```

```
## Parsed with column specification:
## cols(
##   node = col_character(),
##   taxon = col_character(),
##   species = col_character(),
##   random = col_character(),
##   block_id = col_double(),
##   iteration = col_double(),
##   density = col_double(),
##   acc_ls = col_character(),
##   total_density = col_double(),
##   density_ratio = col_double(),
##   multi_sp = col_double(),
##   para = col_character(),
##   block_corr = col_double()
## )
```

```r
df_calmi <- readr::read_csv("CALMI_tidy_corr.csv")
```

```
## Parsed with column specification:
## cols(
##   node = col_character(),
##   taxon = col_character(),
##   species = col_character(),
##   random = col_character(),
##   block_id = col_double(),
##   iteration = col_double(),
##   density = col_double(),
##   acc_ls = col_character(),
##   total_density = col_double(),
```

```
##   density_ratio = col_double(),
##   multi_sp = col_double(),
##   para = col_character(),
##   block_corr = col_double()
## )
```

```r
df_musmu <- readr::read_csv("MUSMU_tidy_corr.csv")
```

```
## Parsed with column specification:
## cols(
##   node = col_character(),
##   taxon = col_character(),
##   species = col_character(),
##   random = col_character(),
##   block_id = col_double(),
##   iteration = col_double(),
##   density = col_double(),
##   acc_ls = col_character(),
##   total_density = col_double(),
##   density_ratio = col_double(),
##   multi_sp = col_double(),
##   para = col_character(),
##   block_corr = col_double()
## )
```

```r
df_mizye <- readr::read_csv("MIZYE_tidy_corr.csv")
```

```
## Parsed with column specification:
## cols(
##   node = col_character(),
##   taxon = col_character(),
##   species = col_character(),
##   random = col_character(),
##   block_id = col_double(),
##   iteration = col_double(),
##   density = col_double(),
##   acc_ls = col_character(),
##   total_density = col_double(),
##   density_ratio = col_double(),
##   multi_sp = col_double(),
##   para = col_character(),
##   block_corr = col_double()
## )
```

```r
df_sacko <- readr::read_csv("SACKO_tidy_corr.csv")
```

```
## Parsed with column specification:
## cols(
##   node = col_character(),
##   taxon = col_character(),
##   species = col_character(),
##   random = col_character(),
##   block_id = col_double(),
##   iteration = col_double(),
##   density = col_double(),
##   acc_ls = col_character(),
```

```
##   total_density = col_double(),
##   density_ratio = col_double(),
##   multi_sp = col_double(),
##   para = col_character(),
##   block_corr = col_double()
## )
```

```
df_strpu <- readr::read_csv("STRPU_tidy_corr.csv")
```

```
## Parsed with column specification:
## cols(
##   node = col_character(),
##   taxon = col_character(),
##   species = col_character(),
##   random = col_character(),
##   block_id = col_double(),
##   iteration = col_double(),
##   density = col_double(),
##   acc_ls = col_character(),
##   total_density = col_double(),
##   density_ratio = col_double(),
##   multi_sp = col_double(),
##   para = col_character(),
##   block_corr = col_double()
## )
```

```
map_signif_level <- c(`****` = 1e-04, `***` = 0.001,  `**` = 0.01, `*` = 0.05, ns = 1)

make_plot <- function(tbl,
                      key = "observed",
                      comparisons = list(c("observed", "random")),
                      bracket_y = NULL,
                      ylims = c(-0.5 , 1.5)) {

  if(is.null(bracket_y)) {
    h = ylims[2] - ylims[1]
    bracket_y = c(.9,.825,.75)*h + ylims[1]
  }

size.summary <- tbl %>% dplyr::filter(random == "observed") %>%  dplyr::group_by(node, para) %>%  dplyr
  ggplot(tbl, aes_string(x = 'random', y = 'block_corr', fill = 'random')) +
    #geom_violin(draw_quantiles = c(0.25, 0.5, 0.75)) +
    geom_boxplot(outlier.shape=NA) +
    facet_grid(para ~ node) +
    theme_cowplot(font_size = 6) +
   geom_signif(comparisons = list(c("observed", "random")),
               test = "wilcox.test", test.args = list(paired = FALSE, exact = FALSE), na.rm = TRUE,
               map_signif_level = map_signif_level,
               color="black", tip_length = 0.01, size = .25, textsize = 1,
               y_position = bracket_y,  data = NULL) +
    scale_y_continuous(name = "Block correlation", limits = c(-0.5, 1.5)) +
    theme(legend.title = element_blank(),
          plot.margin = unit(c(1,0,0,0), units='cm'),
          legend.position = 'bottom',
          legend.justification = 'center',
```

```r
          strip.text = element_text(size = 6, margin = margin(5,0,5,0,'pt')),
          axis.ticks.x = element_blank(),
          axis.title.y = element_text(size = 7),
          axis.text = element_text(size = 6)) +
  geom_text(data = size.summary, aes(x=1, y= 1.2,hjust=0.4,label=label), inherit.aes=F, size = 1)
}
```

Make boxplots

```r
p_calmi <- make_plot(df_calmi)
```

```
## `summarise()` regrouping output by 'node' (override with `.groups` argument)
```

```r
p_cragi <- make_plot(df_cragi)
```

```
## `summarise()` regrouping output by 'node' (override with `.groups` argument)
```

```r
p_mizye <- make_plot(df_mizye)
```

```
## `summarise()` regrouping output by 'node' (override with `.groups` argument)
```

```r
p_musmu <- make_plot(df_musmu)
```

```
## `summarise()` regrouping output by 'node' (override with `.groups` argument)
```

```r
p_sacko <- make_plot(df_sacko)
```

```
## `summarise()` regrouping output by 'node' (override with `.groups` argument)
```

```r
p_strpu <- make_plot(df_strpu)
```

```
## `summarise()` regrouping output by 'node' (override with `.groups` argument)
```

```r
ggsave(filename = 'SF7A_calmi_corr_boxplots.pdf',
       plot = p_calmi,
       units = 'cm',
       width = 13,
       height = 7)
```

```
## Warning: Removed 1 rows containing non-finite values (stat_boxplot).
```

```r
ggsave(filename = 'SF7B_cragi_corr_boxplots.pdf',
       plot = p_cragi,
       units = 'cm',
       width = 13,
       height = 7)

ggsave(filename = 'SF7C_mizye_corr_boxplots.pdf',
       plot = p_mizye,
       units = 'cm',
       width = 13,
       height = 7)

ggsave(filename = 'SF7D_musmu_corr_boxplots.pdf',
       plot = p_musmu,
       units = 'cm',
       width = 13,
       height = 7)
```

```r
ggsave(filename = 'SF7E_sacko_corr_boxplots.pdf',
       plot = p_sacko,
       units = 'cm',
       width = 13,
       height = 7)

ggsave(filename = 'SF7F_strpu_corr_boxplots.pdf',
       plot = p_strpu,
       units = 'cm',
       width = 13,
       height = 7)
```

```
## Warning: Removed 7 rows containing non-finite values (stat_boxplot).
```

Figure 4 - wnt regression lines

NSMR

```
library(readr)
library(ggplot2)
library(ggrepel)
library(ggpubr)
library(cowplot)
```

```
##
## Attaching package: 'cowplot'
## The following object is masked from 'package:ggpubr':
##
##     get_legend
```

```
library(patchwork)
```

```
##
## Attaching package: 'patchwork'
## The following object is masked from 'package:cowplot':
##
##     align_plots
```

Now we'll load up the data

```
df_wg_stats <- readr::read_csv("genome_stats.csv")
```

```
## Parsed with column specification:
## cols(
##   species = col_character(),
##   genome_length = col_double(),
##   gene_count = col_double()
## )
```

```
df_wnt <- readr::read_csv("wnt.tidydf.csv")
```

```
## Parsed with column specification:
## cols(
##   node = col_character(),
##   taxon = col_character(),
##   species = col_character(),
##   random = col_character(),
##   block_id = col_double(),
##   iteration = col_double(),
##   density = col_double(),
##   acc_ls = col_character(),
##   total_density = col_double(),
##   density_ratio = col_double(),
##   multi_sp = col_character(),
```

```
##   para = col_character()
## )
```

```
df_hox <- readr::read_csv("hox.tidydf.csv")
```

```
## Parsed with column specification:
## cols(
##   node = col_character(),
##   taxon = col_character(),
##   species = col_character(),
##   random = col_character(),
##   block_id = col_double(),
##   iteration = col_double(),
##   density = col_double(),
##   acc_ls = col_character(),
##   total_density = col_double(),
##   density_ratio = col_double(),
##   multi_sp = col_character(),
##   para = col_character()
## )
```

Now we'll add some columns to wgd stats, drop the cols we don't need in wnt and hox df to cleanup

```
df_wg_stats$recip_wgd <- df_wg_stats$genome_length/ df_wg_stats$gene_count


prep_my_df <- function(tbl){
  outdf <- tbl %>%
    dplyr::filter(random == 'observed') %>%
    dplyr::mutate(invertebrate = dplyr::case_when(
                                    taxon == 'Vertebrate'  ~ 'Vertebrate',
                                    taxon != 'Vertebrate'  ~ 'Invertebrate'),
                  recip_block_density = 1 / density) %>%
    dplyr::select(c(taxon, species, density, total_density, invertebrate, recip_block_density)) %>%
    dplyr::left_join(df_wg_stats, by = 'species')
  outdf$taxon <- as.factor(outdf$taxon)
  return(outdf)
}

df_wnt <- prep_my_df(df_wnt)
df_hox <- prep_my_df(df_hox)
```

Now this is for getting linear fit, also preparing the palette

```
wg_lm <- lm(formula = recip_wgd ~ genome_length,
                              data = df_wg_stats)
wg_lm2  <- lm(formula = recip_wgd / 2 ~ genome_length,
                              data = df_wg_stats)

mypalette <- list('#1CA1FB', '#CC52AB', '#FA7850', '#AB1E3D', '#32B559', '#E61F00', '#106E82')
names(mypalette) <- c('Cnidarian', 'Tunicate', 'Ecdysozoan', 'Lophotrochozoan', 'Ambulacrarian', 'Cephal
```

This is to prepare for shading between the two ablines we provide

```
slope_wgd <- coef(wg_lm)[[2]]
slope_2_wgd <- coef(wg_lm2)[[2]]
intercept_wgd <- coef(wg_lm)[[1]]
```

```
intercept_2_wgd <- coef(wg_lm2)[[1]]

df_wg_stats$estimated_recip_wgd <- df_wg_stats$genome_length* slope_wgd + intercept_wgd
df_wg_stats$twice_estimated_recip_wgd <- df_wg_stats$genome_length* slope_2_wgd + intercept_2_wgd
```

plot with regression as boundaries

```
make_plot <- function(tbl){
ggplot(tbl, aes_string(x = 'genome_length', y = 'recip_block_density', color = 'taxon')) +
  ggplot2::geom_ribbon(aes(x = genome_length,
                           ymin = estimated_recip_wgd,
                           ymax = twice_estimated_recip_wgd),
              data = df_wg_stats,
              inherit.aes = F,
              fill = 'grey90',
              color = 'grey80',
              linetype = 2)+
  ggplot2::scale_color_manual(values = mypalette)+
  ggplot2::geom_smooth(method = 'lm', se = F, size = 0.5) +
  ggplot2::geom_point()+
  cowplot::theme_cowplot() +
  ggplot2::scale_x_continuous(name = 'Assembly size (bp)',
                              trans = 'log10') +
  ggplot2::scale_y_continuous(name = 'Reciprocal of gene density (bp/gene)',
                              trans = 'log10')+
  ggplot2::theme(legend.title = element_blank(),
          plot.margin = unit(c(1,0,0,0), units='cm'),
          legend.position = 'bottom',
          legend.justification = 'center',
          strip.text = element_text(size = 6, angle = 90, margin = margin(5,0,5,0,'pt')),
          axis.title.x = element_text(size = 7),
          axis.title.y = element_text(size = 7),
          axis.text = element_text(size = 6),
          legend.text = element_text(size = 7))
}

wnt_p <- make_plot(df_wnt)
hox_p <- make_plot(df_hox)

prow <- cowplot::plot_grid(wnt_p + ggplot2::theme(legend.position="none"),
                           hox_p + ggplot2::theme(legend.position="none"),
                           align = 'vh',
                           labels = c("A", "B", "C"),
                           hjust = -1,
                           nrow = 1)
```

```
## `geom_smooth()` using formula 'y ~ x'
## `geom_smooth()` using formula 'y ~ x'
```

```
#legend_b <- get_legend(hox_p + theme(legend.position="bottom"))

p <- cowplot::plot_grid(prow, ncol = 1, rel_heights = c(1, .2))

ggsave(plot = p,
       filename = 'hox_wnt_regressions.pdf',
```

```
       unit = 'cm',
       width = 15,
       height = 7)
```

SF10 hox and wnt boxplots

NSMR

```
library(readr)
library(ggplot2)
library(gridExtra)
library(grid)
library(ggpubr)
library(cowplot)
```

```
##
## Attaching package: 'cowplot'

## The following object is masked from 'package:ggpubr':
##
##     get_legend
```

```
library(RColorBrewer)
```

Now we'll load up the data, change iteration/phylo_hypothesis to factor type data

```
df_wnt <- readr::read_csv("wnt.tidydf.csv")
```

```
## Parsed with column specification:
## cols(
##   node = col_character(),
##   taxon = col_character(),
##   species = col_character(),
##   random = col_character(),
##   block_id = col_double(),
##   iteration = col_double(),
##   density = col_double(),
##   acc_ls = col_character(),
##   total_density = col_double(),
##   density_ratio = col_double(),
##   multi_sp = col_character(),
##   para = col_character()
## )
```

```
df_hox <- readr::read_csv("hox.tidydf.csv")
```

```
## Parsed with column specification:
## cols(
##   node = col_character(),
##   taxon = col_character(),
##   species = col_character(),
##   random = col_character(),
##   block_id = col_double(),
##   iteration = col_double(),
##   density = col_double(),
```

```
##   acc_ls = col_character(),
##   total_density = col_double(),
##   density_ratio = col_double(),
##   multi_sp = col_character(),
##   para = col_character()
## )
```

Here we define a function to make boxplots of the supp figure.

```
map_signif_level <- c(`****` = 1e-04, `***` = 0.001,  `**` = 0.01, `*` = 0.05, ns = 1)

make_plot <- function(tbl,
                      key = "observed",
                      comparisons = list(c("observed", "random")),
                      bracket_y = NULL,
                      ylims = c(-2.5 , 2.5)) {

  if(is.null(bracket_y)) {
    h = ylims[2] - ylims[1]
    bracket_y = c(.9,.825,.75)*h + ylims[1]
  }

  size.summary <- tbl %>% dplyr::filter(random == "observed") %>%  dplyr::group_by(taxon) %>%  dplyr::su
  ggplot(tbl, aes_string(x = 'random', y = 'log10_density_ratio', fill = 'random')) +
    geom_boxplot(outlier.shape = NA) +
    facet_grid(~ taxon) +
    theme_cowplot() +
    theme(axis.title.x = element_blank(), axis.text.x = element_blank()) +
    geom_signif(comparisons = comparisons,
                test = "wilcox.test", test.args = list(paired = FALSE, exact = FALSE), na.rm = TRUE,
                map_signif_level = map_signif_level,
                color="black", tip_length = 0.01, size = .5, textsize = 2,
                y_position = bracket_y,  data = NULL) +
    scale_y_continuous(name = "log10(Density ratio)", limits = c(-2.5, 2.5)) +
    theme(legend.title = element_blank(),
          plot.margin = unit(c(1,0,0,0), units='cm'),
          legend.position = 'bottom',
          legend.justification = 'center',
          strip.text = element_text(size = 6, angle = 90, margin = margin(5,0,5,0,'pt')),
          axis.ticks.x = element_blank(),
          axis.title.y = element_text(size = 7),
          axis.text = element_text(size = 6)) +
    geom_text(data=size.summary, aes(x=1,y=2.2,hjust = 0.5,label = label), size = 2, inherit.aes=F)
}
```

Modify data format to make it easy to use

```
df_wnt$taxon <- factor(df_wnt$taxon, levels=c('Poriferan','Ctenophore','Placozoan','Cnidarian', 'Acoel'
df_wnt$node <- as.factor(df_wnt$node)
df_wnt$random <- as.factor(df_wnt$random)
df_wnt$species <- as.factor(df_wnt$species)
df_wnt$log10_density_ratio <- log10(df_wnt$density_ratio)

df_hox$taxon <-  factor(df_hox$taxon, levels=c('Poriferan','Ctenophore','Placozoan','Cnidarian', 'Acoel
df_hox$node <- as.factor(df_hox$node)
df_hox$random <- as.factor(df_hox$random)
```

```r
df_hox$species <- as.factor(df_hox$species)
df_hox$log10_density_ratio <- log10(df_hox$density_ratio)
```

Make the plots and save them

```r
hox_p <- make_plot(df_hox)
```

```
## `summarise()` ungrouping output (override with `.groups` argument)
```

```r
wnt_p <- make_plot(df_wnt)
```

```
## `summarise()` ungrouping output (override with `.groups` argument)
```

```r
gridplot <- gridExtra::grid.arrange(grobs = list(hox_p,wnt_p), ncol = 2)
```

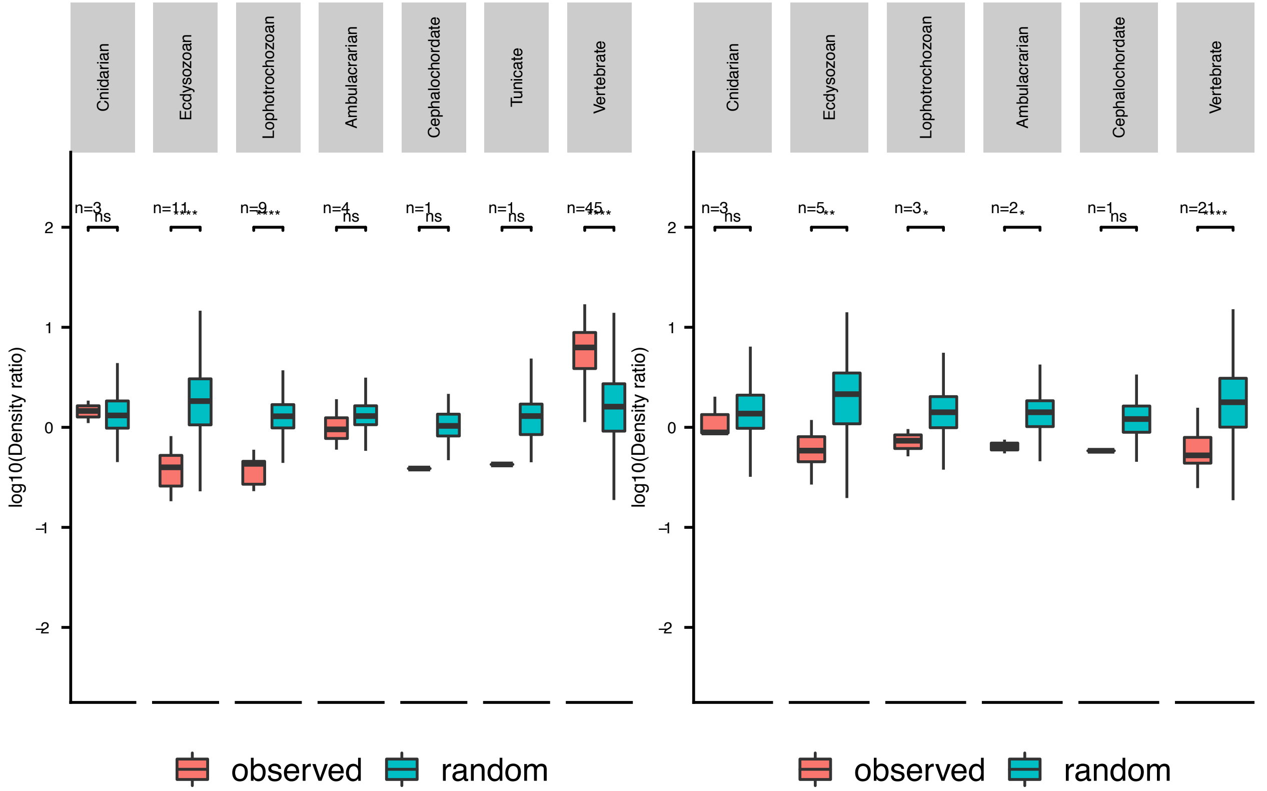


```r
ggsave(plot = gridplot,
       filename = 'hox_wnt.pdf',
       unit = 'cm',
       width = 15,
       height = 12)
```

SF5

NSMR

```r
library(readr)
library(ggplot2)
library(ggrepel)
library(ggpubr)
library(scales)
```

```
##
## Attaching package: 'scales'

## The following object is masked from 'package:readr':
##
##     col_factor
```

```r
library(cowplot)
```

```
##
## Attaching package: 'cowplot'

## The following object is masked from 'package:ggpubr':
##
##     get_legend
```

Now we'll load up the data, change iteration/phylo_hypothesis to factor type data

```r
df <- readr::read_csv("key_nodes.tidydf.csv")
```

```
## Parsed with column specification:
## cols(
##   node = col_character(),
##   taxon = col_character(),
##   species = col_character(),
##   random = col_character(),
##   block_id = col_double(),
##   iteration = col_double(),
##   density = col_double(),
##   acc_ls = col_character(),
##   all_acc_ls = col_character(),
##   total_density = col_double(),
##   total_genome_length = col_double(),
##   density_ratio = col_double(),
##   multi_sp = col_double(),
##   para = col_character(),
##   mean_dist_pair = col_double(),
##   mean_dist_pair_norm = col_double(),
##   median_dist_pair = col_double(),
##   median_dist_pair_norm = col_double()
## )
```

```r
df_stats <- readr::read_csv("genome_stats.csv")
```

```
## Parsed with column specification:
## cols(
##   species = col_character(),
##   genome_length = col_double(),
##   gene_count = col_double()
## )
```

```r
df <- dplyr::left_join(df, df_stats, by = 'species')
```

Plot reciprocal genome density as a funtion of assembly size

```r
df$reciprocal_total_density <- 1 / df$total_density

p<- ggplot2::ggplot(df, aes_string(x= 'genome_length', y = 'reciprocal_total_density')) +
    ggplot2::geom_smooth(method = lm) +
    ggplot2::geom_point() +
    ggpubr::stat_cor(size = 2, label.y = 175000) +
    ggpubr::stat_regline_equation(size = 2, label.y = 200000) +
    ggplot2::scale_y_continuous(name = "Reciprocal of whole genome density") +
    ggplot2::scale_x_continuous(name = "Assembly size") +
    cowplot::theme_cowplot(font_size = 8)

ggsave(filename = 'SF2_Metazoa_assembly_size_total_density.pdf',
       plot = p,
       units = 'cm',
       width = 10,
       height = 6)
```

```
## `geom_smooth()` using formula 'y ~ x'
```

SF8

NSMR

```
library(readr)
library(ggplot2)
library(gridExtra)
library(grid)
library(gdata)
```

```
## gdata: read.xls support for 'XLS' (Excel 97-2004) files ENABLED.

##

## gdata: read.xls support for 'XLSX' (Excel 2007+) files ENABLED.

##
## Attaching package: 'gdata'

## The following object is masked from 'package:gridExtra':
##
##     combine

## The following object is masked from 'package:stats':
##
##     nobs

## The following object is masked from 'package:utils':
##
##     object.size

## The following object is masked from 'package:base':
##
##     startsWith
```

```
library(ggpubr)
library(cowplot)
```

```
##
## Attaching package: 'cowplot'

## The following object is masked from 'package:ggpubr':
##
##     get_legend
```

```
library(RColorBrewer)
library(pheatmap)
library(tidyr)
```

```
df <- readr::read_csv('key_nodes.tidydf.csv')
```

```
## Parsed with column specification:
## cols(
##   node = col_character(),
```

```
##   taxon = col_character(),
##   species = col_character(),
##   random = col_character(),
##   block_id = col_double(),
##   iteration = col_double(),
##   density = col_double(),
##   acc_ls = col_character(),
##   all_acc_ls = col_character(),
##   total_density = col_double(),
##   total_genome_length = col_double(),
##   density_ratio = col_double(),
##   multi_sp = col_double(),
##   para = col_character(),
##   mean_dist_pair = col_double(),
##   mean_dist_pair_norm = col_double(),
##   median_dist_pair = col_double(),
##   median_dist_pair_norm = col_double()
## )
```

Here we define a function to make boxplots by taxon

```
map_signif_level <- c(`****` = 1e-04, `***` = 0.001,  `**` = 0.01, `*` = 0.05, ns = 1)

make_plot <- function(tbl,
                      key = "observed",
                      comparisons = list(c("observed", "random")),
                      bracket_y = NULL,
                      ylims = c(0 , 0.0001)) {

  if(is.null(bracket_y)) {
    h = ylims[2] - ylims[1]
    bracket_y = c(.9,.825,.75)*h + ylims[1]
  }

  size.summary <- tbl %>% dplyr::filter(random == "observed") %>%  dplyr::group_by(taxon) %>%  dplyr::su
  ggplot(tbl, aes_string(x = 'random', y = 'median_dist_pair_norm', fill = 'random')) +
    geom_boxplot(outlier.shape = NA) +
    facet_grid(~ taxon) +
    theme_cowplot() +
    theme(axis.title.x = element_blank(), axis.text.x = element_blank()) +
    geom_signif(comparisons = comparisons,
                test = "wilcox.test", test.args = list(paired = FALSE, exact = FALSE), na.rm = TRUE,
                map_signif_level = map_signif_level,
                color="black", tip_length = 0.01, size = .5, textsize = 2,
                y_position = bracket_y,  data = NULL) +
    scale_y_continuous(name = "Median distance between consecutive genes (fraction of genome)", limits =
    theme(legend.title = element_blank(),
          plot.margin = unit(c(1,0,0,0), units='cm'),
          legend.position = 'bottom',
          legend.justification = 'center',
          strip.text = element_text(size = 6, angle = 90, margin = margin(5,0,5,0,'pt')),
          axis.ticks.x = element_blank(),
          axis.title.y = element_text(size = 7),
          axis.text = element_text(size = 6)) +
    geom_text(data=size.summary, aes(x=1,y= 0.000093,hjust = 0.5,label = label), size = 2, inherit.aes=F
```

```
}
```

```
df$taxon <- factor(df$taxon, levels=c('Poriferan','Ctenophore','Placozoan','Cnidarian', 'Acoel','Ecdyso
df$node <- as.factor(df$node)
df$random <- as.factor(df$random)
df$species <- factor(df$species, levels = c('CAPOW', 'SALRO', 'AMPQU', 'SYCCI', 'MNELE', 'PLEBA', 'TRIA

df_para_Met <- df %>% dplyr::filter(para == 'para' & node == 'Metazoa')
df_para_Par <- df %>% dplyr::filter(para == 'para' & node == 'Parahoxozoa')
df_para_Pla <- df %>% dplyr::filter(para == 'para' & node == 'Planulozoa')
df_para_Bil <- df %>% dplyr::filter(para == 'para' & node == 'Bilateria')
df_para_Ver <- df %>% dplyr::filter(para == 'para' & node == 'Vertebrata')
df_para_Lop <- df %>% dplyr::filter(para == 'para'  & node == 'Lophotrochozoa')
df_not_para_Met <- df %>% dplyr::filter(para == 'not_para' & node == 'Metazoa')
df_not_para_Par <- df %>% dplyr::filter(para == 'not_para' & node == 'Parahoxozoa')
df_not_para_Pla <- df %>% dplyr::filter(para == 'not_para' & node == 'Planulozoa')
df_not_para_Bil <- df %>% dplyr::filter(para == 'not_para' & node == 'Bilateria')
df_not_para_Ver <- df %>% dplyr::filter(para == 'not_para' & node == 'Vertebrata')
df_not_para_Lop <- df %>% dplyr::filter(para == 'not_para' & node == 'Lophotrochozoa')
```

Every make_plot call for all the possibilities. Done so so that we can have ggpubr tests with facetting.

```
p1 <- make_plot(df_not_para_Met)
```

```
## `summarise()` ungrouping output (override with `.groups` argument)
```

```
p2 <- make_plot(df_para_Met)
```

```
## `summarise()` ungrouping output (override with `.groups` argument)
```

```
p3 <- make_plot(df_not_para_Par)
```

```
## `summarise()` ungrouping output (override with `.groups` argument)
```

```
p4 <- make_plot(df_para_Par)
```

```
## `summarise()` ungrouping output (override with `.groups` argument)
```

```
p5 <- make_plot(df_not_para_Pla)
```

```
## `summarise()` ungrouping output (override with `.groups` argument)
```

```
p6 <- make_plot(df_para_Pla)
```

```
## `summarise()` ungrouping output (override with `.groups` argument)
```

```
p7 <- make_plot(df_not_para_Bil)
```

```
## `summarise()` ungrouping output (override with `.groups` argument)
```

```
p8 <- make_plot(df_para_Bil)
```

```
## `summarise()` ungrouping output (override with `.groups` argument)
```

```
p9 <- make_plot(df_not_para_Ver)
```

```
## `summarise()` ungrouping output (override with `.groups` argument)
```

```
p10 <- make_plot(df_para_Ver)
```

```
## `summarise()` ungrouping output (override with `.groups` argument)
```

```r
p11 <- make_plot(df_not_para_Lop)
```

```
## `summarise()` ungrouping output (override with `.groups` argument)
```

```r
p12 <- make_plot(df_para_Lop)
```

```
## `summarise()` ungrouping output (override with `.groups` argument)
```

```r
gridplot <- gridExtra::grid.arrange(grobs = list(p1,p2,p3,p4,p5,p6,p7,p8,p9,p10,p11,p12), ncol = 2)
```

```
## Warning: Removed 73 rows containing non-finite values (stat_boxplot).
## Warning: Removed 322 rows containing non-finite values (stat_boxplot).
## Warning: Removed 62 rows containing non-finite values (stat_boxplot).
## Warning: Removed 423 rows containing non-finite values (stat_boxplot).
## Warning: Removed 680 rows containing non-finite values (stat_boxplot).
## Warning: Removed 834 rows containing non-finite values (stat_boxplot).
## Warning: Removed 639 rows containing non-finite values (stat_boxplot).
## Warning: Removed 272 rows containing non-finite values (stat_boxplot).
## Warning: Removed 34 rows containing non-finite values (stat_boxplot).
## Warning: Removed 207 rows containing non-finite values (stat_boxplot).
## Warning: Removed 107 rows containing non-finite values (stat_boxplot).
```

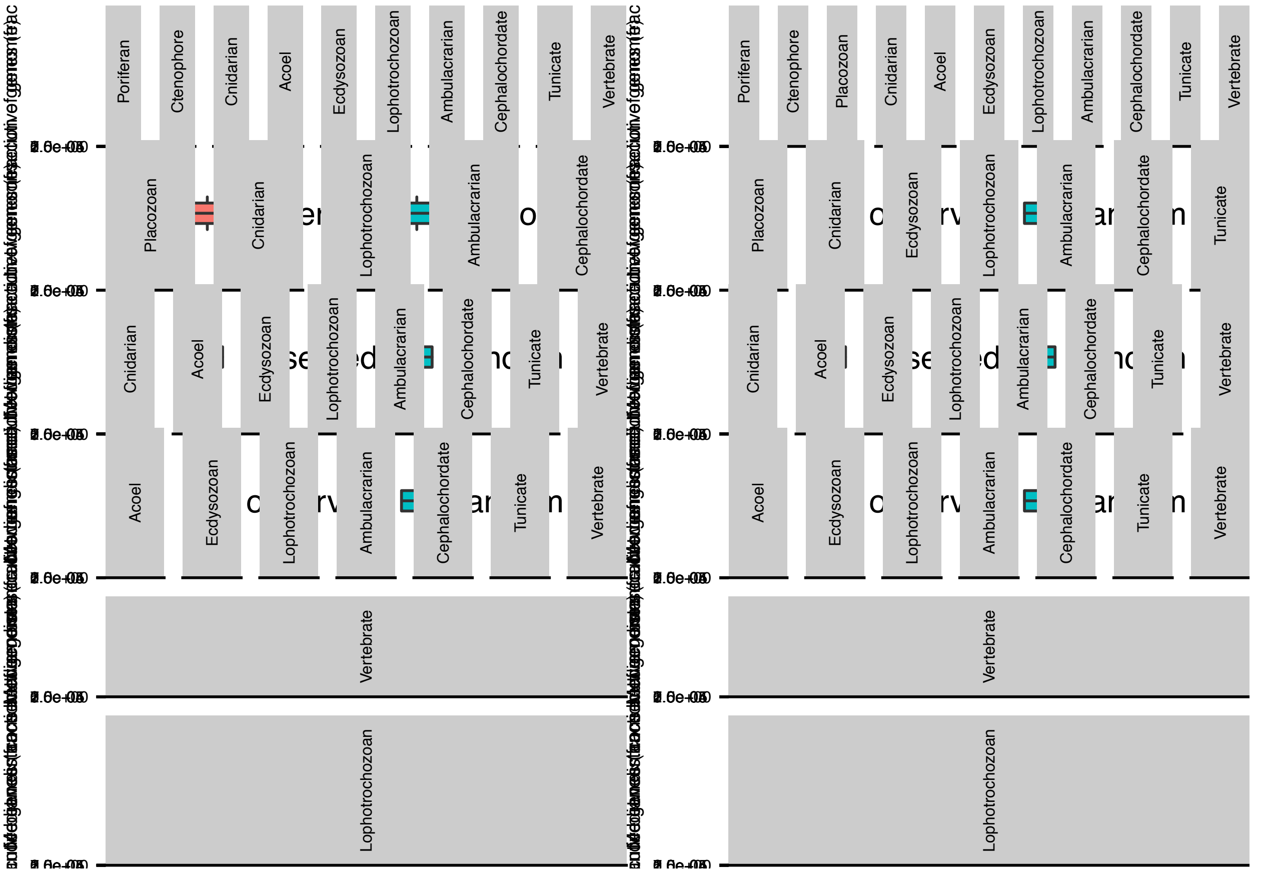

```
ggsave(plot = gridplot,
       filename = 'SF5_consecutivepairs_taxons_meds.pdf',
       unit = 'cm',
       width = 30,
       height = 80)
```

./02. Data prep/LongestIsoforms_ENSEMBL.py

```
#! /usr/bin/env python3

import sys
import re
from itertools import groupby

print(
"""
This is LongestIsoforms_NCBI.py
Usage: LongestIsoforms_NCBI.py input.fa output.fa
"""
)

input_file = sys.argv[1]
output_file = sys.argv[2]



print("the input file is: "+input_file)
print("the output file is: "+output_file)



def fasta_parse(fasta_iterator): #when given a fasta file, the function yields
tuples of (header, sequence)
    with open(fasta_iterator) as f:
        fasta_iterator = (x[1] for x in groupby(f, lambda line: line[0] == ">"))
#create alternating groups of lines starting by > and the others
        for line in fasta_iterator:
            header_string = line.__next__()[1:].strip()     #line[0] is the ">",
so everything else on the line is the header string
            sequence = "".join(s.strip() for s in fasta_iterator.__next__())  #We
join all the objects in the list following the header list (groupby output)
            Search_string = 'gene\:([^\s]+)'
            gene_name = re.search(Search_string, header_string).group(1)
            yield (header_string, sequence, gene_name)



fasta = fasta_parse(input_file) #calls the function we defined to parse the file
passed as an argument

output = []

#The groupby creates a generator, and the if and else loops iterate through it.
#If you uncomment the block with the print statements, the generator will be
exhausted, so the output file (generated by the second block) will be empty.
"""

for sublist_iterator in groupby(sorted(fasta, key = lambda x: x[2]), lambda x:
```

```
x[2]): #group sequences by gene names. Groupby works only on contiguous lines, so
we need to sort the lists according to gene names. This way transcripts (sublists)
will get fused with contiguous sublists sharing the same gene name.
    gene = list(sublist_iterator[1])  #sublist_iterator[1] is a list of lists.
Higher level lists are genes.
    if len(gene) == 1:                  #One transcript by gene.
        output.append(">"+str(gene[0][0])+'\n'+str(gene[0][1]))
    else:                               #len(gene) only equal or greater than 1.
Can't be zero.
        longest_transcript = sorted(gene, key=lambda isoform: isoform[1], reverse
= False) #sorted function, the key being the second field of the subsublist (i.e.,
the sequence)
        output.append(">"+str(longest_transcript[0]
[0])+'\n'+str(longest_transcript[0][1]))
"""
with open(output_file,"w") as g:
    for sublist_iterator in groupby(sorted(fasta, key = lambda x: x[2]), lambda x:
x[2]): #group sequences by gene names. Groupby works only on contiguous lines, so
we need to sort the lists according to gene names. This way transcripts (sublists)
will get fused with contiguous sublists sharing the same gene name.
        gene = list(sublist_iterator[1])      #sublist_iterator[1] is a generator
of lists. Higher level lists are genes.
        if len(gene) == 1:                  #One transcript by gene.
            g.write(">"+str(gene[0][0])+'\n'+str(gene[0][1])+'\n')
        else:                               #len(gene) only equal or greater than
1. Can't be zero.
            longest_transcript = sorted(gene, key=lambda isoform: len(isoform[1]),
reverse = True) #sorted function, the key being the second field of the subsublist
(i.e., the sequence length). In the event that several isoforms have the same
length, the alphabetical sorting inherited  by the gene name sorting prevails, and
the isoform with the lowest number (e.g. a or X1) and highest sequence length is
retained
            g.write(">"+str(longest_transcript[0]
[0])+'\n'+str(longest_transcript[0][1])+'\n')
```

./02. Data prep/LongestIsoforms_HOIHO_HYDVU_PTYFL.py

```
#! /usr/bin/env python3

import sys
import re
from itertools import groupby

print(
"""
This is LongestIsoforms_HOIHO_HYDVU_PTYFL.py
Usage: LongestIsoforms_HOIHO_HYDVU_PTYFL.py input.fa output.fa
Note: should work with any file with headers beginning by an Augustus-type AN
(space-delimited fields)
"""
```

```
)

input_file = sys.argv[1]
output_file = sys.argv[2]



print("the input file is: "+input_file)
print("the output file is: "+output_file)


def fasta_parse(fasta_file): #when given a fasta file, the function yields tuples
of (header, sequence)
    with open(fasta_file) as f:
        fasta_iterator = (x[1] for x in groupby(f, lambda line: line[0] == ">"))
#create alternating groups of lines starting by > and the others
        for header in fasta_iterator:
            header_string = header.__next__()[1:].strip()       #line[0] is the
">", so everything else on the line is the header string
            sequence = "".join(s.strip() for s in fasta_iterator.__next__())  #We
join all the objects in the list following the header list (groupby output)
            Search_string = '(.*)\.\w+[^$, ^\s]'
            gene_name = re.search(Search_string, header_string).group(1)
            yield (header_string, sequence, gene_name) #since we start on a >, 2
__next__ actions mean we go back on a > line


fasta = fasta_parse(input_file) #calls the function we defined to parse the file
passed as an argument

output = []

#The groupby creates a generator, and the if and else loops iterate through it.
#If you uncomment the block with the print statements, the generator will be
exhausted, so the output file (generated by the second block) will be empty.
"""

for sublist_iterator in groupby(sorted(fasta, key = lambda x: x[2]), lambda x:
x[2]): #group sequences by gene names. Groupby works only on contiguous lines, so
we need to sort the lists according to gene names. This way transcripts (sublists)
will get fused with contiguous sublists sharing the same gene name.
    gene = list(sublist_iterator[1])  #sublist_iterator[1] is a list of lists.
Higher level lists are genes.
    if len(gene) == 1:                  #One transcript by gene.
        output.append(">"+str(gene[0][0])+'\n'+str(gene[0][1]))
    else:                               #len(gene) only equal or greater than 1.
Can't be zero.
        longest_transcript = sorted(gene, key=lambda isoform: isoform[1], reverse
= False) #sorted function, the key being the second field of the subsublist (i.e.,
the sequence)
        output.append(">"+str(longest_transcript[0]
[0])+'\n'+str(longest_transcript[0][1]))
"""
with open(output_file,"w") as g:
```

```
    for sublist_iterator in groupby(sorted(fasta, key = lambda x: x[2]), lambda x:
x[2]): #group sequences by gene names. Groupby works only on contiguous lines, so
we need to sort the lists according to gene names. This way transcripts (sublists)
will get fused with contiguous sublists sharing the same gene name.
        gene = list(sublist_iterator[1])      #sublist_iterator[1] is a generator
of lists. Higher level lists are genes.
        if len(gene) == 1:                    #One transcript by gene.
            g.write(">"+str(gene[0][0])+'\n'+str(gene[0][1])+'\n')
        else:                                 #len(gene) only equal or greater than
1. Can't be zero.
            longest_transcript = sorted(gene, key=lambda isoform: len(isoform[1]),
reverse = True) #sorted function, the key being the second field of the subsublist
(i.e., the sequence length). In the event that several isoforms have the same
length, the alphabetical sorting inherited  by the gene name sorting prevails, and
the isoform with the lowest number (e.g. a or X1) and highest sequence length is
retained
            g.write(">"+str(longest_transcript[0]
[0])+'\n'+str(longest_transcript[0][1])+'\n')
```

./02. Data prep/LongestIsoforms_NCBI.py

```
#! /usr/bin/env python3

import sys
import re
from itertools import groupby

print(
"""
This is LongestIsoforms_NCBI_v2.py
Usage: LongestIsoforms_NCBI_v2.py input.fa input.gff output.fa
"""
)

input_file = sys.argv[1]
input_gff = sys.argv[2]
output_file = sys.argv[3]


print("the input fasta file is: "+input_file)
print("the input gff file is: "+input_gff)


d_gene_ID = {}
#dictionary build from gff file. Gene ID as values, protein ID as keys.
search_string_gID = 'GeneID:(\d+)'
search_string_protID = ';protein_id=(\w+\.\d+)'

with open(input_gff, "r") as f:
```

```
    for line in f:
        line = line.strip()
        try:
            d_value = re.search(search_string_gID, line).group(1) #gene ID is
value
            d_key = re.search(search_string_protID, line).group(1) #protein ID is
key
            if d_key not in d_gene_ID.keys():
                d_gene_ID[d_key] = d_value
        except:
            pass #skips line if any re.search is "None"

def fasta_parse(fasta_file): #when given a fasta file, the function yields tuples
of (header, sequence)
    with open(fasta_file) as g:
        fasta_iterator = (x[1] for x in groupby(g, lambda line: line[0] == ">"))
#create alternating groups of lines starting by > and the others
        for header in fasta_iterator:
            header_string = header.__next__()[1:].strip()       #line[0] is the
">", so everything else on the line is the header string
            sequence = "".join(s.strip() for s in fasta_iterator.__next__()) #We
join all the objects in the list following the header list (groupby output)
            search_string_prot_an = '([^\s]+)\s'
            protein_AN = re.search(search_string_prot_an, header_string).group(1)
            try:
                gene_ID = d_gene_ID[protein_AN]
                yield (header_string, sequence, d_gene_ID[protein_AN])
            except KeyError: #happens when protein not in the gff, e.g. when gff
was filtered
                pass

fasta = fasta_parse(input_file) #calls the function we defined to parse the file
passed as an argument



#The groupby creates a generator, and the if and else loops iterate through it.
#If you uncomment the block with the print statements, the generator will be
exhausted, so the output file (generated by the second block) will be empty.
#This can be useful if you want to have a quick look on STDOUT.
"""
output = []

for sublist_iterator in groupby(sorted(fasta, key = lambda x: x[2]), lambda x:
x[2]): #group sequences by gene IDs. Groupby works only on contiguous lines, so we
need to sort the lists according to gene names. This way transcripts (sublists)
will get fused with contiguous sublists sharing the same gene name.
    gene = list(sublist_iterator[1])  #sublist_iterator[1] is a list of lists.
Higher level lists are genes.
    if len(gene) == 1:                  #One transcript by gene.
        output.append(">"+str(gene[0][0])+'\n'+str(gene[0][1]))
    else:                               #len(gene) only equal or greater than 1.
Can't be zero.
        longest_transcript = sorted(gene, key=lambda isoform: isoform[1], reverse
= False) #sorted function, the key being the second field of the subsublist (i.e.,
```

```
the sequence)
        output.append(">"+str(longest_transcript[0]
[0])+'\n'+str(longest_transcript[0][1]))

for x in output:
    print(x)
"""

#Block of code to generate the file
with open(output_file,"w") as g:
    for sublist_iterator in groupby(sorted(fasta, key = lambda x: x[2]), lambda x:
x[2]): #group sequences by gene names. Groupby works only on contiguous lines, so
we need to sort the lists according to gene IDs. This way transcripts (sublists)
will get fused with contiguous sublists sharing the same gene name.
        gene = list(sublist_iterator[1])      #sublist_iterator[1] is a generator
of lists. Higher level lists are genes.
        if len(gene) == 1:                    #One transcript by gene.
            g.write(">"+str(gene[0][0])+'\n'+str(gene[0][1])+'\n')
        else:                                 #len(gene) only equal or greater than
1. Can't be zero.
            longest_transcript = sorted(gene, key=lambda isoform: len(isoform[1]),
reverse = True) #sorted function, the key being the second field of the subsublist
(i.e., the sequence length). In the event that several isoforms have the same
length, the order within the source file prevails.
            g.write(">"+str(longest_transcript[0]
[0])+'\n'+str(longest_transcript[0][1])+'\n')
```

./02. Data prep/LongestIsoforms_SCHME.py

```
#! /usr/bin/env python3

import sys
import re
from itertools import groupby

print(
"""
This is LongestIsoforms_SCHME.py
Usage: LongestIsoforms_SCHME.py input.fa input.gff output.fa
"""
)

input_file = sys.argv[1]
input_gff = sys.argv[2]
output_file = sys.argv[3]
```

```
print("the input fasta file is: "+input_file)
print("the input gff file is: "+input_gff)


d_gene_ID = {}
#dictionary build from gff file. Gene ID as values, protein ID as keys.
search_string_gID = 'Parent=(SMESG\w+\.\d+)'
search_string_protID = 'ID=(SMEST\w+\.\d+)'



with open(input_gff, "r") as f:
    for line in f:
        line = line.strip()
        try:
            d_value = re.search(search_string_gID, line).group(1) #gene ID is
value
            d_key = re.search(search_string_protID, line).group(1) #protein ID is
key
            if d_key not in d_gene_ID.keys():
                d_gene_ID[d_key] = d_value
        except:
            pass #skips line if any re.search is "None"

def fasta_parse(fasta_file): #when given a fasta file, the function yields tuples
of (header, sequence)
    with open(fasta_file) as g:
        fasta_iterator = (x[1] for x in groupby(g, lambda line: line[0] == ">"))
#create alternating groups of lines starting by > and the others
        for header in fasta_iterator:
            header_string = header.__next__()[1:].strip()       #line[0] is the
">", so everything else on the line is the header string
            sequence = "".join(s.strip() for s in fasta_iterator.__next__()) #We
join all the objects in the list following the header list (groupby output)
            search_string_prot_an = '([^\s]+)\s'
            gene_ID = d_gene_ID[header_string]
            yield (header_string, sequence, gene_ID)

fasta = fasta_parse(input_file) #calls the function we defined to parse the file
passed as an argument


#The groupby creates a generator, and the if and else loops iterate through it.
#If you uncomment the block with the print statements, the generator will be
exhausted, so the output file (generated by the second block) will be empty.
#This can be useful if you want to have a quick look on STDOUT.
"""
output = []

for sublist_iterator in groupby(sorted(fasta, key = lambda x: x[2]), lambda x:
x[2]): #group sequences by gene IDs. Groupby works only on contiguous lines, so we
need to sort the lists according to gene names. This way transcripts (sublists)
will get fused with contiguous sublists sharing the same gene name.
    gene = list(sublist_iterator[1])  #sublist_iterator[1] is a list of lists.
```

```
Higher level lists are genes.
    if len(gene) == 1:                    #One transcript by gene.
        output.append(">"+str(gene[0][0])+'\n'+str(gene[0][1]))
    else:                                 #len(gene) only equal or greater than 1.
Can't be zero.
        longest_transcript = sorted(gene, key=lambda isoform: isoform[1], reverse
= False) #sorted function, the key being the second field of the subsublist (i.e.,
the sequence)
        output.append(">"+str(longest_transcript[0]
[0])+'\n'+str(longest_transcript[0][1]))

for x in output:
    print(x)
"""

#Block of code to generate the file
with open(output_file,"w") as g:
    for sublist_iterator in groupby(sorted(fasta, key = lambda x: x[2]), lambda x:
x[2]): #group sequences by gene names. Groupby works only on contiguous lines, so
we need to sort the lists according to gene IDs. This way transcripts (sublists)
will get fused with contiguous sublists sharing the same gene name.
        gene = list(sublist_iterator[1])      #sublist_iterator[1] is a generator
of lists. Higher level lists are genes.
        if len(gene) == 1:                    #One transcript by gene.
            g.write(">"+str(gene[0][0])+'\n'+str(gene[0][1])+'\n')
        else:                                 #len(gene) only equal or greater than
1. Can't be zero.
            longest_transcript = sorted(gene, key=lambda isoform: len(isoform[1]),
reverse = True) #sorted function, the key being the second field of the subsublist
(i.e., the sequence length). In the event that several isoforms have the same
length, the order within the source file prevails.
            g.write(">"+str(longest_transcript[0]
[0])+'\n'+str(longest_transcript[0][1])+'\n')
```

./02. Data prep/pymakeMap.py

```
#! /usr/bin/env python3

import argparse
import sys
import os
import re
from operator import itemgetter
import csv
import itertools


def parsegff(inputfile):
```

```
    """parsegff ouputs nested list architecture. List of lists of CDS sharing same
protein of lists sharing the same chromosome.
    e.g. Protein1 has two locus assigned to it:
    [[[Chr1, Protein1, CDS1][Chr1, Protein1, CDS2][Chr2, Protein1, CDS1][Chr2,
Protein1, CDS2]]]
    We'll keep only the first group of each sublevel (i.e. one locus per protein).
    In the case of there is only one locus per protein, we just end up with a
protein of length 1, and sublist_tmp[0] extracts it.
    In NCBI, NC_ taken in priority (locus on a chromosome), then NT_ then NW_ ))
    """
    list_raw_chrom = []
    with open(inputfile, 'r', encoding="ISO-8859-1") as f:
        for line in f:
            if (line.startswith('#') or len(line.split('\t')) < 9):
                pass
            else:
                line = line.strip()
                line = line.split('\t')
                if line[2] == feature:
                    try:
                        seq_name = re.search(seq_name_search_string,
line[8]).group(1)
                        seq_chrom = line[0]
                        seq_start = line[3]
                        seq_stop =  line[4]
                        seq_orientation = line[6]
                        seq_name_w_prefix = seq_prefix+'_'+seq_name
                        list_temp = [seq_prefix,seq_name_w_prefix, seq_chrom,
seq_orientation, seq_start, seq_stop]
                        list_raw_chrom.append(list_temp)
                    #in certain cases, CDS do not have protein product (e.g. in
NCBI gffs "exception=rearrangement required for product")
                    except:
                        pass
                else:
                    pass
    list_groupby_protein_acc = []
    for _, g_same_protein in itertools.groupby(sorted(list_raw_chrom, key =
itemgetter(1)), key = itemgetter(1)):
        list_groupby_protein_acc.append(list(g_same_protein))
    for sublist in list_groupby_protein_acc:
        sublist_tmp = []
        for _, g_same_chrom in itertools.groupby(sorted(sublist, key =
itemgetter(2)), key = itemgetter(2)):
            sublist_tmp.append(list(g_same_chrom))
        yield sublist_tmp[0]#Just the first locus. The sublist is sorted
alphabetically so in the case of NCBI proteins, locus located on DNA with
accessions starting with NC_ (longest, if there is conflict with alternative loci)
are used.
    print('gff file loaded!')

def storechrom(parsed_gff_file):
    y = 0
    for sublist in parsed_gff_file:
```

```
        y += 1
        ls_coordinates = []
        for x in range(0,len(sublist)):
            ls_coordinates.append([int(sublist[x][4]),int(sublist[x][5])])
        yield[sublist[0][0], sublist[0][1]+suffix, sublist[0][2], sublist[0][3],
ls_coordinates] #keep exon information.
    print('{} sequences found in the gff.'.format(str(y)))

def fastafilteredchrom(unfilteredchrom, fastafile):
    ls_accessions = []
    with open(fastafile, 'r') as f:
        for line in f:
            if line.startswith('>'):
                line = line.lstrip('>')
                line = line.rstrip()
                line = line.split(' ')
                ls_accessions.append(line[0])
            else:
                pass
    print('{} sequences found in the fasta file.'.format(len(ls_accessions)))
    y = 0
    for seq_record in unfilteredchrom:
        if seq_record[1] in ls_accessions:
            y += 1
            yield seq_record
            del ls_accessions[ls_accessions.index(seq_record[1])] #reduce the list
size, so that when we iterate through again, the list is smaller
    print('{} sequences from the gff will be retained.'.format(y))


def clusfilteredchrom(unfilteredchrom, clusfile):
    ls_accessions = []
    with open(clusfile, 'r') as f:
        for line in f:
            line = line.rstrip()
            line = line.split('\t')
            species_count = 0
            for x in line [2:]:
                species_count += seq_prefix in x
            if species_count < len(line[2:]): # can only be lower or equal, not
more.
                ls_accessions.extend(line[2:])
            else:
                pass
    ls_accessions_prefix = [accession for accession in ls_accessions if
accession.startswith(seq_prefix)]
    y = 0
    for seq_record in unfilteredchrom:
        if seq_record[1] in ls_accessions_prefix:
            y += 1
            yield seq_record
            del ls_accessions_prefix[ls_accessions_prefix.index(seq_record[1])]
    print('{} sequences in the clus file start with {} and are not species-
specific.'.format(str(y), seq_prefix))
```

```
def test_transcript_overlap(ls_exons_transcript1,ls_exons_transcript2):
    """
    testing the overlap of two transcripts.
    We also check that the overlap is of at least 20 bp as minimum overlap to
avoid exons such as [100,3000] and [3000,400] to be considered overlapping.
    """
    start1 = min(list(itertools.chain.from_iterable(ls_exons_transcript1)))
    end1 = max(list(itertools.chain.from_iterable(ls_exons_transcript1)))
    start2 = min(list(itertools.chain.from_iterable(ls_exons_transcript2)))
    end2 = max(list(itertools.chain.from_iterable(ls_exons_transcript2)))
    length_overlap = min(abs(end1 - start1), abs(end1 - start2), abs(end2 -
start1), abs(end2 - start2))
    overlap = max(start1, start2) > min(end1, end2)
    if overlap and length_overlap > 20:
        return True
    else:
        return False

def test_shared_exon(ls_exons_transcript1,ls_exons_transcript2):
    ls_exons_transcript1_sets = [set(x) for x in ls_exons_transcript1] # transform
exons into sets. this way, even if exons are not in the same order in two
transcripts, they'll be found to be identical (as {a,b} = {b,a})
    ls_exons_transcript2_sets = [set(x) for x in ls_exons_transcript2]
    return any([x in ls_exons_transcript1_sets for x in
ls_exons_transcript2_sets]) #any([]) returns False. The list comprehension looks
if one set of coordinates(exon). If no subset is shared, it returns False.

def test_shared_coordinates(ls_exons_transcript1,ls_exons_transcript2):
    """
    make a list of maxipmum and minimums of exons. If a maximum or a minimum of
the exons is shared, there is an overlap.
    this distinguishes between a simple shared coordinate, where if the start and
stop of an exon are shared it'd consider thgere is an overlap.
    e.g. exons[1,3] and [2,3] are overlapping, but exons [1,3] and [3,4] are not
    """
    transcript1_minls = [min(x) for x in ls_exons_transcript1]
    transcript1_maxls = [max(x) for x in ls_exons_transcript1]
    transcript2_minls = [min(x) for x in ls_exons_transcript2]
    transcript2_maxls = [max(x) for x in ls_exons_transcript2]
    max_shared = any([x in transcript1_maxls for x in transcript2_maxls])
    min_shared = any([x in transcript1_minls for x in transcript2_minls])
    return any([min_shared,max_shared])

def assign_ids_to_pair(AN1, AN2, Dict_AN):
    if AN1 in Dict_AN.keys() and AN2 in Dict_AN.keys():
        if Dict_AN[AN1] == Dict_AN[AN2]: # Check if the two gene IDs are the same
            pass
        elif Dict_AN[AN1] != Dict_AN[AN2]: #if gene IDs are distinct, we'll fuse
the two groups
            for AN, ID in Dict_AN.items():
                if ID == Dict_AN[AN2]:
                    Dict_AN[AN] = Dict_AN[AN1]
                    Dict_AN[AN2] = Dict_AN[AN1]
```

```
    elif AN1 not in Dict_AN.keys() and AN2 not in Dict_AN.keys():
        new_id = max(Dict_AN.values()) + 1
        Dict_AN[AN1] = new_id
        Dict_AN[AN2] = new_id
    elif AN1 not in Dict_AN.keys():
        Dict_AN[AN1] = Dict_AN[AN2]
    elif AN2 not in Dict_AN.keys():
        Dict_AN[AN2] = Dict_AN[AN1]

#almostchrom is the output of storechrom, fastafilteredchrom, clusfilteredchrom
functions.
#Nested list architecture, racapitulates chrom for the first 4 colums. Only a
fifth column is a nested list of exons [[exon1_start, exon1_stop],[exon2_start,
exon2_stop]].
def filterchrombysharedexons(almostchrom):
    tmp_chrom = []
    filteredchrom = []
    d_AN_gene_ids = {'':0}#for later, this'll come in handy
    for _,scaffold in itertools.groupby(sorted(almostchrom, key = itemgetter(2)),
key = itemgetter(2)): #sublist of this groupy are still chrom, but grouped by
scaffold
        sorted_scaffold = sorted(scaffold, key = lambda x: len(x[4]), reverse =
True)
        for transcript1 in sorted_scaffold: #sort the genes within the scaffold
according to their number of exons. This
            accession_transcript1 = transcript1[1]
            transcript1_exon_ls = transcript1[4]
            for transcript2 in
sorted_scaffold[(sorted_scaffold.index(transcript1)+1):]: # loop only starting
from the othergene located after the gene already compared genes before to
everything else. Also, if indexes are out of bounds, this won't raise an error, as
the list will be empty, it won't iterate through it and this do nothing.
                transcript2_exon_ls = transcript2[4]
                if test_transcript_overlap(transcript1_exon_ls,
transcript2_exon_ls) is True:
                    nb_exons_transcript1 = len(transcript1_exon_ls) #total number
of exons = length of list
                    nb_exons_transcript2 = len(transcript2_exon_ls)
                    length_transcript1 =
max(itertools.chain.from_iterable(transcript1_exon_ls)) -
min(itertools.chain.from_iterable(transcript1_exon_ls))   # chain to isolate
coordinates. Max - min = CDS length
                    length_transcript2 =
max(itertools.chain.from_iterable(transcript2_exon_ls)) -
min(itertools.chain.from_iterable(transcript2_exon_ls))
                    accession_transcript2 = transcript2[1]
                    if nb_exons_transcript1 >= 3:
                        if nb_exons_transcript2 >= 3:
                            transcript1_internal_exons_ls =
transcript1_exon_ls[1:-1]
                            transcript2_internal_exons_ls =
transcript2_exon_ls[1:-1]
                            if test_shared_exon(transcript1_internal_exons_ls,
transcript2_internal_exons_ls) is True:
```

```
                                assign_ids_to_pair(accession_transcript1,
accession_transcript2, d_AN_gene_ids)
                        elif nb_exons_transcript2 == 2:
                            if test_shared_coordinates(transcript1_exon_ls,
transcript2_exon_ls) is True:
                                assign_ids_to_pair(accession_transcript1,
accession_transcript2, d_AN_gene_ids)
                        elif nb_exons_transcript2 == 1:
                            ls_test = []
                            for exon in transcript1_exon_ls:
                                ls_test.append(test_transcript_overlap([exon],
[transcript2[4][0]]))
                            if any(ls_test) is True:
                                assign_ids_to_pair(accession_transcript1,
accession_transcript2, d_AN_gene_ids)
                    elif nb_exons_transcript1 == 2:
                        if nb_exons_transcript2 == 2:
                            if test_shared_coordinates(transcript1_exon_ls,
transcript2_exon_ls) is True:
                                assign_ids_to_pair(accession_transcript1,
accession_transcript2, d_AN_gene_ids)
                        elif nb_exons_transcript2 == 1:
                            ls_test = []
                            for exon in transcript1_exon_ls:
                                ls_test.append(test_transcript_overlap([exon],
[transcript2[4][0]]))
                            if any(ls_test) is True:
                                assign_ids_to_pair(accession_transcript1,
accession_transcript2, d_AN_gene_ids)
                    elif nb_exons_transcript1 == 1:
                        if test_transcript_overlap([transcript1[4][0]],
[transcript2[4][0]]) is True:
                            assign_ids_to_pair(accession_transcript1,
accession_transcript2, d_AN_gene_ids)
            if accession_transcript1 not in d_AN_gene_ids.keys():
                new_id = max(d_AN_gene_ids.values()) + 1
                d_AN_gene_ids[accession_transcript1] = new_id
    for record in almostchrom:
        exon_ls = record[4]
        sum_length_of_exons = sum([max(stop - start, start - stop) for start,stop
in exon_ls]) #max is not necessary, but it is yet another check, if exons happen
to be reversed
        modified_sublist = record[0:4]#Prefix, accession, scaffold and strand
        start = min(itertools.chain.from_iterable(exon_ls))
        stop = max(itertools.chain.from_iterable(exon_ls))
        modified_sublist.append(start)
        modified_sublist.append(stop)
        modified_sublist.append(d_AN_gene_ids[record[1]])
        modified_sublist.append(sum_length_of_exons)
        tmp_chrom.append(modified_sublist)
    y = 0
    for _,genes in (itertools.groupby(sorted(tmp_chrom, key = itemgetter(6)), key
= itemgetter(6))): #groupby gene id
        genes = sorted(genes, key = itemgetter(7), reverse = True) # sort eachy
```

```
gene the length of exon sum
        y += 1
        yield [str(x) for x in genes[0][0:6]]
    print('{} sequences have been identified as the longest isoform of their
respective genes.'.format(y))




if __name__ == "__main__"::
    parser = argparse.ArgumentParser(description="Converts gff into chrom, only
one start/stop ouputted per protein .\
        For NCBI, in the case of several loci for one protein script prioritizes
chromosome coordinates before scaffolds.\
        Tested on NCBI, ENSEMBL, JGI, and B. lanceolatum gtf files. \
        For any other GFF file, check your outputted chrom file.")
    parser.add_argument("-gff", "--gff_input", help = "Gff input file.", required
= True)
    parser.add_argument("-p", "--prefix", help = "PREFIX is the PREFIX used in
your fasta file.", required = True)
    parser.add_argument("-f", "--feature", help = "FEATURE is the feature column
value in the GFF file (e.g. exon, mRNA, CDS).", required = True)
    parser.add_argument("-k", "--key", help = "KEY is in the last column of the
GFF and appears as `KEY=SEQUENCENAME` in the input gff.", required = True)
    parser.add_argument("-o", "--output_chrom", help = "Name of output chrom
file.",required = True)
    parser.add_argument("-d", "--delete_redundancy", help = " If two transcripts
share one exon (same start and stop, same scaffold), only the longest is kept.",
action = 'store_true', default = False)
    parser.add_argument("-r", "--ref_filter", help = "OPTIONAL: reference format
for filtering. files are either 'fasta' file or 'clus'. use only with -F", choices
= ['fasta','clus'], default = None)
    parser.add_argument("-F", "--filter_file", help = "OPTIONAL: File to be used
for filtering. Use only with -r", default = None)
    parser.add_argument("-S", "--suffix", help = "suffix found in the accessions
of the fasta but absent of the gff, e.g. _1. Use is your proteins are obtained
from transdecoder.", default = '')
    args = parser.parse_args()
    input_gff = args.gff_input
    seq_prefix = args.prefix
    feature = args.feature
    seq_key = args.key
    output_chrom = args.output_chrom
    suffix = args.suffix
    filter_by_exons = args.delete_redundancy
    #if the chrom file does not recover the search string, this is the one to
modify
    #It is also possible that the KEY you specified is not located on the FEATURE-
positive lines
    seq_name_search_string = (seq_key+'\s*[:="\']*([^,|;"\']+)[;|"\s\n]*')

    unfiltered_chrom = list(storechrom(parsegff(input_gff)))

    none_conditions_filtering = [args.ref_filter == None, args.filter_file ==
```

```
None]
    some_conditions_filtering = [args.ref_filter != None, args.filter_file !=
None]

    #I no filtering by exon is required, we test which filters are applied.
    if filter_by_exons is False:
        with open(output_chrom, 'w') as output:
            if all(none_conditions_filtering) is True:
                    file_to_use = unfiltered_chrom
            elif all(some_conditions_filtering) is True:
                if args.ref_filter == 'fasta':
                    file_to_use = list(fastafilteredchrom(unfiltered_chrom,
args.filter_file))
                elif args.ref_filter == 'clus':
                    file_to_use = list(clusfilteredchrom(unfiltered_chrom,
args.filter_file))
            else:
                print('Unspecified filtering method or file for accession
filtering using fasta/clus file')
            for line in file_to_use:
                output_line = line[0:4]

output_line.append(str(min(itertools.chain.from_iterable(line[4]))))

output_line.append(str(max(itertools.chain.from_iterable(line[4]))))
                output.write('\t'.join(output_line)+'\n')
    elif filter_by_exons is True:
        with open(output_chrom, 'w') as output:
            if all(none_conditions_filtering) is True:
                file_to_use = filterchrombysharedexons(unfiltered_chrom)
            elif all(some_conditions_filtering) is True:
                if args.ref_filter == 'fasta':
                    file_to_use =
filterchrombysharedexons(list(fastafilteredchrom(unfiltered_chrom,
args.filter_file)))
                elif args.ref_filter == 'clus':
                    file_to_use =
filterchrombysharedexons(list(clusfilteredchrom(unfiltered_chrom,
args.filter_file)))
            else:
                print('Unspecified filtering method or file for accession
filtering using fasta/clus file')
            for line in file_to_use:
                output.write('\t'.join(line)+'\n')
```

./02. Data prep/sk_gff_preparation.py

```
#! usr/local/env python3
import sys
import re

"""
Usage: gff_preparation my_file.gff
for SkowalevskiiJGIv3.0.longestTrs.gff3
SACKO names are gene names (i.e. not on CDS lines) We want them to be here.
"""



d_ID = {}
search_string_pacid = 'pacid=(\d+);*'
search_string_protID = 'Name=(\w+);*'

input_gff = sys.argv[1]

with open(input_gff, "r") as f:
        for line in f:
            if line.startswith("#"):
                pass
            else:
                line = line.strip()
                line = line.split('\t')
                if 'mRNA' in line[2]:
                    try:
                        d_value = re.search(search_string_protID,
line[8]).group(1) #prot ID is value
                        d_key = re.search(search_string_pacid, line[8]).group(1)
#pacid is key
                        if d_key not in d_ID.keys():
                            d_ID[d_key] = d_value
                    except:
                        pass #skips line if any re.search is "None"

with open("output.txt", "w") as g:
    with open(input_gff, "r") as f:
        for line in f:
            line = line.strip()
            parsed_line = line.split('\t')
            if len(parsed_line) == 9:
                if parsed_line[2] == 'CDS':
                    line_pacid = re.search(search_string_pacid,
parsed_line[8]).group(1)
                    output_line = line+';protein_id='+d_ID[line_pacid]
                    print(output_line)
                else:
                    output_line = line
                    print(output_line)
```

```
            else:
                print(line)
```

./04. Microsynteny/correct_blocks_coordinates.py

```
#! /usr/bin/env python3

import re
import sys

sys.stderr.write("""This is correct-block coordinates. Takes as input the
"blocks/synt" file ouputted by the microsynteny pipeline,
comma-separated list of chrom files, and outputs a .synt file with the correct
block coordinates.
Usage: correct_blocks_coordinates.py myblocks.synt
mychrom1.chrom,mychrom2.chrom...,mychromn.chrom > corrected_blocks.synt
""")

f_blocks = sys.argv[1]
f_chrom = sys.argv[2].split(',')

d_start = {}
d_stop = {}

for file in f_chrom:
    with open(file, 'r') as f:
        for line in f:
            line = line.strip()
            line = line.split("\t")
            d_start[line[1]] = int(line[4])
            d_stop[line[1]] = int(line[5])

search_string_scaffold = '([^:]+):'


with open(f_blocks, 'r') as g:
    for line in g:
        ls_coordinates = []
        line = line.rstrip()
        line = line.split("\t")
        ls_genes = line[9].split(',')
        scaffold = re.search(search_string_scaffold,line[7]).group(1)
        for gene in ls_genes:
            ls_coordinates.append(d_start[gene])
            ls_coordinates.append(d_stop[gene])
        max_coord = max(ls_coordinates)
        min_coord = min(ls_coordinates)
        output = '\t'.join(line[0:7]) + '\t' +\
                    scaffold + ':' + str(min_coord) + '..' + str(max_coord) + '\t'
```

```
+\
                    str(abs(max_coord - min_coord)) + '\t' +\
                    line[9]
        print(output)
```

./04. Microsynteny/pymakeRandChrom.py

```
#! /usr/bin/env python3

import argparse
import sys
import os
import numpy as np
import pandas as pd

parser = argparse.ArgumentParser(description = "Randomizes the gene order of a
chrom file. Outputs randomized chrom with extension .rand.n in the working
directory")
parser.add_argument('input', type = str, nargs = '?', default = None)
parser.add_argument('-f', '--filetype', choices = ['chrom','list'], help = 'type
of input: either chrom file directly or a newline-separated list of chrom files.
If -f is not used, will try to get filenames from stdin.')
parser.add_argument('-n', '--number', help = 'number of randomized chroms to
generate', default = 1, type = int)
parser.add_argument('-o', '--output_directory', help = 'working directory to use.
If none is provided, randomized chrom files will be written in current
directory.', default = os.getcwd())


args = parser.parse_args()

number_randomizations = args.number + 1
accession_column = 1

filelist = []
if args.filetype == 'list':
    with open(args.input, 'r') as f:
        for filename in f:
            filename = filename.rstrip()
            filelist.append(filename)
elif args.filetype == 'chrom':
    filelist.append(args.input)
elif sys.stdin.isatty() is False:
    for line in sys.stdin:
        line = line.rstrip()
        filelist.append(line)
else:
    print('WARNING! Chrom files to randomize should be provided through a
```

```
filelist, directly in the command, or through stdin.')

for filename in filelist:
    for n in range(1, number_randomizations):
        output_filename = '{}/{}.rand.{}'.format(args.output_directory,
os.path.basename(filename), str(n))
        current_chrom = pd.read_csv(filename, header = None, sep = '\t')
        current_chrom[accession_column] =
np.random.permutation(current_chrom[accession_column])
        with open(output_filename, 'w') as f:
            f.write(current_chrom.to_csv(sep='\t', index=False, header=False))
```

./05. gene density analysis/BlocksByNode.py

```
#!/usr/bin/env python3

import argparse
import sys
import ete3

parser = argparse.ArgumentParser(description = 'Provides reports of block content
of specified nodes')
parser.add_argument('-c',
                    '--clusters_id',
                    help = 'Tsv file, the multi-species clusters of microsyntenic
blocks \
                        output of microsynteny pipeline (makeClusters3.pl).',
                    required = True)
parser.add_argument('-b',
                    '--block_list',
                    help = 'Tsv file, microsyntenic blocks details, output of
microsynteny pipeline\
                    (makeClusters3.pl).',
                    required = True)
parser.add_argument('-s',
                    '--species_tree',
                    help = 'Tree of the PREFIX in the second column of the block
list,\
                        newick format with node names (e.g.\
                        (((D:0.723274,F:0.567784)E:0.067192,
(B:0.279326,H:0.756049)B:0.807788); ).',
                    required = True)
parser.add_argument('-n',
                    '--node_names',
                    help = 'Space separated list of names of nodes of interest
from in the species tree\
                    (e.g. "E B A C".',
                    nargs = '+',
                    required = True)
```

```
parser.add_argument('-m',
                    '--species_threshold',
                    help = "Minimum number n of species per clade for node
inference\
                    (e.g. for novel blocks, requires n species in at least two
ingroups,\
                    and no species in outgroup\
                    for ancestral blocks, n species in one ingroup an n in one
outgroup).\
                    By default, n=2. For ingroup/outgroup of size < n, all species
of said\
                    ingroup/outgroup are required to posess the block\
                    (e.g. if ingroup size of 1, the species is required to
posess.",
                    type = int,
                    default = 2)
parser.add_argument('-r',
                    '--report',
                    help = 'Type of report printed to STDOUT [Default: short] :
\n\
                            - "short": number of blocks per node. \n\
                            - "clusters_list": filters one multi-species block per
line,\
                                cluster IDs (field 1), ancestral/novel nodes of
the block (field 2),\
                                species list (field 3), block_ids (field 4+).\
                                For getting a *.clusters file with only a subset,\
                                pipe SyntByNode output to `cut -f1,4-`.\n\
                            - "blocks_list": blocks within the filtered multi-
species blocks\n\
                            - "tree_ASCII": block count per specified node on an
ASCII tree\n\
                            - "tree_NH": block count per specified node on a
newick tree',
                    choices = ['short', 'clusters_list', 'blocks_list',
'tree_ASCII', 'tree_NH'],
                    default = 'short')
parser.add_argument('-t',
                    '--block_type',
                    help = 'Specify whether you want to report all blocks
("total"),\
                        only the ones inherited from older nodes ("ancestral") or
only "novel" ones.\
                        Multiple options can be specified [Default: total]',
                    choices = ['total','ancestral','novel'],
                    nargs = '+',
                    default = 'total')
args = parser.parse_args()

#Exit the script if you ask for list with more than one type of blocks to report
if len(args.block_type) > 1 and 'list' in args.report:
    sys.stderr.write(f'the {args.report} option can only be used when searching\
        for only one type of blocks! (ONLY total, ONLY ancestral or ONLY
novel.)\n')
```

```
    sys.exit()



#Makes a generator of lists, each list being an ingroup.
def get_ingroups(taxonomic_node, speciestree):
    """
    :param taxonomic node: of whcih the ingroups and outgroups must be determined
    :param species tree: tree to use for determining outgroups and ingroups
    :return: nested list of ingroups, one list per children node
    """
    output_list = []
    for node in speciestree.traverse("preorder"):
        if node.name == taxonomic_node:
            for ingroup in node.children:
                tmp_ig_list = []
                for TaxonName in ingroup.iter_leaf_names(is_leaf_fn=None):
                    tmp_ig_list.append(TaxonName)
                output_list.append(tmp_ig_list)
    return output_list

def get_outgroups(taxonomic_node, speciestree):
    """
    :param taxonomic node: of whcih the ingroups and outgroups must be determined
    :param species tree: tree to use for determining outgroups and ingroups
    :return: list of outgroups
    """
    output_list = []
    cached_tree = speciestree.copy()
    for node in cached_tree.traverse("postorder"):
        if node.name == taxonomic_node and node.is_root() is False:
            node.detach()
        elif node.name == taxonomic_node and node.is_root() is True:
            return output_list
    for taxon_name in cached_tree.iter_leaf_names(is_leaf_fn = None):
        output_list.append(taxon_name)
    return output_list



def blockspeciespairs(blockinfofile):
    """
    :param blockinfofile: file with block info
    :returns: a dict with blocks as keys, species as values
    """
    output_dict = {}
    with open(blockinfofile, 'r') as f:
        for line in f:
            block_id, species, *_ = line.rstrip().split('\t')
            output_dict[block_id] = species
    return output_dict



def blockclusterpairs(clusterinfofile):
    """
```

```
    :param clusterinfofile: file with multi-species block info
    :returns: a dict with block ids as keys, multisp ids as values
    """
    output_dict = {}
    with open(clusterinfofile, 'r') as f:
        for line in f:
            cluster_id, *block_id_ls = line.rstrip().split('\t')
            for block_id in block_id_ls:
                output_dict[block_id] = cluster_id
    return output_dict


class clade:
    """
    A clade instance. As the class is created, we automatically isolate ingroups
and outgroups using the user-provided speciestree.
    'blocks' attributes are lists of the cluster_ids (i.e. multi-species blocks)
    """
    def __init__(clade, name):
        clade.name = name
        clade.ingroups = get_ingroups(clade.name, speciestree)
        clade.outgroups = get_outgroups(clade.name, speciestree)
        clade.total_blocks = []
        clade.novel_blocks = []
        clade.ancestral_blocks = []


def get_block_type(clade, specieslist, n):
    """
    Determines whether a block is ancestral or novel at a node of interest
    :param clade:  clade instance, node of interest
    :param specieslist: list of species posessing the block
    :param n: species threshold. If block novel, needs to be found in 2 ingroups
in at least n species
        if ancestral, block needs to be found in n species in ingroup, n species
in outgroup
    :returns: a list of the block type states, if it's novel or ancestral it's
also part of total
    """
    nb_species_per_ingroup = []
    for ingroup in clade.ingroups:
        species_IG = [x in ingroup for x in specieslist] #bool array, sum of it is
nb of positives since True counts as one.
        if len(ingroup) >= n:
            nb_species = sum(x in ingroup for x in specieslist)
        elif len(ingroup) < n: #in the event that an ingroup is smaller than the
specified threshold
            if sum(x in ingroup for x in specieslist) == len(ingroup):
                nb_species = n # if all the species of the ingroup posess the
pair, threshold is satisfied
            else:
                nb_species = sum(x in ingroup for x in specieslist)
        nb_species_per_ingroup.append(nb_species)
    nb_populated_ingroups = sum(x >= n for x in nb_species_per_ingroup)
```

```
    nb_species_outgroups = sum(x in clade.outgroups for x in specieslist)
    nb_species_ingroups = sum(nb_species_per_ingroup)
    pair_is_novel = [nb_populated_ingroups >= 2 and nb_species_outgroups == 0]
    pair_is_ancestral = [nb_species_ingroups >= n and nb_species_outgroups >= n,
                         nb_populated_ingroups > 0 and nb_species_outgroups ==
len(clade.outgroups)]
    output_ls = []
    if any(pair_is_ancestral):
        return ['ancestral', 'total']
    elif any(pair_is_novel):
        return ['novel', 'total']
    else:
        return []


def get_node_states(clustersIDfile, list_clade_instances, dictspeciesblock):
    with open(clustersIDfile,"r") as f:
        for line in f:
            cluster_id, *block_id_ls = line.rstrip().split('\t')
            species_list = []
            for block_id in block_id_ls:
                species_list.append(dictspeciesblock[block_id])
                set_species_list = set(species_list)
            for i in range(len(list_clade_instances)):
                current_block_type = get_block_type(list_clade_instances[i],
set_species_list, args.species_threshold)
                if 'total' in args.block_type and 'total' in current_block_type:
                     list_clade_instances[i].total_blocks.append(cluster_id)
                if 'novel' in args.block_type and 'novel' in current_block_type:
                     list_clade_instances[i].novel_blocks.append(cluster_id)
                if 'ancestral' in args.block_type and 'ancestral' in
current_block_type:
                     list_clade_instances[i].ancestral_blocks.append(cluster_id)


def print_clusters_list(clustersIDfile,list_clade_instances, dictspeciesblock):
    with open(clustersIDfile,"r") as f:
        for line in f:
            cluster_id, *block_id_ls = line.rstrip().split('\t')
            block_id_str = "\t".join(block_id_ls)
            species_list, node_list = [], []
            for taxon in list_clade_instances:
                keep_block = False
                conditions_to_keep_block = [
                    'total' in args.block_type and cluster_id in
taxon.total_blocks,
                    'ancestral' in args.block_type and cluster_id in
taxon.ancestral_blocks,
                    'novel' in args.block_type and cluster_id in
taxon.novel_blocks]
                if any(conditions_to_keep_block): keep_block = True
                else: keep_block = False
                if keep_block is True:
                    for block_id in block_id_ls:
```

```
                        species_list.append(dictspeciesblock[block_id])
                        set_species_list = set(species_list)
                        node_list.append(taxon.name)
            if node_list != []:
                node_ls_str = ','.join(set(node_list))

print(f'{cluster_id}\t{",".join(set(node_list))}\t{",".join(set_species_list)}\t{b
lock_id_str}')


def print_blocks_list(blocksIDfile,list_clade_instances, dictblockclusters):
    with open(blocksIDfile, 'r') as f:
        for line in f:
            block_id, line_rest = line.rstrip().split('\t', maxsplit = 1)
            node_list = []
            try:
                cluster_id = dictblockclusters[block_id]
                for taxon in list_clade_instances:
                    if 'total'in args.block_type and cluster_id in
taxon.total_blocks:
                        node_list.append(taxon.name)
                    if 'ancestral' in args.block_type and cluster_id in
taxon.ancestral_blocks:
                        node_list.append(taxon.name)
                    if 'novel' in args.block_type and cluster_id in
taxon.novel_blocks:
                        node_list.append(taxon.name)
                if node_list != []:
                    print(f'{cluster_id}\t{block_id}\t{line_rest}')
            except KeyError:
                pass


def print_short(list_clade_instances):
    ls_taxons = []
    for taxon in list_clade_instances:
        ls_taxons.append(taxon.name)
    print('\t'.join(['taxon', 'blocktype', 'count']))
    for taxon in list_clade_instances:
        if 'total' in args.block_type:
            print(f'{taxon.name}\ttotal\t{len(taxon.total_blocks)}')
        if 'ancestral' in args.block_type:
            print(f'{taxon.name}\tancestral\t{len(taxon.ancestral_blocks)}')
        if 'novel' in args.block_type:
            print(f'{taxon.name}\tnovel\t{len(taxon.novel_blocks)}')


def print_tree (list_clade_instances, species_tree):
    cached_tree = speciestree.copy()
    ls_taxons = []
    for taxon in list_clade_instances:
        ls_taxons.append(taxon.name)
    for node in cached_tree.traverse("preorder"):
        if node.name in ls_taxons:
```

```
            i = getattr(ls_taxons, 'index')(node.name) #ls_taxons and
ls_clade_instances are essentially the same list order. We get the indes from
ls_taxons, and use this index to extract class info from ls_clade_instances
            node_clade = list_clade_instances[i]
            if 'total' in args.block_type:
                node.name = f'{node.name}_{len(node_clade.total_blocks)}'
            if 'ancestral' in args.block_type:
                node.name = f'{node.name}_{len(node_clade.ancestral_blocks)}'
            if 'novel' in args.block_type:
                node.name = f'{node.name}_{len(node_clade.novel_blocks)}'
    if args.report == 'tree_ASCII':
        print(cached_tree.get_ascii(show_internal=True))
    if args.report == 'tree_NH':
        print(cached_tree.write(format = 1))


#import the speciestree
speciestree = ete3.Tree(args.species_tree, format=1)

#argument check. if one of the node names is in root and asks for novel blocks,
stop the script
if speciestree.get_tree_root().name in args.node_names:
    raise AttributeError(f'It is not possible to determine novel blocks\
        of {speciestree.get_tree_root().name }, as it is the root node')



#create useful dictionaries for the following functions
d_block_species = blockspeciespairs(args.block_list)
d_block_clusters = blockclusterpairs(args.clusters_id)

#modify args.node_names into a list of clade instances.
for x in range(0,len(args.node_names)):
    args.node_names[x] = clade(args.node_names[x])

# This modifes clade instances within the list. Adding lists of cluster to the
attributes created as empty lists at the __init__ of the clade instance
get_node_states(args.clusters_id, args.node_names, d_block_species)

if 'tree' in args.report:
    print_tree(args.node_names, speciestree)
    sys.stderr.write(f'Nodes are as follows: {"_".join(args.block_type)}\n')
elif args.report == 'short':
    print_short(args.node_names)
elif 'list' in args.report:
    sys.stderr.write(f'The {", ".join(args.block_type)} blocks are printed to
stdout.\n')
    if args.report == 'clusters_list':
        print_clusters_list(args.clusters_id, args.node_names, d_block_species)
    else:
        print_blocks_list(args.block_list, args.node_names, d_block_clusters)
```

./05. gene density analysis/block_correlation analysis.py

```
#! /usr/bin/env python3

import pandas as pd
import numpy as np
import scipy.stats
import itertools
import collections
import argparse


parser = argparse.ArgumentParser(description = 'takes a tidy dataframe as input,
made with make_tidy_density_df.py')
parser.add_argument('-t', '--results_df', help = 'tidy dataframe, one observation
by line', required = True)
parser.add_argument('-s', '--full_synt_file', help = 'original (unfiltered synt
file)', required = True)
parser.add_argument('-e', '--expression_data', help = 'full tpm table')
parser.add_argument('-p', '--prefix', help = 'prefix to add to accessions in
expression data')
parser.add_argument('-o', '--output', help = 'output prefix', required = True)

args = parser.parse_args()


def load_expr(expr):
    """
    load expression data
    unexpressed genes (0 tpms in all stages) are deleted
    :param expr: expression table, first line is header, firts column is accession
    second column length, rest of fields are expression by stage
    """
    output_dict = {}
    with open(expr, 'r') as f:
        header = f.readline()
        for line in f:
            transcript_id, _, *tpms = line.rstrip().split() #second column is
length
            tpms = np.array([np.float64(x) for x in tpms])
            if args.prefix != '':
                transcript_id = f'{args.prefix}_{transcript_id}'
            if tpms.max() == 0:
                pass
            else:
                output_dict[transcript_id] = tpms
    return output_dict


def block_correlation(acc_str):
    """
```

```
    computes block correlation from block_id
    uses exp_dict(names: numpy 1D arrays with expression per stage)
    and block_dict(block_id: list of expressed genes of the block)
    :param myid: block_id
    :returns: block correlation
    """
    acc_ls = acc_str.split(',')
    corrs = []
    to_remove = []
    for gene in acc_ls:
        if exp_dict.get(gene) is None:
            to_remove.append(gene)
    acc_ls = list(set(acc_ls) - set(to_remove))
    if len(acc_ls) < 3:
        return np.nan
    else:
        for genea, geneb in itertools.combinations(acc_ls, 2):
            exp_genea = exp_dict[genea]
            exp_geneb = exp_dict[geneb]
            corr = scipy.stats.spearmanr(exp_genea, exp_geneb).correlation
            if corr == 1:
                corr = np.float64(0.9999999999999999) #arctanh(1) = inf, mean of
any array with one infinity value is infinity, and tanh(inf) = 1. This avoids
block correlation of one when one pair has a value of 1.
            if corr == -1:
                corr = np.float64(-0.9999999999999999)
            corrs.append(corr)
        corrs = np.array(corrs)
        block_corr = np.tanh(np.mean(np.arctanh(corrs)))
        return block_corr


"""

tmp_args = collections.namedtuple('tmp_args', ['results_df', 'full_synt_file',
'expression_data', 'prefix', 'output'])

args =
tmp_args('../../02_REDUX_gene_density_analysis/density_whole_genome/key_nodes.tidy
df.csv',

'/scratch/robert/2019_microsynteny_size_constraints/01_microsynteny/chrom_of/5.blo
cks.3.syn_corrected.synt',
                 '../CALMI/CALMI_transcript_tpms_all_samples.tsv',
                 'CALMI',
                 'density_blockcorrelation')


"""

order = ['Vertebrate', 'Tunicate', 'Cephalochordate', 'Ambulacrarian',
'Lophotrochozoan', 'Ecdysozoan', 'Acoel', 'Cnidarian', 'Placozoan', 'Ctenophore',
'Poriferan', 'Metazoa_outgroup']
species_dict = {'Vertebrate' :
```

```
['HOMSA','MUSMU','CHEMY','GALGA','XENTR','LATCH','MAYZE','HIPCO','DANRE','LEPOC','
CALMI'],
                'Tunicate': ['CIOIN'],
                'Cephalochordate' : ['BRALA'],
                'Ambulacrarian' : ['SACKO','PTYFL','STRPU','ACAPL'],
                'Lophotrochozoan' : ['CAPTE',
'EUPSC','LOTGI','MIZYE','CRAGI','HELRO','ADIVA','LINAN','SCHME'],
                'Ecdysozoan' :
['DROME','ANOGA','TRICA','DAPPU','STRMA','IXOSC','PARTE','CAEEL'],
                'Acoel' : ['HOFMI'],
                'Cnidarian' : ['NEMVE','EXAPA','ACRMI','HYDVU','CLYHE','AURAU'],
                'Placozoan' : ['HOIHO','TRIAD'],
                'Ctenophore' : ['PLEBA','MNELE'],
                'Poriferan' : ['SYCCI','AMPQU'],
                'Metazoa_outgroup' : ['SALRO','CAPOW'],
}


order_sp = [species for taxon in order for species in species_dict[taxon]]


results_df = pd.read_csv(args.results_df)


exp_dict = load_expr(args.expression_data)

species_df_obs = results_df.query("random == 'observed' & species ==
@args.prefix")
species_df_obs['block_corr'] = species_df_obs['acc_ls'].map(lambda x:
block_correlation(x))
species_df_obs = species_df_obs.dropna()

blocks_to_keep = species_df_obs.block_id.tolist()

species_df_rand = results_df.query("random == 'random' & species == @args.prefix &
block_id in @blocks_to_keep" )
species_df_rand['block_corr'] = species_df_rand['acc_ls'].map(lambda x:
block_correlation(x))
species_df_rand = species_df_rand.dropna()

species_df = pd.concat([species_df_obs, species_df_rand])

species_df.to_csv(args.output, index = False)
```

./05. gene density analysis/case_studies_density_surround.py

```
import argparse
```

```
import collections
import glob
import itertools
import numpy as np
import os
import pandas as pd
import sys

parser = argparse.ArgumentParser(description = """
Each block is decomposed into N bins (bin size = 1/N block width)
the upstream and downstream 2N bins are also included
Gene density is calculated for 5N bins
Outputs tidy df where each line is a bin
block_id, multi_sp, species obs and bin location are outputted
""")
parser.add_argument('-s', '--synt_file', help = "synt file where to pick the
blocks from", type = str, required = True)
parser.add_argument('-r', '--rand_synt_file', help = "randomly sampled blocks",
type = str, required = True)
parser.add_argument('-m', '--multi_sp', help = 'Multi species block (total), used
to get multi_sp ID', type = str, required = True)
parser.add_argument('-g', '--genome_folder', help = 'folder where genome files are
located, prefix in the name dot separated. e.g. EUPSC.lachesis201904.genome.fa,
CRAGI.NCBIgenome.fasta',type = str, required = True)
parser.add_argument('-c', '--chrom_folder', help = 'folder where chrom files are
located, files should have the *chrom extension', required = True)
parser.add_argument('-n', '--n_bins', help = 'number of bins by block', default =
5, type = int)
parser.add_argument('-o', '--output', help = 'output name')
args = parser.parse_args()

#tmp_args = collections.namedtuple('tmp_args', 'synt_file rand_synt_file multi_sp
genome_folder chrom_folder n_bins output')
#args =
tmp_args("/scratch/robert/2019_microsynteny_size_constraints/03_REDUX_density_HOX_
WNT/wnt/wnt.syn.synt",
#
"/scratch/robert/2019_microsynteny_size_constraints/03_REDUX_density_HOX_WNT/wnt/w
nt.syn.random.synt",
#
"/scratch/robert/2019_microsynteny_size_constraints/03_REDUX_density_HOX_WNT/wnt/w
nt.syn.clusters",
#
"/scratch/robert/2019_microsynteny_size_constraints/02_REDUX_gene_density_analysis
/genomes/",
#
"/scratch/robert/2019_microsynteny_size_constraints/01_microsynteny/chrom/",
#                 5,
#                 "test")

def parse_fasta(handle):
    """
    parses fasta file
    :param handle: filehandle of a fasta file
```

```
    :return: a generator, pairs of header/sequence items. Sequence devoid of
newline characters.
    """
    fasta_iter = (list(g) for _,g in itertools.groupby(handle, lambda l:
l.startswith('>')))
    for header_group, sequence_group in zip(*[fasta_iter]*2):
        header_string = ''.join(header_group).lstrip('>').rstrip()
        header = ''.join(header_group).lstrip('>').rstrip().split()[0]
        seq = ''.join([line.rstrip() for line in sequence_group])
        yield header, seq


def get_ranges(coords_string, nb_bins, species, lengths_dict):
    """
    Return pairs of values start end of bins, given a block, and a nb of bins
    :param coords_string: coords as written in synt file (scaffold:start...end)
    :param nb_bins: nb of bins the block should be split into
    :param species: prefix of species where the block is found
    :param lengths_dict: nested length dict where k,k,v are prefix, scaffold,
length
    :returns: a list of lists of length 4,
    start end of the bins that can be sampled (won't sample outside of block
boundaries),
    and the normalized coords so that 0 = block start, 1 = block end
    """
    output = []
    bin_factors = [round(x, 1) for x in np.linspace(-2, 3, num = nb_bins * 5 + 1)]
    bin_factors = zip(bin_factors, bin_factors[1:])
    chromosome, start, end = coords_string.replace('..', ':').split(':')
    start = int(start)
    end = int(end)
    len_block = end - start
    bin_size = round(len_block / nb_bins)
    bin_lower = start - 2 * len_block
    bin_upper = end + 2 * len_block
    len_chrom = lengths_dict[species][chromosome]
    bin_breaks = [i for i in range(bin_lower, bin_upper, bin_size)] + [bin_upper]
    bin_breaks = zip(bin_breaks, bin_breaks[1:])
    bin_bounds = [itertools.chain.from_iterable((n, bp)) for n, bp in
zip(bin_factors, bin_breaks) if (any([x < 0 for x in bp]) is False and any([x >
len_chrom for x in bp]) is False)]
    return bin_bounds


print('Loading genome scaffold lengths...', file = sys.stderr)
genomes_ls = [file for file in glob.glob(f'{args.genome_folder}/*') if 'genome' in
file]
genomes_lengths_dict = {}
for genome_file in genomes_ls:
    prefix = os.path.basename(genome_file).split('.')[0]
    print(prefix)
    with open(genome_file, 'r') as f:
        lengths_d = {header: len(sequence) for header, sequence in parse_fasta(f)}
        genomes_lengths_dict[prefix] = lengths_d
```

```
print('Done!\n', file = sys.stderr)

print('Loading chromfiles...', file = sys.stderr)

chrom_ls = [file for file in glob.glob(f'{args.chrom_folder}/*chrom')]
chrom_dict = {}
total_density_dict = {}
total_lengths_dict = {}
for chrom in chrom_ls:
    prefix = os.path.basename(chrom).split('.')[0]
    chrom_dict[prefix] = pd.read_csv(chrom, sep = '\t', names = ['prefix',
'accession', 'chromosome', 'strand', 'start', 'end'])
    total_nb_genes = len(chrom_dict[prefix])
    total_length = sum(genomes_lengths_dict[prefix].values())
    total_density_dict[prefix] = total_nb_genes/total_length
    total_lengths_dict[prefix] = total_length

print(f'Done!\n', file = sys.stderr)

print('Loading multi_sp...', file = sys.stderr)
multi_sp_dict = {}
with open(args.multi_sp, 'r') as f:
    for line in f:
        multi_sp, *block_id_ls = line.rstrip().split('\t')
        multi_sp_dict.update({block_id:multi_sp for block_id in block_id_ls})

print(f'Done!\n', file = sys.stderr)

#bin_loc is a factor, N bins of normalized bp size 1/n
header = ['block_id', 'iteration', 'multi_sp', 'species', 'obs', 'bin_location',
'breaks_location', 'density']
results = []
for file, observed in [[args.synt_file, 'observed'], [args.rand_synt_file,
'random']]:
    with open(file, 'r') as f:
        for line in f:
            block_id, species, _, _, _, _, _, coords, *_ = line =
line.rstrip().split('\t')
            print(block_id)
            block_chromosome = coords.split(':')[0]
            iteration = 0
            multi_sp = multi_sp_dict.get(block_id)
            if multi_sp == None:
                block_id, iteration = block_id.split('.')
                multi_sp = multi_sp_dict.get(block_id)
            chrom_df = chrom_dict[species]
            tmp_df = chrom_df.query(('chromosome == @block_chromosome'))
            for start_factor, end_factor, start_breaks, end_breaks in \
                get_ranges(coords, args.n_bins, species, genomes_lengths_dict):
                factor_location = round((start_factor + end_factor) / 2, 1)
                break_location = f'[{start_breaks}:{end_breaks})'
                end_breaks = end_breaks -1
                overlapping_genes_df = tmp_df.query('@start_breaks <= start <
```

```
@end_breaks |@start_breaks < end <= @end_breaks| start <= @start_breaks <
end|start < @end_breaks <= end')
                density = len(overlapping_genes_df) / (end_breaks - start_breaks)
                results.append([block_id,
                                iteration,
                                multi_sp,
                                species,
                                observed,
                                factor_location,
                                break_location,
                                density])


df = pd.DataFrame(results, columns = header)

df.to_csv(args.output, sep = '\t', index = False)
```

./05. gene density analysis/figure2_prep_data_scatterplots.py

```
#! /usr/bin/env python3

import pandas as pd
import numpy as np
import seaborn as sns
import matplotlib.pyplot as plt
import scipy.stats
import itertools



#plot preferences, move onto other file
order = ['Vertebrate', 'Tunicate', 'Cephalochordate', 'Ambulacrarian',
'Lophotrochozoan', 'Ecdysozoan', 'Acoel', 'Cnidarian', 'Placozoan', 'Ctenophore',
'Poriferan', 'Metazoa_outgroup']
species_dict = {'Vertebrate' :
['HOMSA','MUSMU','CHEMY','GALGA','XENTR','LATCH','MAYZE','HIPCO','DANRE','LEPOC','
CALMI'],
                'Tunicate': ['CIOIN'],
                'Cephalochordate' : ['BRALA'],
                'Ambulacrarian' : ['SACKO','PTYFL','STRPU','ACAPL'],
                'Lophotrochozoan' : ['CAPTE',
'EUPSC','LOTGI','MIZYE','CRAGI','HELRO','ADIVA','LINAN','SCHME'],
                'Ecdysozoan' :
['DROME','ANOGA','TRICA','DAPPU','STRMA','IXOSC','PARTE','CAEEL'],
                'Acoel' : ['HOFMI'],
                'Cnidarian' : ['NEMVE','EXAPA','ACRMI','HYDVU','CLYHE','AURAU'],
                'Placozoan' : ['HOIHO','TRIAD'],
                'Ctenophore' : ['PLEBA','MNELE'],
```

```
                'Poriferan' : ['SYCCI','AMPQU'],
                'Metazoa_outgroup' : ['SALRO','CAPOW'],
}


results_df = pd.read_csv('key_nodes.tidydf.csv')

results_df_fig2 = results_df.groupby(['multi_sp', 'random', 'taxon',
'node']).median() # median of all the values
results_df_fig2.reset_index(inplace = True)
del results_df_fig2['block_id']
del results_df_fig2['iteration']
del results_df_fig2['density']
del results_df_fig2['total_density']


df = results_df_fig2.copy()
df = df.pivot_table(index=['multi_sp', 'taxon', 'node'],
                    columns='random',
                    values=['density_ratio'])
df.columns = ['obs_density_ratio', 'rand_density_ratio']
df.reset_index(inplace = True)
df['deviation_to_random'] = (df['obs_density_ratio'] - df['rand_density_ratio']) /
df['obs_density_ratio']


df2 = df.copy()
df2['order'] = df2['taxon'].map(lambda x: order.index(x))
df2 = df2.pivot_table(index=['multi_sp', 'node'],
                      columns='taxon',
                      values=['deviation_to_random'])
df2.columns = [x[1] for x in df2.columns]
df2.reset_index(inplace = True)

order_taxons = [tax for tax in order if tax in df.taxon.to_list()]
order_columns = [x for x in df2.columns if x not in order_taxons] + order_taxons

df2 = df2[order_columns]

df2.to_csv('raw_data_scatter.csv', index = False)
```

./05. gene density analysis/make_tidy_density_df.py

```
#! /usr/bin/env python3

import argparse
import collections
import glob
import itertools
import numpy as np
```

```
import os
import pandas as pd
import sys

parser = argparse.ArgumentParser(description = """
makes a single tidy df with gene densities by block (one line per iteration)
finds para/not para blocks. random blocks para attributes are inferrend from the
observed counterpart
provides two lists off accs. acc_ls is syntenic blocks, all_acc_ls also includes
intervening genes
""")
parser.add_argument('-s', '--synt_folder', help = """folder where synt files,
filename should start by nodename, dot-separated, extension should be *synt.
random should appear in the name.
                                                    e.g. Bilateria.novel.synt, and
the random blocks Bilateria.novel.random.synt""", default = os.getcwd())
parser.add_argument('-g', '--genome_folder', help = 'folder where genome files are
located, prefix in the name dot separated. e.g. EUPSC.lachesis201904.genome.fa,
CRAGI.NCBIgenome.fasta', required = True)
parser.add_argument('-c', '--chrom_folder', help = 'folder where chrom files are
located, files should have the *chrom extension', required = True)
parser.add_argument('-m', '--multi_sp', help = 'Multi species block (total), used
to get multi_sp ID', required = True)
parser.add_argument('-og', '--ortho', help = 'orthology file, clus format',
required = True)
parser.add_argument('-o', '--output', help = 'output prefix', default = 'density')
args = parser.parse_args()


#tmp_args = collections.namedtuple('tmp_args', ['synt_folder', 'genome_folder',
'chrom_folder', 'multi_sp', 'ortho', 'output'])
#args = tmp_args(os.getcwd(), '../../genomes/', '../../../01_microsynteny/chrom/',
'../../../01_microsynteny/chrom_of/5.blocks.3.syn.clusters',
'../../../01_microsynteny/Orthofinder.clus', 'key_nodes_w_consecutive_pairs')

def parse_fasta(handle):
    """
    parses fasta file
    :param handle: filehandle of a fasta file
    :return: a generator, pairs of header/sequence items. Sequence devoid of
newline characters.
    """
    fasta_iter = (list(g) for _,g in itertools.groupby(handle, lambda l:
l.startswith('>')))
    for header_group, sequence_group in zip(*[fasta_iter]*2):
        header_string = ''.join(header_group).lstrip('>').rstrip()
        header = ''.join(header_group).lstrip('>').rstrip().split()[0]
        seq = ''.join([line.rstrip() for line in sequence_group])
        yield header, seq



def parse_blocks(syntfile):
    """
    Get coordinates of all the blocks in a file
```

```
    :param syntfile : synt file
    :return: dictionary, keys are species prefixes, values are a list of
coordinates
    """
    block_dict = {}
    block = collections.namedtuple('block',
                                    ['id','chromosome', 'start', 'end', 'acc_ls'])
    with open(syntfile, 'r') as f:
        for line in f:
            line = line.rstrip().split('\t')
            block_id, species, _, _, _, _, _, coords, _, *rest = line #works with
output of pick_random_blocks
            acc_ls = rest[0] #acc_ls is the first item there
            chromosome, start, stop = coords.replace('..', ':').split(':')
            if block_dict.get(species, False) is False:
                block_dict[species] = [block(block_id, chromosome, int(start),
int(stop), acc_ls)]
            else:
                block_dict[species].append(block(block_id, chromosome, int(start),
int(stop), acc_ls))
    return block_dict


def load_ortho(ortho):
    """
    load orthology
    :param ortho: Orthofinder output, clus format
    :returns: dict, acc as keys, OG as values
    """
    output_dict = {}
    with open(ortho, 'r') as f:
        for line in f:
            og, _, *acc_ls = line.rstrip().split('\t')
            for acc in acc_ls:
                output_dict[acc] = og
    return output_dict


def block_para(acc_ls):
    """
    Determines whether a block is para or not para based on OG composition
    if more than 50 % of genes belong to same OGm it's para
    :param myid: acc_ls, string with comma-separated accessions
    :returns: para/not_para
    """
    acc_ls = acc_ls.split(',')
    og_ls = [ortho_dict[acc] for acc in acc_ls]
    counts = []
    for og in set(og_ls):
        counts.append(og_ls.count(og))
    para_conds = [count > (0.4 * len(og_ls)) for count in counts]
    if any(para_conds) is True:
        return 'para'
    else:
```

```
        return 'not_para'


def median_consecutivedist(complete_acc_ls):
    """
    when provided a list of accessions, calculates the mean intergenic distance
between genes of the list
    """
    dist_ls = collections.deque()
    acc_ls = complete_acc_ls.split(",")
    prefix = acc_ls[0].split('_')[0]
    df_coords = chrom_dict[prefix].query('accession in @acc_ls').sort_values(by =
"start")
    acc_ls = df_coords.accession.tolist()
    start_ls = df_coords.start.tolist()
    end_ls = df_coords.end.tolist()
    for i in range(len(acc_ls) -1):
        j = i+1
        acc1,end_1 = acc_ls[i], end_ls[i]
        acc2, start2 = acc_ls[j], start_ls[j]
        intergenic_dist = start2 - end_1
        if intergenic_dist < 0: intergenic_dist = 0
        dist_ls.append(intergenic_dist)
    return statistics.median(dist_ls)




species_dict = {'Vertebrate' :
['HOMSA','MUSMU','CHEMY','GALGA','XENTR','LATCH','MAYZE','HIPCO','DANRE','LEPOC','
CALMI'],
                'Tunicate': ['CIOIN'],
                'Cephalochordate' : ['BRALA'],
                'Ambulacrarian' : ['SACKO','PTYFL','STRPU','ACAPL'],
                'Lophotrochozoan' : ['CAPTE',
'EUPSC','LOTGI','MIZYE','CRAGI','HELRO','ADIVA','LINAN','SCHME'],
                'Ecdysozoan' :
['DROME','ANOGA','TRICA','DAPPU','STRMA','IXOSC','PARTE','CAEEL'],
                'Acoel' : ['HOFMI'],
                'Cnidarian' : ['NEMVE','EXAPA','ACRMI','HYDVU','CLYHE','AURAU'],
                'Placozoan' : ['HOIHO','TRIAD'],
                'Ctenophore' : ['PLEBA','MNELE'],
                'Poriferan' : ['SYCCI','AMPQU'],
                'Metazoa_outgroup' : ['SALRO','CAPOW'],
}

species_dict_r = {species: taxon for taxon,species_ls in species_dict.items() for
species in species_ls}


synt_obs_ls = [file for file in glob.glob(f'{args.synt_folder}/*synt') if 'random'
not in file]
synt_rand_ls = [file for file in glob.glob(f'{args.synt_folder}/*synt') if
'random' in file]
```

```
nodes_obs_dict = {os.path.basename(syntfile).split('.')[0]: parse_blocks(syntfile)
for syntfile in synt_obs_ls}
nodes_rand_dict = {os.path.basename(syntfile).split('.')[0]:
parse_blocks(syntfile) for syntfile in synt_rand_ls}


print('Loading genome info...', file = sys.stderr)

genomes_ls = [file for file in glob.glob(f'{args.genome_folder}/*') if 'genome' in
file]
genomes_lengths_dict = {}
for genome_file in genomes_ls:
    prefix = os.path.basename(genome_file).split('.')[0]
    print(prefix)
    with open(genome_file, 'r') as f:
        lengths_d = {header: len(sequence) for header, sequence in parse_fasta(f)}
        genomes_lengths_dict[prefix] = lengths_d

print('Done!\n', file = sys.stderr)


print('Processing chrom...', file = sys.stderr)

chrom_ls = [file for file in glob.glob(f'{args.chrom_folder}/*chrom')]
chrom_dict = {}
total_density_dict = {}
total_lengths_dict = {}
for chrom in chrom_ls:
    prefix = os.path.basename(chrom).split('.')[0]
    chrom_dict[prefix] = pd.read_csv(chrom, sep = '\t', names = ['prefix',
'accession', 'chromosome', 'strand', 'start', 'end'])
    total_nb_genes = len(chrom_dict[prefix])
    total_length = sum(genomes_lengths_dict[prefix].values())
    total_density_dict[prefix] = total_nb_genes/total_length
    total_lengths_dict[prefix] = total_length

print(f'Done!\n', file = sys.stderr)

print('Processing multi_sp...', file = sys.stderr)
multi_sp_dict = {}
with open(args.multi_sp, 'r') as f:
    for line in f:
        multi_sp, *block_id_ls = line.rstrip().split('\t')
        multi_sp_dict.update({block_id:multi_sp for block_id in block_id_ls})

print(f'Done!\n', file = sys.stderr)

print('Processing orthology...', file = sys.stderr)
ortho_dict = load_ortho(args.ortho)
print(f'Done!\n', file = sys.stderr)


results_ls = collections.deque()
for node in nodes_obs_dict.keys():
```

```
    for species in nodes_obs_dict[node].keys():
        taxon = species_dict_r[species]
        chrom_df = chrom_dict[species]
        print('obs', node, species)
        for block in nodes_obs_dict[node][species]:
            tmp_df = chrom_df.query(('chromosome == @block.chromosome'))
            overlapping_genes_df = tmp_df.query('@block.start <= start <
@block.end |@block.start < end <= @block.end| start <= @block.start < end|start <
@block.end <= end')
            density = len(overlapping_genes_df) / (block.end - block.start)
            all_acc_ls = overlapping_genes_df.accession.tolist()
            all_acc_ls = ",".join(all_acc_ls)
            iteration = 1
            results_ls.append([node, taxon, species, 'observed', block.id,
iteration, density, block.acc_ls, all_acc_ls])


for node in nodes_rand_dict.keys():
    for species in nodes_rand_dict[node].keys():
        taxon = species_dict_r[species]
        chrom_df = chrom_dict[species]
        print('rand', node, species)
        for block in nodes_rand_dict[node][species]:
            tmp_df = chrom_df.query(('chromosome == @block.chromosome'))
            overlapping_genes_df = tmp_df.query('@block.start <= start <
@block.end |@block.start < end <= @block.end| start <= @block.start < end|start <
@block.end <= end')
            density = len(overlapping_genes_df) / (block.end - block.start)
            all_acc_ls = overlapping_genes_df.accession.tolist()
            all_acc_ls = ",".join(all_acc_ls)
            block_id, iteration = block.id.split('.')
            results_ls.append([node, taxon, species, 'random', block_id,
iteration, density, block.acc_ls, all_acc_ls])


results_df = pd.DataFrame(results_ls, columns =  ['node', 'taxon', 'species',
'random', 'block_id', 'iteration', 'density', 'acc_ls', 'all_acc_ls'])

#df for having rand and obs on the same plots
results_df['total_density'] = results_df['species'].map(total_density_dict)
results_df['total_genome_length'] = results_df['species'].map(total_lengths_dict)
results_df['density_ratio'] = results_df['density'] / results_df['total_density']
results_df['multi_sp'] = results_df['block_id'].map(multi_sp_dict)

#Assign para based on which obs the random block was sampled from
obs_df = results_df.query("random == 'observed'")
obs_df['para'] = obs_df['acc_ls'].map(lambda x: block_para(x))
para_dict = dict(zip(obs_df['block_id'], obs_df['para']))

results_df['para'] = results_df['block_id'].map(para_dict)
results_df['median_dist_pair'] = results_df['all_acc_ls'].map(lambda x:
median_consecutivedist(x))
results_df['median_dist_pair_norm'] = results_df['median_dist_pair'] /
results_df['total_genome_length']
```

```
results_df.to_csv(f'{args.output}.tidydf.csv', index = False)
```

./05. gene density analysis/MUSMU_reformat.py

```
#! /usr/bin/env python3

import re
import sys
import pandas as pd
import glob

"""
Usage:
MUSMU_reformat.py protlist expression_data MUSMU_gff
"""

if len(4 > sys.argv > 1):
    _, protein_file, gff_file = sys.argv
else:
    raise ArgumentError("""
        Usage:
        MUSMU_reformat.py protlist expression_data MUSMU_gff
        protlist: list of neyline separated protein accessions
        expression data: mouse encode file to use
        gff file:a gff file
        """)


#load proteins
protein_ls = {}
with open(protein_file, 'r') as f:
    for line in f:
        line = line.rstrip()
        prot = line.split('_', 1)[1]
        protein_ls[prot] = ''

#load gff
accessions_dict = {}
re_transcript = 'Parent'+'\s*[:="\']*([^,|;"\']+)[;|"\s\n]*'
re_protein = 'protein_id'+'\s*[:="\']*([^,|;"\']+)[;|"\s\n]*'
with open(gff_file) as f:
    for line in f:
        if (line.startswith('#') or len(line.split('\t')) < 9):
            pass
        else:
            fields = line.rstrip().split('\t')
```

```
            _, _, feature, *_,comments = fields
            if feature == 'CDS':
                if 'exception' not in comments:
                    protein_id = re.search(re_protein, comments).group(1)
                    if protein_ls.get(protein_id) != None:
                        transcript_id = re.search(re_transcript,
comments).group(1)
                        transcript_id = transcript_id.split('-')[1].split('.')[0]
                        accessions_dict[transcript_id] = protein_id
                        del protein_ls[protein_id]

results =  {}
for file in glob.glob('19-tissues-expr/*.expr'):
    basename = os.path.basename(file)
    with open(file, 'r') as f:
        _ = f.readline()
        for line in f:
            line = line.rstrip().split()
            gene_id , *_, FPKM, _, _ = line
            protein_id = accessions_dict.get(gene_id)
            if protein_id != None:
                if results.get(basename) == None:
                    results[basename] = {protein_id:FPKM}
                else:
                    results[basename][protein_id] = FPKM

df = pd.DataFrame(results)
df = df.fillna(0.0)


stages = {'bone_marrow' : ['boneMarrow1-zy24.gene.expr', 'boneMarrow2-
zy26.gene.expr'],
'E14.5_brain': ['brain-E14.5-1.expr', 'brain-E14.5-2.expr'],
'cerebellum': ['cerebellum1-zy21.gene.expr', 'cerebellum2-zy22.gene.expr'],
'cortex': ['cortex1-zy13.gene.expr', 'cortex2-zy14.gene.expr'],
'E14.5_heart': ['heart-E14.5-1.expr', 'heart-E14.5-2.expr'],
'heart':['heart1-zy6.gene.expr', 'heart2-zy7.gene.expr'],
'intestine':['intestine-2.expr', 'intestine-3.expr'],
'kidney':['kidney1-zy15.gene.expr', 'kidney2-zy16.gene.expr'],
'E14.5_limb': ['limb-E14.5-1.expr', 'limb-E14.5-2.expr'],
'E14.5-liver':['liver-E14.5-1.expr', 'liver-E14.5-2.expr'],
'liver':['liver1-zy4.gene.expr', 'liver2-zy5.gene.expr'],
'lung':['lung1-zy10.gene.expr', 'lung2-zy11.gene.expr'],
'mESC':['mESC-zy27.gene.expr', 'mESC-zy28.gene.expr'],
'MEF':['mef-male1-zy17.gene.expr', 'mef-male2-zy18.gene.expr'],
'olfactory_bulb':['olfactory-1.expr', 'olfactory-2.expr'],
'placenta':['placenta-1.expr', 'placenta-2.expr'],
'spleen':['spleen1-zy8.gene.expr', 'spleen2-zy9.gene.expr'],
'testes':['testes-1.expr', 'testes-2.expr'],
'thymus':['thymus-1.expr', 'thymus-2.expr']}

df_med = pd.DataFrame()
```

```
for stage in stages.keys():
    df_med[stage] = df[stages[stage]].median(axis = 1)
```

./05. gene density analysis/rename_filter_transcripts.py

```
#! /usr/bin/env python3

import sys
import os
import re
import itertools
import argparse
import collections


parser = argparse.ArgumentParser(description = 'Take as input a chrom file, a gff
and a fasta of transcripts')
parser.add_argument('-c',
                    '--chrom',
                    help = 'chrom file',
                    required = True)

parser.add_argument('-a',
                    '--annot',
                    help = 'gff file. script works best if mitochondrial genomes
are out',
                    required = True)
parser.add_argument('-f',
                    '--fasta',
                    help = 'transcript fasta file',
                    required = True)
parser.add_argument('-ft',
                    '--feature_transcript',
                    help ='feature line where transcripts are mapped to parents',
                    type = str,
                    required = True)
parser.add_argument('-fp',
                    '--feature_protein',
                    help ='feature line where proteibns are mapped to parents',
                    type = str,
                    required = True)
parser.add_argument('-kpp',
                    '--key_parent_protein',
                    help ='gff key, where "key_parent=parent on lines yhere ye
look at protein"',
                    type = str,
                    required = True)
parser.add_argument('-kpt',
                    '--key_parent_transcript',
                    help ='gff key, where "key_parent=parent on lines yhere ye
```

```
look at protein"',
                    type = str,
                    required = True)
parser.add_argument('-kp',
                    '--key_protein_id',
                    help ='gff key, where "key_protein_id=accession"',
                    type = str,
                    required = True)
parser.add_argument('-kt',
                    '--key_transcript_id',
                    help ='gff key, where "key_transcript=accession"',
                    type = str,
                    required = True)
parser.add_argument('-o',
                    '--output',
                    help = 'prefix of the output file, default is prefix parsed
form the chrom. results saved in PREFIX.binsize.density',
                    default = False)
args = parser.parse_args()

def parse_chrom(handle):
    """
    :param handle: handle of the chrom file
    :returns: accession list, no prefix
    """
    output_list = []
    for line in handle:
        prefix, acc, *_ = line.rstrip().split('\t')
        finalacc = acc.replace(f'{prefix}_', '')
        output_list.append(finalacc)
    return output_list

def parse_fasta(handle):
    """
    parses fasta file
    :param handle: filehandle of a fasta file
    :return: a generator, pairs of header/sequence items. Sequence devoid of
newline characters.
    """
    fasta_iter = (list(g) for _,g in itertools.groupby(handle, lambda l:
l.startswith('>')))
    for header_group, sequence_group in zip(*[fasta_iter]*2):
        header_string = ''.join(header_group).lstrip('>').rstrip()
        header = ''.join(header_group).lstrip('>').rstrip().split()[0]
        seq = ''.join([line.rstrip() for line in sequence_group])
        yield header, seq

def parse_gff(handle):
    """
    parse gff file in to dict
    :param handle:
    :returns: dict transcript_id: protein_id
    """
    parent_transcript_dict = {}
```

```
    protein_parent_dict = {}
    re_key = '\s*[:="\']*([^,|;"\']+)[;|"\s\n]*'
    p_parent_re = args.key_parent_protein + re_key
    t_parent_re = args.key_parent_transcript + re_key
    transcript_re = args.key_transcript_id + re_key
    protein_re = args.key_protein_id + re_key
    for line in handle:
        if (line.startswith('#') or len(line.split('\t')) < 9):
            pass
        else:
            fields = line.rstrip().split('\t')
            _, _, feature, *_,comments = fields
            if feature == args.feature_transcript:
                try:
                    tran_id = re.search(transcript_re, comments).group(1)
                    parent_id = re.search(t_parent_re, comments).group(1)
                    parent_transcript_dict[parent_id] = tran_id
                except AttributeError:
                    pass
            elif feature == args.feature_protein:
                try:
                    prot_id = re.search(protein_re, comments).group(1)
                    parent_id = re.search(p_parent_re, comments).group(1)
                    protein_parent_dict[prot_id] = parent_id
                except AttributeError:
                    pass
    print(list(protein_parent_dict.items()))
    output_dict = {parent_transcript_dict[parent]:prot for prot, parent in
protein_parent_dict.items()}
    return output_dict

with open(args.annot, 'r') as annot:
    acc_dict = parse_gff(annot)

with open(args.chrom, 'r') as chrom:
    non_redundant_prots = parse_chrom(chrom)

with open(args.fasta, 'r') as fasta, open(args.output, 'w') as out:
    for header, seq in parse_fasta(fasta):
        try:
            header_prot = acc_dict[header]
            if header_prot in non_redundant_prots:
                out.write(f'>{header_prot}\n{seq}\n')
        except KeyError:
            pass
```

./05. gene density analysis/whole_genome_stats.py

```
#! /usr/bin/env python3

import argparse
import csv
import glob
import itertools
import os
import subprocess


parser = argparse.ArgumentParser(description = 'takes chom and genome folders, 
lists the files, returns a df with whole genome length gene counts and species')
parser.add_argument('-c', '--chrom', help = 'chrom folder', required = True)
parser.add_argument('-g', '--genome', help = 'genome folder', required = True)
parser.add_argument('-o', '--output', help = 'output name', required = True)
args = parser.parse_args()


def parse_fasta(handle):
    """
    parses fasta file
    :param handle: filehandle of a fasta file
    :return: a generator, pairs of header/sequence items. Sequence devoid of 
newline characters.
    """
    fasta_iter = (list(g) for _,g in itertools.groupby(handle, lambda l: 
l.startswith('>')))
    for header_group, sequence_group in zip(*[fasta_iter]*2):
        header_string = ''.join(header_group).lstrip('>').rstrip()
        header = ''.join(header_group).lstrip('>').rstrip().split()[0]
        seq = ''.join([line.rstrip() for line in sequence_group])
        yield header, seq


def count_file_lines(file_path):
    """
    Counts the number of lines in a file using wc utility.
    :param file_path: path to file
    :return: int, no of lines
    """
    num = subprocess.check_output(['wc', '-l', file_path])
    num = num.split()
    return int(num[0])



chrom_ls = sorted(glob.glob(f'{args.chrom}/*chrom'))
genome_ls = sorted([x for x in glob.glob(f'{args.genome}/*genome') if 
```

```
os.path.isdir(x) is False])

print(chrom_ls)

print(genome_ls)

results = [['species', 'genome_length', 'gene_count']]
for chrom, genome in zip(chrom_ls, genome_ls):
    prefix = os.path.basename(chrom).split('.')[0]
    print(f'prefix is: {prefix}')
    genome_length = 0
    print(f'parsing genome file {genome}...')
    with open(genome) as f:
        for _, seq in parse_fasta(f):
            genome_length += len(seq)
    print(f'Done!')
    print(f'parsing chrom file {genome}...')
    print(f'Done!')
    gene_count = count_file_lines(chrom)
    results.append([prefix, genome_length, gene_count])

with open(f'{args.output}.csv', 'w', newline='') as csvfile:
    writer = csv.writer(csvfile)
    writer.writerows(results)
```

./06. GO analysis/enrichment_comparisons.py

```
#!/usr/bin/env python3


import os
import pandas as pd
import glob

taxo_dict =
{'ACRMI':'Cnidarian','ADIVA':'Lophotrochozoan','AMPQU':'Poriferan','ACAPL':'InvDeu
t','ANOGA':'Ecdysozoan','AURAU':'Cnidarian','BRALA':'InvDeut','CAEEL':'Ecdysozoan'
,'CALMI':'Vertebrate','CAPTE':'Lophotrochozoan','CAPOW':'Metazoa_outgroup','CHEMY'
:'Vertebrate','CIOIN':'InvDeut','CLYHE':'Cnidarian','CRAGI':'Lophotrochozoan','DAN
RE':'Vertebrate','DAPPU':'Ecdysozoan','DROME':'Ecdysozoan','EUPSC':'Lophotrochozoa
n','EXAPA':'Cnidarian','GALGA':'Vertebrate','HELRO':'Lophotrochozoan','HIPCO':'Ver
tebrate','HOFMI':'Acoel','HOIHO':'Placozoan','HOMSA':'Vertebrate','HYDVU':'Cnidari
an','IXOSC':'Ecdysozoan','LATCH':'Vertebrate','LEPOC':'Vertebrate','LINAN':'Lophot
rochozoan','LOTGI':'Lophotrochozoan','MAYZE':'Vertebrate','MIZYE':'Lophotrochozoan
','MNELE':'Ctenophore','MUSMU':'Vertebrate','NEMVE':'Cnidarian','PARTE':'Ecdysozoa
n','PLEBA':'Ctenophore','PTYFL':'InvDeut','SACKO':'InvDeut','SALRO':'Metazoa_outgr
oup','SCHME':'Lophotrochozoan','STRMA':'Ecdysozoan','STRPU':'InvDeut','SYCCI':'Por
iferan','TRICA':'Ecdysozoan','TRIAD':'Placozoan','XENTR':'Vertebrate'}
filelist_meta = glob.glob('Metazoa/*.xlsx')
```

```
filelist_planu = glob.glob('Planulozoa/*.xlsx')
filelist_bila = glob.glob('Bilateria/*.xlsx')
taxonomy_ls = ['Metazoa_outgroup', 'Ctenophore', 'Poriferan', 'Placozoan',
'Cnidarian', 'Acoel', 'Ecdysozoan', 'Lophotrochozoan', 'InvDeut', 'Vertebrate']

def make_dicts(filelist):
    GO_dict = {}
    GO_strings = {}
    for file in filelist:
        df = pd.read_excel(file)
        species = os.path.basename(file).split('_')[0]
        df_enriched = df.query("enrichment =='e'")
        GO_ls = list(df_enriched.get('GO'))
        for GO in GO_ls:
            if GO in GO_dict.keys():
                GO_dict[GO].append(species)
            else:
                GO_dict[GO] = [species]
                GO_strings[GO] = df_enriched.query('GO == @GO').iloc[0]['name']
    return GO_dict, GO_strings

#Metazoa terms
GO_dict_meta, GO_strings_meta = make_dicts(filelist_meta)

basal_metazoan = ['Ctenophore','Poriferan']
other_metazoans = ['Placozoan', 'Cnidarian', 'Acoel', 'Ecdysozoan',
'Lophotrochozoan', 'InvDeut', 'Vertebrate']

GO_ls_bytaxo = []
for GO,species_ls in GO_dict_meta.items():
    taxo_ls = [taxo_dict[species] for species in species_ls]
    nb_basal_in_taxon = len([species for species in taxo_ls if species in
basal_metazoan])
    nb_other_in_taxon = len([species for species in taxo_ls if species in
other_metazoans])
    total_nb_species_with_GO = len(species_ls)
    if nb_basal_in_taxon > 0 and nb_other_in_taxon > 0 and
total_nb_species_with_GO >= 8:
        GO_ls_bytaxo.append([GO, GO_strings_meta[GO], ','.join(species_ls)])

with open('Metazoa_report_GO_enriched.tsv', 'w') as f:
    f.write(pd.DataFrame(GO_ls_bytaxo, columns = ['GO', 'name', 'species
list']).to_csv(sep = '\t', index = False))

#Planulozoa novel terms
GO_dict_planu, GO_strings_planu = make_dicts(filelist_planu)

Cnidaria = ['Cnidarian']
Bilateria = ['Acoel', 'Ecdysozoan', 'Lophotrochozoan', 'InvDeut', 'Vertebrate']
GO_ls_bytaxo = []
for GO,species_ls in GO_dict_planu.items():
        taxo_ls = [taxo_dict[species] for species in species_ls]
        nb_Cnidaria_in_taxon = len([species for species in taxo_ls if species in
Cnidaria])
```

```
        nb_Bilateria_in_taxon = len([species for species in taxo_ls if species in
Bilateria])
        total_nb_species_with_GO = len(species_ls)
        if nb_Cnidaria_in_taxon >= 3 and nb_Bilateria_in_taxon >= 8:
                GO_ls_bytaxo.append([GO, GO_strings_planu[GO],
','.join(species_ls)])

GO_ls_bytaxo = list(sorted(GO_ls_bytaxo, key = lambda x:x[2]))

with open('Planu_report_GO_enriched.tsv', 'w') as f:
        f.write(pd.DataFrame(GO_ls_bytaxo, columns = ['GO', 'name', 'species
list']).to_csv(sep = '\t', index = False))

#Bilateria novel terms
GO_dict_planu, GO_strings_planu = make_dicts(filelist_bila)

protostomia = ['Ecdysozoan', 'Lophotrochozoan']
deuterostomia = ['InvDeut', 'Vertebrate']
GO_ls_bytaxo = []
for GO,species_ls in GO_dict_planu.items():
    taxo_ls = [taxo_dict[species] for species in species_ls]
    nb_protostomia_in_taxon = len([species for species in taxo_ls if species in
protostomia])
    nb_deuterostomia_in_taxon = len([species for species in taxo_ls if species in
deuterostomia])
    total_nb_species_with_GO = len(species_ls)
    if nb_protostomia_in_taxon >= 4 and nb_deuterostomia_in_taxon >= 4:
        GO_ls_bytaxo.append([GO, GO_strings_planu[GO], ','.join(species_ls)])

GO_ls_bytaxo = list(sorted(GO_ls_bytaxo, key = lambda x:x[2]))

with open('Bila_report_GO_enriched.tsv', 'w') as f:
    f.write(pd.DataFrame(GO_ls_bytaxo, columns = ['GO', 'name', 'species
list']).to_csv(sep = '\t', index = False))
```

./06. GO analysis/GO_enrichment.py

```
#!/usr/bin/env python3

import argparse
import pandas as pd
from goatools.obo_parser import GODag
from goatools.anno.idtogos_reader import IdToGosReader
from goatools.goea.go_enrichment_ns import GOEnrichmentStudyNS
from goatools.godag_plot import plot_gos, plot_results, plot_goid2goobj


parser = argparse.ArgumentParser(description = """Plots goterms enriched GO terms.
Pvalue of 0,01, BH procedure correction for multiple testing.
```

```
    Will test all 3 namespaces (Biological Process, Molecular Function, Cellular
Compartment""")
parser.add_argument('-i', '--ids2go', help = 'GO annotation, ids2go. Two fields,
first one is accessions, second one is semicolon-delimited lis of GO annotations.
', required = True)
parser.add_argument('-go', '--obo', help = 'obo file to use in the GO enrichment
analysis', required = True)
parser.add_argument('-s', '--study', help = 'list of sequences from the study,
newline-delimited', required = True)
parser.add_argument('-b', '--background', help = 'List of the background
sequences, i.e. all the sequences from the animal, newline-delimited', required =
True)
parser.add_argument('-p', '--propagate_counts', help = 'propagate GO_counts,
default: False', action = 'store_true')
parser.add_argument('-o', '--output', help = 'Name of the output filename prefix')
args = parser.parse_args()


def file2list(sequences_list):
    acc_ls = []
    with open(sequences_list, 'r') as f:
        for line in f:
            acc = line.rstrip()
            acc_ls.append(acc)
    return acc_ls

if args.output is None:
    output_fname = f'{args.study.split(".")[0]}_GOAE'
else:
    output_fname = args.output

#Import the GO hierarchy, downloaded from http://geneontology.org/ontology/go-
basic.obo
obodag = GODag(args.obo)

# Read ids2go format. Store annotations in a list of named tuples. Specify obo
graph to use
objanno = IdToGosReader(args.ids2go, godag = obodag)

"""
Get namespace2association. Basically, a nested dict as follows:
    ns2assoc[namespace][association]: GO
    namespace is: BP (biological_process), MF (molecular_function), CC
(cellular_component)
    assocation is: a protein_id
    GO is: the set of GO IDs associated with that protein_ID
"""
ns2assoc = objanno.get_ns2assc()

background = file2list(args.background)

goeaobj = GOEnrichmentStudyNS(
        background, # List of background proteins
        ns2assoc, # geneid/GO associations
```

```
        obodag, # Ontologies
        propagate_counts = args.propagate_counts,
        alpha = 0.05, # we'll change the pvalue cut-off to 0.01
        methods = ['fdr_bh']) # defult multipletest correction method

study_list = file2list(args.study)

# 'p_' means "pvalue". 'fdr_bh' is the multiple test correction, Benjamini-
Hochberg.
goea_results_all = goeaobj.run_study(study_list)
goea_results_sig = [r for r in goea_results_all if r.p_fdr_bh < 0.05]

goeaobj.wr_xlsx(f'{output_fname}.xlsx', goea_results_sig)
goeaobj.wr_txt(f'{output_fname}.txt', goea_results_sig)

#Isolate only the significant GOs we use the attribute GO of the
GOEnrichmentRecord object located in the significant goea_results
GO_list = [GOEnrichmentRecord.GO for GOEnrichmentRecord in goea_results_sig]

#This'll make one single plot with the 3 GO namespaces.
plot_gos(f'{output_fname}.pdf',
    GO_list, # Source GO ids
    obodag,
    goea_results = goea_results_sig) # Use pvals for coloring
```

./07. Block correlation/pairwise_corr_analysis.py

```
#! /usr/bin/env python3


import pandas as pd
import numpy as np
import scipy.stats
import itertools
import collections
import argparse

parser = argparse.ArgumentParser(description = 'takes a tidy dataframe as input,
made with make_tidy_density_df.py, outputs block corr and bp distance between
every 2 genes.')
parser.add_argument('-t', '--results_df', help = 'tidy dataframe, one observation
by line', required = True)
parser.add_argument('-s', '--full_synt_file', help = 'original (unfiltered synt
file)', required = True)
parser.add_argument('-og', '--orthology', help = 'orthology file, clus format',
required = True)
parser.add_argument('-e', '--expression_data', help = 'full tpm table')
parser.add_argument('-c', '--chrom', help = 'prefix to add to accessions in
expression data')
```

```
parser.add_argument('-o', '--output', help = 'output prefix', required = True)

args = parser.parse_args()

"""
tmp_args = collections.namedtuple('args', ['results_df', 'full_synt_file',
'expression_data', 'chrom','orthology'])

args =
tmp_args('../../02_REDUX_gene_density_analysis/density_whole_genome/key_nodes.tidy
df.csv',
                 '../../01_microsynteny/chrom_of/5.blocks.3.syn_corrected.synt',

'/scratch/robert/2019_12_Neuropeptides/TPM_normalizations/CRAGI/CRAGI_transcript_t
pms_all_samples.tsv',
                 '../../01_microsynteny/chrom/CRAGI.chrom',
                 '../../01_microsynteny/Orthofinder.clus')
"""

def load_expr(expr, prefix):
    """
    load expression data
    unexpressed genes (0 tpms in all stages) are deleted
    :param expr: expression table, first line is header, firts column is accession
    second column length, rest of fields are expression by stage
    """
    output_dict = {}
    with open(expr, 'r') as f:
        header = f.readline()
        for line in f:
            transcript_id, _, *tpms = line.rstrip().split() #second column is
length
            tpms = np.array([np.float64(x) for x in tpms])
            if prefix != '':
                transcript_id = f'{prefix}_{transcript_id}'
            if tpms.max() == 0:
                pass
            else:
                output_dict[transcript_id] = tpms
    return output_dict


def cleanup_acc_ls(acc_str, exp_dict):
    """
    deletes acc for acc ls if they're not in the filtered exp dict
    :param acc_ls: a list of accessions, as strings, comma separated
    :param exp_dict: expresison dictionary, output of exp_dict
    :returns: filtered acc_ls (as list object)
    """
    acc_ls = acc_str.split(',')
    to_remove = []
    for gene in acc_ls:
        if exp_dict.get(gene) is None:
            to_remove.append(gene)
```

```
    acc_ls = list(set(acc_ls) - set(to_remove))
    return acc_ls


def load_ortho(ortho):
    """
    load orthology
    :param ortho: Orthofinder output, clus format
    :returns: dict, acc as keys, OG as values
    """
    output_dict = {}
    with open(ortho, 'r') as f:
        for line in f:
            og, _, *acc_ls = line.rstrip().split('\t')
            for acc in acc_ls:
                output_dict[acc] = og
    return output_dict


order = ['Vertebrate', 'Tunicate', 'Cephalochordate', 'Ambulacrarian',
'Lophotrochozoan', 'Ecdysozoan', 'Acoel', 'Cnidarian', 'Placozoan', 'Ctenophore',
'Poriferan', 'Metazoa_outgroup']
species_dict = {'Vertebrate' :
['HOMSA','MUSMU','CHEMY','GALGA','XENTR','LATCH','MAYZE','HIPCO','DANRE','LEPOC','
CALMI'],
                'Tunicate': ['CIOIN'],
                'Cephalochordate' : ['BRALA'],
                'Ambulacrarian' : ['SACKO','PTYFL','STRPU','ACAPL'],
                'Lophotrochozoan' : ['CAPTE',
'EUPSC','LOTGI','MIZYE','CRAGI','HELRO','ADIVA','LINAN','SCHME'],
                'Ecdysozoan' :
['DROME','ANOGA','TRICA','DAPPU','STRMA','IXOSC','PARTE','CAEEL'],
                'Acoel' : ['HOFMI'],
                'Cnidarian' : ['NEMVE','EXAPA','ACRMI','HYDVU','CLYHE','AURAU'],
                'Placozoan' : ['HOIHO','TRIAD'],
                'Ctenophore' : ['PLEBA','MNELE'],
                'Poriferan' : ['SYCCI','AMPQU'],
                'Metazoa_outgroup' : ['SALRO','CAPOW'],
}


order_sp = [species for taxon in order for species in species_dict[taxon]]



results_df = pd.read_csv(args.results_df)

chrom_df = pd.read_csv(args.chrom, sep = '\t', names = ['prefix', 'accession',
'chromosome', 'strand', 'start', 'end'])
prefix = chrom_df.prefix.values[0]
ortho_dict = load_ortho(args.orthology)
exp_dict = load_expr(args.expression_data, prefix)

full_df = pd.read_csv(args.results_df)
```

```
species_df = full_df.query('species == @prefix')

with open(args.output, 'w') as g:
    g.write(f'genea\tgeneb\tblock_id\tnode\tpara\tcorr\tdist\trandom\n')
    for row in species_df.values.tolist():
        node, _, species, random, block_id, _, _, acc_str, *_ = row
        acc_ls = cleanup_acc_ls(acc_str, exp_dict)
        for genea, geneb in itertools.combinations(acc_ls,2):
            exp_genea = exp_dict[genea]
            exp_geneb = exp_dict[geneb]
            coords_df = chrom_df.query('accession in [@genea, @geneb]')[['start',
'end']]
            coords_ls = sorted([int(y) for x in coords_df.values for y in x])
            corr = scipy.stats.spearmanr(exp_genea, exp_geneb).correlation
            dist = coords_ls[2] - coords_ls[1]
            if ortho_dict[genea] == ortho_dict[geneb]:
                para = 'para'
            else:
                para = 'not_para'

g.write(f'{genea}\t{geneb}\t{block_id}\t{node}\t{para}\t{corr}\t{dist}\t{random}\n
')
```

./07. Block correlation/rename_filter_transcripts.py

```
#! /usr/bin/env python3

import sys
import os
import re
import itertools
import argparse
import collections


parser = argparse.ArgumentParser(description = 'Take as input a chrom file, a gff
and a fasta of transcripts')
parser.add_argument('-c',
                    '--chrom',
                    help = 'chrom file',
                    required = True)

parser.add_argument('-a',
                    '--annot',
                    help = 'gff file. script works best if mitochondrial genomes
are out',
                    required = True)
```

```
parser.add_argument('-f',
                    '--fasta',
                    help = 'transcript fasta file',
                    required = True)
parser.add_argument('-ft',
                    '--feature_transcript',
                    help ='feature line where transcripts are mapped to parents',
                    type = str,
                    required = True)
parser.add_argument('-fp',
                    '--feature_protein',
                    help ='feature line where proteibns are mapped to parents',
                    type = str,
                    required = True)
parser.add_argument('-kpp',
                    '--key_parent_protein',
                    help ='gff key, where "key_parent=parent on lines yhere ye
look at protein"',
                    type = str,
                    required = True)
parser.add_argument('-kpt',
                    '--key_parent_transcript',
                    help ='gff key, where "key_parent=parent on lines yhere ye
look at protein"',
                    type = str,
                    required = True)
parser.add_argument('-kp',
                    '--key_protein_id',
                    help ='gff key, where "key_protein_id=accession"',
                    type = str,
                    required = True)
parser.add_argument('-kt',
                    '--key_transcript_id',
                    help ='gff key, where "key_transcript=accession"',
                    type = str,
                    required = True)
parser.add_argument('-o',
                    '--output',
                    help = 'prefix of the output file, default is prefix parsed
form the chrom. results saved in PREFIX.binsize.density',
                    default = False)
args = parser.parse_args()

def parse_chrom(handle):
    """
    :param handle: handle of the chrom file
    :returns: accession list, no prefix
    """
    output_list = []
    for line in handle:
        prefix, acc, *_ = line.rstrip().split('\t')
        finalacc = acc.replace(f'{prefix}_', '')
        output_list.append(finalacc)
    return output_list
```

```
def parse_fasta(handle):
    """
    parses fasta file
    :param handle: filehandle of a fasta file
    :return: a generator, pairs of header/sequence items. Sequence devoid of
newline characters.
    """
    fasta_iter = (list(g) for _,g in itertools.groupby(handle, lambda l:
l.startswith('>')))
    for header_group, sequence_group in zip(*[fasta_iter]*2):
        header_string = ''.join(header_group).lstrip('>').rstrip()
        header = ''.join(header_group).lstrip('>').rstrip().split()[0]
        seq = ''.join([line.rstrip() for line in sequence_group])
        yield header, seq

def parse_gff(handle):
    """
    parse gff file in to dict
    :param handle:
    :returns: dict transcript_id: protein_id
    """
    parent_transcript_dict = {}
    protein_parent_dict = {}
    re_key = '\s*[:="\']*([^,|;"\']+)[;|"\s\n]*'
    p_parent_re = args.key_parent_protein + re_key
    t_parent_re = args.key_parent_transcript + re_key
    transcript_re = args.key_transcript_id + re_key
    protein_re = args.key_protein_id + re_key
    for line in handle:
        if (line.startswith('#') or len(line.split('\t')) < 9):
            pass
        else:
            fields = line.rstrip().split('\t')
            _, _, feature, *_,comments = fields
            if feature == args.feature_transcript:
                try:
                    tran_id = re.search(transcript_re, comments).group(1)
                    parent_id = re.search(t_parent_re, comments).group(1)
                    parent_transcript_dict[parent_id] = tran_id
                except AttributeError:
                    pass
            elif feature == args.feature_protein:
                try:
                    prot_id = re.search(protein_re, comments).group(1)
                    parent_id = re.search(p_parent_re, comments).group(1)
                    protein_parent_dict[prot_id] = parent_id
                except AttributeError:
                    pass
    print(list(protein_parent_dict.items()))
    output_dict = {parent_transcript_dict[parent]:prot for prot, parent in
protein_parent_dict.items()}
    return output_dict
```

```
with open(args.annot, 'r') as annot:
    acc_dict = parse_gff(annot)

with open(args.chrom, 'r') as chrom:
    non_redundant_prots = parse_chrom(chrom)

with open(args.fasta, 'r') as fasta, open(args.output, 'w') as out:
    for header, seq in parse_fasta(fasta):
        try:
            header_prot = acc_dict[header]
            if header_prot in non_redundant_prots:
                out.write(f'>{header_prot}\n{seq}\n')
        except KeyError:
            pass
```

./09. Graph analysis/Block_to_OGcommus.py

```
#! /usr/bin/env python3

import sys
import collections
import itertools
import glob
import argparse
import statistics

parser = argparse.ArgumentParser(description = 'Provides reports of block content
of specified noppdes')
parser.add_argument("-c", "--clusters_id", help = "Tsv file, the multi-species
clusters of microsyntenic blocks, output of the microsynteny pipeline
(makeClusters3.pl).", required = True)
parser.add_argument("-b", "--block_list", help = "Tsv file, microsyntenic blocks
details, output of the microsynteny pipeline (makeClusters3.pl).", required =
True)
parser.add_argument("-r", "--random_blocks", help = "Tsv file, microsyntenic
blocks details, output of Bob's script, corrected to fit the same format as the
observed blocks", required = True)
parser.add_argument("-g", "--chromfiles", help = "List of the chromfiles to use",
type = str, required = True, nargs = "+")
parser.add_argument("-og", "--orthology", help = "Orthology file, clus format",
type = str, required = True)
parser.add_argument("-o", "--output", help = "Prefix of the output files.", type =
```

```
str, default = "output")
parser.add_argument("--custom_orthology", help = "use flag if custom_orthology.",
action = "store_true")
args = parser.parse_args()


def nested_dict():
    """ Create a nested dict. Function is picklable """
    return collections.defaultdict(nested_dict)


def read_chromfiles(chromfiles_list, orthology_dict):
    """
    Reads a list of files in the .chrom format
    Returns a nested dict as follows:
    [OG] [Species] [chromosome/scaffold] [accession] [coordinates]
    Coordinates is a tuple of len 3:
        (position on the chrom (1-based) , start, stop)
    Second dict will just contain accessions
    if args.custim_orthology is true, all the values will be stored in the chrom
    if it's false, orthology will be used to filter the coordinates
    """
    output_dict = nested_dict()
    accession_pos_dict = {}
    species_ls = [name.split('/')[-1].split('.')[0] for name in chromfiles_list]
    for file in chromfiles_list:
        species = file.split('/')[-1].split('.')[0]
        sys.stderr.write(f'Reading file {file}\n')
        tmp_coords = nested_dict()
        with open(file, 'r') as chrom_file:
            for line in chrom_file:
                _, accession, scaffold, _, start, stop =
line.rstrip(None).split('\t')
                if args.custom_orthology is True:
                    tmp_coords[scaffold][accession] = [start,stop]
                elif accession in orthology_dict.keys():
                    tmp_coords[scaffold][accession] = [start,stop]
        for chromosome in tmp_coords.keys():
            chrsort = sorted((tmp_coords[chromosome]).keys(),key = lambda x:
int((tmp_coords[chromosome])[x][0]))
            pos = 0
            for gene in chrsort:
                pos = pos + 1
                if args.custom_orthology is False:
                    output_dict[orthology_dict[gene]][species][chromosome][gene] =
[pos] + tmp_coords[chromosome][gene]
                else:
                    try:
                        output_dict[orthology_dict[gene]][species][chromosome]
[gene] = [pos] + tmp_coords[chromosome][gene]
                    except KeyError:
                        output_dict['other'][species][chromosome][gene] = [pos] +
tmp_coords[chromosome][gene]
                accession_pos_dict[gene] = [pos] + tmp_coords[chromosome][gene]
```

```
    sys.stderr.write('Chroms loaded!\n')
    return output_dict, species_ls, accession_pos_dict


def block_id_dict(handle, og_dictionary):
    """
    returns a dict with block id as keys,
    named tuples as values: species, chromosome, acc_ls, og_pairs
    og_pairs are the OG_pairs found in a given block.
    another dict is the reverse dict (acc as keys, block as values)
    aother dict is the index of the accession within the block (used for
determinining random dists)
    """
    output_dict = {}
    output_dict_acc = {}
    output_dict_acc_index = {}
    block = collections.namedtuple('block', 'species accessions chromosome
og_pairs')
    for line in handle:
        line = line.rstrip().split()
        block_id = line[0]
        species = line[1]
        chromosome = line[7].split(':')[0]
        acc_ls = line[9].split(',')
        og_pairs = set([frozenset([og_dictionary[x],og_dictionary[y]]) for x,y in
itertools.combinations(acc_ls, 2)])
        output_dict[block_id] = block(species, acc_ls, chromosome, og_pairs)
        for x in  range(0,len(acc_ls)):
            acc = acc_ls[x]
            output_dict_acc[acc] = block_id
            output_dict_acc_index[acc] = x
    return output_dict, output_dict_acc, output_dict_acc_index

def rand_blocks_positions(handle, accession_pos_dict):
    output_dict = {}
    for line in handle:
        line = line.rstrip().split()
        block_id, iteration_number = line[0].split('.')
        acc_ls = line[9].split(',')
        if iteration_number == "1":
            for x in range(0, len(acc_ls)):
                pos, start, end = [int(x) for x in accession_pos_dict[acc_ls[x]]]
                output_dict[frozenset({block_id, x})] = [[pos],[start],[end]]
        else:
            for x in range(0, len(acc_ls)):
                pos, start, end = [int(x) for x in accession_pos_dict[acc_ls[x]]]
                output_dict[frozenset({block_id, x})][0].append(pos)
                output_dict[frozenset({block_id, x})][1].append(start)
                output_dict[frozenset({block_id, x})][2].append(end)
    return output_dict

#nested lists frozenset{block_string, index} [[pos,pos][starts, start][end,end]]

def og_dict(handle):
```

```
    """
    dict with OGs or accessions as keys
    accession return OGs,
    OGs returns acc_ls
    """
    output_dict = {}
    for line in handle:
        line = line.rstrip().split()
        OG = line[0]
        for acc in line[2:]:
            output_dict[acc] = OG
        output_dict[OG] = line[2:]
    return output_dict

def clus_id_dict(handle):
    """
    dict
    """
    clus_id_dict = {}
    for line in handle:
        line = line.rstrip().split()
        clus_id_dict[line[0]] = line[1:]
    return clus_id_dict




with open(args.orthology, 'r') as filehandle:
    OG_d = og_dict(filehandle)

with open(args.clusters_id, 'r') as filehandle:
    clus_d = clus_id_dict(filehandle)

with open(args.block_list) as filehandle:
    block_d, acc_d, acc_index_d = block_id_dict(filehandle, OG_d)

chrom_dict, total_sp_ls, accession_pos_coords_d =
read_chromfiles(args.chromfiles,OG_d)

class SpeciesDist(object):
    """
    this is just to have a fixed length list of items created in each dictionary
entry in results_dict
    We'll populate it with NA at the start
    And if the OG pair is found in the cluster, we'll change this value to the
actual distance
    """
    __slots__ = total_sp_ls
    def __init__(self):# we'll declare values as NAs in all attributes after
creating the instance
        pass

with open(args.random_blocks) as filehandle:
    rand_blocks_positions_dict = rand_blocks_positions(filehandle,
```

```
accession_pos_coords_d)

dist_dict, bp_dict, dist_dict_rand, bp_dict_rand, OG_commu_dict = {}, {}, {}, {},
{}

for multi_sp_block in clus_d.keys():
    species_ls = [block_d[block].species for block in clus_d[multi_sp_block]]
    acc_ls_total = itertools.chain.from_iterable([block_d[block].accessions for
block in clus_d[multi_sp_block]])
    OG_commu = list(set([OG_d[acc] for acc in acc_ls_total]))
    OG_commu_dict[multi_sp_block] = OG_commu
    for species in species_ls:
        og_pair_set_sp =
set(itertools.chain.from_iterable([block_d[block].og_pairs for block in
clus_d[multi_sp_block] if block_d[block].species == species]))
        acc_ls_set_sp =
list(itertools.chain.from_iterable([block_d[block].accessions for block in
clus_d[multi_sp_block] if block_d[block].species == species]))
        for pair_fzset in og_pair_set_sp:
            if len(pair_fzset) == 1: #OG to self
                og1,og2 = list(pair_fzset)*2
            else:
                og1,og2 = pair_fzset
            key_string_ogpair = f'{multi_sp_block}_{og1}_{og2}'
            og1_filt = [acc for acc in OG_d[og1] if acc in acc_ls_set_sp]
            og2_filt = [acc for acc in OG_d[og2] if acc in acc_ls_set_sp]
            acc_pairs = [[x,y] for x in og1_filt for y in og2_filt if x !=y and
acc_d[x] == acc_d[y]]
            for mydict in dist_dict, bp_dict, dist_dict_rand, bp_dict_rand:
                try:
                    mydict[key_string_ogpair]
                except KeyError: #if key not in the dict, we initialize it
                    mydict[key_string_ogpair] = SpeciesDist()
                    for item in total_sp_ls: #initialize Species_dist object with
NAs, as we iterate only through the existing OG pairs in species
                        setattr(mydict[key_string_ogpair], item, 'NA')
            for gene1,gene2 in acc_pairs:
                block_id_pair = acc_d[gene1]
                chromosome = block_d[block_id_pair].chromosome
                pos1, start1, end1 = [int(x) for x in chrom_dict[og1][species]
[chromosome][gene1]]
                pos2, start2, end2 = [int(x) for x in chrom_dict[og2][species]
[chromosome][gene2]]
                overlap_genes = min(start2,end2) <= max(start1, end1) and
max(start2,end2) >= min(start1, end1)
                if overlap_genes is True:
                    dist_bp = 0
                else:
                    dist_bp = min(abs(end1 - start2), abs(end1 - end2), abs(start1
- start2), abs(start1 - end2))
                dist_genes = abs(pos2 - pos1)
                gene1_index, gene2_index = acc_index_d[gene1],acc_index_d[gene2]
                pos1_r_ls, start1_r_ls, end1_r_ls =
rand_blocks_positions_dict[frozenset({block_id_pair,gene1_index})]
```

```
                pos2_r_ls, start2_r_ls, end2_r_ls =
rand_blocks_positions_dict[frozenset({block_id_pair,gene2_index})]
                dist_gene_rand = statistics.median([abs(pos2_r - pos1_r) for
pos1_r, pos2_r in zip(pos1_r_ls, pos2_r_ls)])
                dist_bp_rand_ls = []
                for start1, end1, start2, end2 in zip(start1_r_ls, end1_r_ls,
start2_r_ls, end2_r_ls):
                    overlap_genes = min(start2,end2) <= max(start1, end1) and
max(start2,end2) >= min(start1, end1)
                    if overlap_genes is True:
                        dist_bp_rand_ls.append(0)
                    else:
                        dist_bp_rand_ls.append(min(abs(end1 - start2), abs(end1 -
end2), abs(start1 - start2), abs(start1 - end2)))
                dist_bp_rand = statistics.median(dist_bp_rand_ls)
                if getattr(dist_dict[key_string_ogpair], species) == 'NA' or
getattr(dist_dict[key_string_ogpair], species) > dist_genes:
                    setattr(dist_dict[key_string_ogpair], species, dist_genes)
                    setattr(dist_dict_rand[key_string_ogpair], species,
dist_gene_rand)
                if getattr(bp_dict[key_string_ogpair], species) == 'NA' or
getattr(bp_dict[key_string_ogpair], species) > dist_bp:
                    setattr(bp_dict[key_string_ogpair], species, dist_bp)
                    setattr(bp_dict_rand[key_string_ogpair], species,
dist_bp_rand)


with open(f'{args.output}.OG_commus', 'w') as f:
    for cluster_id, og_commu in OG_commu_dict.items():
        f.write(cluster_id + '\t' + '\t'.join(og_commu) + '\n')

with open(f'{args.output}.dist', 'w') as f:
    f.write('OG1\tOG2\t' + '\t'.join(total_sp_ls) + '\n')
    for key, value in dist_dict.items():
        multi_sp_block, OG1, OG2 = key.split('_')
        dist_ls_string = '\t'.join([str(getattr(dist_dict[key], species)) for
species in total_sp_ls])
        f.write(f'{multi_sp_block}\t{OG1}\t{OG2}\t{dist_ls_string}\n')

with open(f'{args.output}.bp', 'w') as f:
    f.write('OG1\tOG2\t' + '\t'.join(total_sp_ls) + '\n')
    for key, value in bp_dict.items():
        multi_sp_block, OG1, OG2 = key.split('_')
        dist_ls_string = '\t'.join([str(getattr(bp_dict[key], species)) for
species in total_sp_ls])
        f.write(f'{multi_sp_block}\t{OG1}\t{OG2}\t{dist_ls_string}\n')

with open(f'{args.output}.rand.dist', 'w') as f:
    f.write('OG1\tOG2\t' + '\t'.join(total_sp_ls) + '\n')
    for key, value in dist_dict_rand.items():
        multi_sp_block, OG1, OG2 = key.split('_')
        dist_ls_string = '\t'.join([str(getattr(dist_dict_rand[key], species)) for
species in total_sp_ls])
        f.write(f'{multi_sp_block}\t{OG1}\t{OG2}\t{dist_ls_string}\n')
```

```
with open(f'{args.output}.rand.bp', 'w') as f:
    f.write('OG1\tOG2\t' + '\t'.join(total_sp_ls) + '\n')
    for key, value in bp_dict_rand.items():
        multi_sp_block, OG1, OG2 = key.split('_')
        dist_ls_string = '\t'.join([str(getattr(bp_dict_rand[key], species)) for
species in total_sp_ls])
        f.write(f'{multi_sp_block}\t{OG1}\t{OG2}\t{dist_ls_string}\n')
```
